# Supplementary material for: Information, certainty, and learning
Source: eLife. 2026 Feb 2;13:RP102155. doi: 10.7554/eLife.102155 (PMC12863717; doi:10.7554/eLife.102155)
Supplement: Supplementary file 1. [file elife-102155-supp1.pdf]

# Supplementary Materials

## Data plots for each individual rat

*Each 6-panel figure plots data from one of the 176 rats. The rat number and the CS informativeness (ι), based on the C/T ratio, are shown above the first panel in the top left of the figure.*

***Panel 1** plots the rat's mean response rate (number of pokes per second) during the CS and during the pre-CS period in the inter-trial interval (ITI) on each of the 42 conditioning sessions.*
***Panel 2** (top right) shows on each trial the rat's response rate during the CS (dark grey line) and during the pre-CS period (light grey line). On the same panel, the blue line shows the cumulative poke count during the CS plotted against the cumulative CS duration across trials. (For this, the cumulative poke count excluded the first response in each trial, and the cumulative CS duration excluded the latency to first poke and the time the rat was in the magazine.)*
***Panels 3 and 4** (middle row) plot the cumulative response rates during the CS (solid black line), during the ITI (dashed black line), and the overall response rate in the context (CS plus ITI; dotted black line) across trials. The thick red line plots the nDkl for the comparison between CS rate and overall (context) rate. In Panel 3 (left), response rates were calculated in the conventional manner, as the cumulative number of responses divided by total time. In Panel 4 (right), response rates were calculated as the cumulative number of responses excluding the first response in each CS divided by the remaining time out of the magazine (i.e., excluding the latency to $1^{st}$ response in the CS and excluding the cumulative time in the magazine). The black vertical line marks the trial on which the cumulative CS response rate permanently exceeded the cumulative context response rate. The two red dashed vertical lines to the right of the black line mark the trial on which the nDkl reached 0.82 (Odds 4:1 that CS rate > Context rate) and 1.92 (p < .05 that CS rate = Context rate). Note that these latter two values for the nDkl were calculated starting from the trial on which the CS rate permanently exceeded the context rate (marked by the black vertical line) and therefore do not correspond to the nDkl values calculated from Trial 1 shown by the thick red plotted line. The unbroken red vertical line marks the trial when the nDkl was minimum. To make the initial changes in responding clearer, the x-axis of each plot is truncated at the trial number 1.5 times the trial number at which the p<.05 threshold was reached (e.g., if the nDkl reached p<.05 at Trial 100, the axis is truncated at trial 150), or after a minimum of 20 trials.*
***Panels 5 and 6** (bottom row) show the parsed estimates of the CS response rate and pre-CS response rate estimated from the response rates as described for Panel 4. In both panels, the vertical red lines mark estimates of acquisition based on the $nD_{KL}$ as the Earliest estimate (leftmost), and when the odds against the null hypothesis that the parsed CS and ITI rates were equal reached 4:1, 10:1, 20:1, and 100:1. (The x-axis in Panel 5 has been right-cropped 20 trials after the difference in parsed response rates reached Odds 20:1.)*

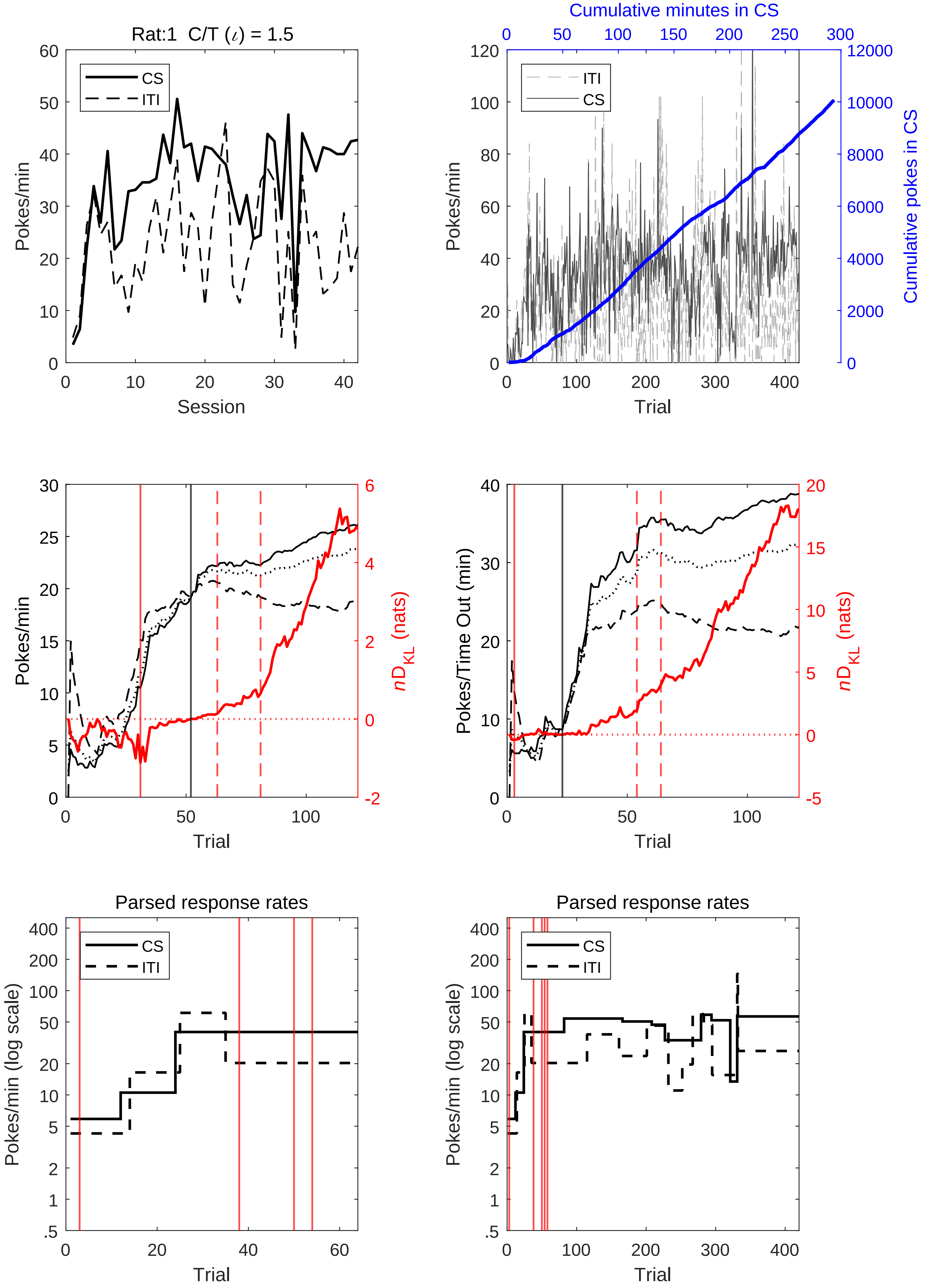

Rat:1 C/T (ι) = 1.5
CS
ITI
Pokes/min
Session
Cumulative minutes in CS
Cumulative pokes in CS
Trial
Pokes/Time Out (min)
nD_KL (nats)
Parsed response rates
Pokes/min (log scale)

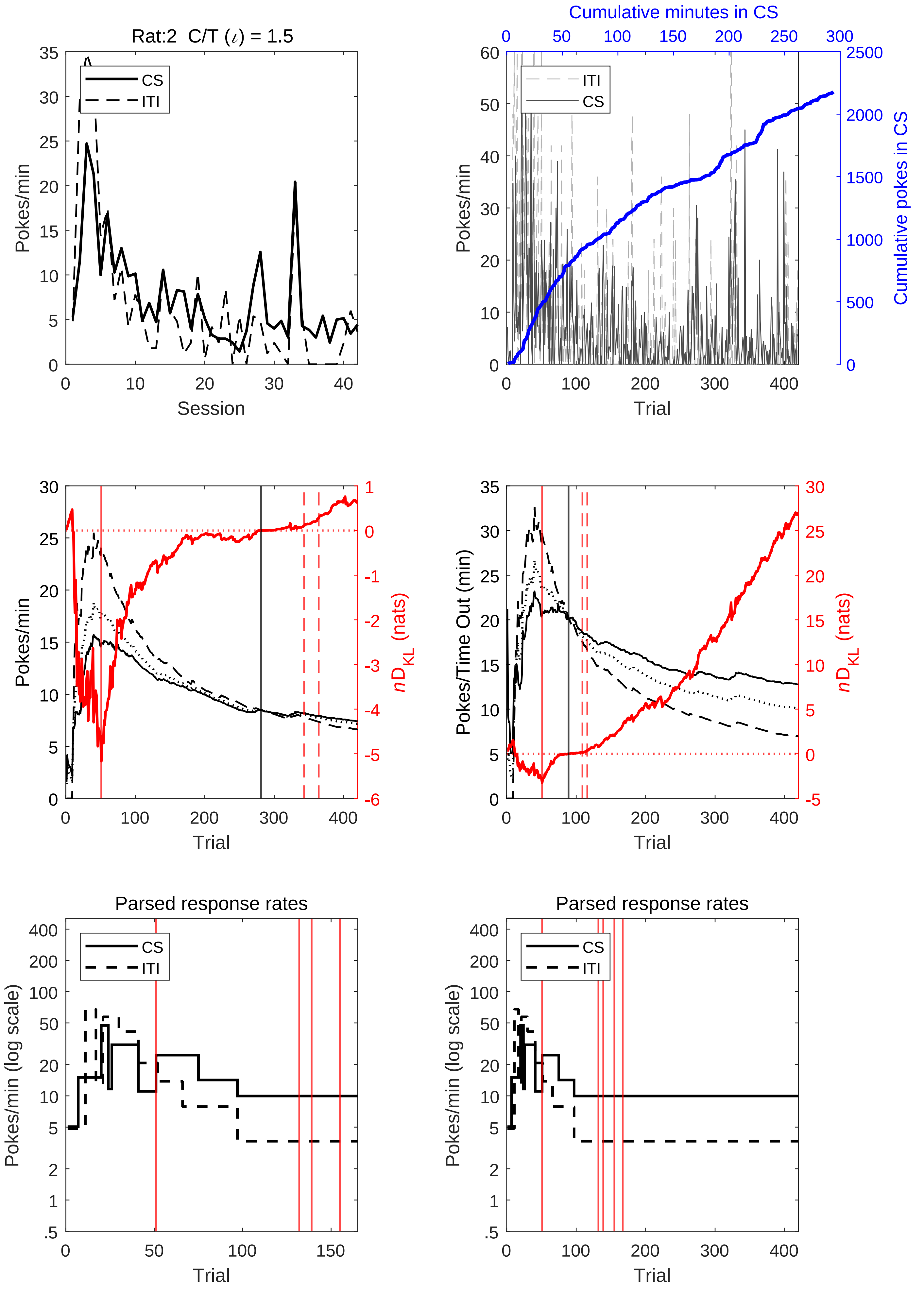

Rat:2 C/T (ι) = 1.5
CS
ITI
Pokes/min
Session
Cumulative minutes in CS
Cumulative pokes in CS
Trial
$nD_{KL}$ (nats)
Pokes/Time Out (min)
Parsed response rates
Pokes/min (log scale)

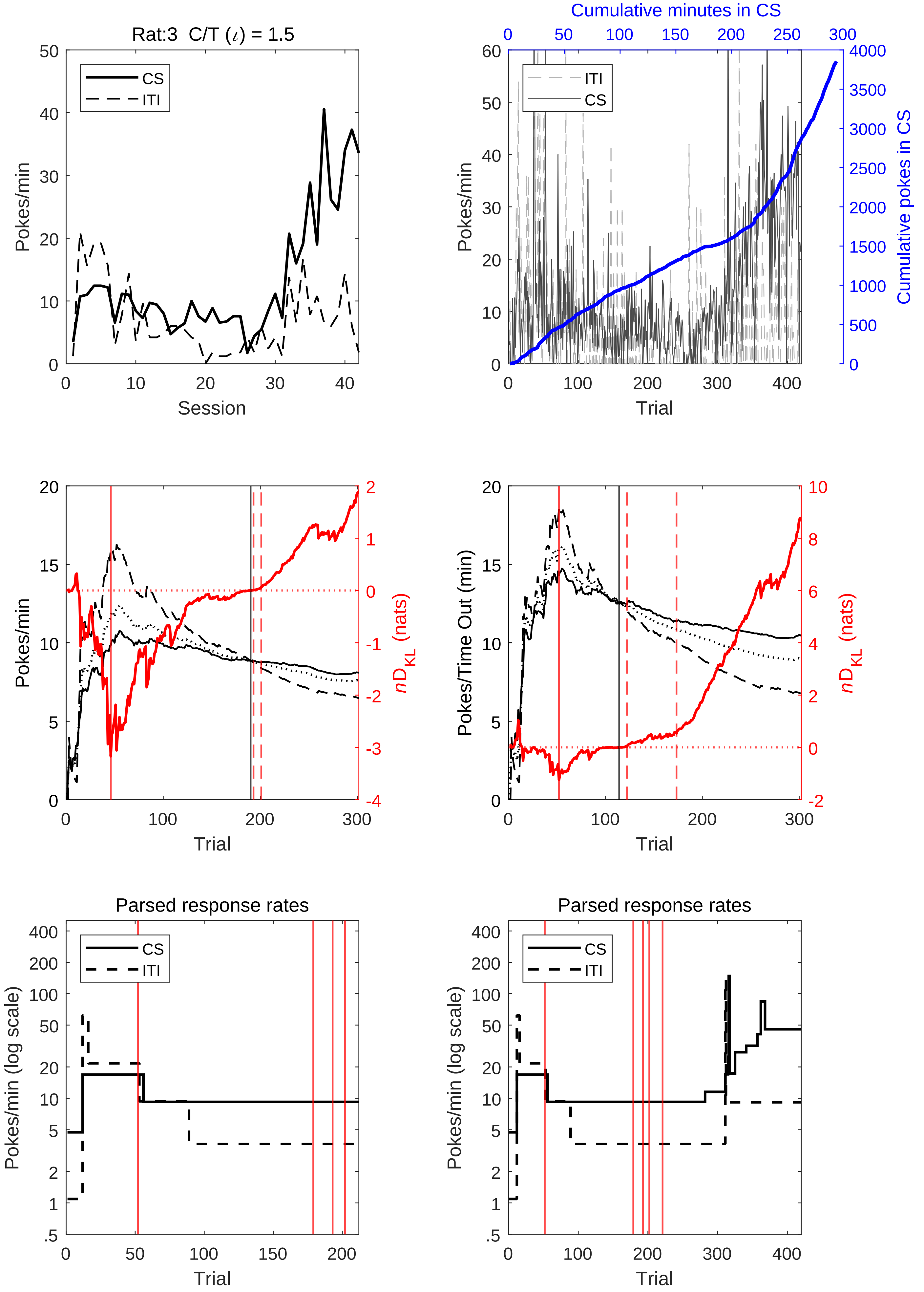

Rat:3 C/T (ι) = 1.5
Pokes/min
Session
CS
ITI
Cumulative minutes in CS
Cumulative pokes in CS
Trial
Pokes/Time Out (min)
nD_KL (nats)
Parsed response rates
Pokes/min (log scale)

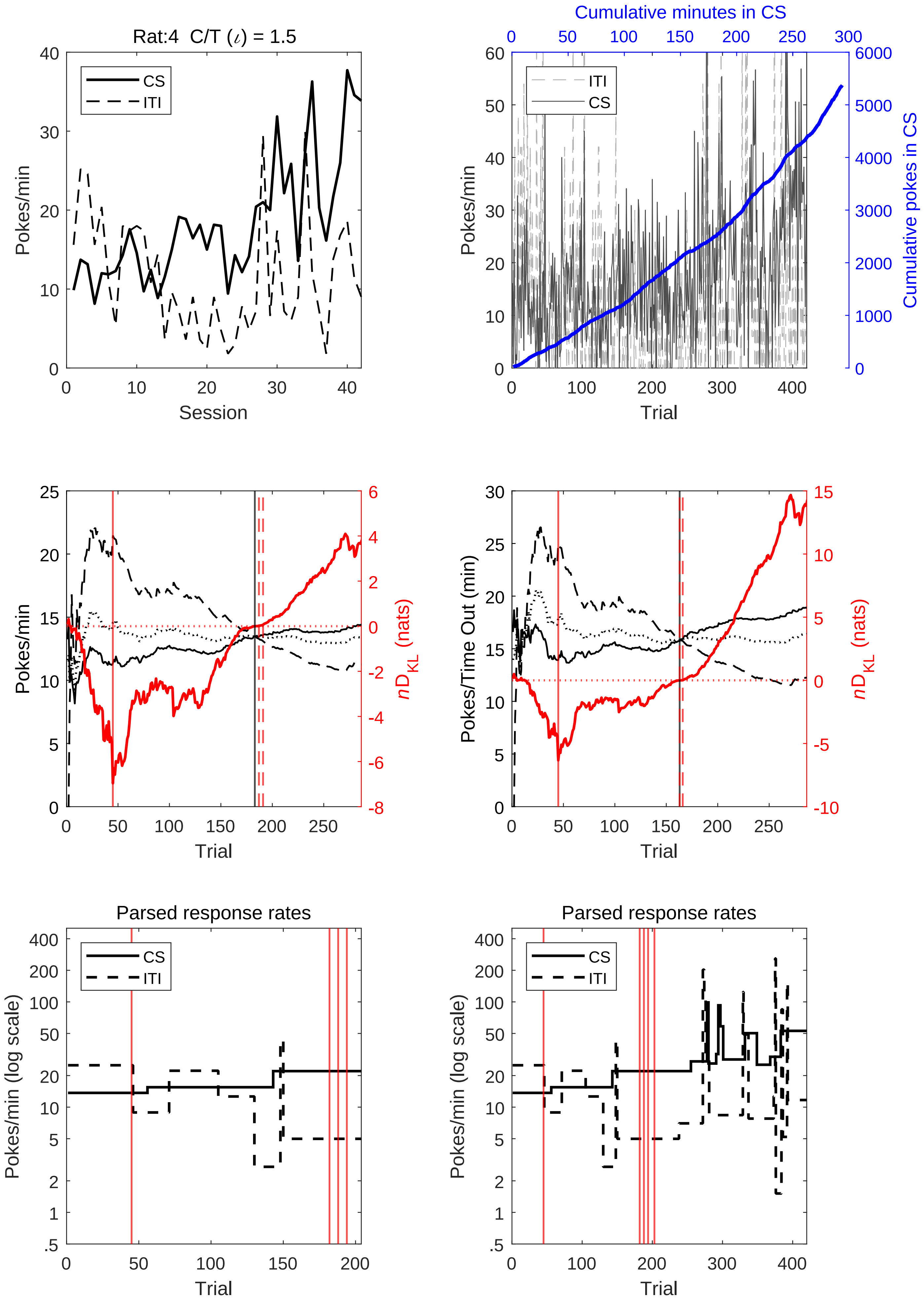
Rat:4 C/T ($\iota$) = 1.5
CS
ITI
Pokes/min
Session
Cumulative minutes in CS
ITI
CS
Pokes/min
Cumulative pokes in CS
Trial
Pokes/min
$n\mathrm{D}_{\mathrm{KL}}$ (nats)
Trial
Pokes/Time Out (min)
$n\mathrm{D}_{\mathrm{KL}}$ (nats)
Trial
Parsed response rates
CS
ITI
Pokes/min (log scale)
Trial
Parsed response rates
CS
ITI
Pokes/min (log scale)
Trial

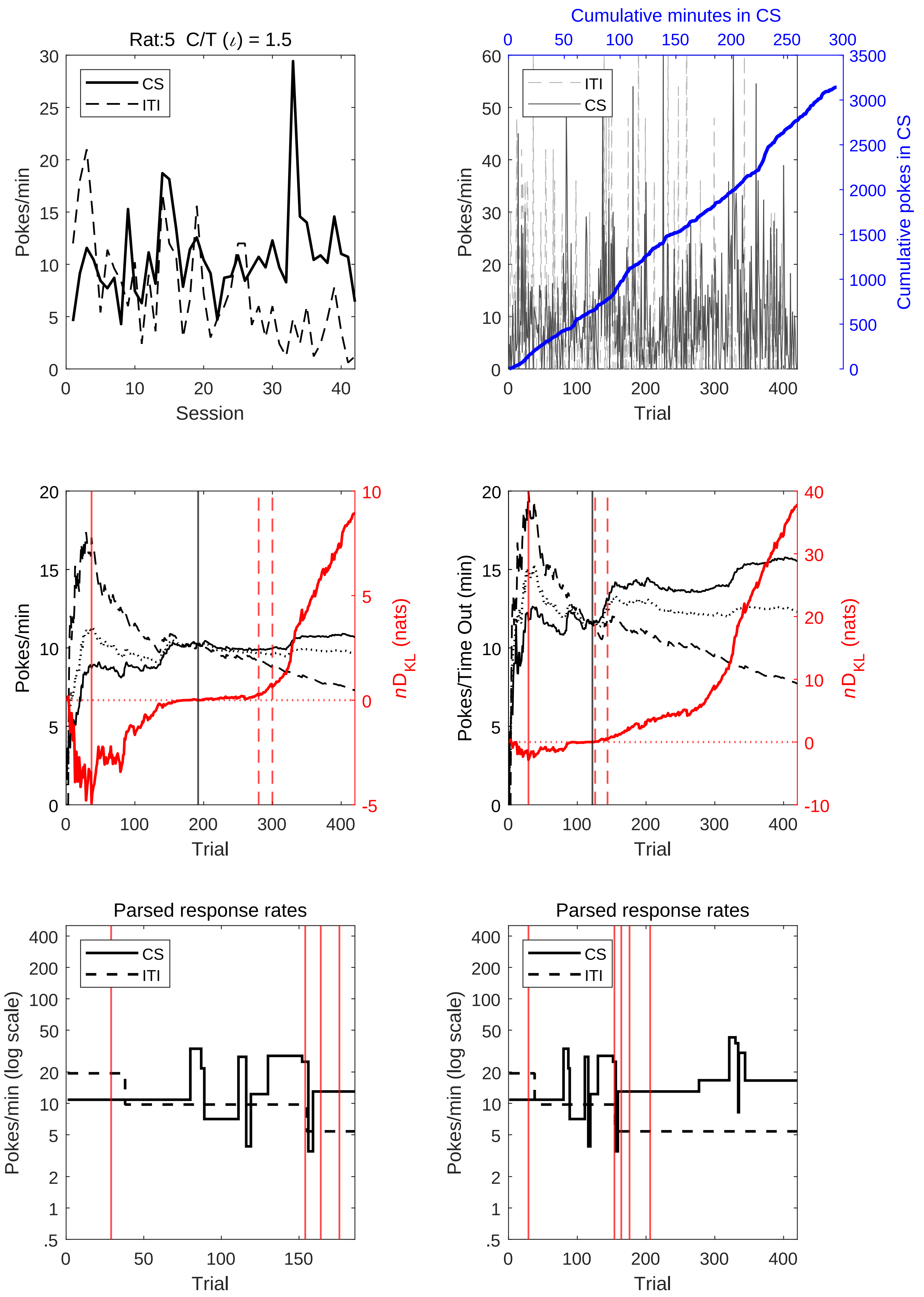

Rat:5 C/T (ι) = 1.5
CS
ITI
Pokes/min
Session
Cumulative minutes in CS
Cumulative pokes in CS
Trial
Pokes/min
nD_KL (nats)
Pokes/Time Out (min)
Parsed response rates
Pokes/min (log scale)

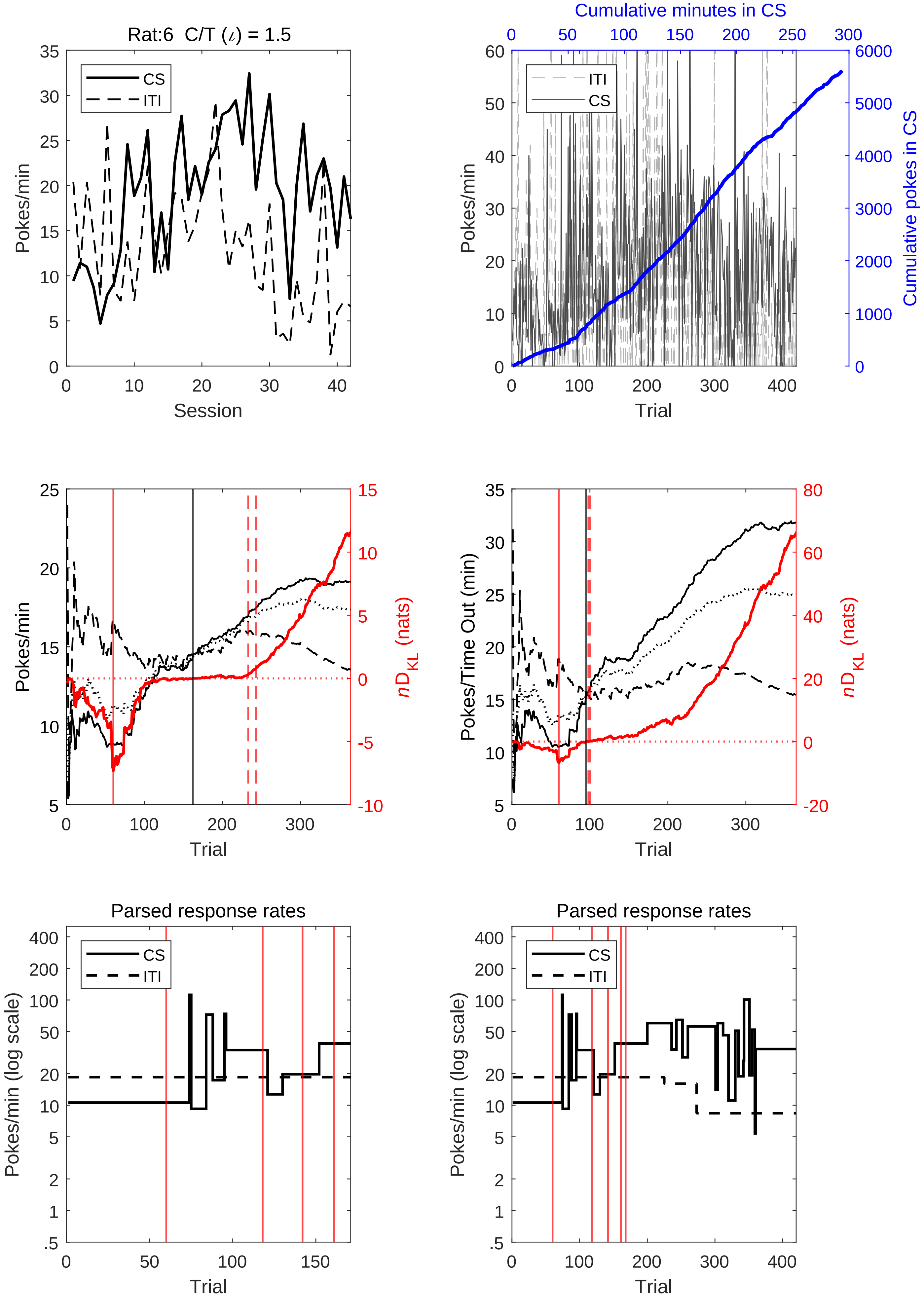

Rat:6 C/T (ι) = 1.5
CS
ITI
Pokes/min
Session
Cumulative minutes in CS
ITI
CS
Pokes/min
Cumulative pokes in CS
Trial
Pokes/min
nD_KL (nats)
Trial
Pokes/Time Out (min)
nD_KL (nats)
Trial
Parsed response rates
CS
ITI
Pokes/min (log scale)
Trial
Parsed response rates
CS
ITI
Pokes/min (log scale)
Trial

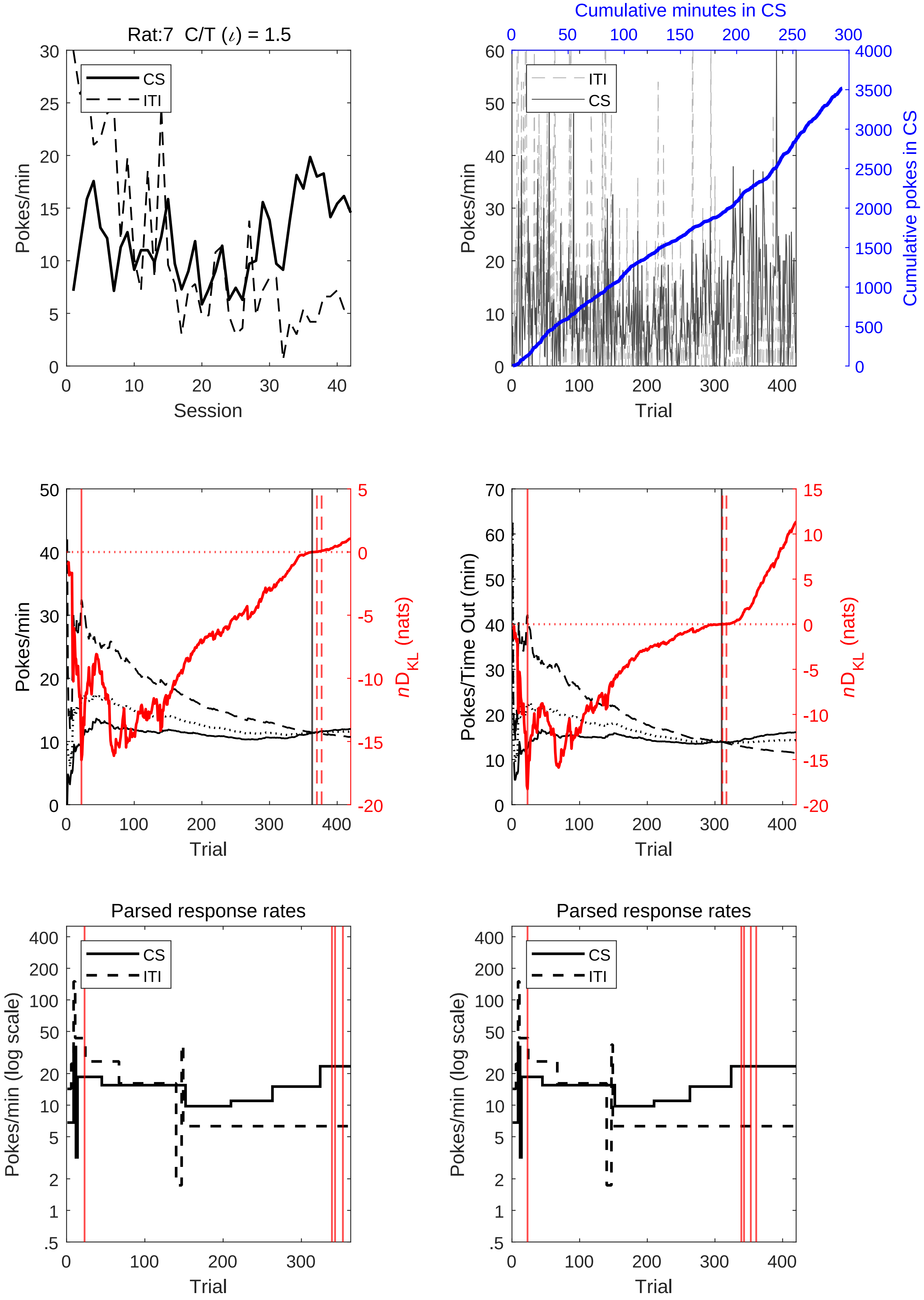
Rat:7 C/T (ι) = 1.5
CS
ITI
Pokes/min
Session
Cumulative minutes in CS
Cumulative pokes in CS
Trial
Pokes/Time Out (min)
$nD_{KL}$ (nats)
Parsed response rates
Pokes/min (log scale)

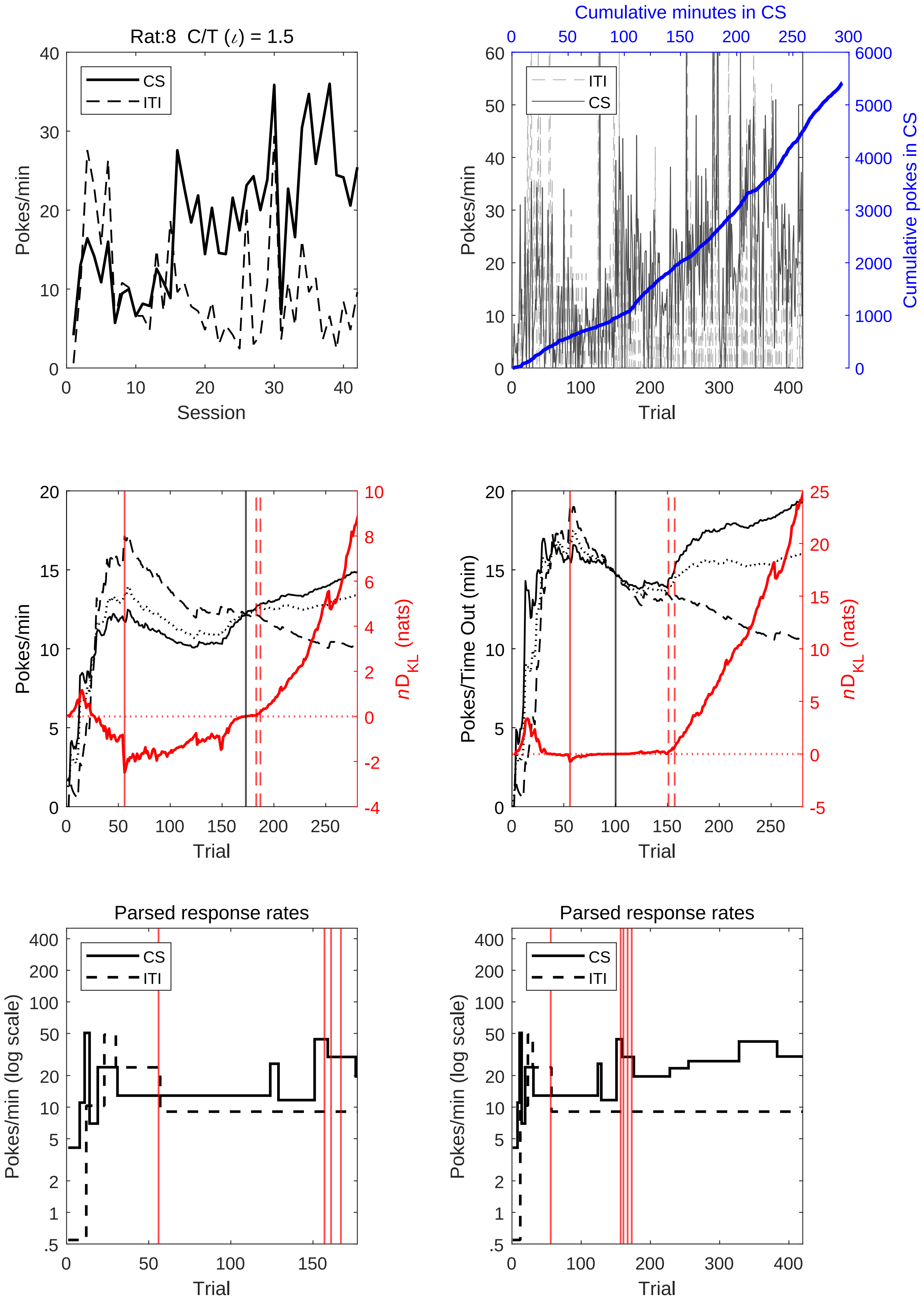

Rat:8 C/T (ι) = 1.5
CS
ITI
Pokes/min
Session
Cumulative minutes in CS
Cumulative pokes in CS
Trial
nD_KL (nats)
Pokes/Time Out (min)
Parsed response rates
Pokes/min (log scale)

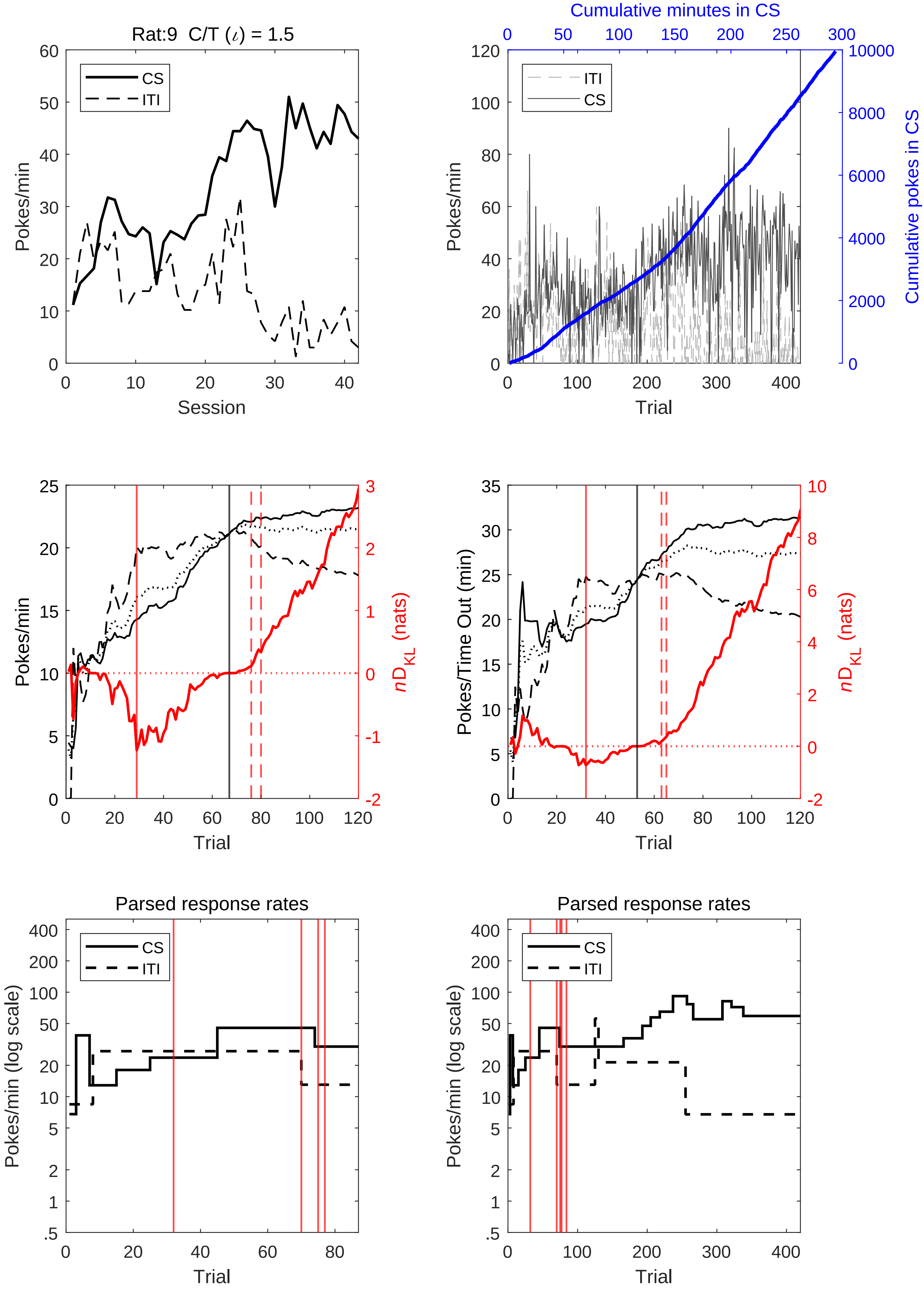
Rat:9 C/T (ι) = 1.5
Pokes/min
Session
CS
ITI
Cumulative minutes in CS
Cumulative pokes in CS
Trial
Pokes/Time Out (min)
nD_KL (nats)
Parsed response rates
Pokes/min (log scale)

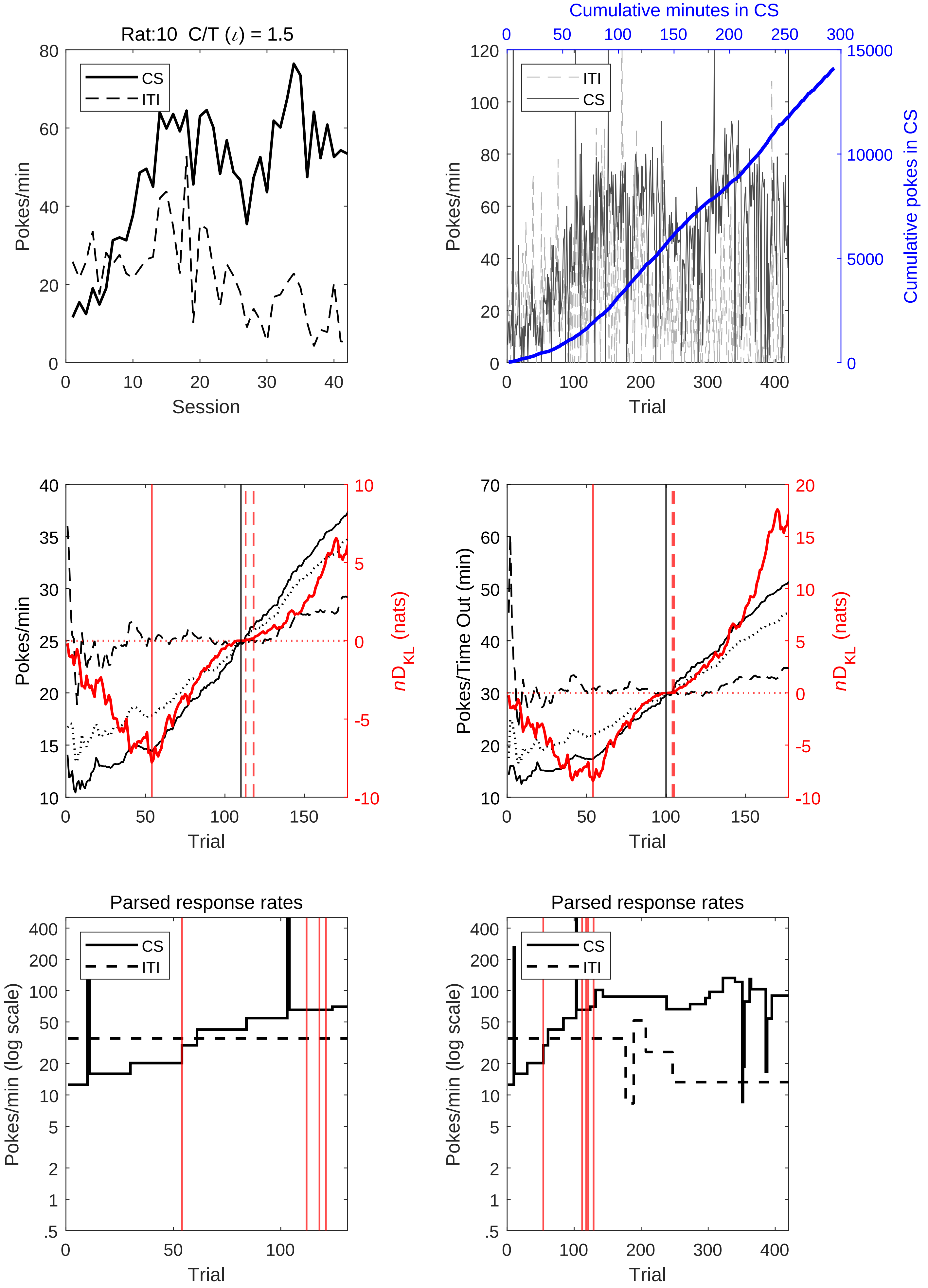
Rat:10 C/T (ι) = 1.5
CS
ITI
Pokes/min
Session
Cumulative minutes in CS
ITI
CS
Pokes/min
Cumulative pokes in CS
Trial
Pokes/min
$nD_{KL}$ (nats)
Trial
Pokes/Time Out (min)
$nD_{KL}$ (nats)
Trial
Parsed response rates
CS
ITI
Pokes/min (log scale)
Trial
Parsed response rates
CS
ITI
Pokes/min (log scale)
Trial

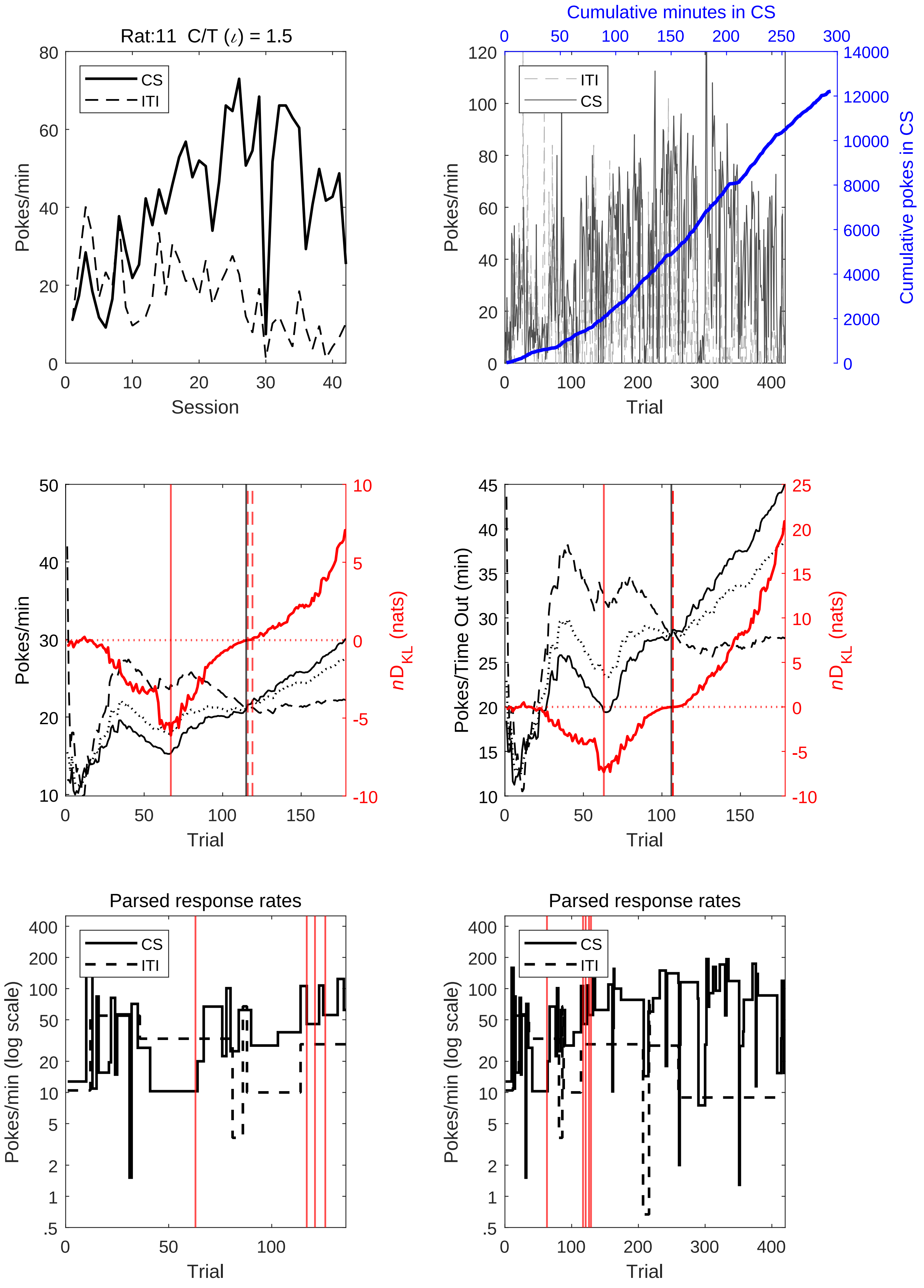
Rat:11 C/T ($\iota$) = 1.5
CS
ITI
Pokes/min
Session
Cumulative minutes in CS
Cumulative pokes in CS
Trial
Pokes/Time Out (min)
$nD_{KL}$ (nats)
Parsed response rates
Pokes/min (log scale)

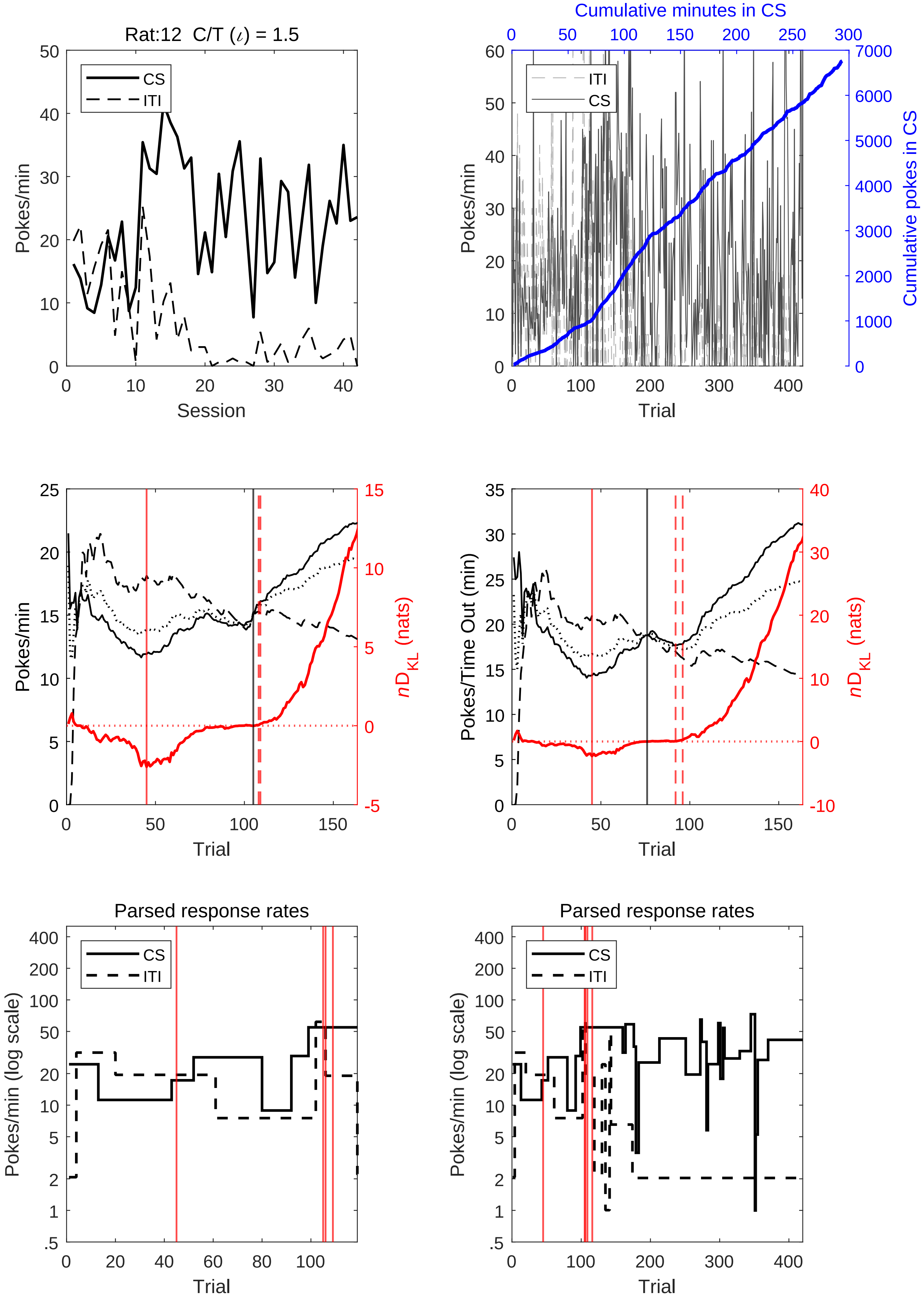
Rat:12 C/T (ι) = 1.5
CS
ITI
Pokes/min
Session
Cumulative minutes in CS
Cumulative pokes in CS
Trial
nD_KL (nats)
Pokes/Time Out (min)
Parsed response rates
Pokes/min (log scale)

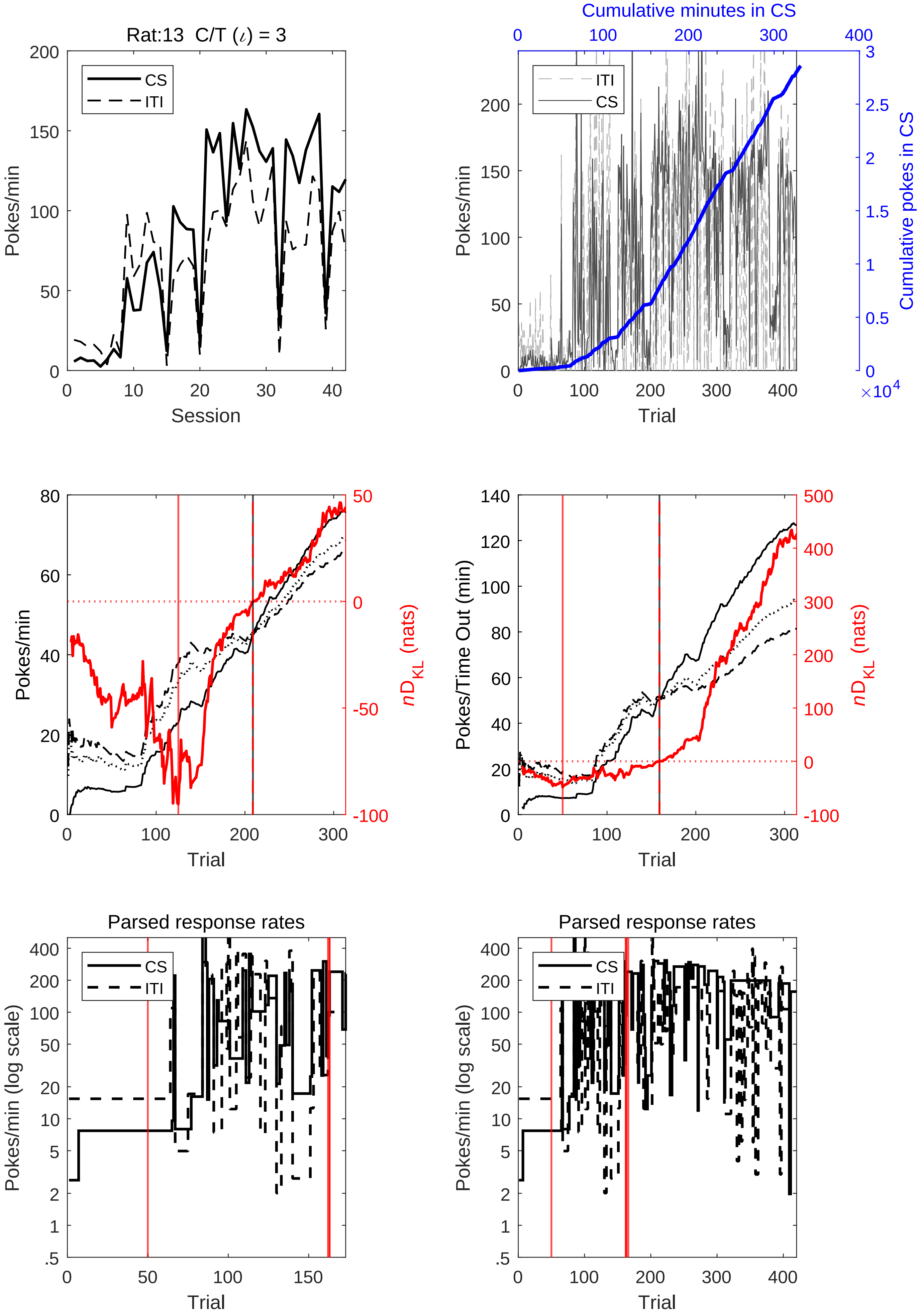

Rat:13 C/T (ι) = 3
Pokes/min
Session
CS
ITI
Cumulative minutes in CS
Cumulative pokes in CS
×10^4
Trial
Pokes/min
nD_KL (nats)
Pokes/Time Out (min)
Parsed response rates
Pokes/min (log scale)

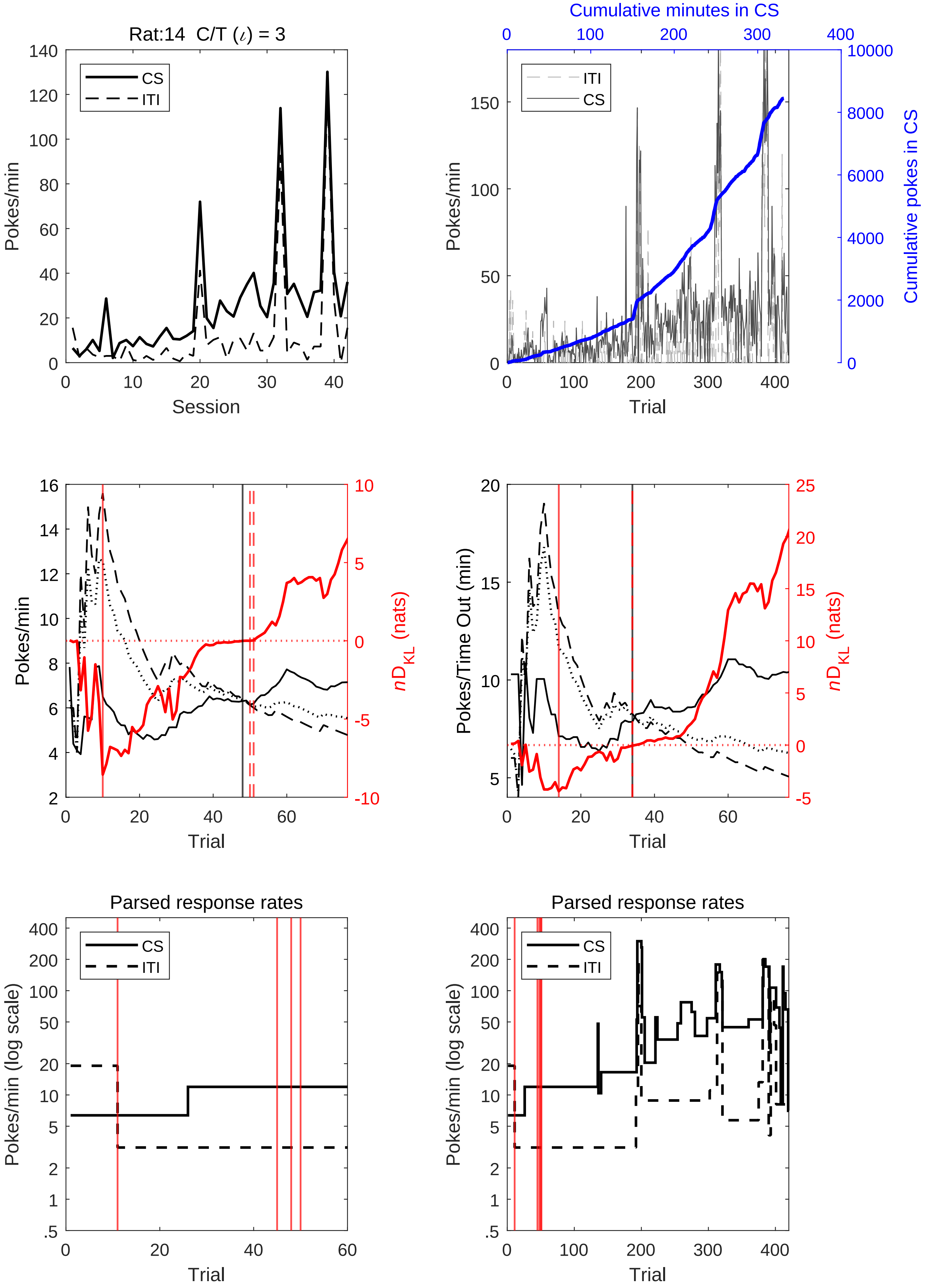
Rat:14 C/T ($\iota$) = 3
CS
ITI
Pokes/min
Session
Cumulative minutes in CS
Cumulative pokes in CS
Trial
$n$D$_{KL}$ (nats)
Pokes/Time Out (min)
Parsed response rates
Pokes/min (log scale)

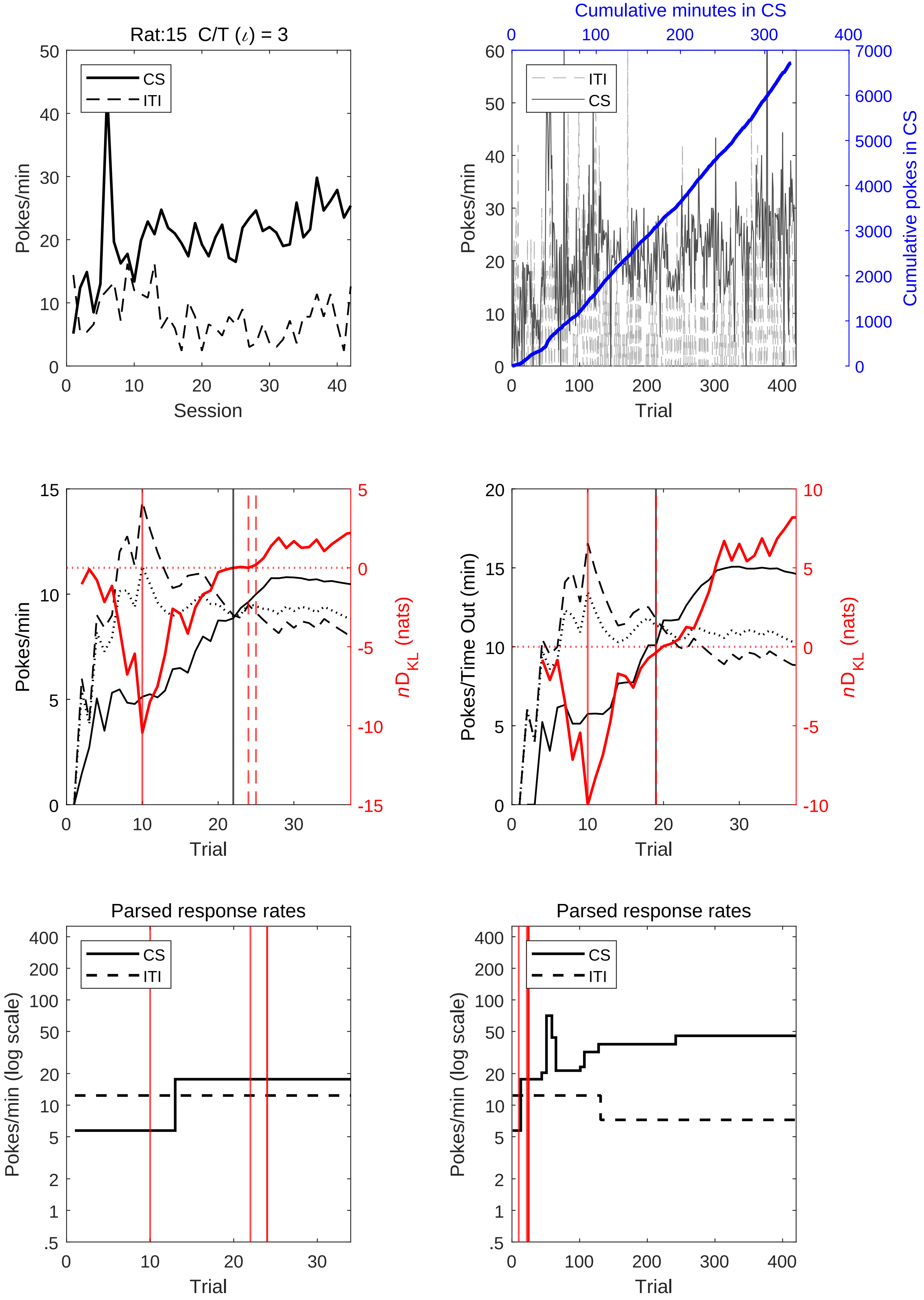
Rat:15 C/T (ι) = 3
CS
ITI
Pokes/min
Session
Cumulative minutes in CS
Cumulative pokes in CS
Trial
Pokes/Time Out (min)
nD_KL (nats)
Parsed response rates
Pokes/min (log scale)

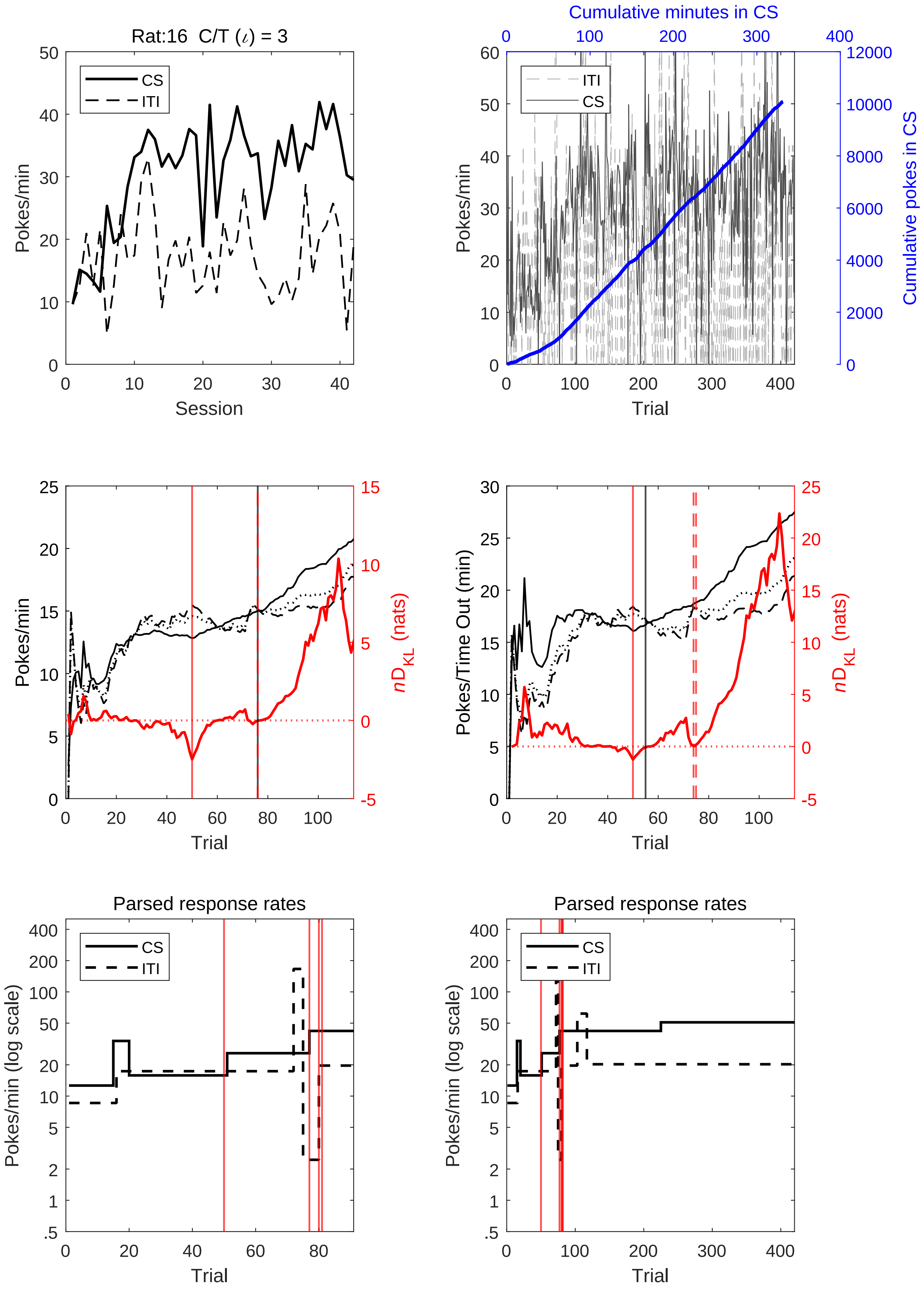

Rat:16 C/T (ι) = 3
CS
ITI
Pokes/min
Session
Cumulative minutes in CS
Cumulative pokes in CS
Trial
Pokes/min
nD_KL (nats)
Pokes/Time Out (min)
Parsed response rates
Pokes/min (log scale)

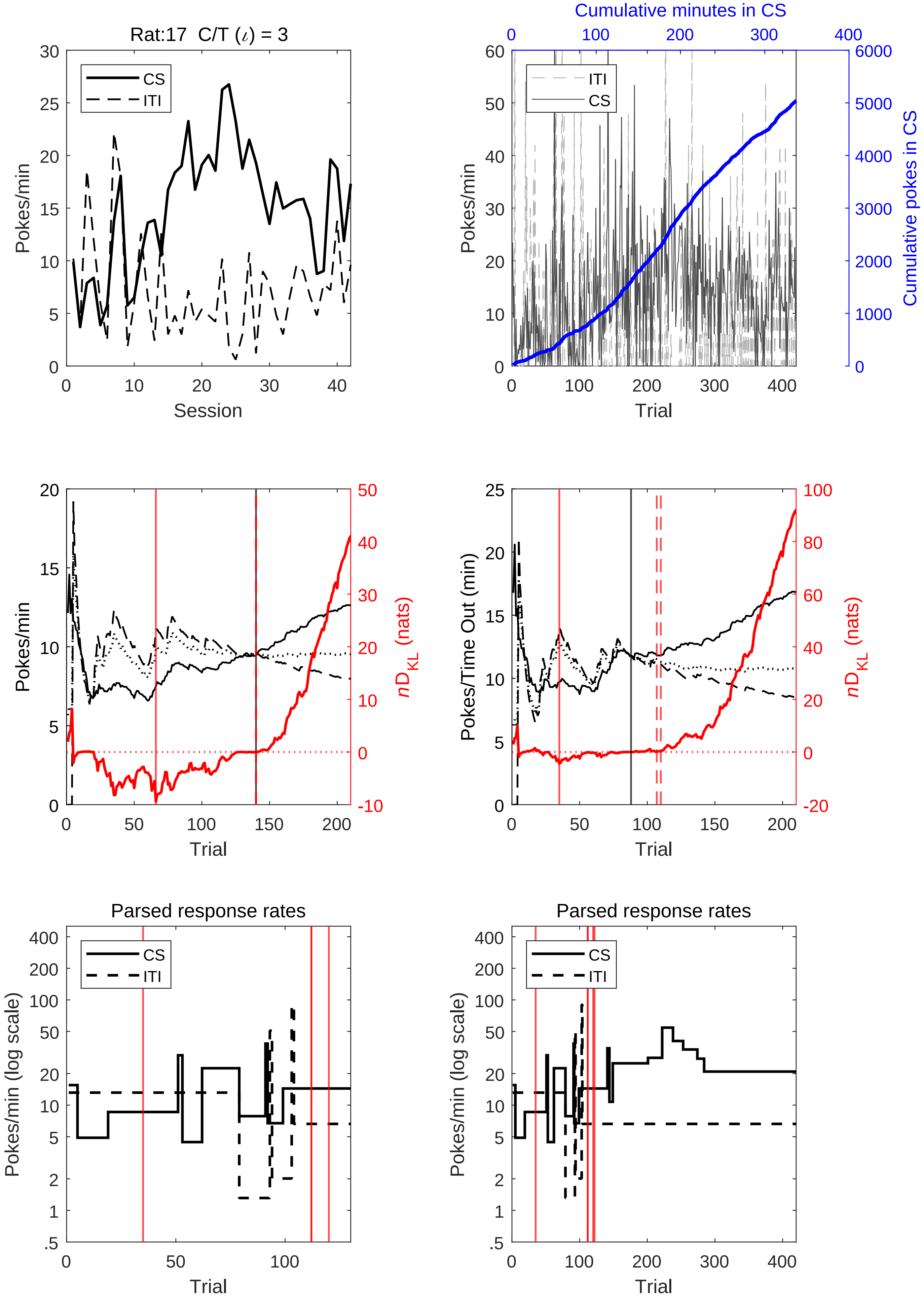

Rat:17 C/T (ι) = 3
CS
ITI
Pokes/min
Session
Cumulative minutes in CS
Cumulative pokes in CS
Trial
nD_KL (nats)
Pokes/Time Out (min)
Parsed response rates
Pokes/min (log scale)

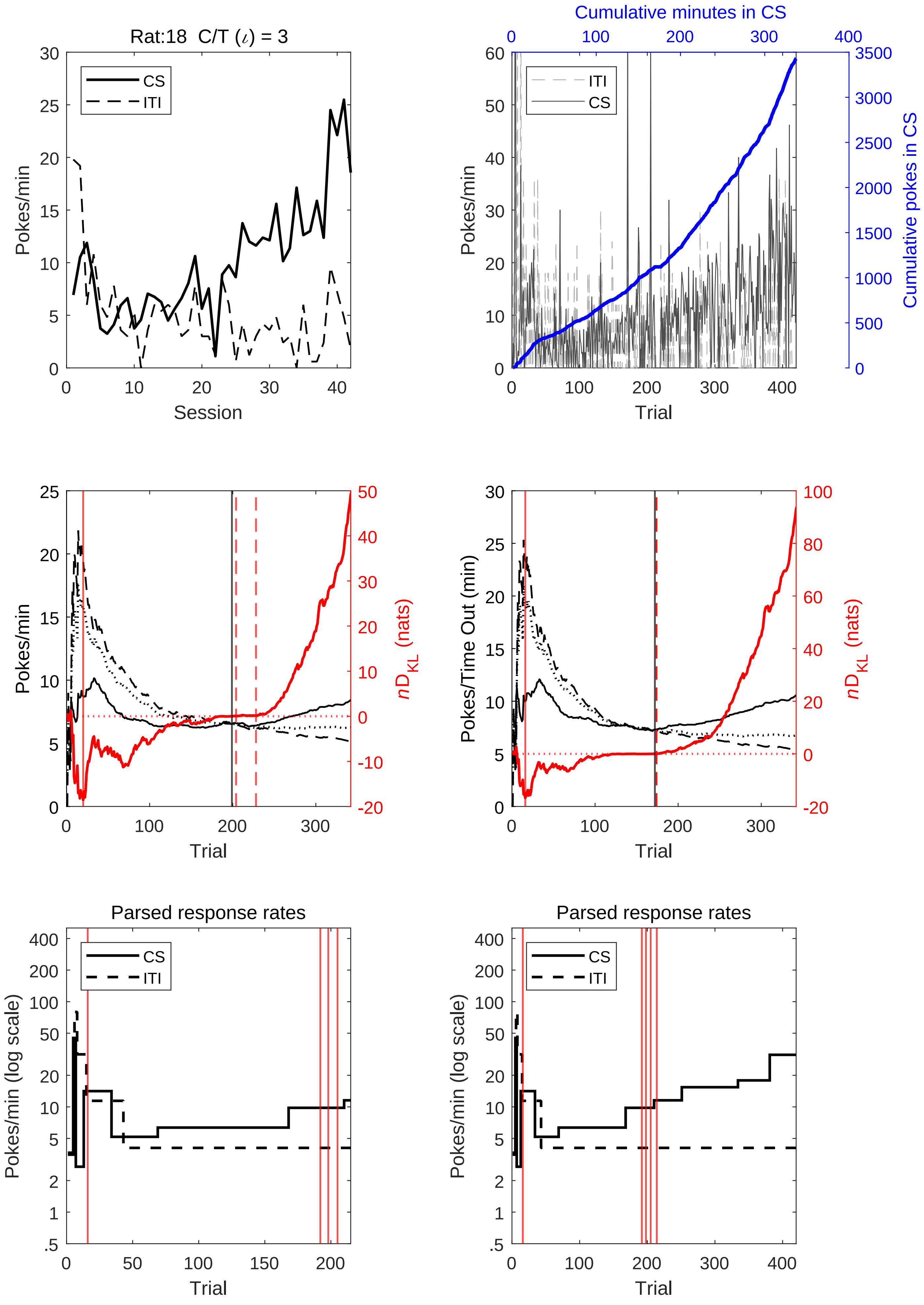

Rat:18 C/T (ι) = 3
CS
ITI
Pokes/min
Session
Cumulative minutes in CS
Cumulative pokes in CS
Trial
nD_KL (nats)
Pokes/Time Out (min)
Parsed response rates
Pokes/min (log scale)

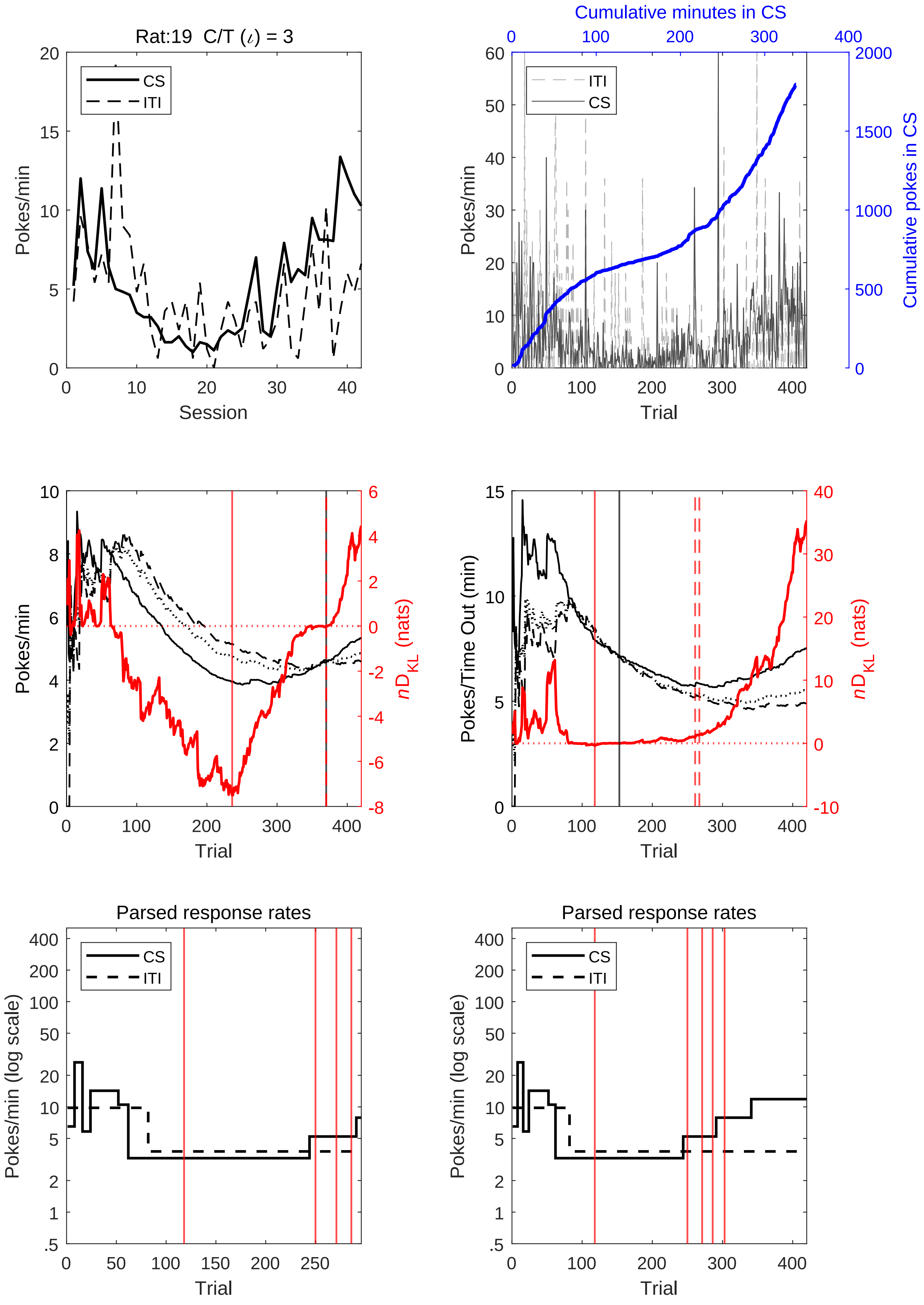

Rat:19  C/T (ι) = 3
CS
ITI
Pokes/min
Session
Cumulative minutes in CS
Cumulative pokes in CS
Trial
nD_KL (nats)
Pokes/Time Out (min)
Parsed response rates
Pokes/min (log scale)

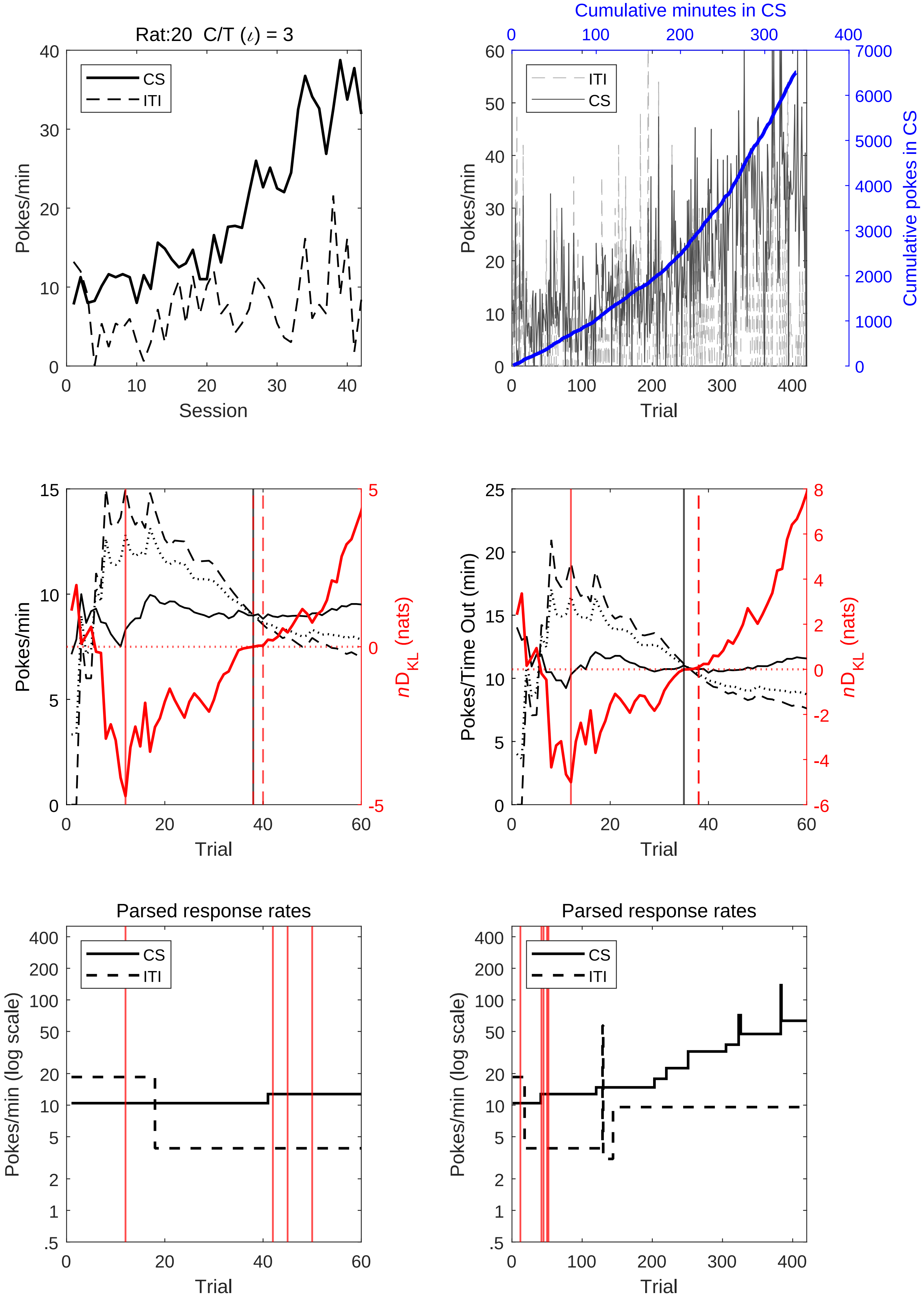

Rat:20 C/T (ι) = 3
CS
ITI
Pokes/min
Session
Cumulative minutes in CS
Cumulative pokes in CS
Trial
nD_KL (nats)
Pokes/Time Out (min)
Parsed response rates
Pokes/min (log scale)

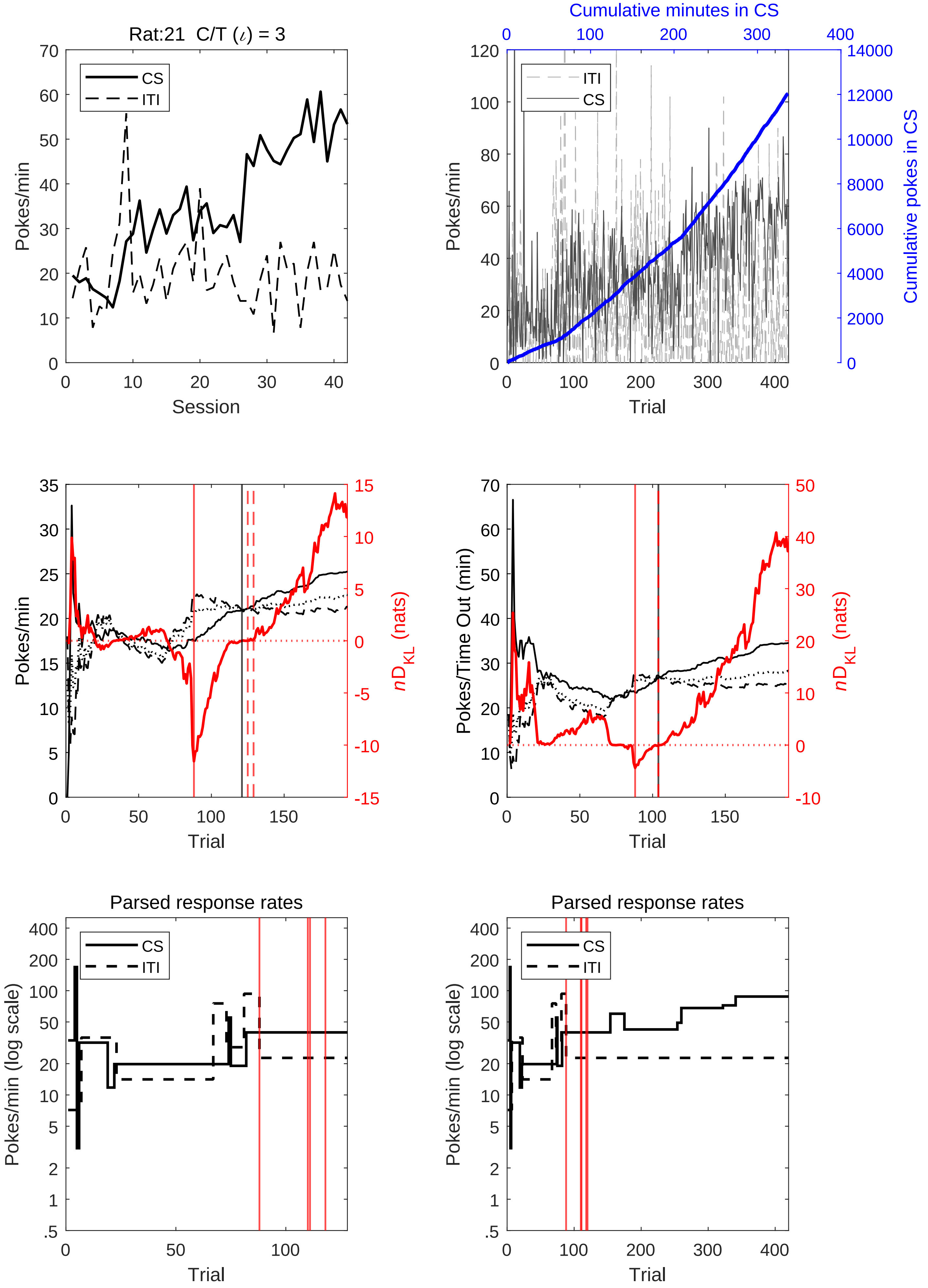
Rat:21 C/T (ι) = 3
CS
ITI
Pokes/min
Session
Cumulative minutes in CS
Cumulative pokes in CS
Trial
nD_KL (nats)
Pokes/Time Out (min)
Parsed response rates
Pokes/min (log scale)

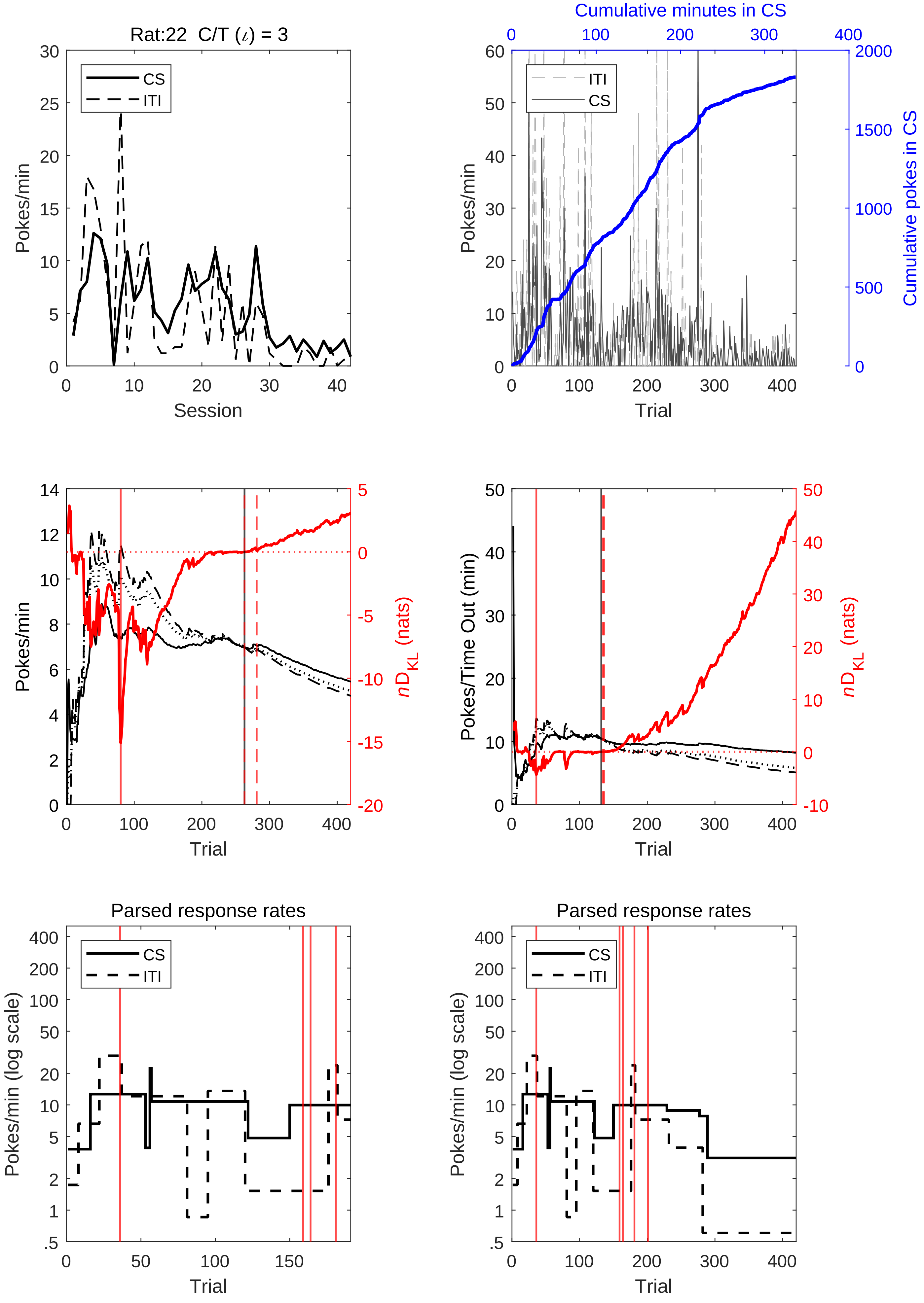
Rat:22 C/T (ι) = 3
CS
ITI
Pokes/min
Session
Cumulative minutes in CS
Cumulative pokes in CS
Trial
nD_KL (nats)
Pokes/Time Out (min)
Parsed response rates
Pokes/min (log scale)

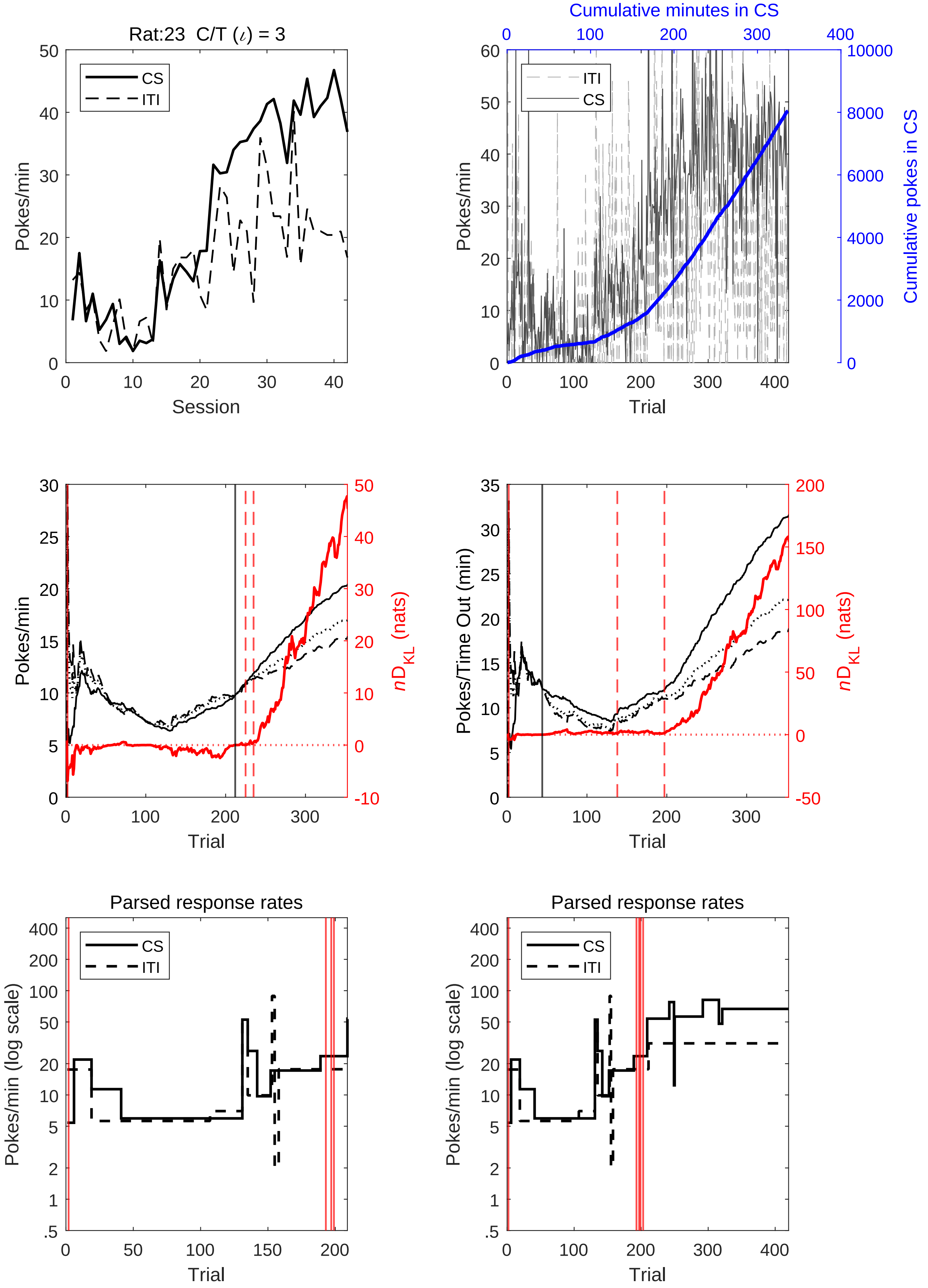
Rat:23 C/T (ι) = 3
CS
ITI
Pokes/min
Session
Cumulative minutes in CS
ITI
CS
Pokes/min
Trial
Cumulative pokes in CS
Pokes/min
Trial
nD_KL (nats)
Pokes/Time Out (min)
Trial
nD_KL (nats)
Parsed response rates
CS
ITI
Pokes/min (log scale)
Trial
Parsed response rates
CS
ITI
Pokes/min (log scale)
Trial

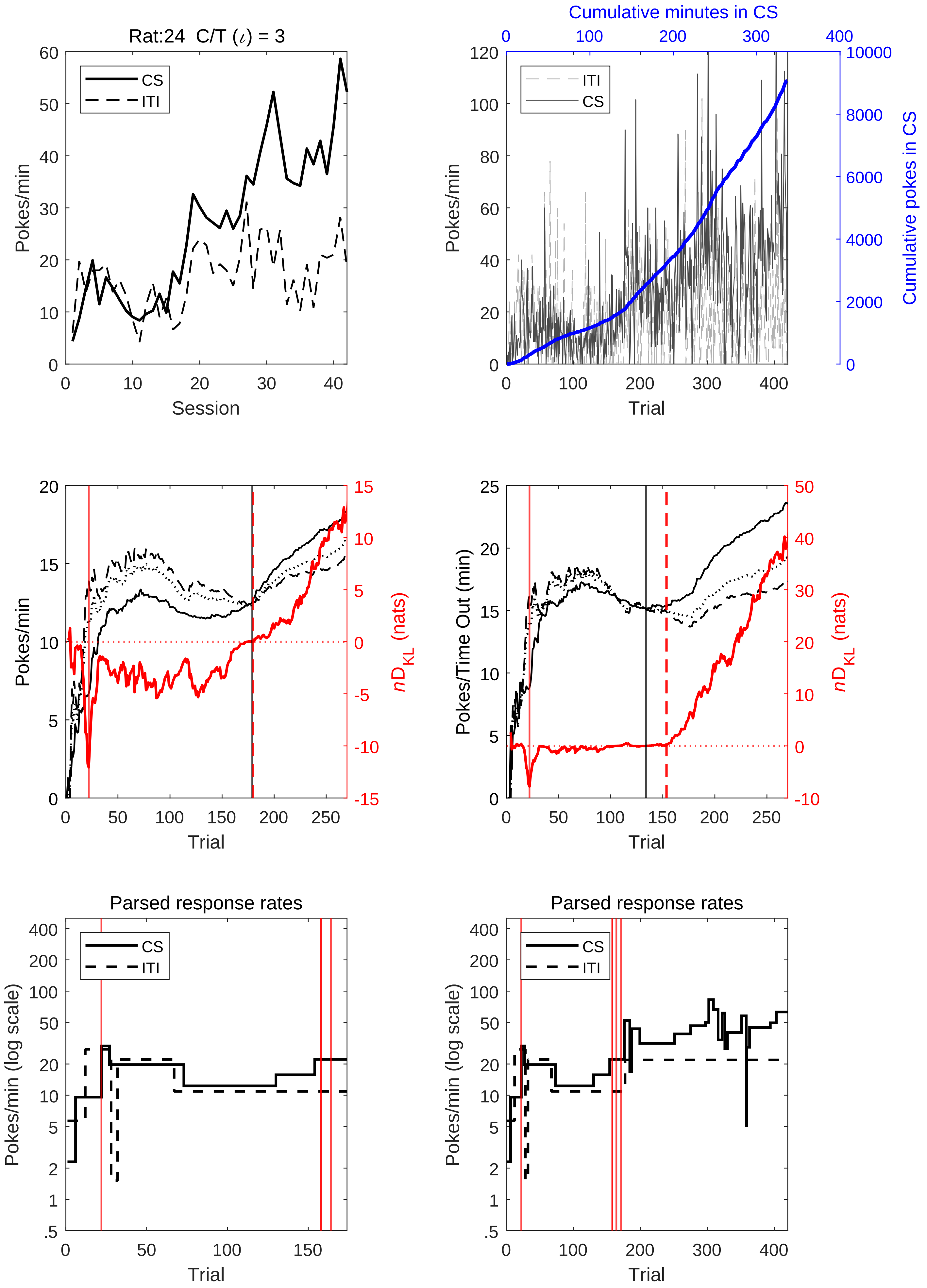
Rat:24 C/T (ι) = 3
CS
ITI
Pokes/min
Session
Cumulative minutes in CS
Cumulative pokes in CS
Trial
Pokes/Time Out (min)
nD_KL (nats)
Parsed response rates
Pokes/min (log scale)

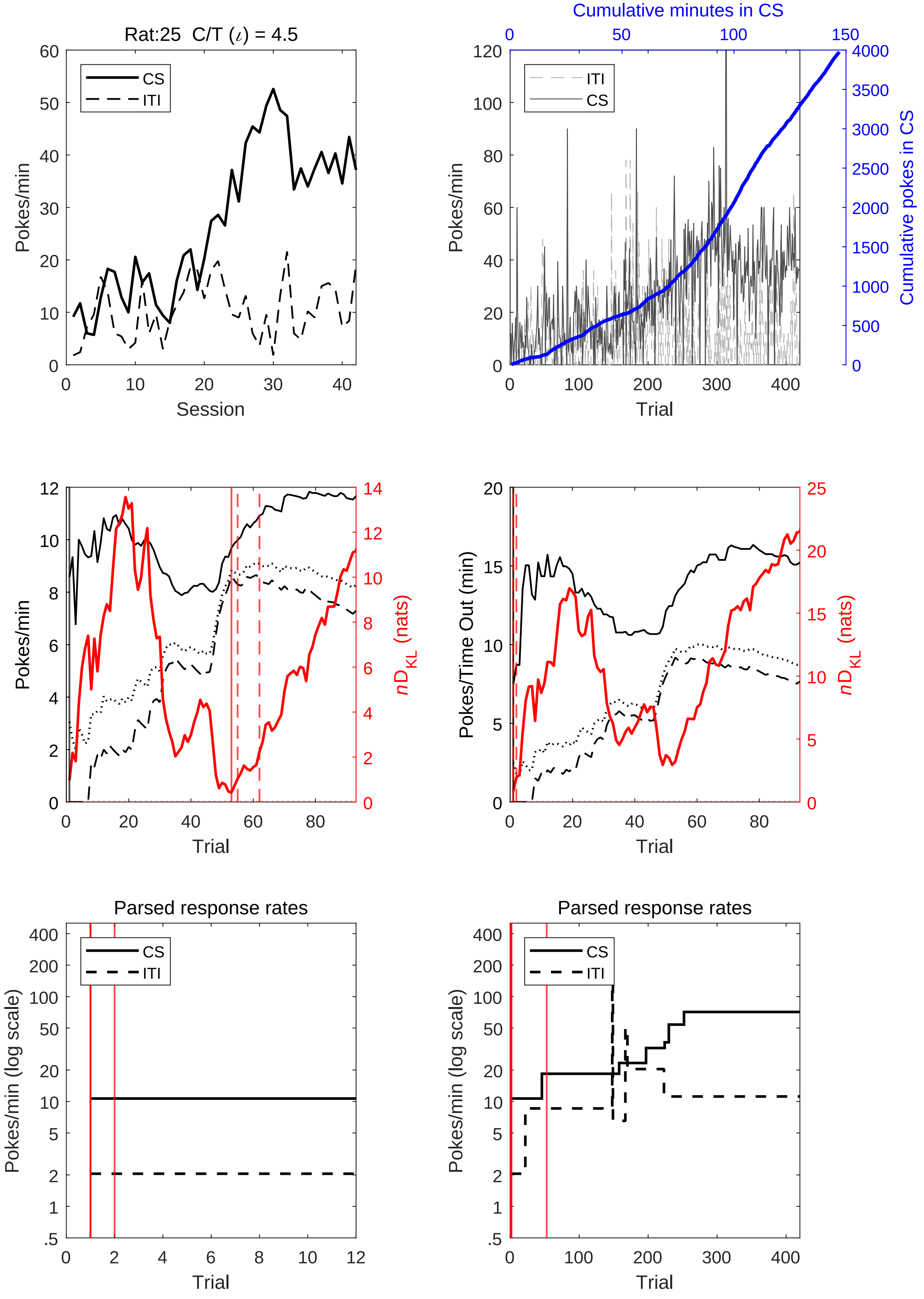
Rat:25 C/T ($\iota$) = 4.5
Pokes/min
Session
CS
ITI
Cumulative minutes in CS
Cumulative pokes in CS
Trial
Pokes/min
$nD_{KL}$ (nats)
Pokes/Time Out (min)
Parsed response rates
Pokes/min (log scale)
Trial

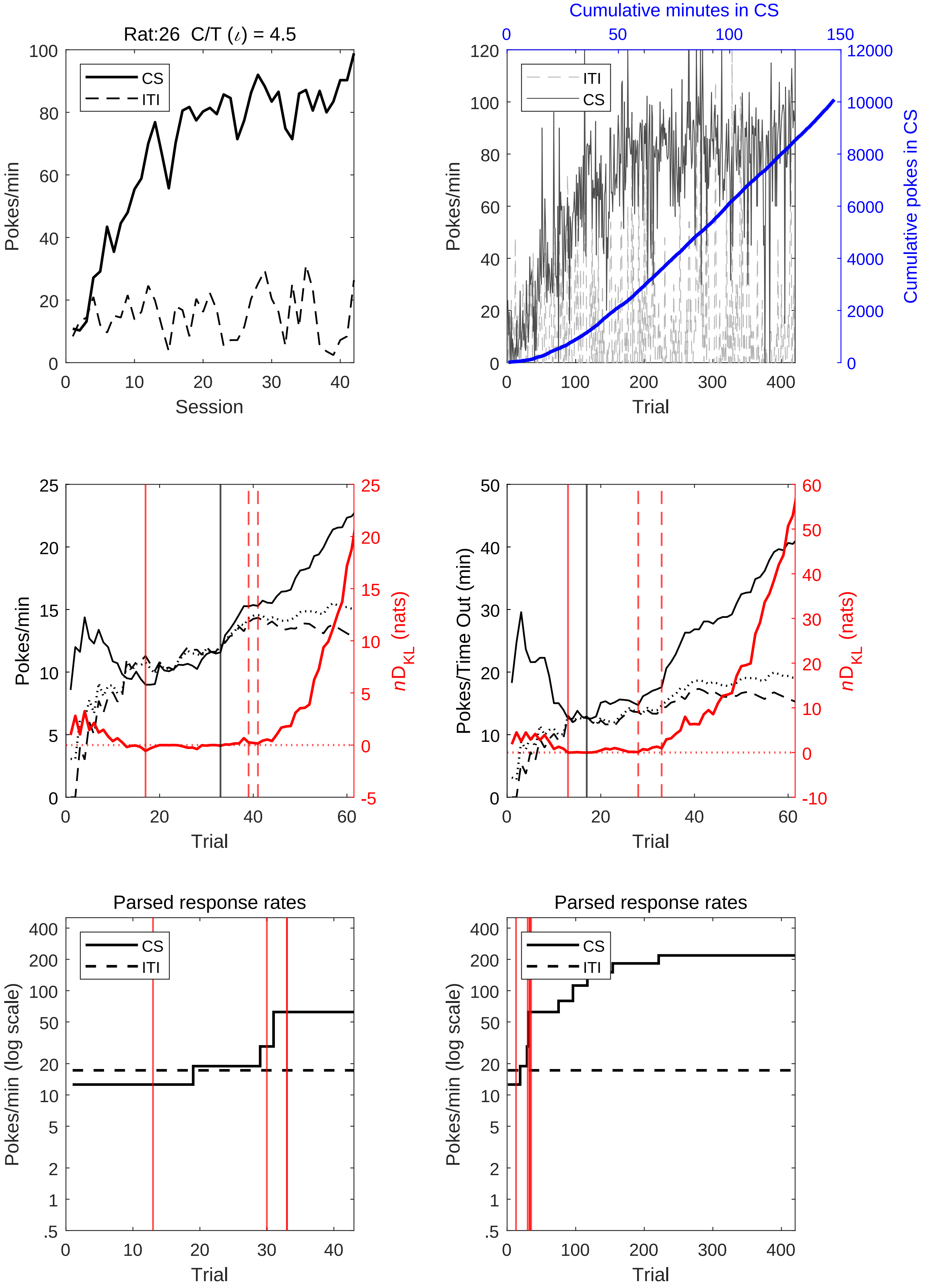
Rat:26 C/T (ι) = 4.5
CS
ITI
Pokes/min
Session
Cumulative minutes in CS
Cumulative pokes in CS
Trial
Pokes/Time Out (min)
$nD_{KL}$ (nats)
Parsed response rates
Pokes/min (log scale)

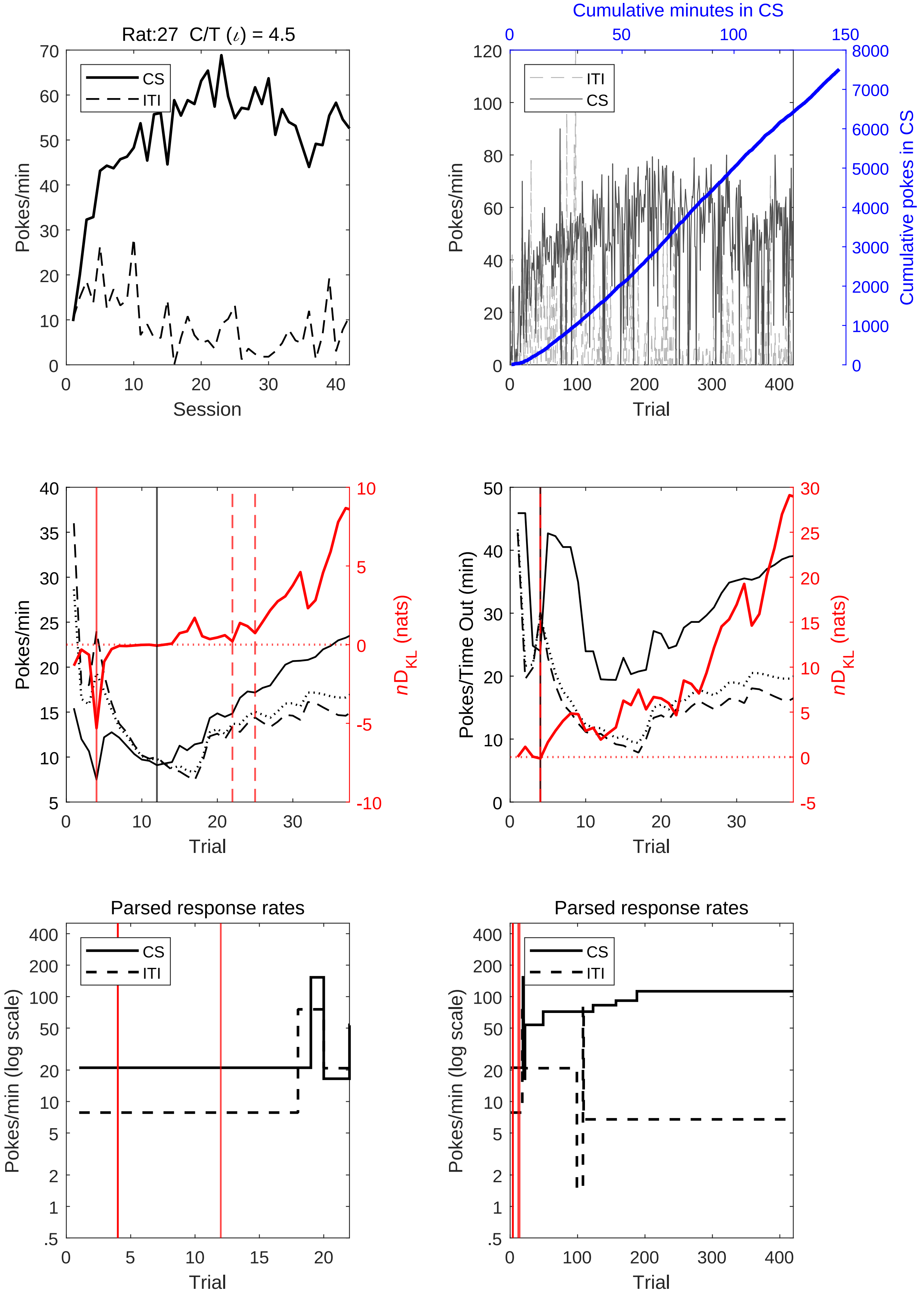
Rat:27 C/T (ι) = 4.5
CS
ITI
Pokes/min
Session
Cumulative minutes in CS
Cumulative pokes in CS
Trial
nD_KL (nats)
Pokes/Time Out (min)
Parsed response rates
Pokes/min (log scale)

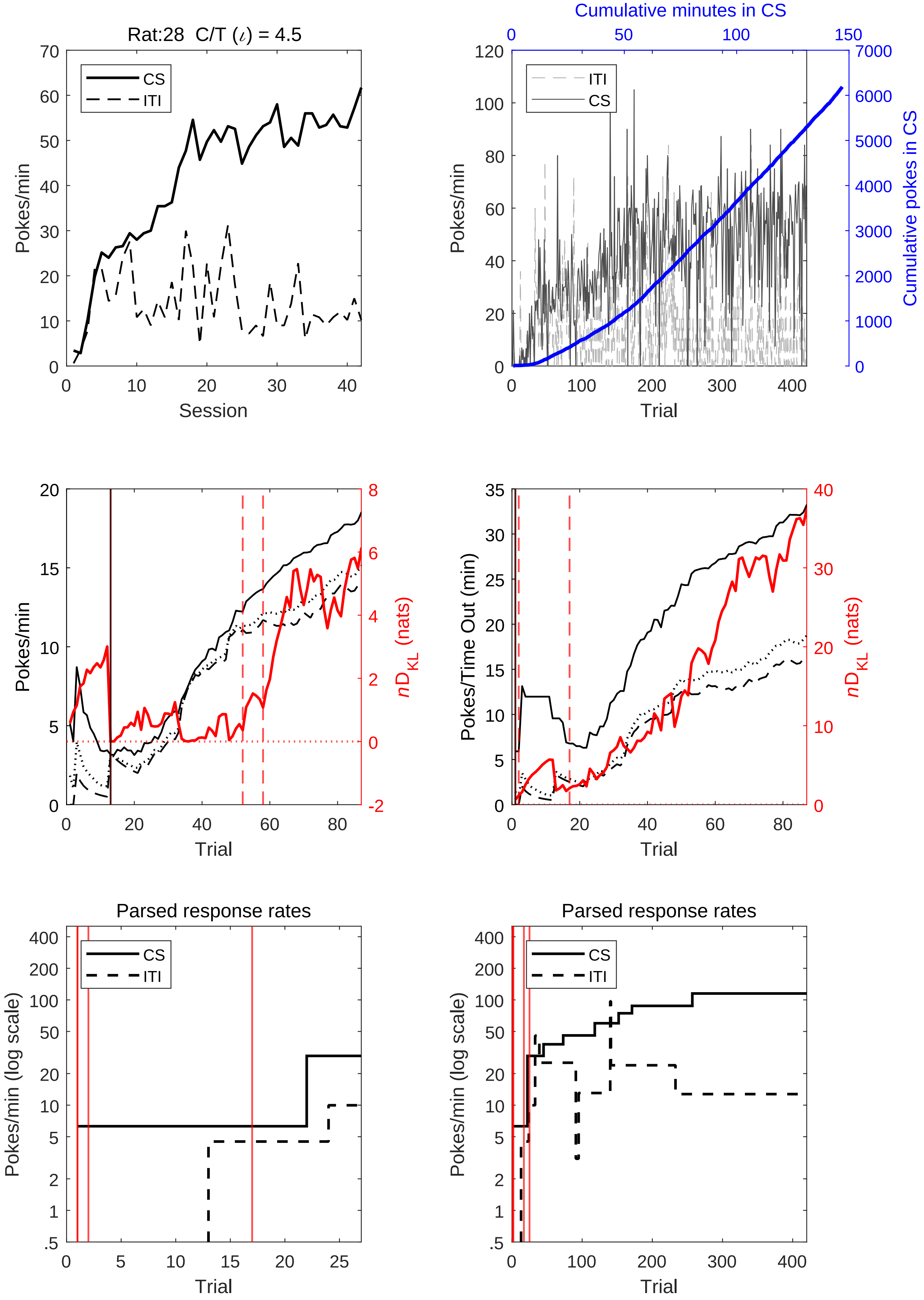
Rat:28 C/T (ι) = 4.5
CS
ITI
Pokes/min
Session
Cumulative minutes in CS
Cumulative pokes in CS
Trial
$nD_{KL}$ (nats)
Pokes/Time Out (min)
Parsed response rates
Pokes/min (log scale)

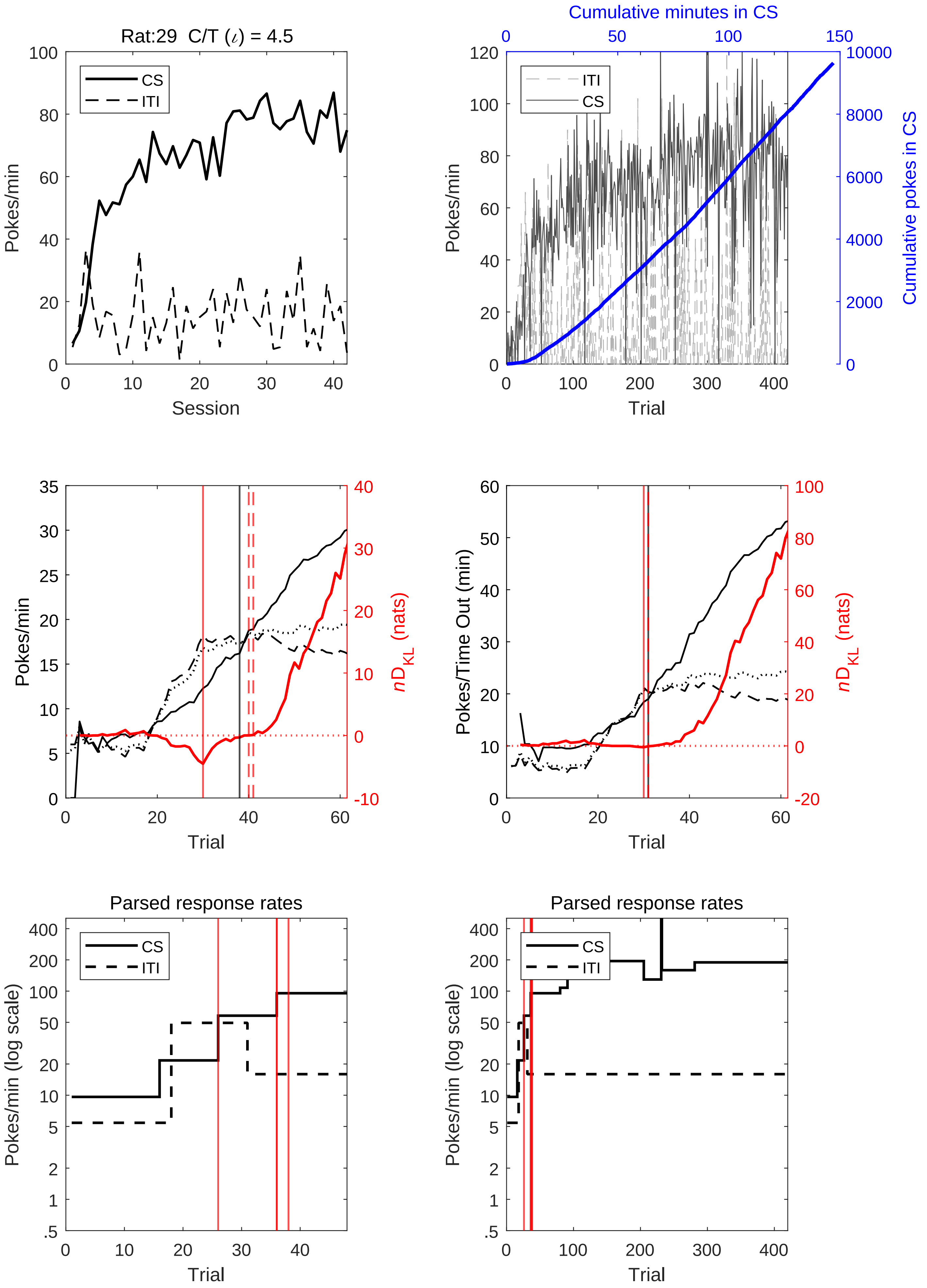
Rat:29 C/T (ι) = 4.5
Pokes/min
Session
CS
ITI
Cumulative minutes in CS
Cumulative pokes in CS
Trial
Pokes/Time Out (min)
$nD_{KL}$ (nats)
Parsed response rates
Pokes/min (log scale)

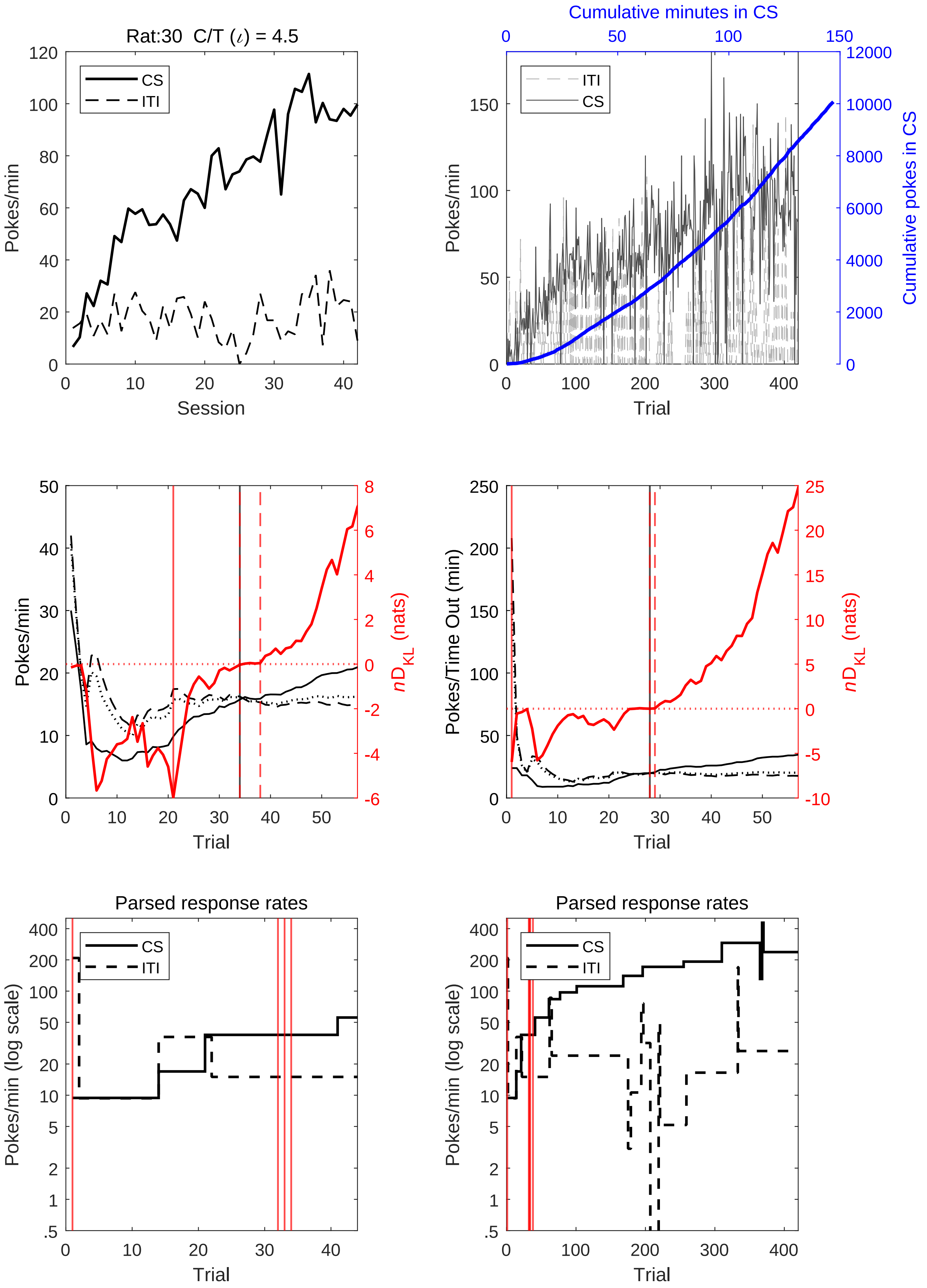
Rat:30 C/T ($\iota$) = 4.5
CS
ITI
Pokes/min
Session
Cumulative minutes in CS
Cumulative pokes in CS
Trial
$nD_{KL}$ (nats)
Pokes/Time Out (min)
Parsed response rates
Pokes/min (log scale)

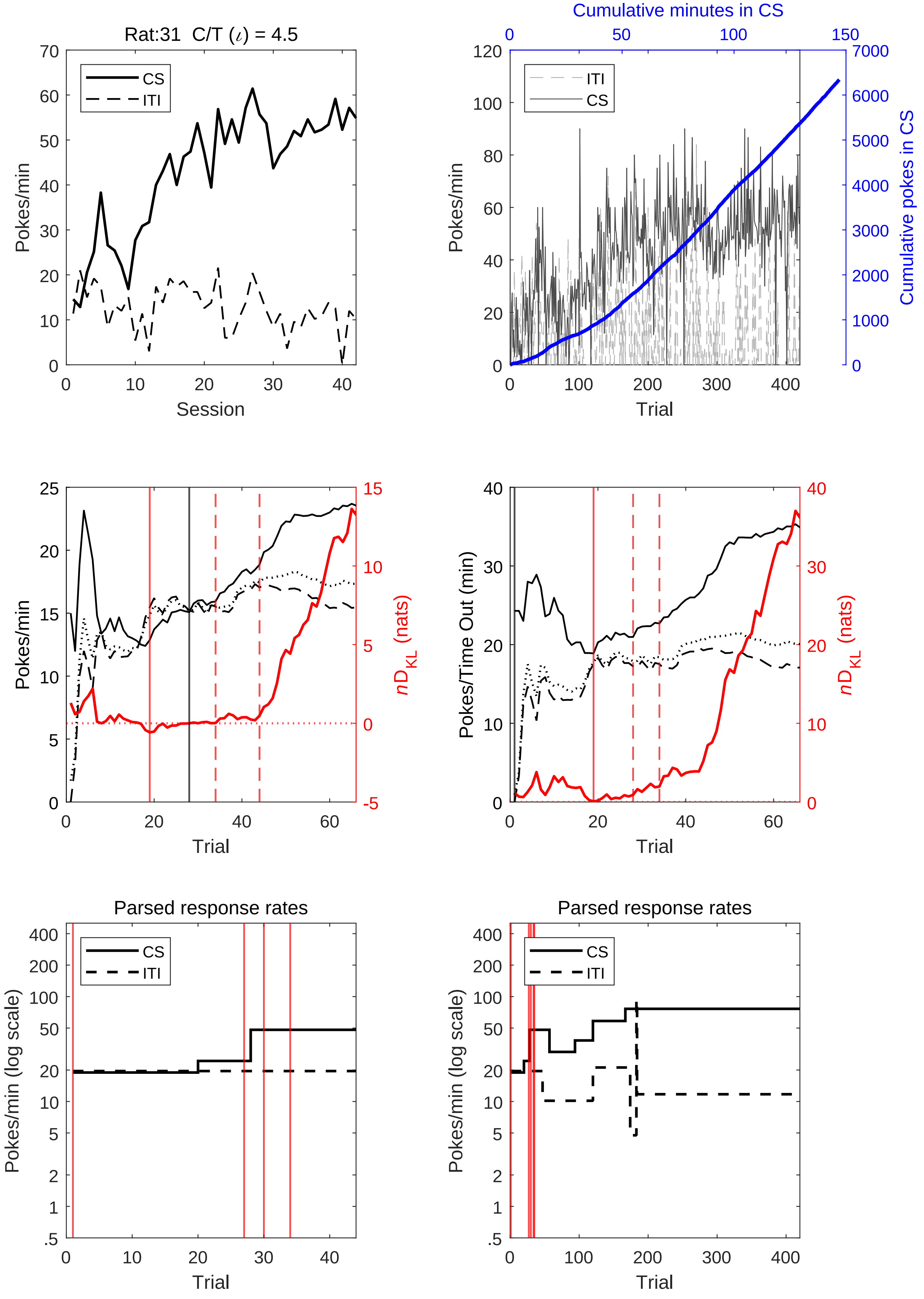

Rat:31  C/T ($\iota$) = 4.5
CS
ITI
Pokes/min
Session
Cumulative minutes in CS
Cumulative pokes in CS
Trial
Pokes/Time Out (min)
$nD_{KL}$ (nats)
Parsed response rates
Pokes/min (log scale)

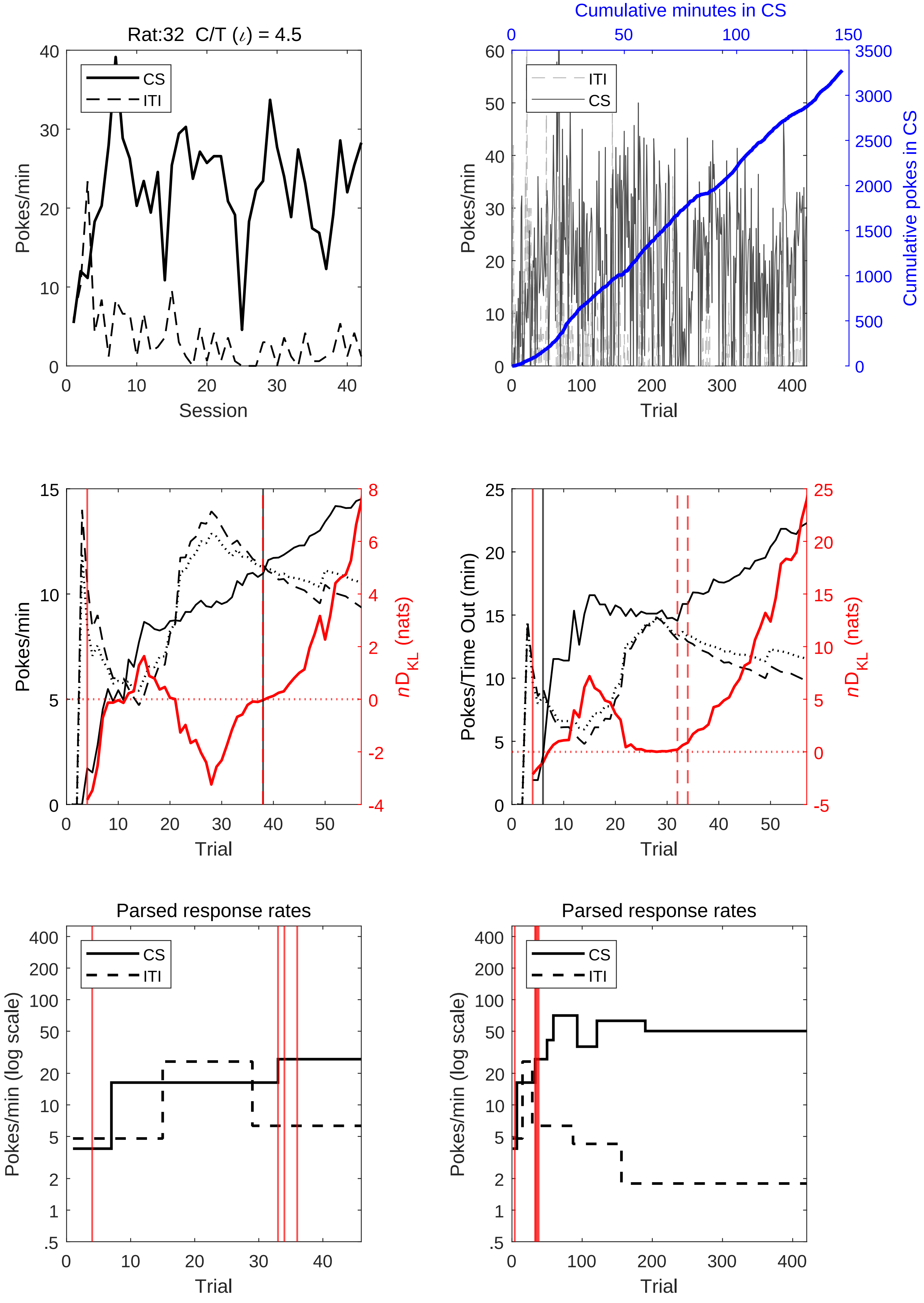

Rat:32 C/T (ι) = 4.5
CS
ITI
Pokes/min
Session
Cumulative minutes in CS
ITI
CS
Pokes/min
Cumulative pokes in CS
Trial
Pokes/min
nD_KL (nats)
Trial
Pokes/Time Out (min)
nD_KL (nats)
Trial
Parsed response rates
CS
ITI
Pokes/min (log scale)
Trial
Parsed response rates
CS
ITI
Pokes/min (log scale)
Trial

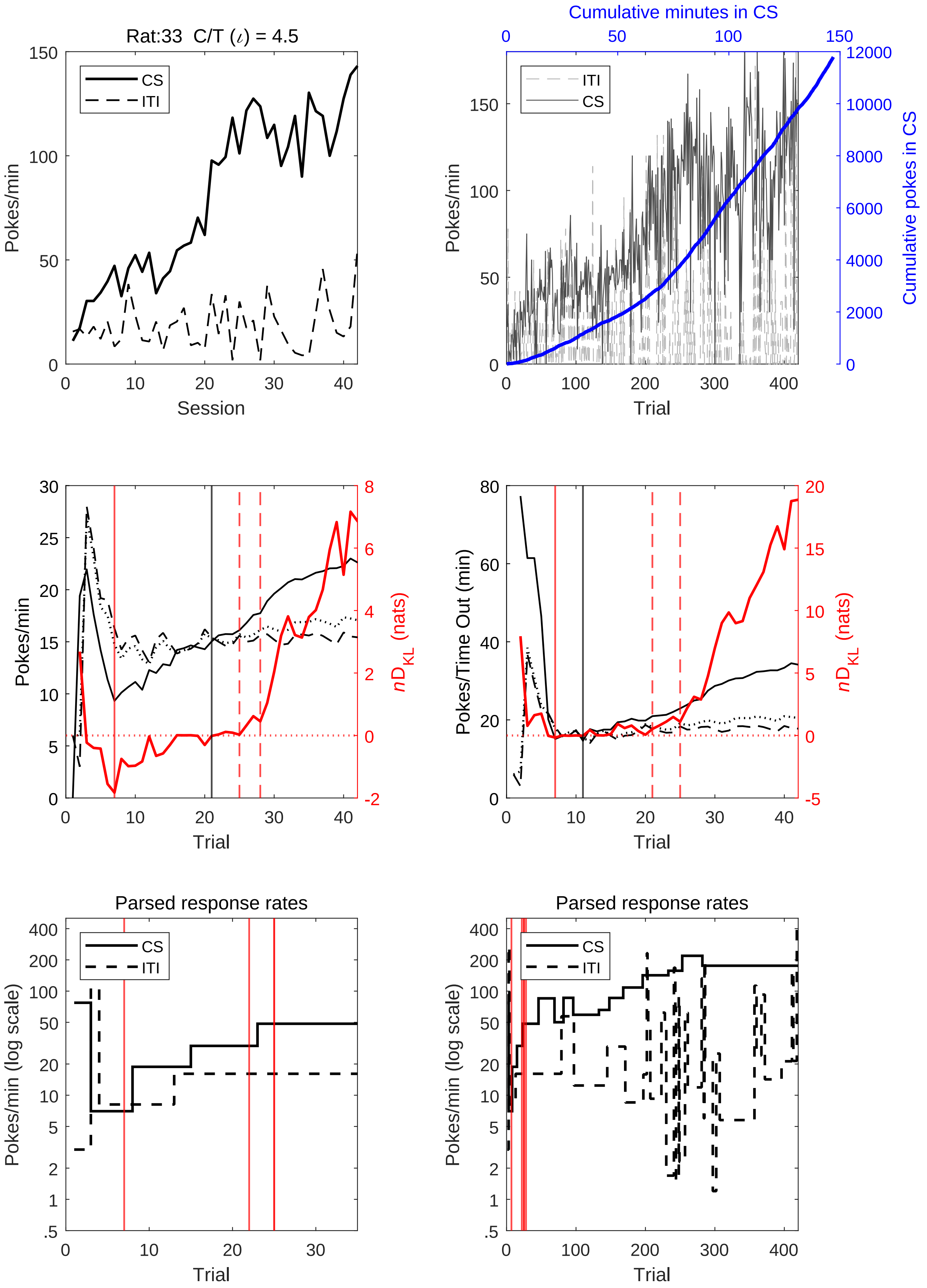
Rat:33 C/T ($\iota$) = 4.5
CS
ITI
Pokes/min
Session
Cumulative minutes in CS
Cumulative pokes in CS
Trial
Pokes/min
$nD_{KL}$ (nats)
Pokes/Time Out (min)
Parsed response rates
Pokes/min (log scale)

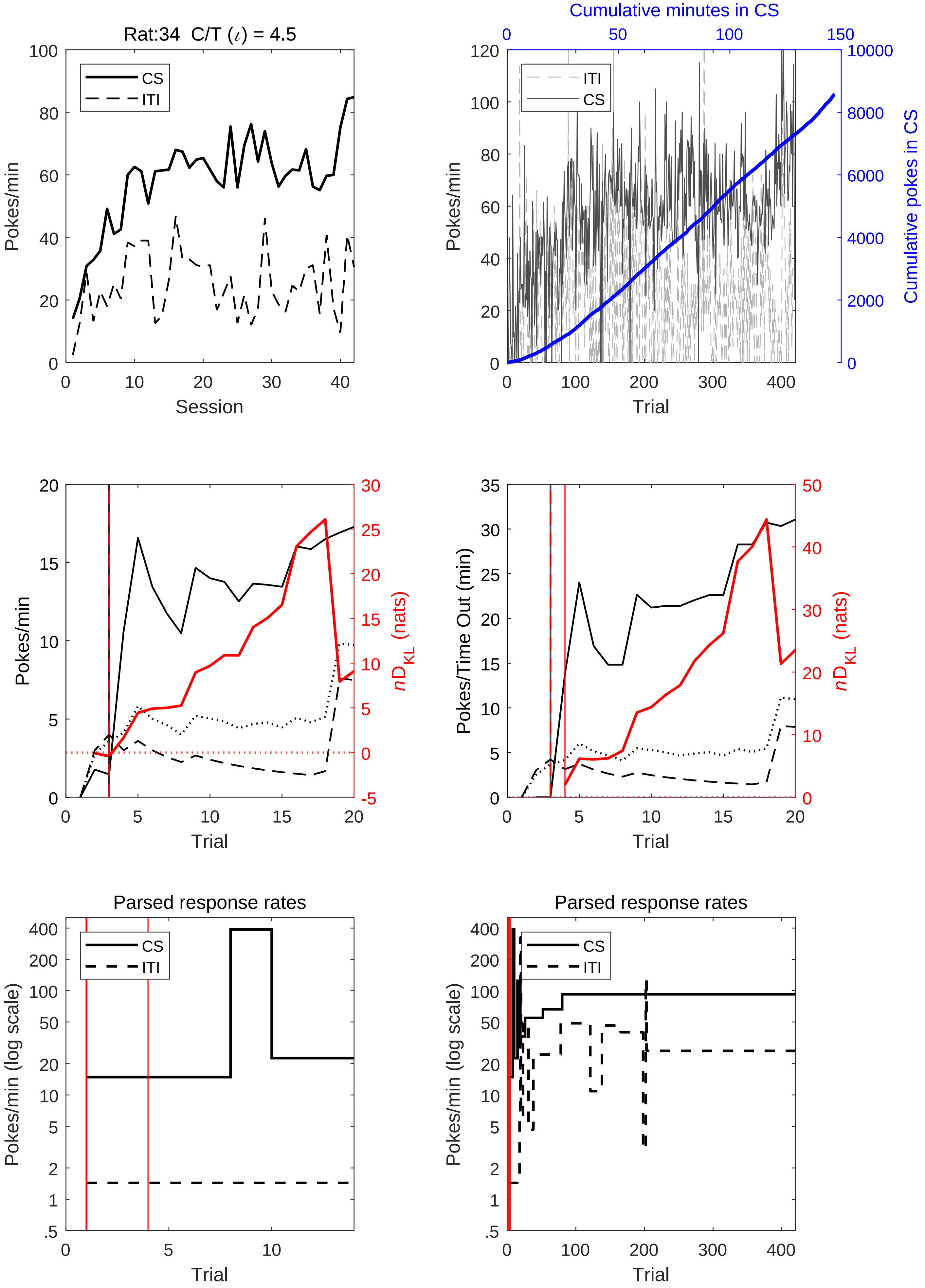
Rat:34 C/T (ι) = 4.5
CS
ITI
Pokes/min
Session
Cumulative minutes in CS
ITI
CS
Pokes/min
Cumulative pokes in CS
Trial
Pokes/min
nD_KL (nats)
Trial
Pokes/Time Out (min)
nD_KL (nats)
Trial
Parsed response rates
CS
ITI
Pokes/min (log scale)
Trial
Parsed response rates
CS
ITI
Pokes/min (log scale)
Trial

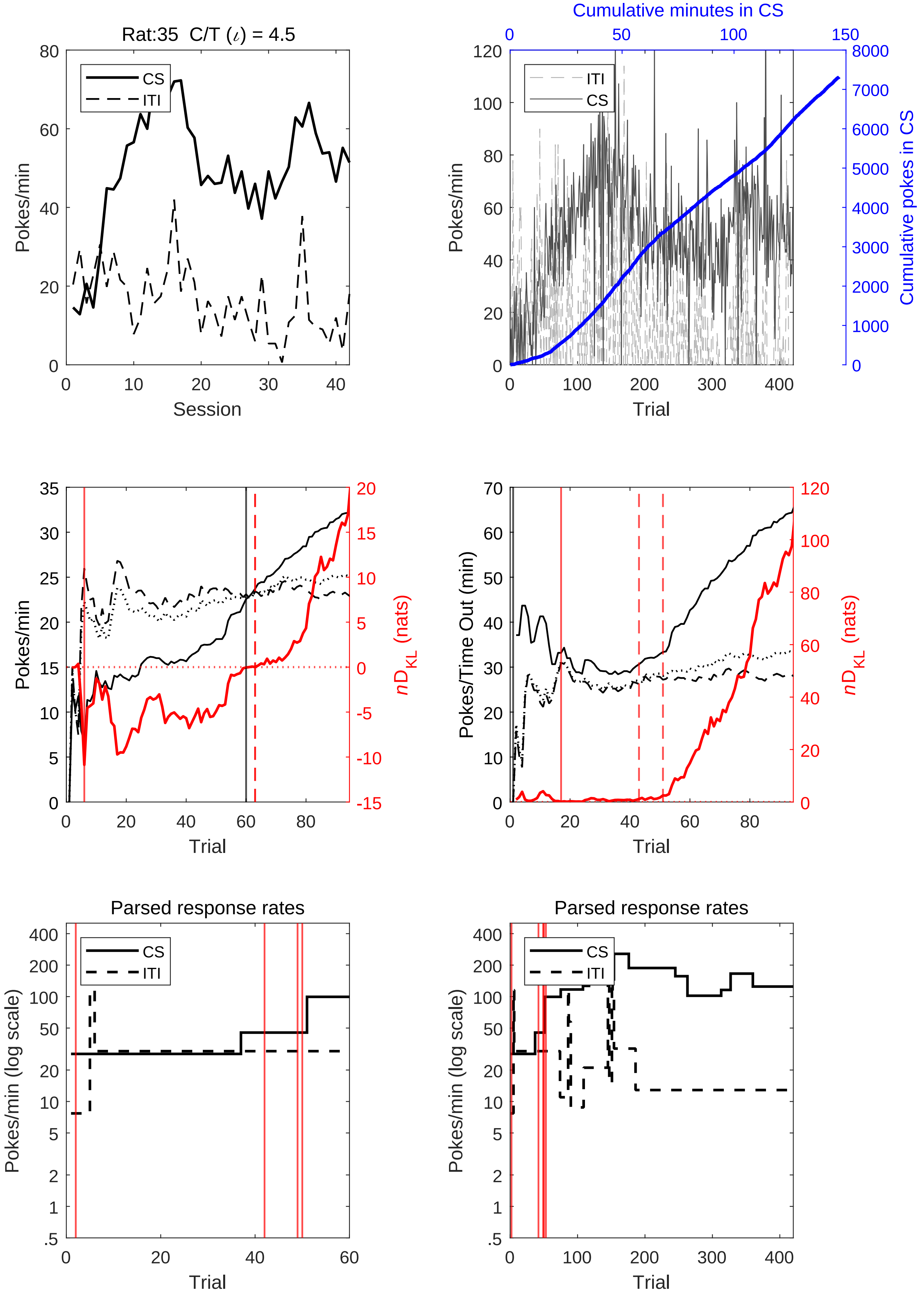
Rat:35 C/T (ι) = 4.5
CS
ITI
Pokes/min
Session
Cumulative minutes in CS
Cumulative pokes in CS
Trial
$nD_{KL}$ (nats)
Pokes/Time Out (min)
Parsed response rates
Pokes/min (log scale)

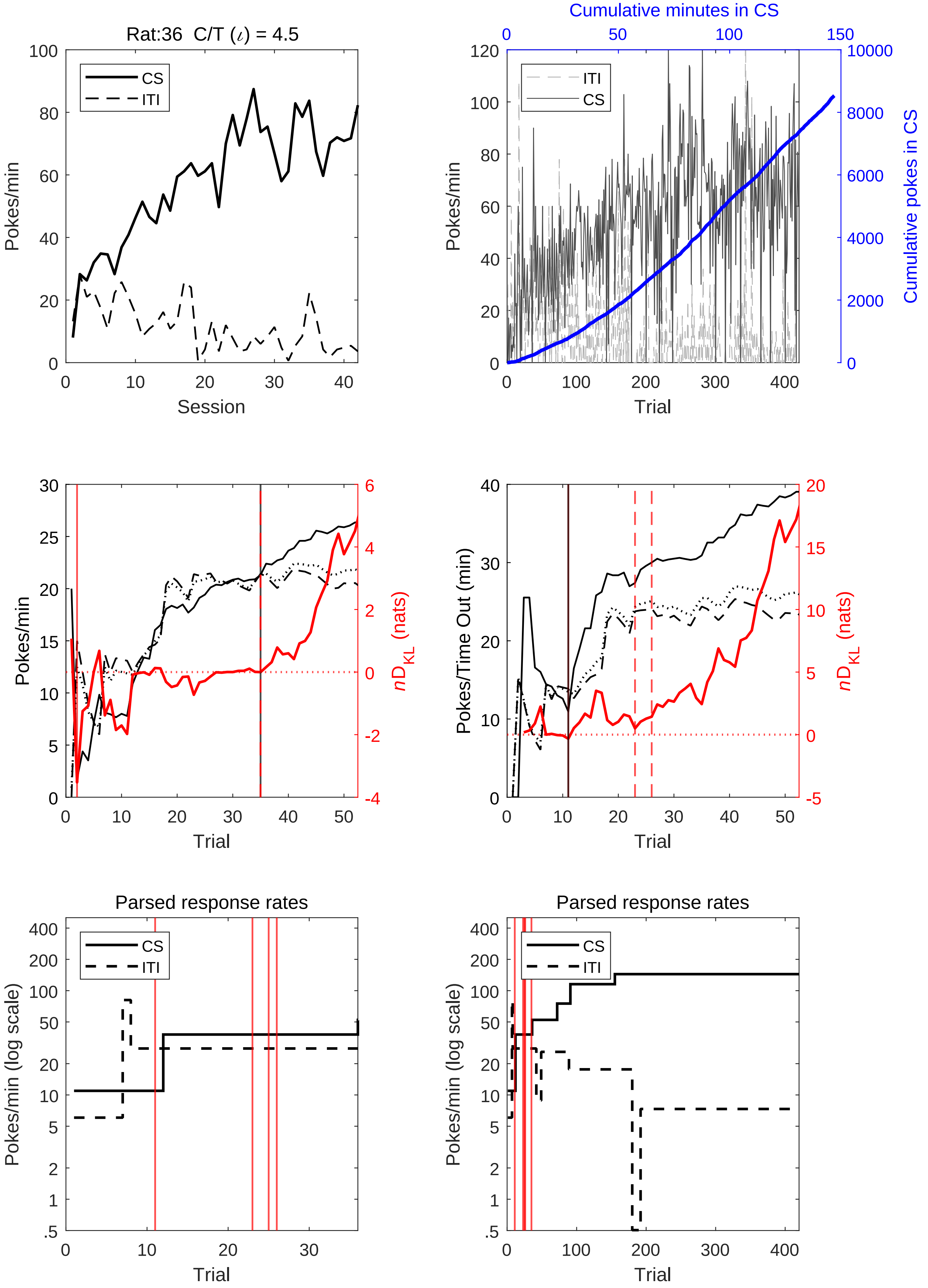
Rat:36 C/T (ι) = 4.5
CS
ITI
Pokes/min
Session
Cumulative minutes in CS
Cumulative pokes in CS
ITI
CS
Trial
Pokes/min
nD_KL (nats)
Pokes/Time Out (min)
Parsed response rates
Pokes/min (log scale)
CS
ITI

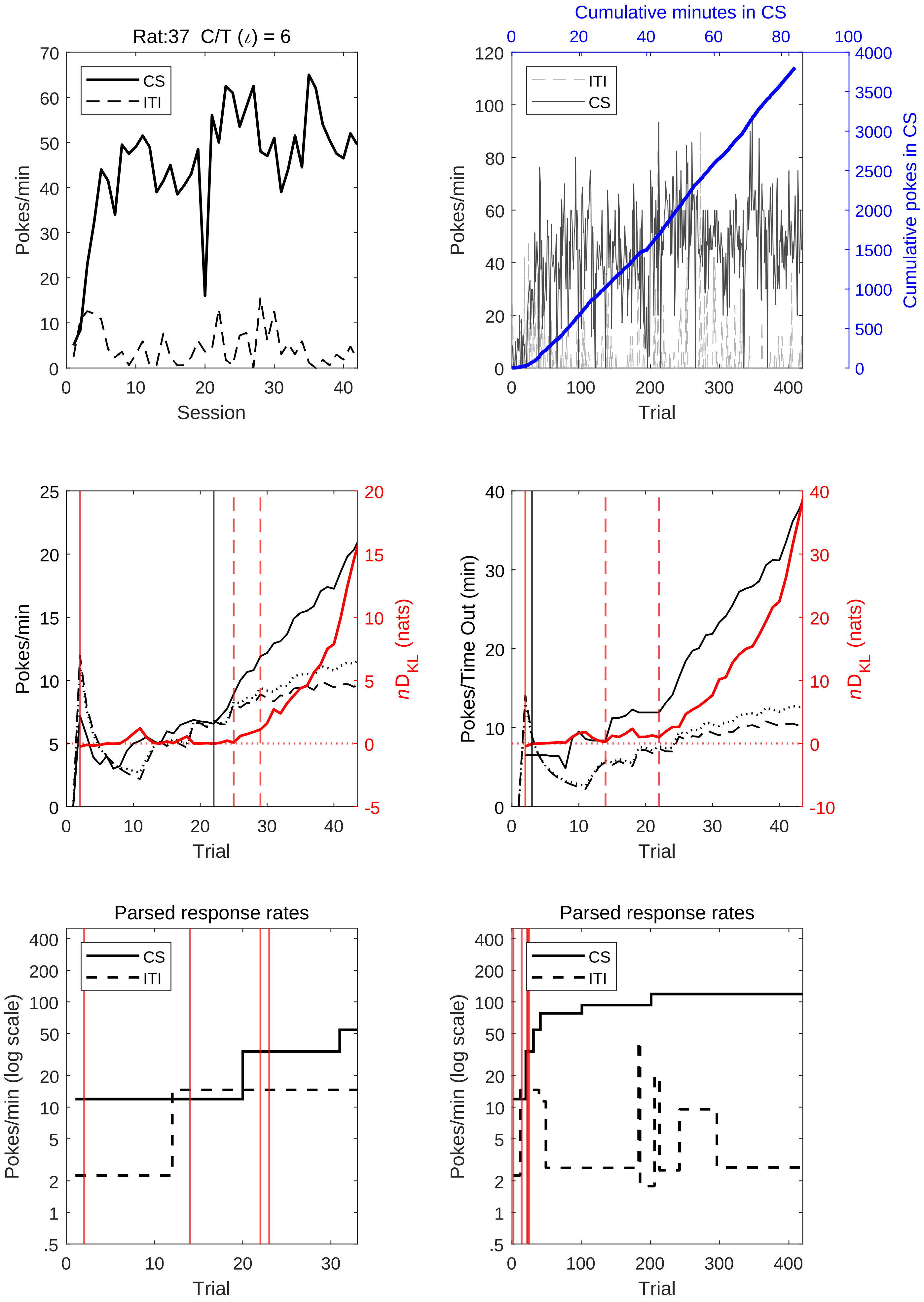

Rat:37 C/T (ι) = 6
CS
ITI
Pokes/min
Session
Cumulative minutes in CS
Cumulative pokes in CS
Trial
nD_KL (nats)
Pokes/Time Out (min)
Parsed response rates
Pokes/min (log scale)

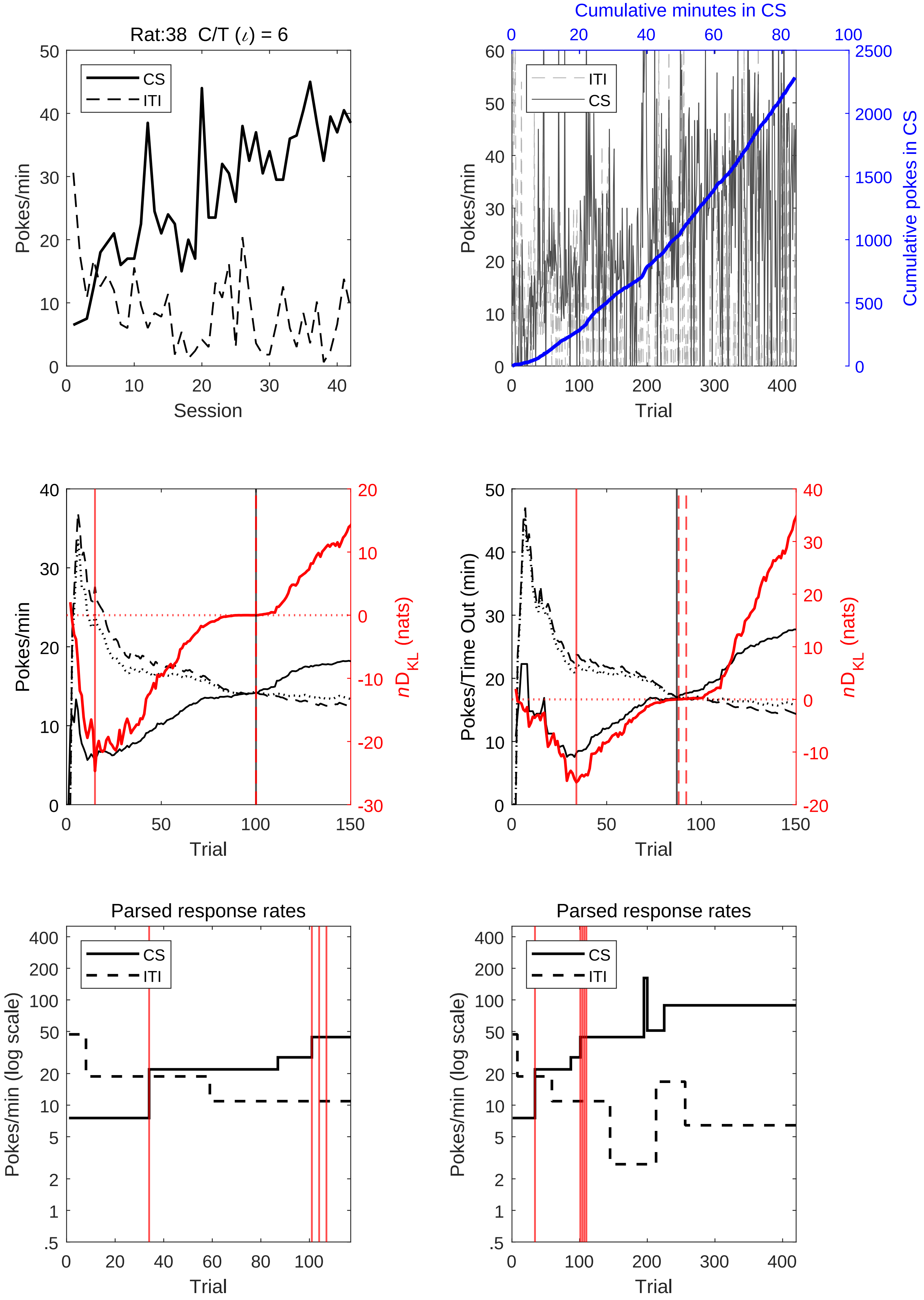
Rat:38 C/T (ι) = 6
CS
ITI
Pokes/min
Session
Cumulative minutes in CS
Cumulative pokes in CS
Trial
nD_KL (nats)
Pokes/Time Out (min)
Parsed response rates
Pokes/min (log scale)

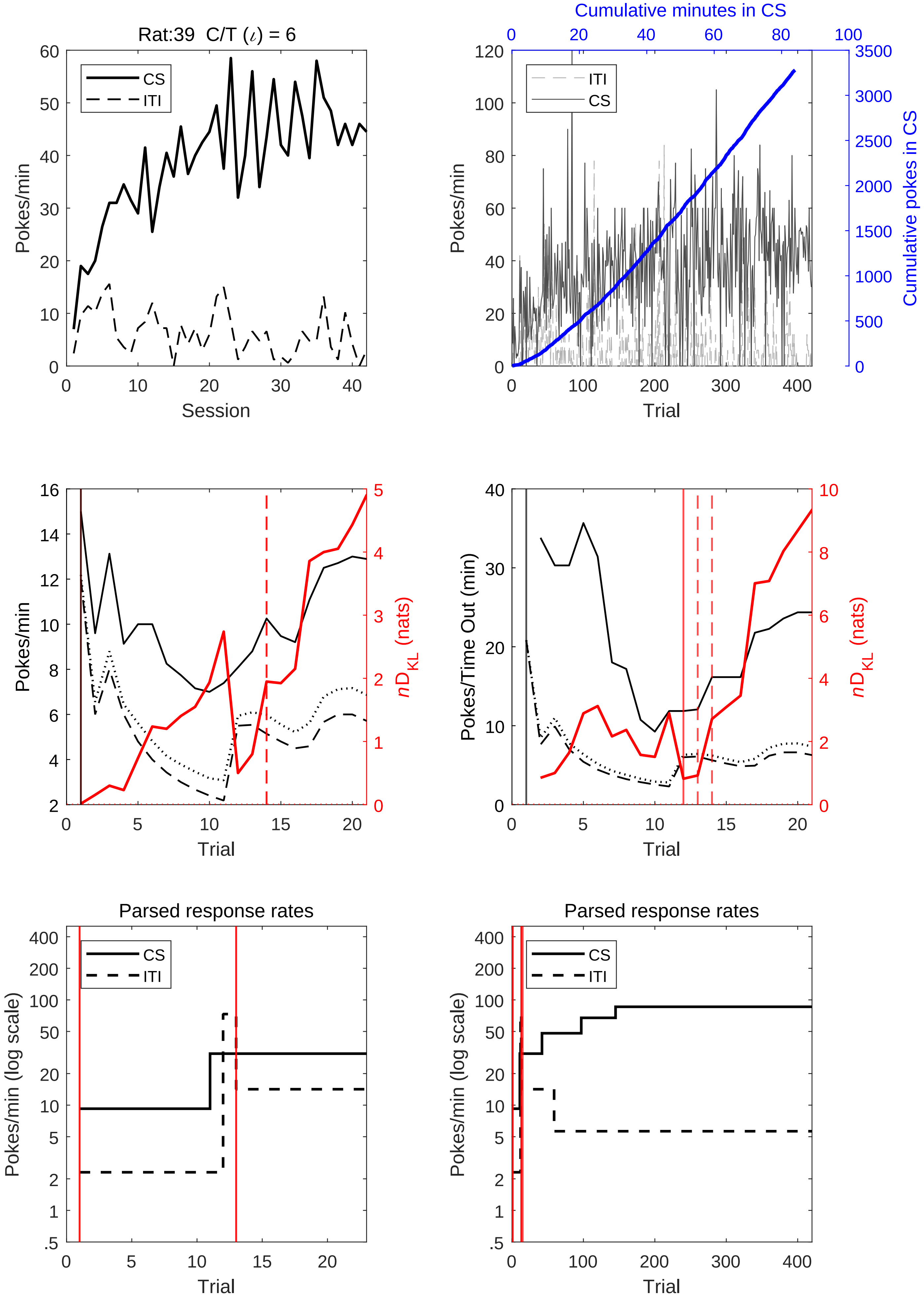
Rat:39 C/T (ι) = 6
CS
ITI
Pokes/min
Session
Cumulative minutes in CS
Cumulative pokes in CS
Trial
nD_KL (nats)
Pokes/Time Out (min)
Parsed response rates
Pokes/min (log scale)

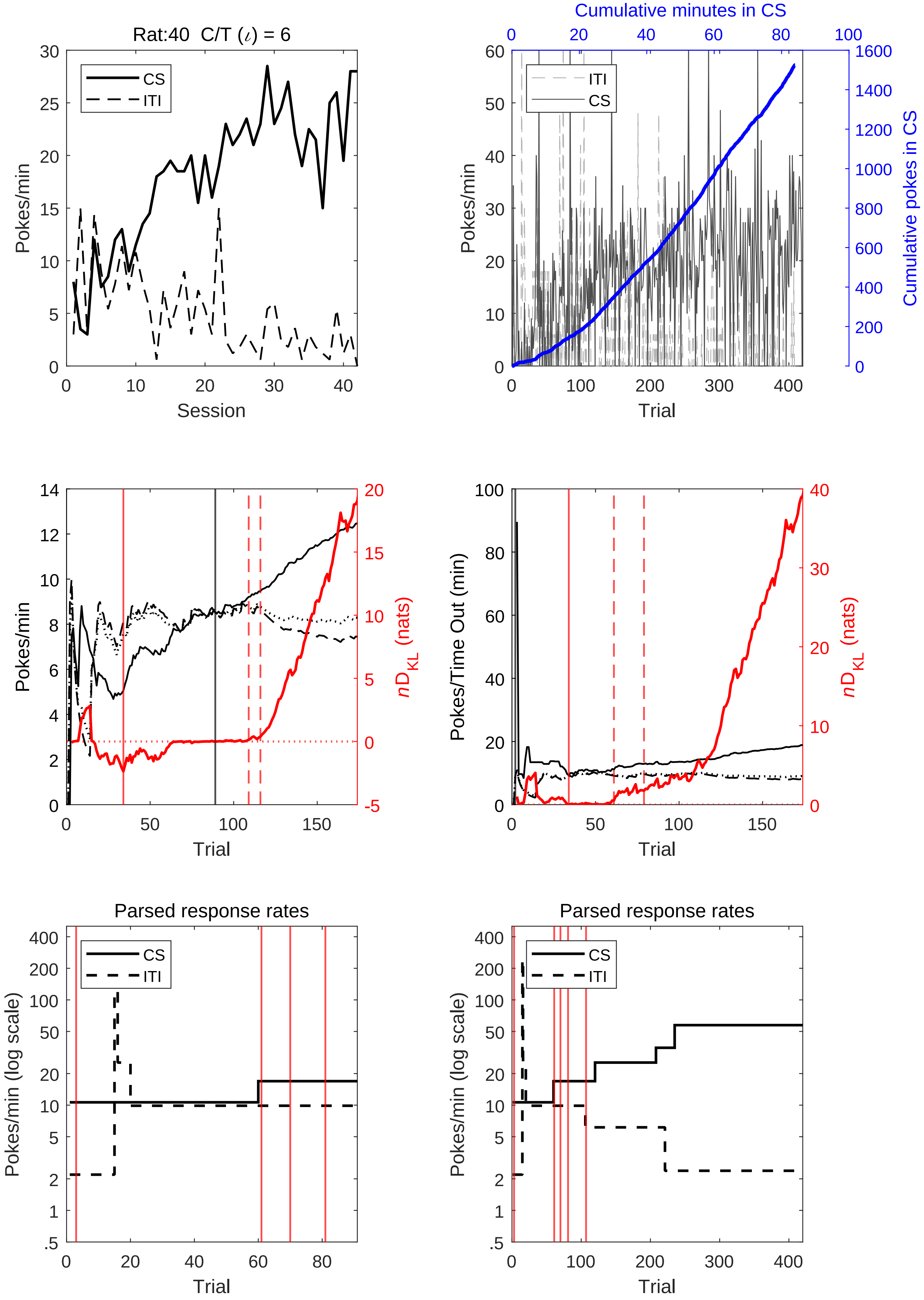

Rat:40 C/T (ι) = 6
CS
ITI
Pokes/min
Session
Cumulative minutes in CS
Cumulative pokes in CS
Trial
nD_KL (nats)
Pokes/Time Out (min)
Parsed response rates
Pokes/min (log scale)

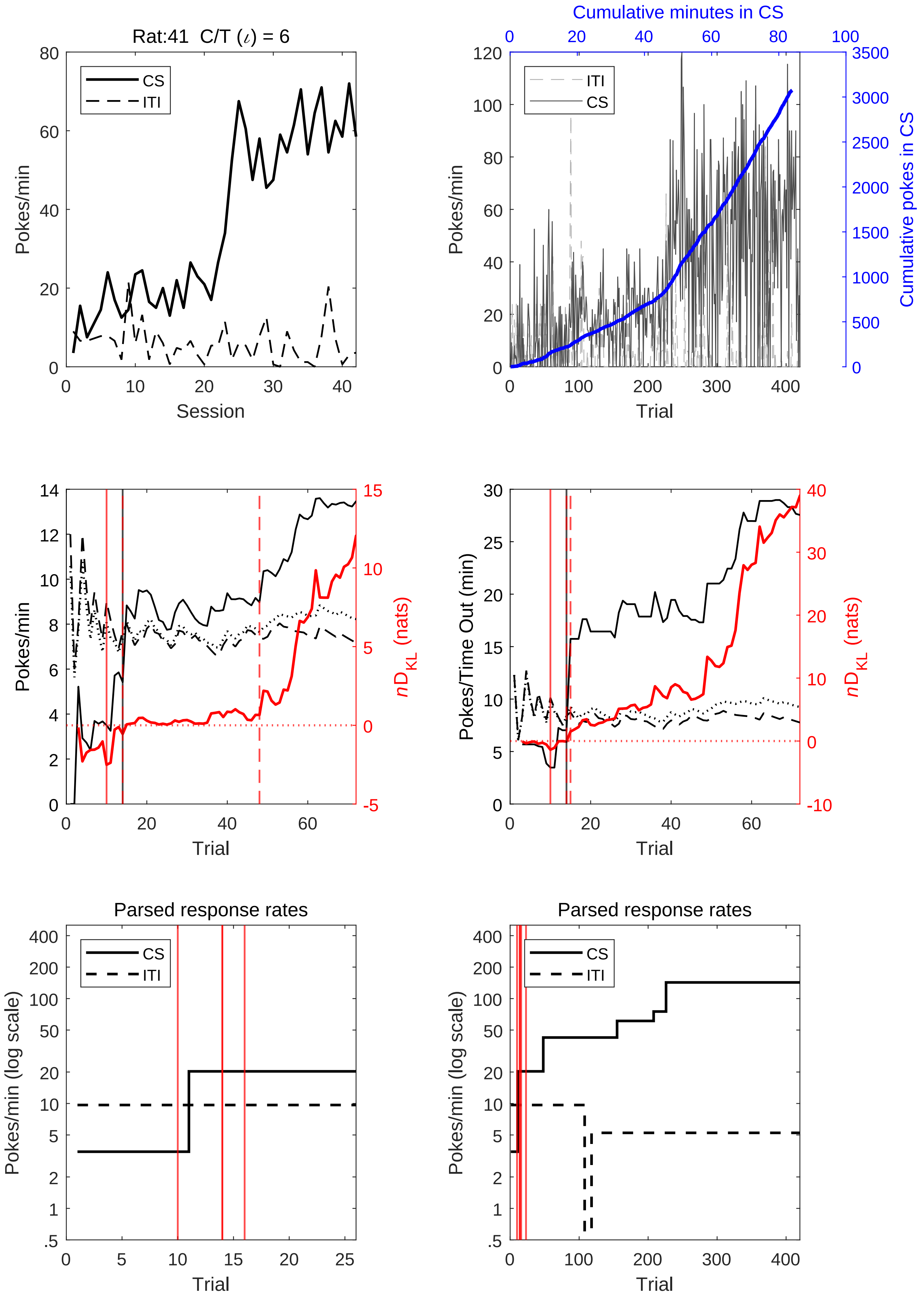
Rat:41 C/T (ι) = 6
CS
ITI
Pokes/min
Session
Cumulative minutes in CS
Cumulative pokes in CS
ITI
CS
Pokes/min
Trial
Pokes/min
$nD_{KL}$ (nats)
Trial
Pokes/Time Out (min)
$nD_{KL}$ (nats)
Trial
Parsed response rates
CS
ITI
Pokes/min (log scale)
Trial
Parsed response rates
CS
ITI
Pokes/min (log scale)
Trial

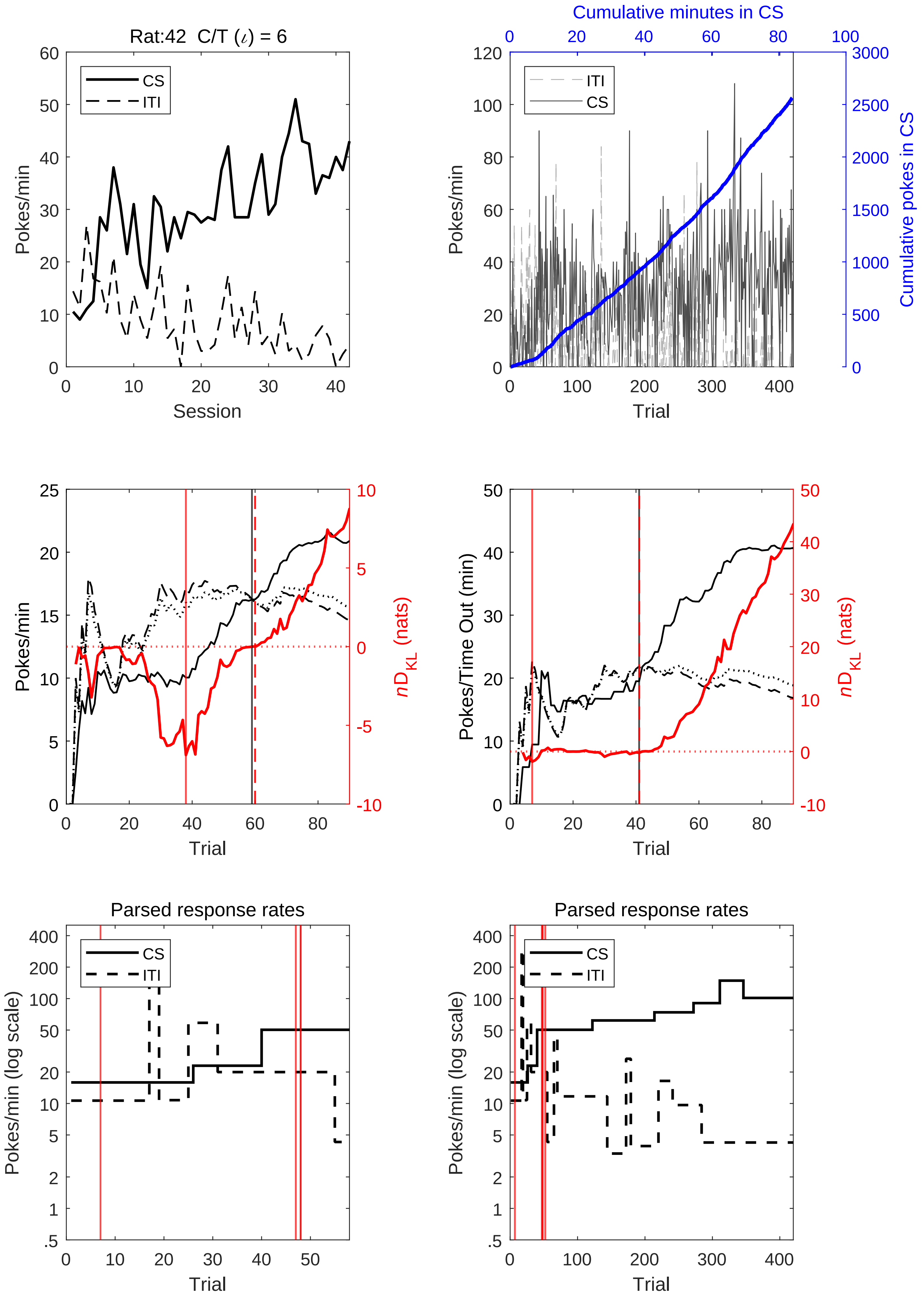
Rat:42 C/T (ι) = 6
CS
ITI
Pokes/min
Session
Cumulative minutes in CS
Cumulative pokes in CS
Trial
nD_KL (nats)
Pokes/Time Out (min)
Parsed response rates
Pokes/min (log scale)

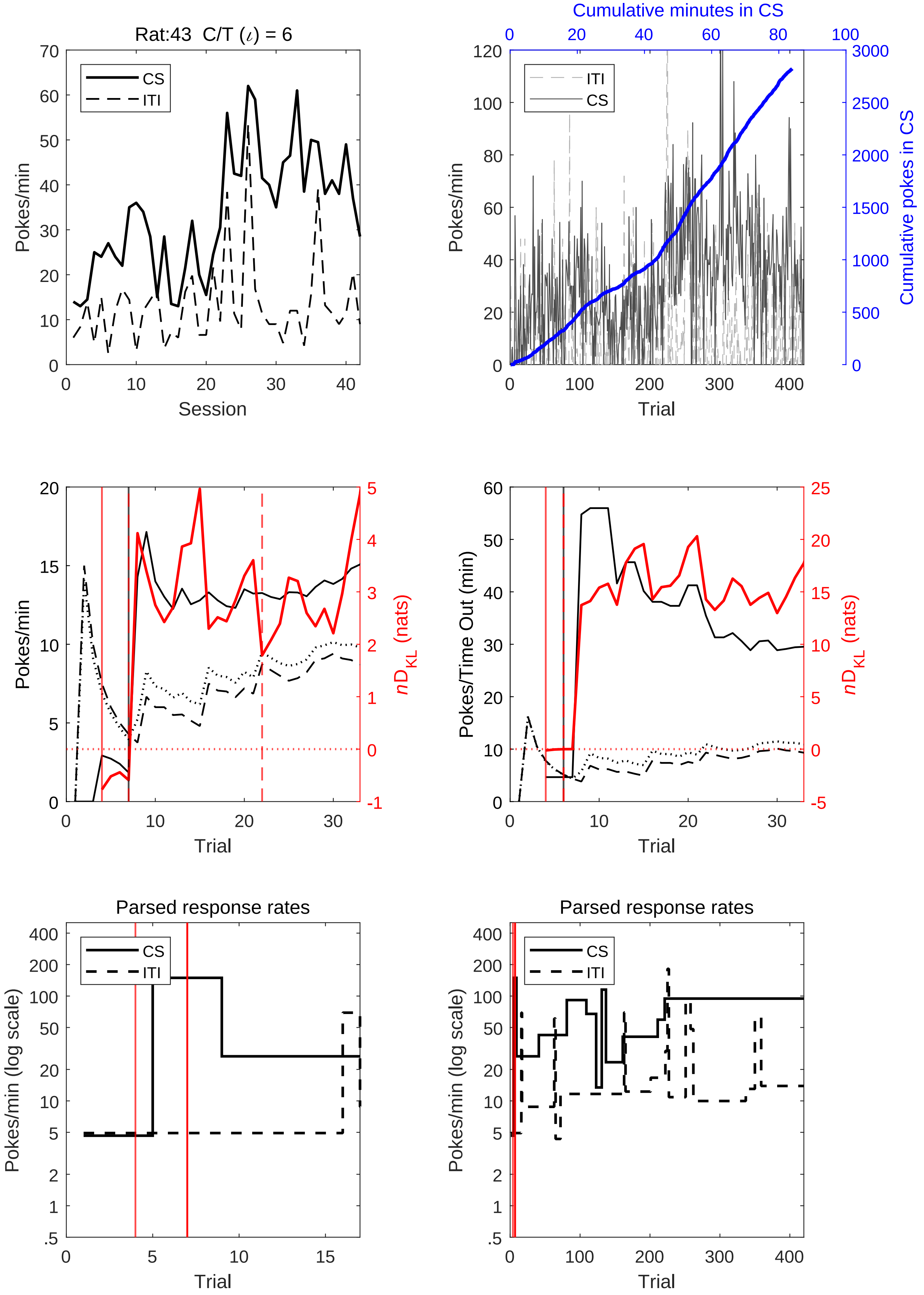
Rat:43 C/T (ι) = 6
CS
ITI
Pokes/min
Session
Cumulative minutes in CS
Cumulative pokes in CS
Trial
nD_KL (nats)
Pokes/Time Out (min)
Parsed response rates
Pokes/min (log scale)

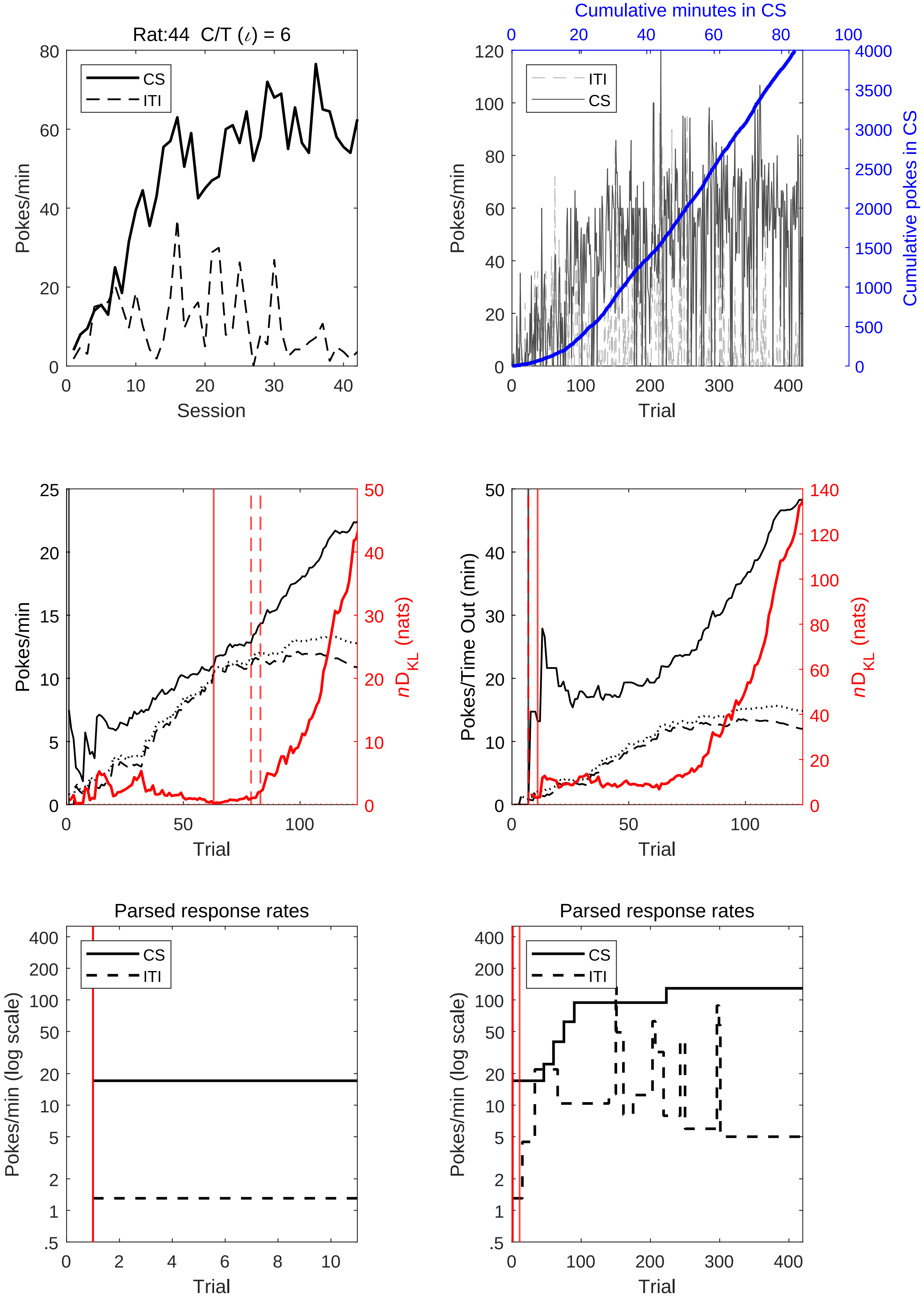

Rat:44 C/T (ι) = 6
CS
ITI
Pokes/min
Session
Cumulative minutes in CS
Cumulative pokes in CS
Trial
nD_KL (nats)
Pokes/Time Out (min)
Parsed response rates
Pokes/min (log scale)

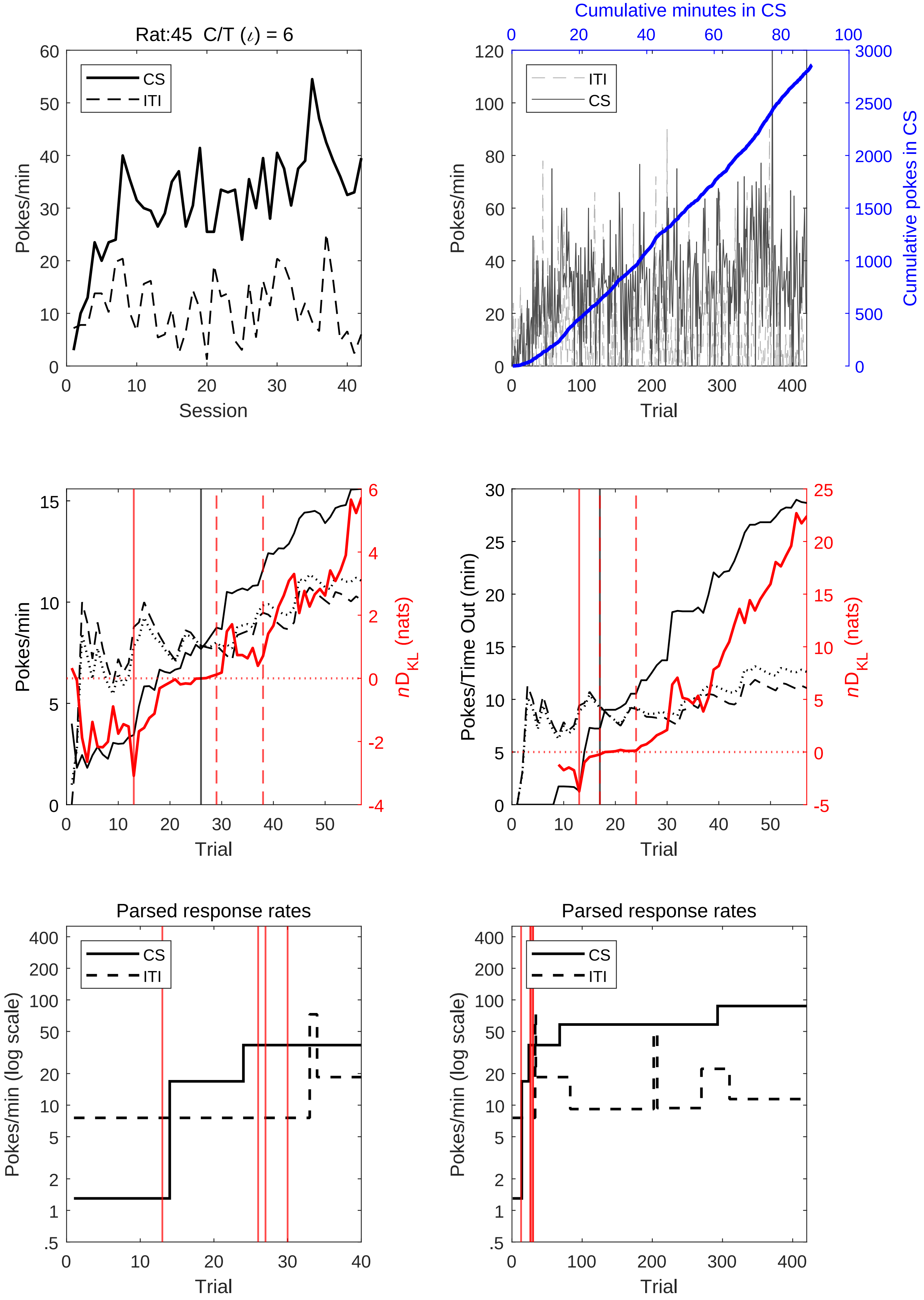
Rat:45 C/T (ι) = 6
CS
ITI
Pokes/min
Session
Cumulative minutes in CS
Cumulative pokes in CS
Trial
$nD_{KL}$ (nats)
Pokes/Time Out (min)
Parsed response rates
Pokes/min (log scale)

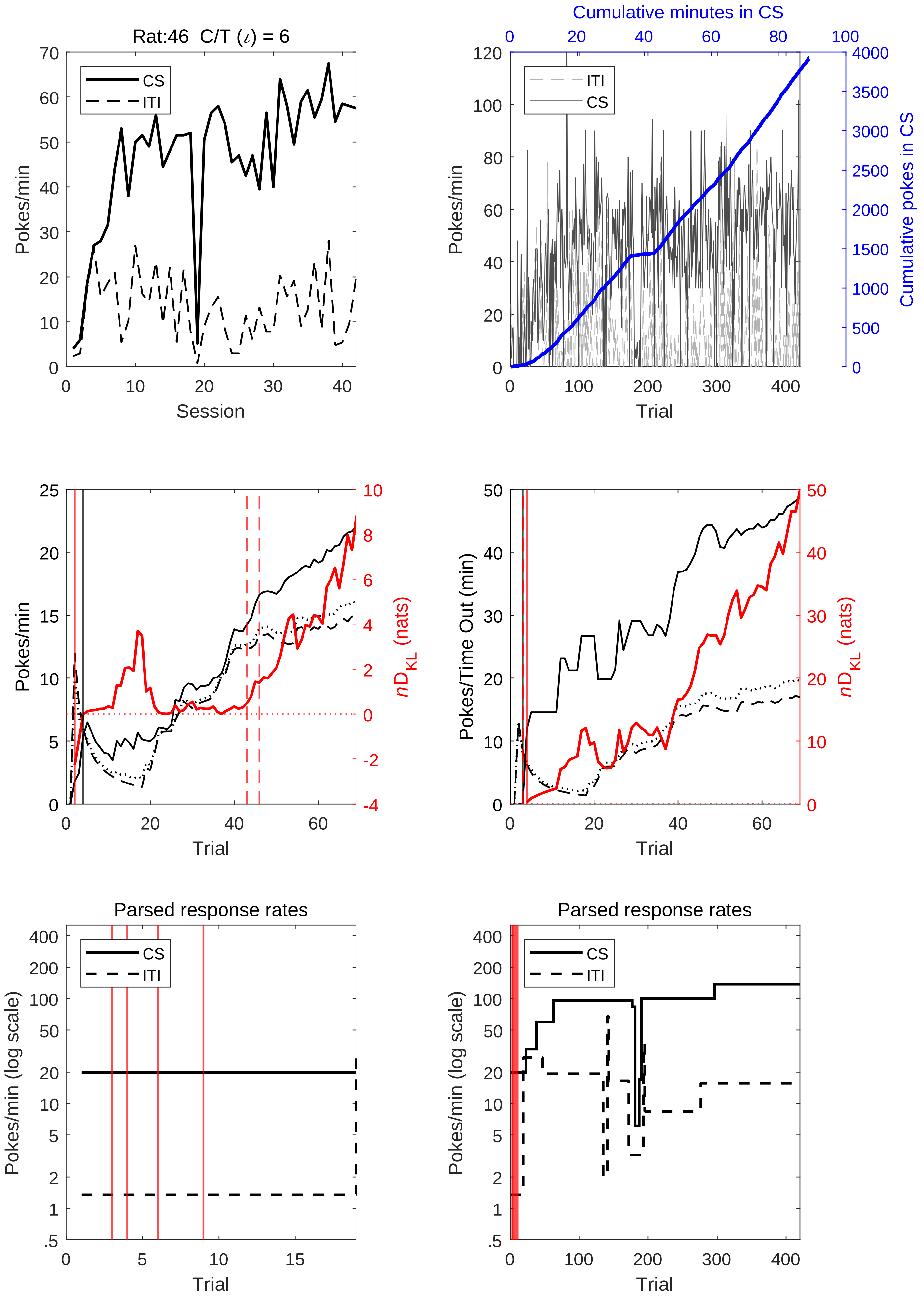
Rat:46 C/T (ι) = 6
CS
ITI
Pokes/min
Session
Cumulative minutes in CS
Cumulative pokes in CS
Trial
nD_KL (nats)
Pokes/Time Out (min)
Parsed response rates
Pokes/min (log scale)

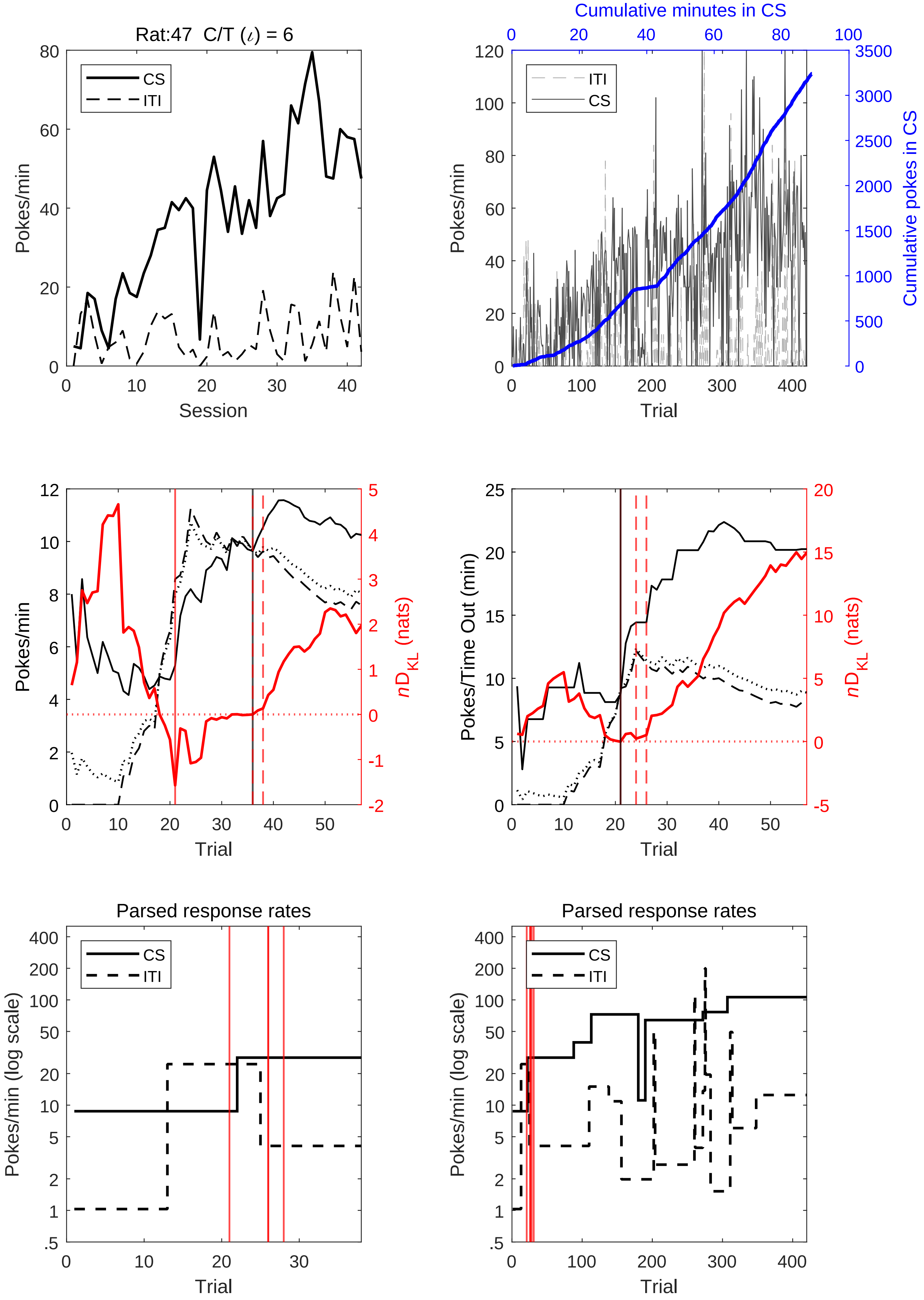
Rat:47 C/T (ι) = 6
CS
ITI
Pokes/min
Session
Cumulative minutes in CS
Cumulative pokes in CS
Trial
Pokes/Time Out (min)
nD_KL (nats)
Parsed response rates
Pokes/min (log scale)

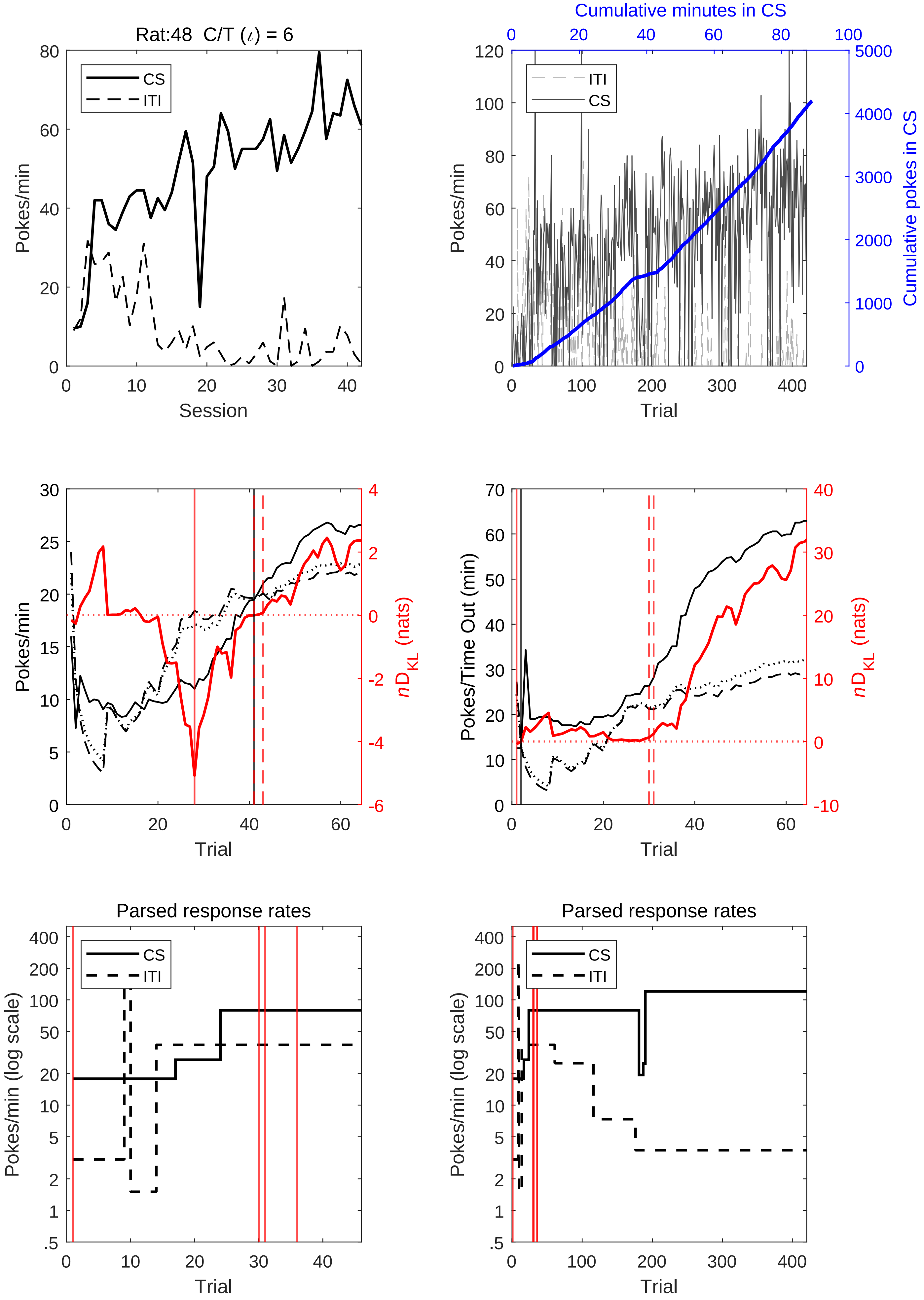

Rat:48 C/T (ι) = 6
CS
ITI
Pokes/min
Session
Cumulative minutes in CS
Cumulative pokes in CS
Trial
nD_KL (nats)
Pokes/Time Out (min)
Parsed response rates
Pokes/min (log scale)

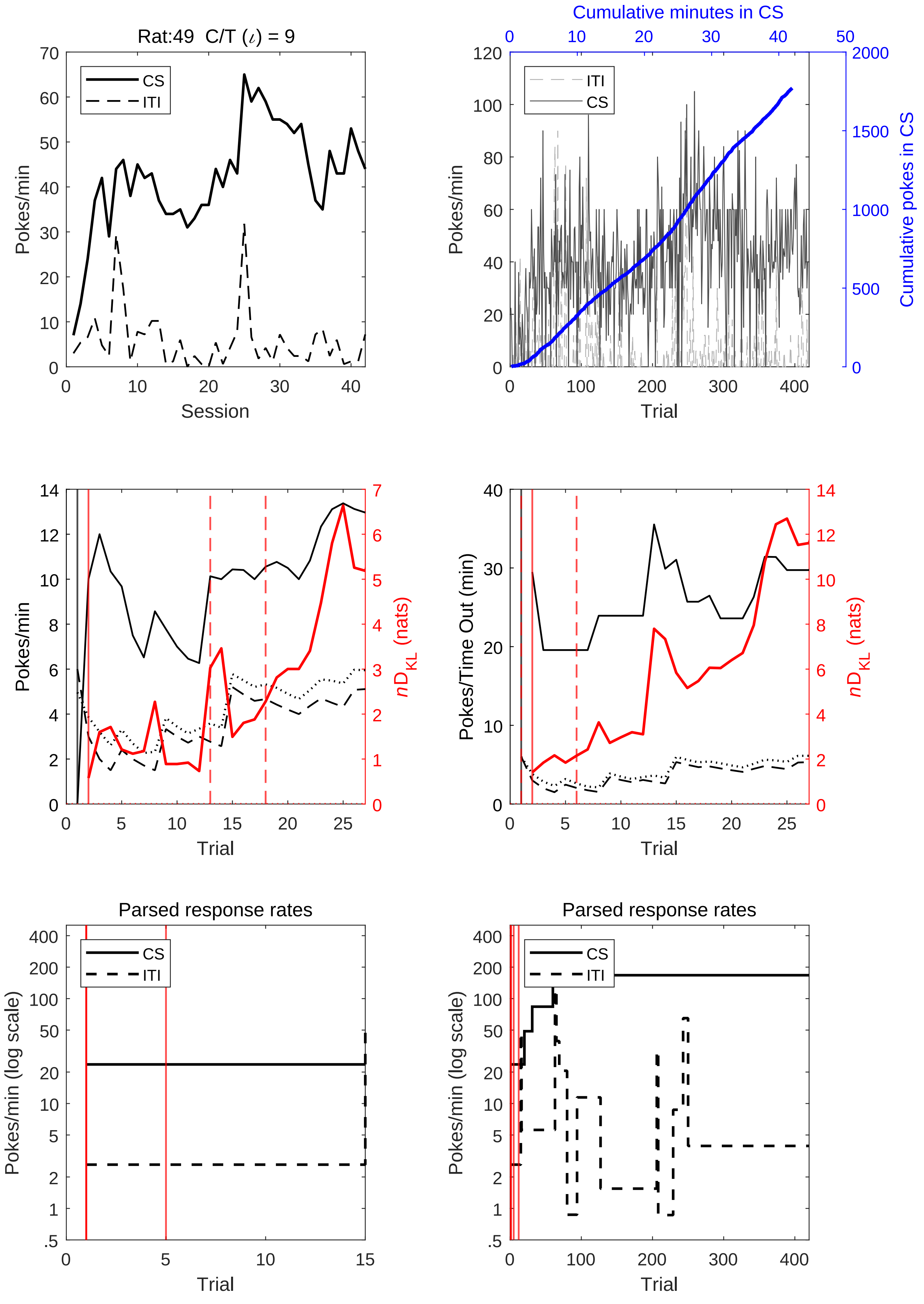
Rat:49  C/T (ι) = 9
Pokes/min
Session
CS
ITI
Cumulative minutes in CS
Cumulative pokes in CS
Trial
Pokes/min
nD_KL (nats)
Pokes/Time Out (min)
Parsed response rates
Pokes/min (log scale)

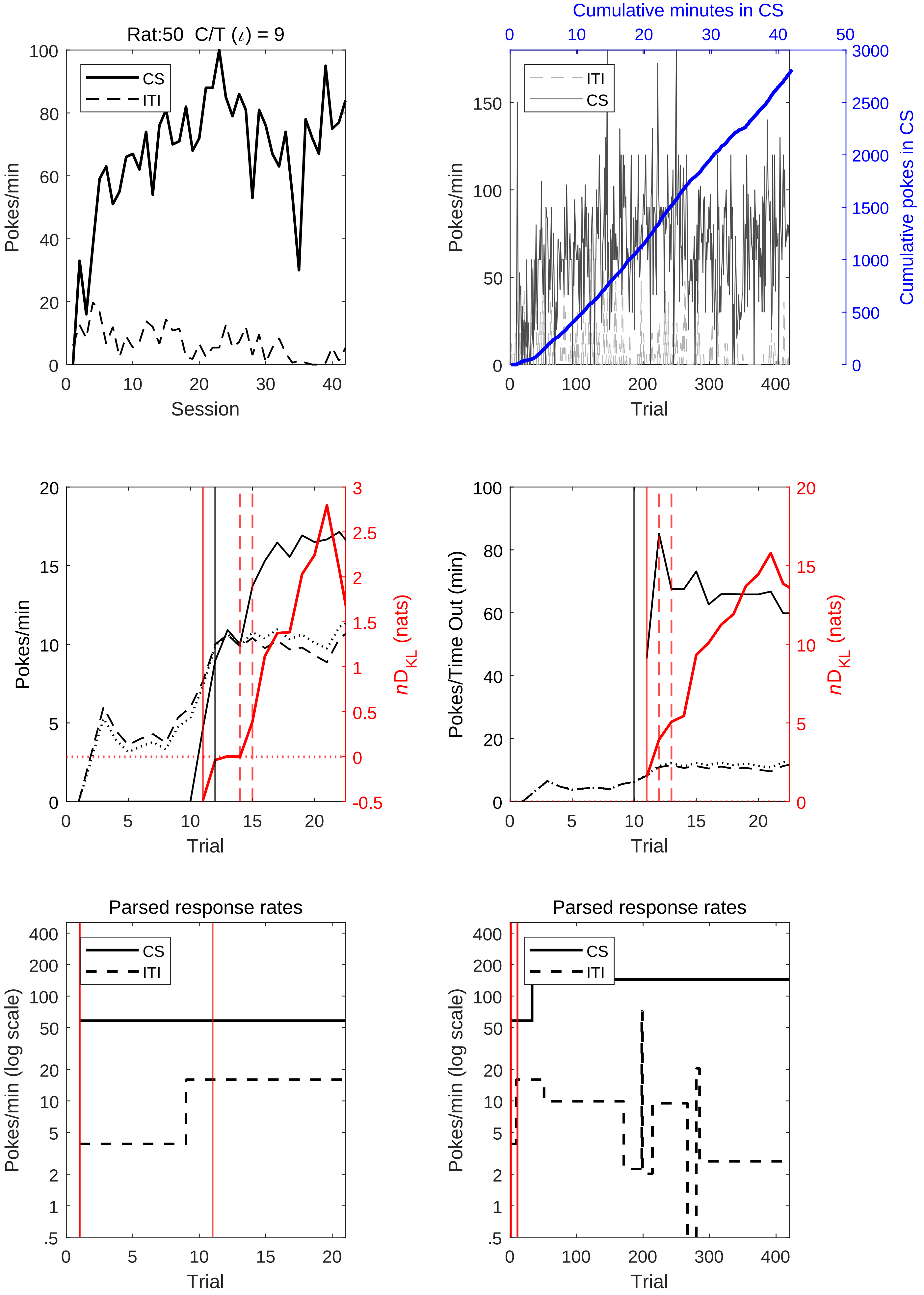
Rat:50 C/T (ι) = 9
CS
ITI
Pokes/min
Session
Cumulative minutes in CS
Cumulative pokes in CS
Trial
nD_KL (nats)
Pokes/Time Out (min)
Parsed response rates
Pokes/min (log scale)

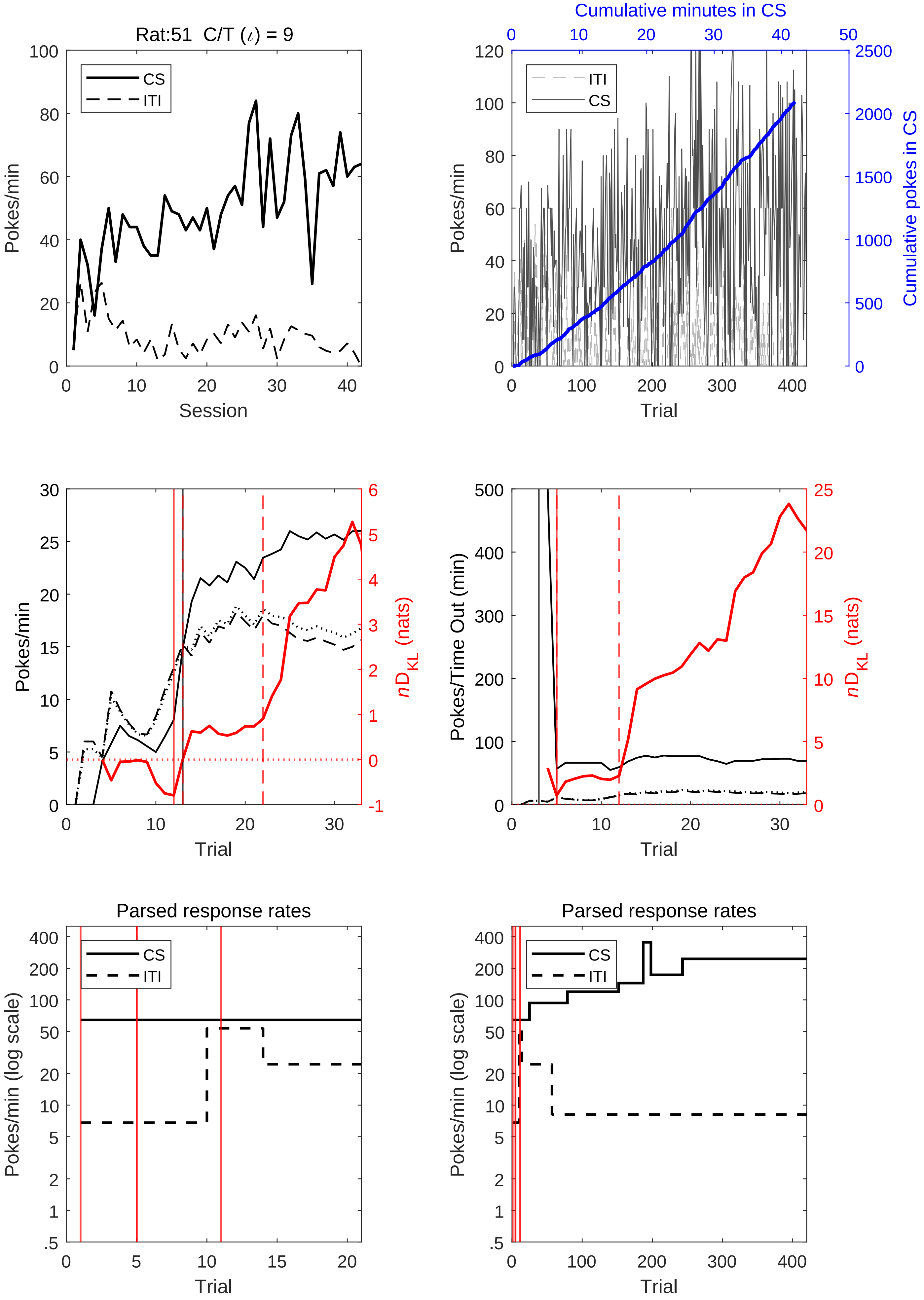
Rat:51 C/T (ι) = 9
CS
ITI
Pokes/min
Session
Cumulative minutes in CS
Cumulative pokes in CS
Trial
$nD_{KL}$ (nats)
Pokes/Time Out (min)
Parsed response rates
Pokes/min (log scale)

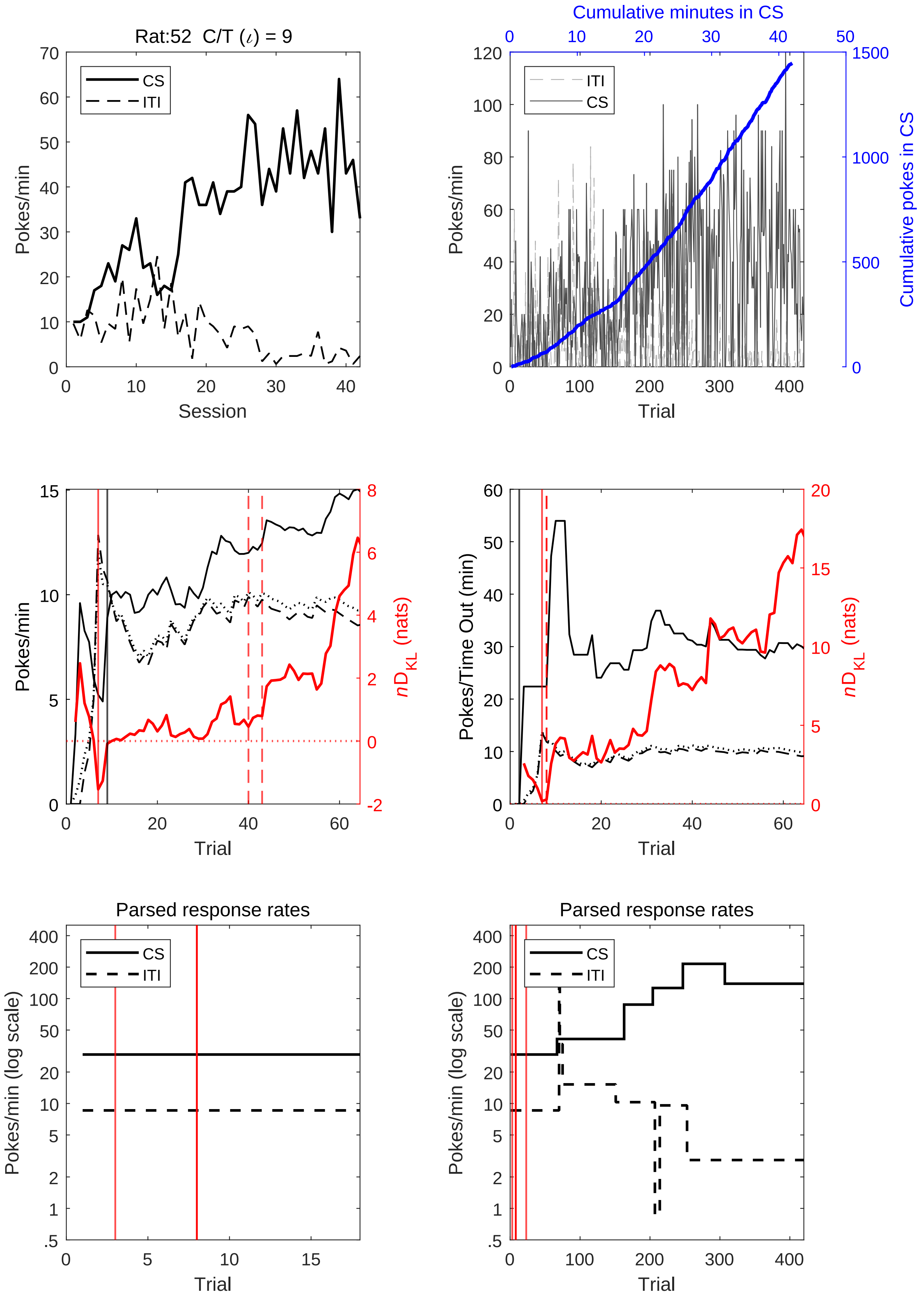
Rat:52 C/T (ι) = 9
CS
ITI
Pokes/min
Session
Cumulative minutes in CS
Cumulative pokes in CS
Trial
Pokes/Time Out (min)
nD_KL (nats)
Parsed response rates
Pokes/min (log scale)

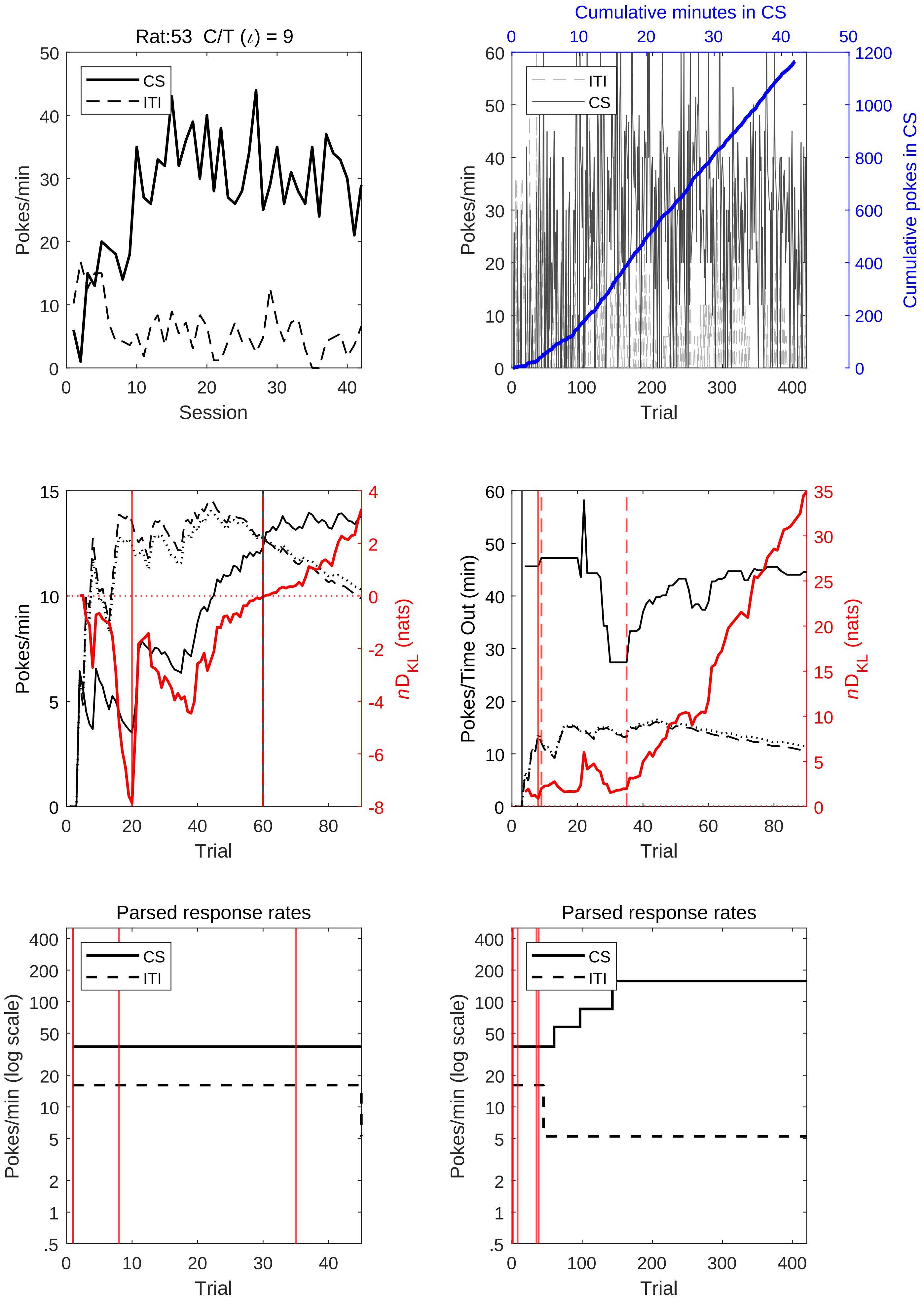
Rat:53 C/T (ι) = 9
CS
ITI
Pokes/min
Session
Cumulative minutes in CS
Cumulative pokes in CS
Trial
nD_KL (nats)
Pokes/Time Out (min)
Parsed response rates
Pokes/min (log scale)

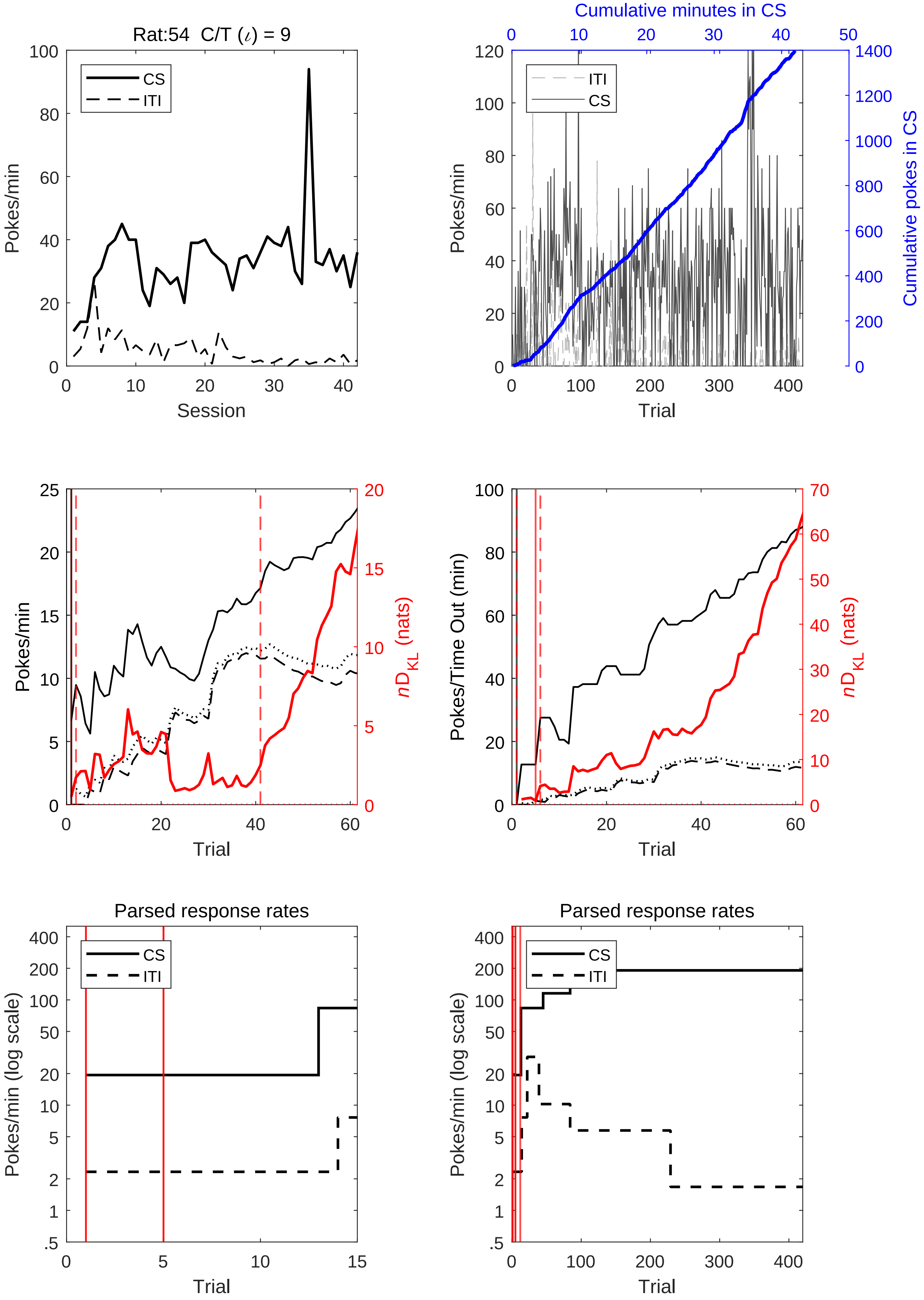

Rat:54 C/T (ι) = 9
CS
ITI
Pokes/min
Session
Cumulative minutes in CS
Cumulative pokes in CS
Trial
Pokes/Time Out (min)
$nD_{KL}$ (nats)
Parsed response rates
Pokes/min (log scale)

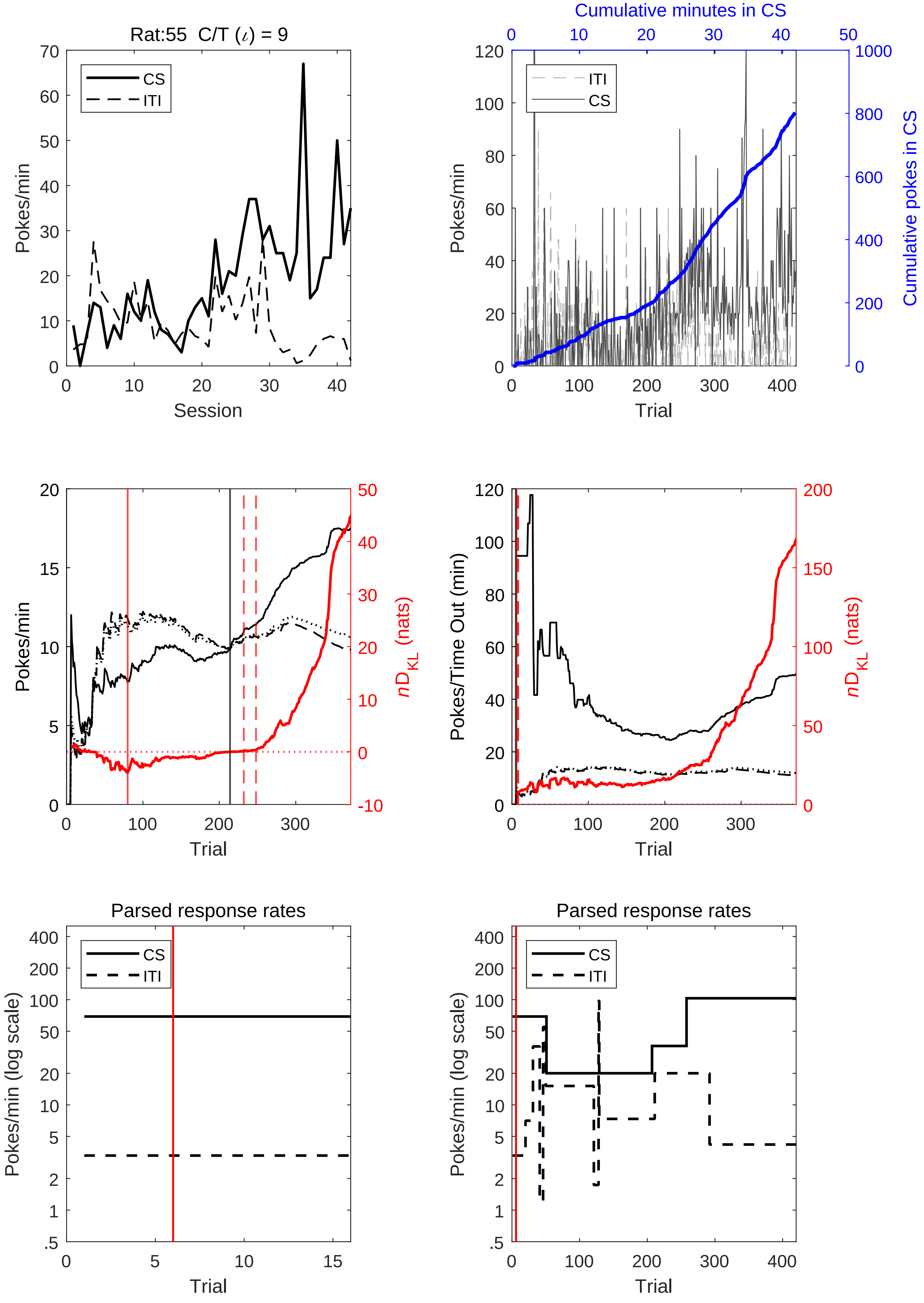
Rat:55 C/T (ι) = 9
CS
ITI
Pokes/min
Session
Cumulative minutes in CS
Cumulative pokes in CS
Trial
nD_KL (nats)
Pokes/Time Out (min)
Parsed response rates
Pokes/min (log scale)

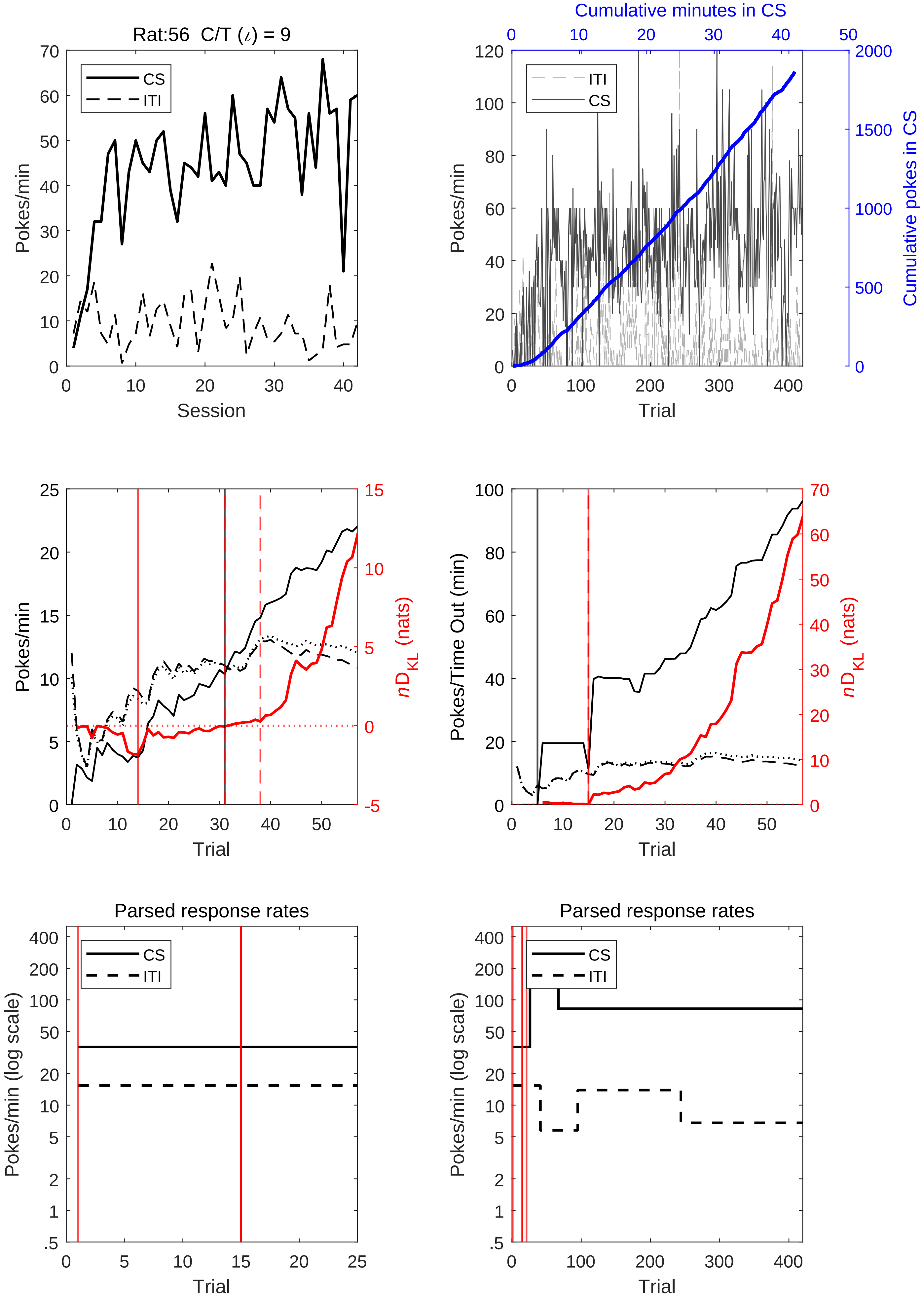
Rat:56 C/T (ι) = 9
CS
ITI
Pokes/min
Session
Cumulative minutes in CS
Cumulative pokes in CS
Trial
nD_KL (nats)
Pokes/Time Out (min)
Parsed response rates
Pokes/min (log scale)

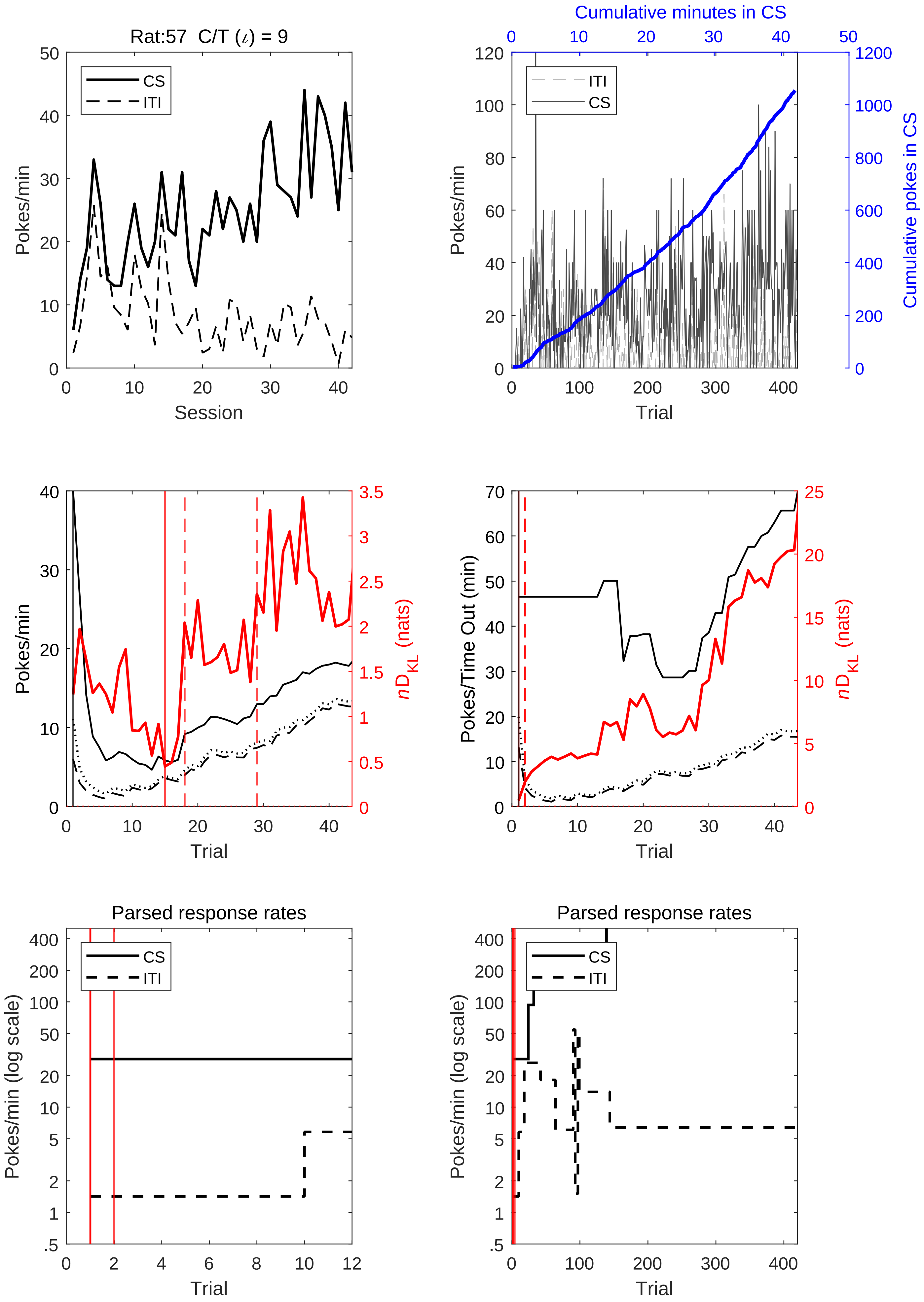
Rat:57 C/T (ι) = 9
CS
ITI
Pokes/min
Session
Cumulative minutes in CS
Cumulative pokes in CS
Trial
nD_KL (nats)
Pokes/Time Out (min)
Parsed response rates
Pokes/min (log scale)

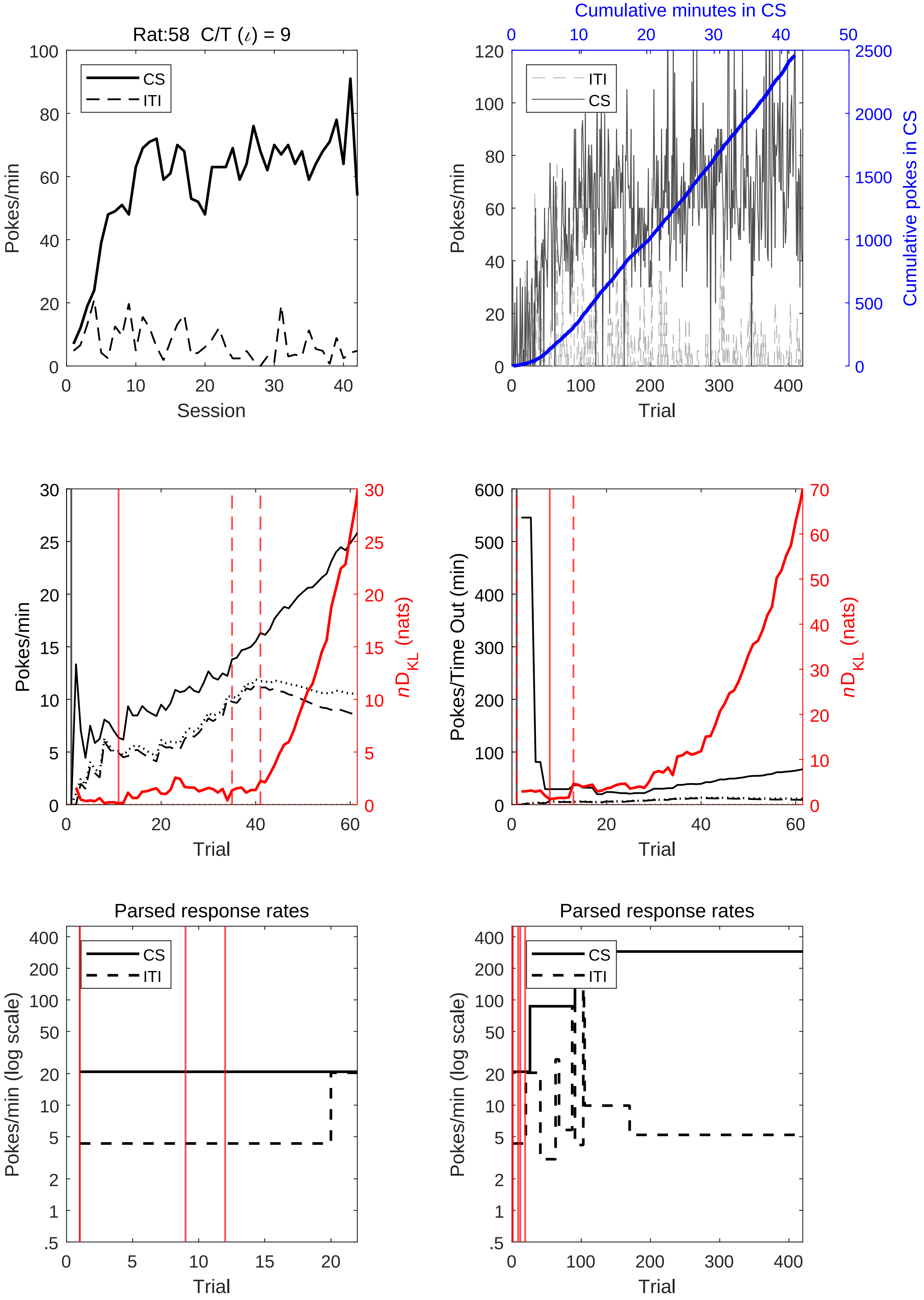

Rat:58 C/T ($\iota$) = 9
CS
ITI
Pokes/min
Session
Cumulative minutes in CS
Cumulative pokes in CS
Trial
Pokes/Time Out (min)
$nD_{KL}$ (nats)
Parsed response rates
Pokes/min (log scale)

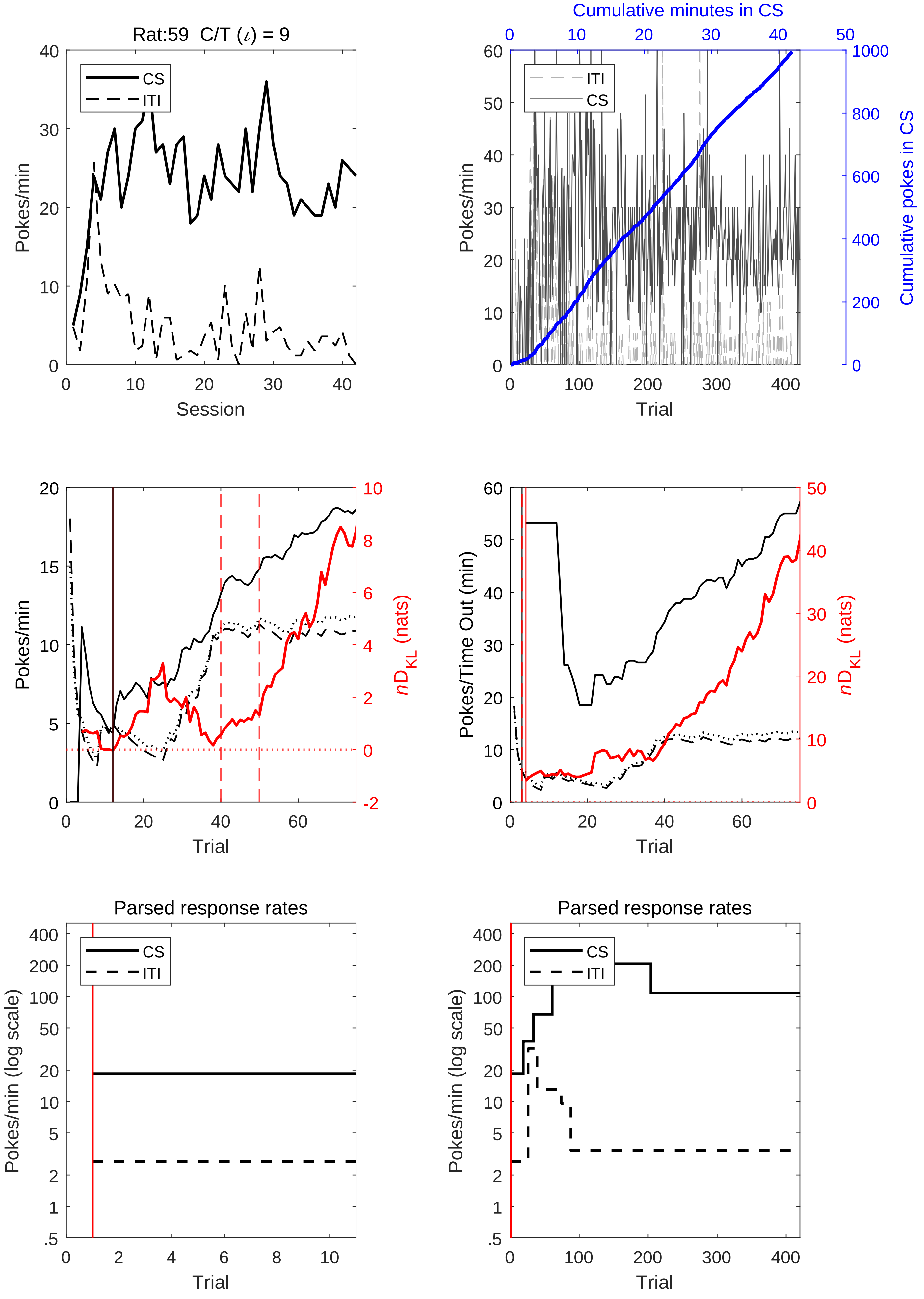

Rat:59 C/T (ι) = 9
CS
ITI
Pokes/min
Session
Cumulative minutes in CS
Cumulative pokes in CS
Trial
nD_KL (nats)
Pokes/Time Out (min)
Parsed response rates
Pokes/min (log scale)

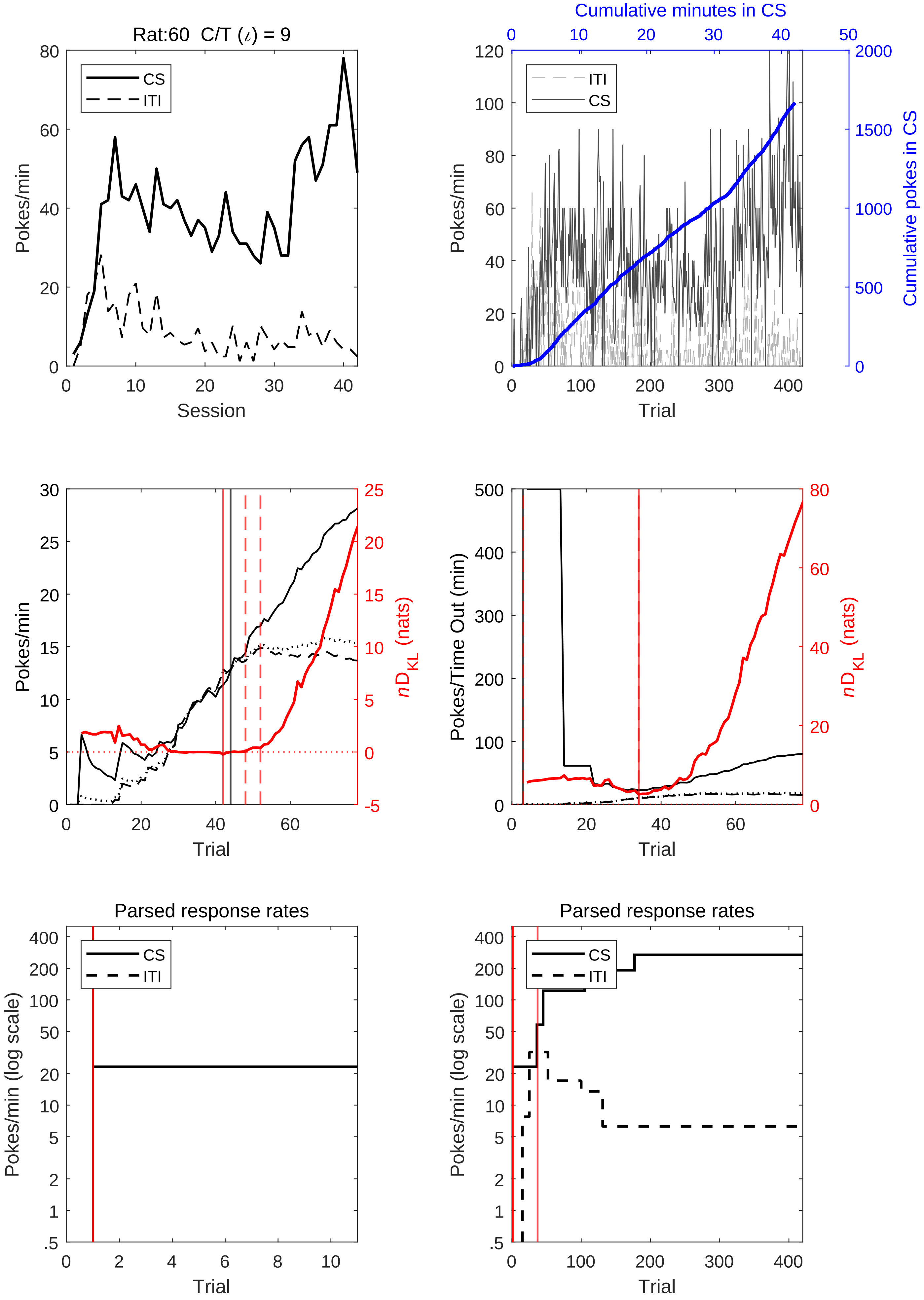

Rat:60 C/T (ι) = 9
Pokes/min
Session
CS
ITI
Cumulative minutes in CS
Cumulative pokes in CS
Trial
nD_KL (nats)
Pokes/Time Out (min)
Parsed response rates
Pokes/min (log scale)

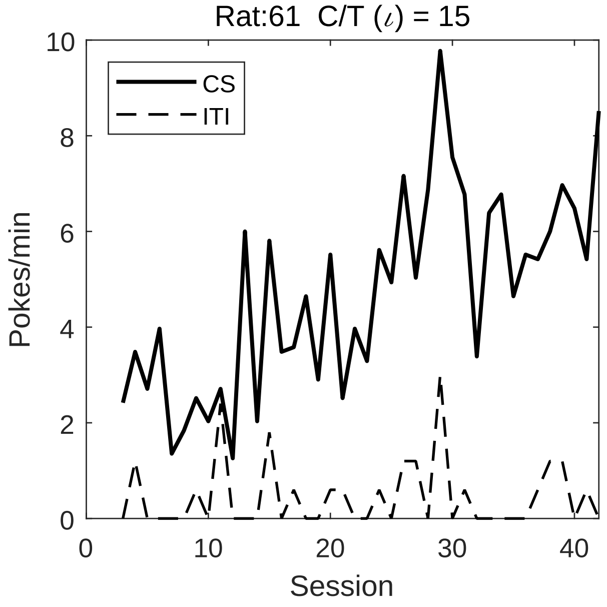

Rat:61 C/T (ι) = 15
CS
ITI
Pokes/min
Session
0
2
4
6
8
10
0
10
20
30
40


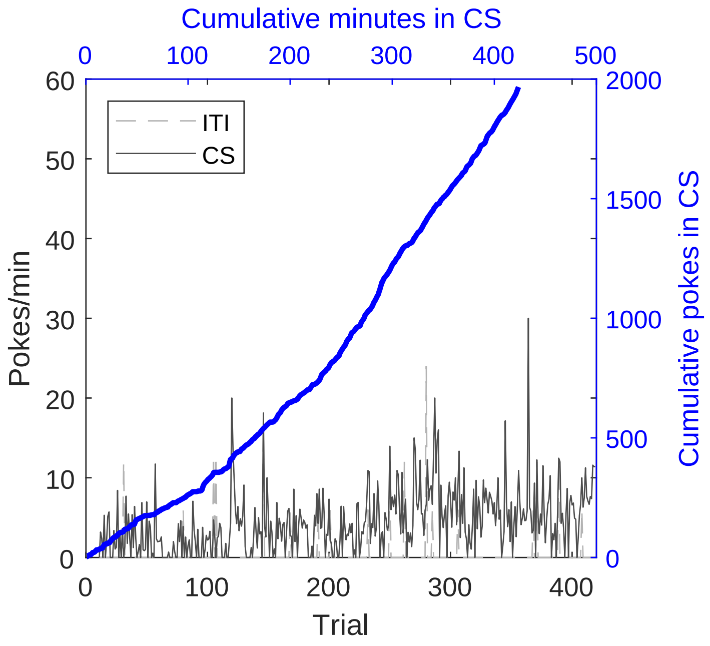

Cumulative minutes in CS
0
100
200
300
400
500
ITI
CS
Pokes/min
Cumulative pokes in CS
Trial
0
10
20
30
40
50
60
0
500
1000
1500
2000
0
100
200
300
400

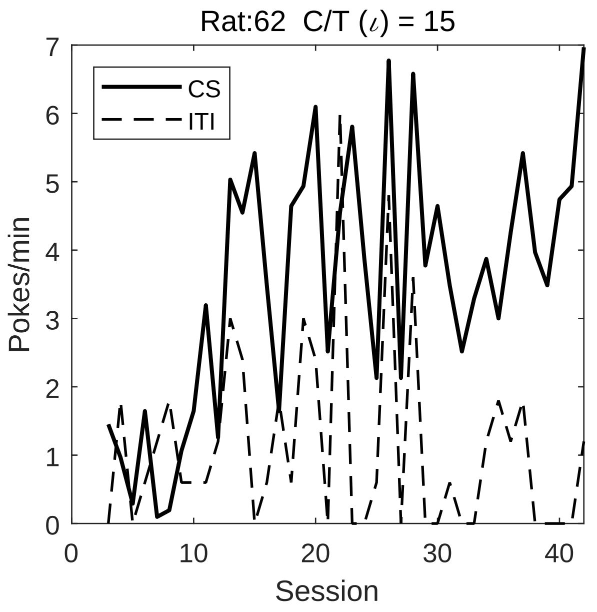

Rat:62 C/T (ι) = 15
CS
ITI
Pokes/min
Session


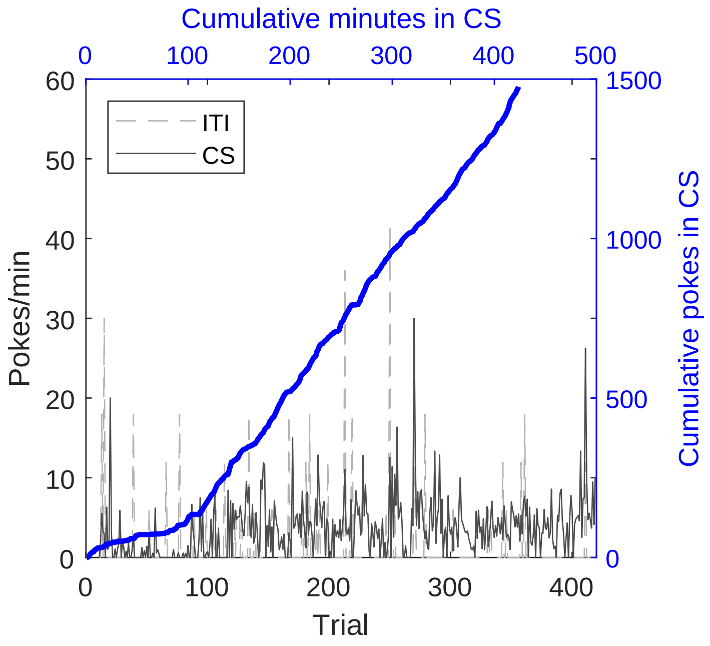

Cumulative minutes in CS
ITI
CS
Pokes/min
Cumulative pokes in CS
Trial

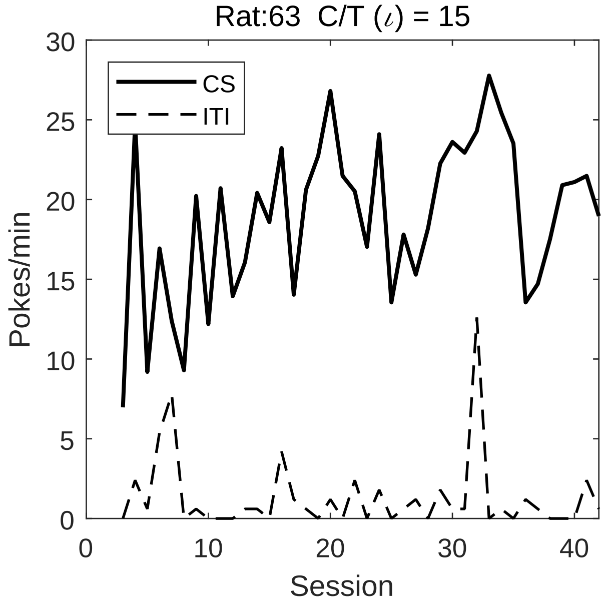
Rat:63 C/T (ι) = 15
CS
ITI
Pokes/min
Session
0
10
20
30
40
0
5
10
15
20
25
30

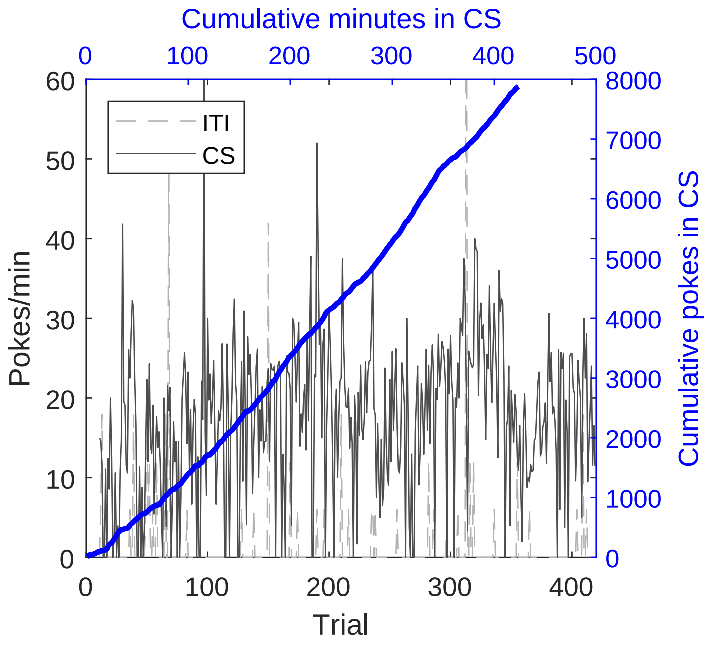
Cumulative minutes in CS
ITI
CS
Pokes/min
Trial
Cumulative pokes in CS
0
100
200
300
400
500
0
10
20
30
40
50
60
1000
2000
3000
4000
5000
6000
7000
8000

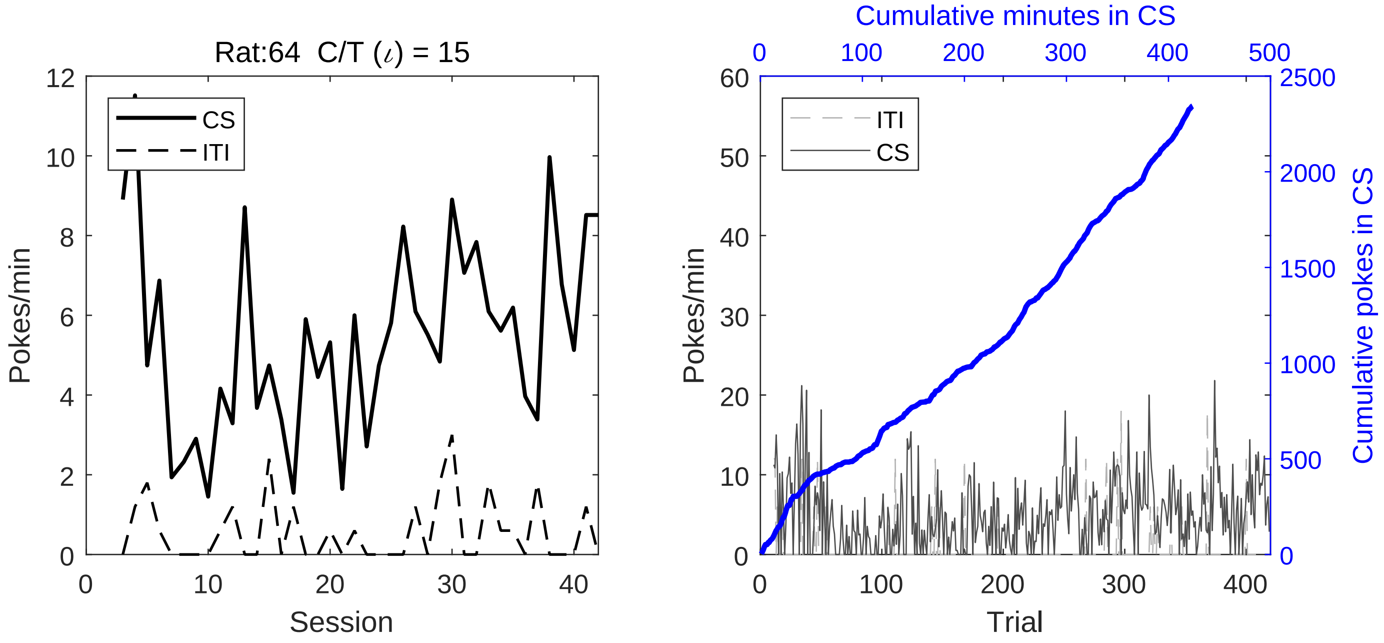
Rat:64 C/T (ι) = 15
CS
ITI
Pokes/min
Session
Cumulative minutes in CS
ITI
CS
Pokes/min
Trial
Cumulative pokes in CS

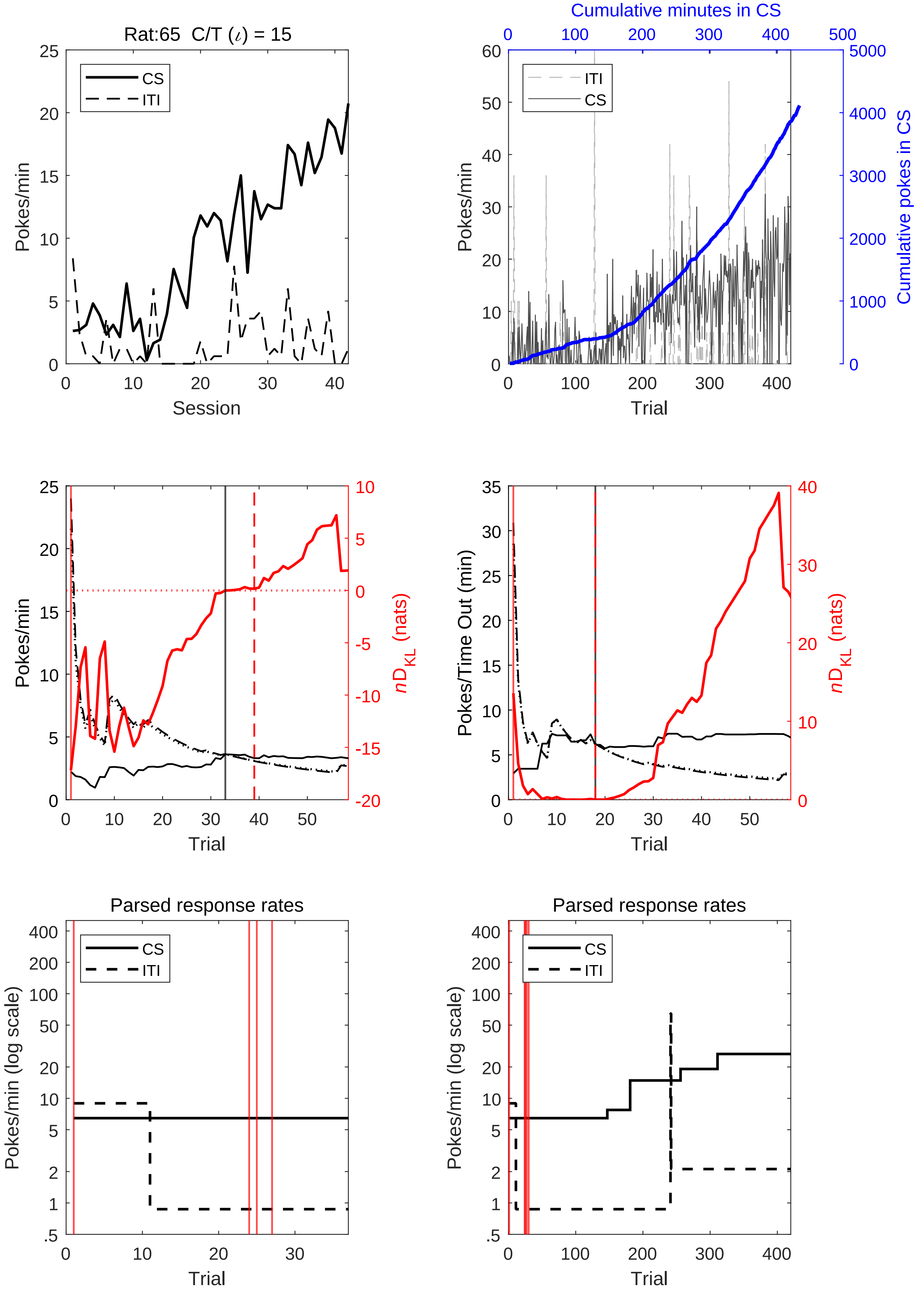

Rat:65 C/T (ι) = 15
CS
ITI
Pokes/min
Session
Cumulative minutes in CS
Cumulative pokes in CS
Trial
nD_KL (nats)
Pokes/Time Out (min)
Parsed response rates
Pokes/min (log scale)

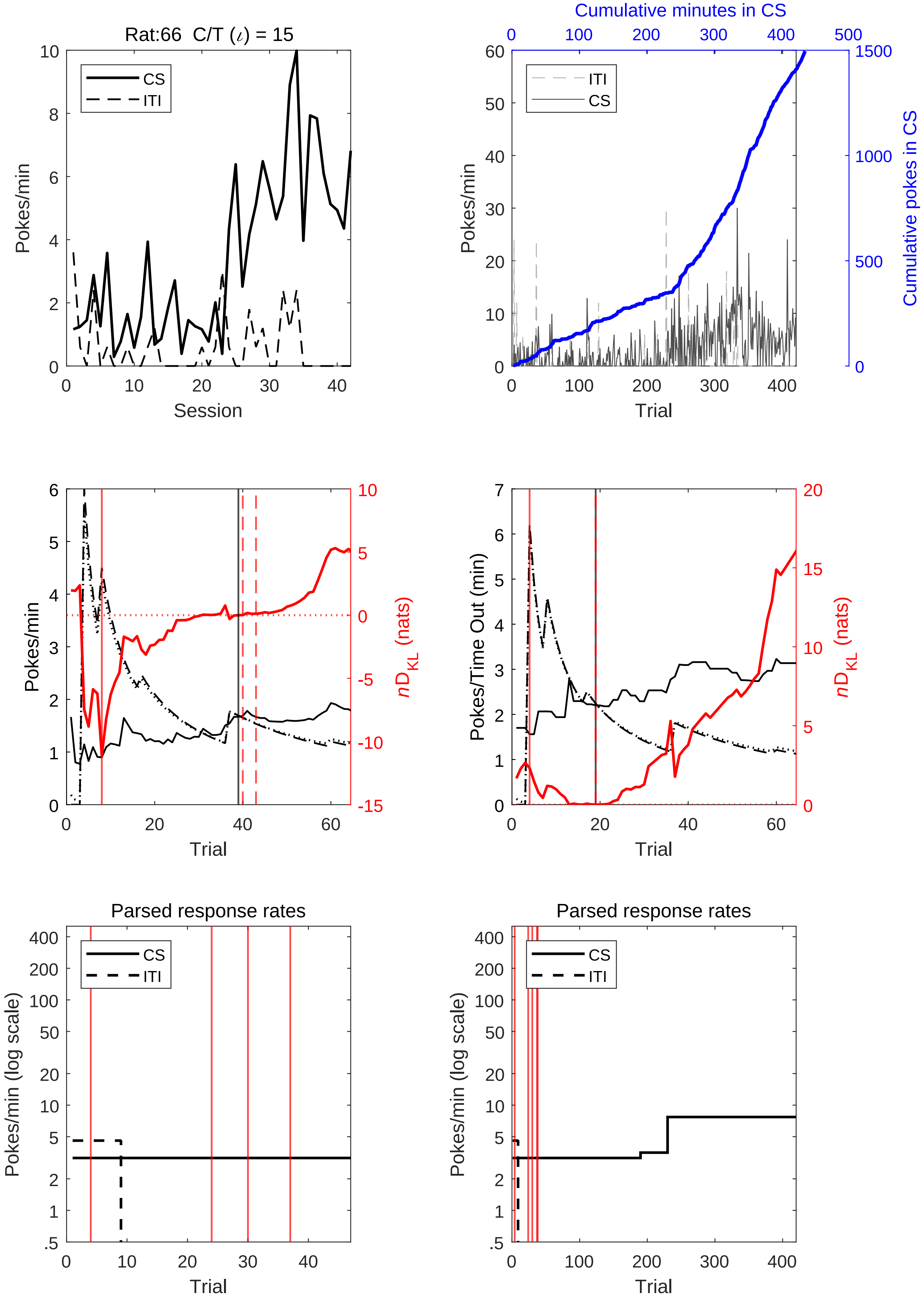
Rat:66 C/T (ι) = 15
CS
ITI
Pokes/min
Session
Cumulative minutes in CS
Cumulative pokes in CS
Trial
Pokes/Time Out (min)
nD_KL (nats)
Parsed response rates
Pokes/min (log scale)

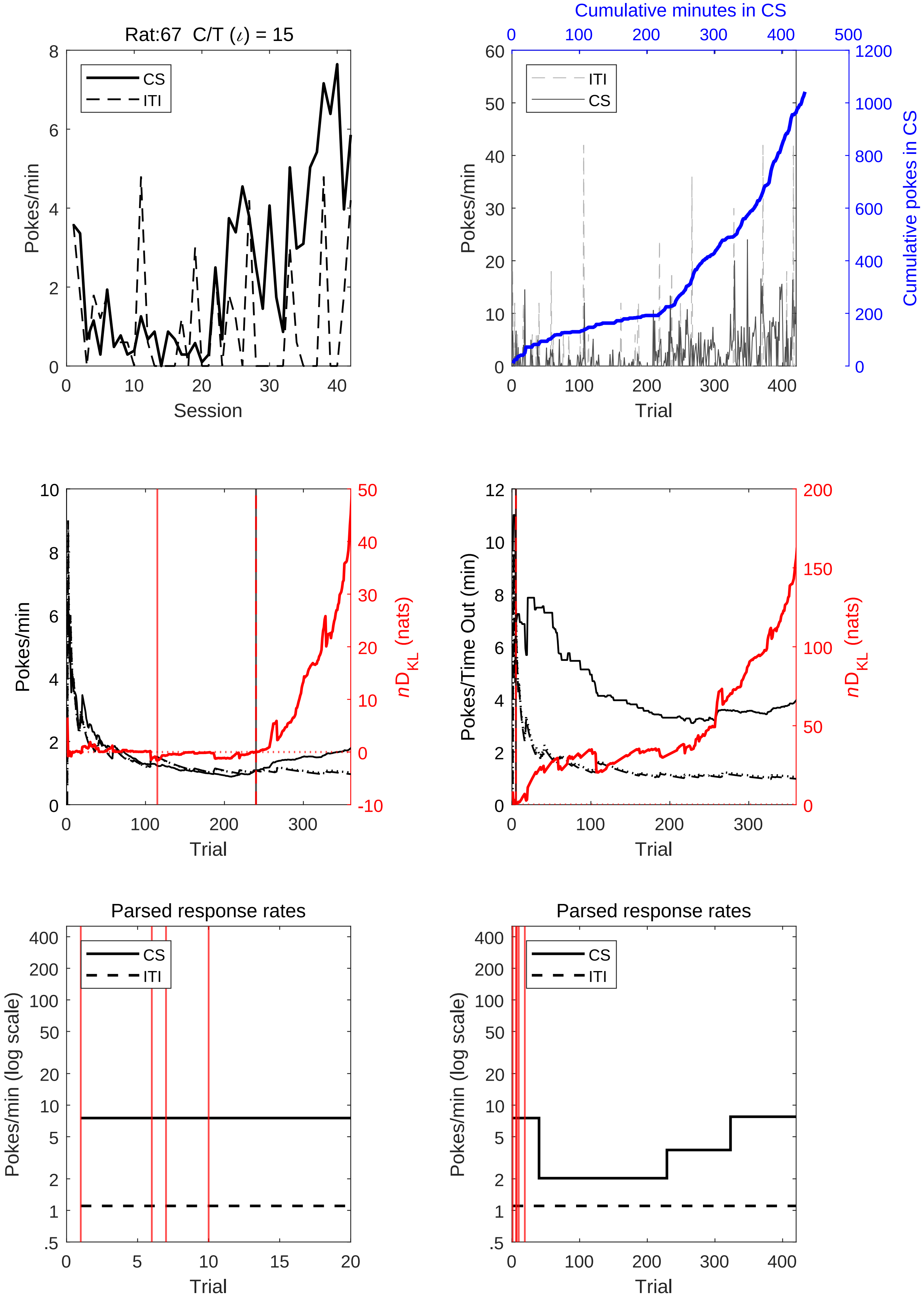

Rat:67 C/T (ι) = 15
CS
ITI
Pokes/min
Session
Cumulative minutes in CS
ITI
CS
Pokes/min
Cumulative pokes in CS
Trial
Pokes/min
$nD_{KL}$ (nats)
Trial
Pokes/Time Out (min)
$nD_{KL}$ (nats)
Trial
Parsed response rates
CS
ITI
Pokes/min (log scale)
Trial
Parsed response rates
CS
ITI
Pokes/min (log scale)
Trial

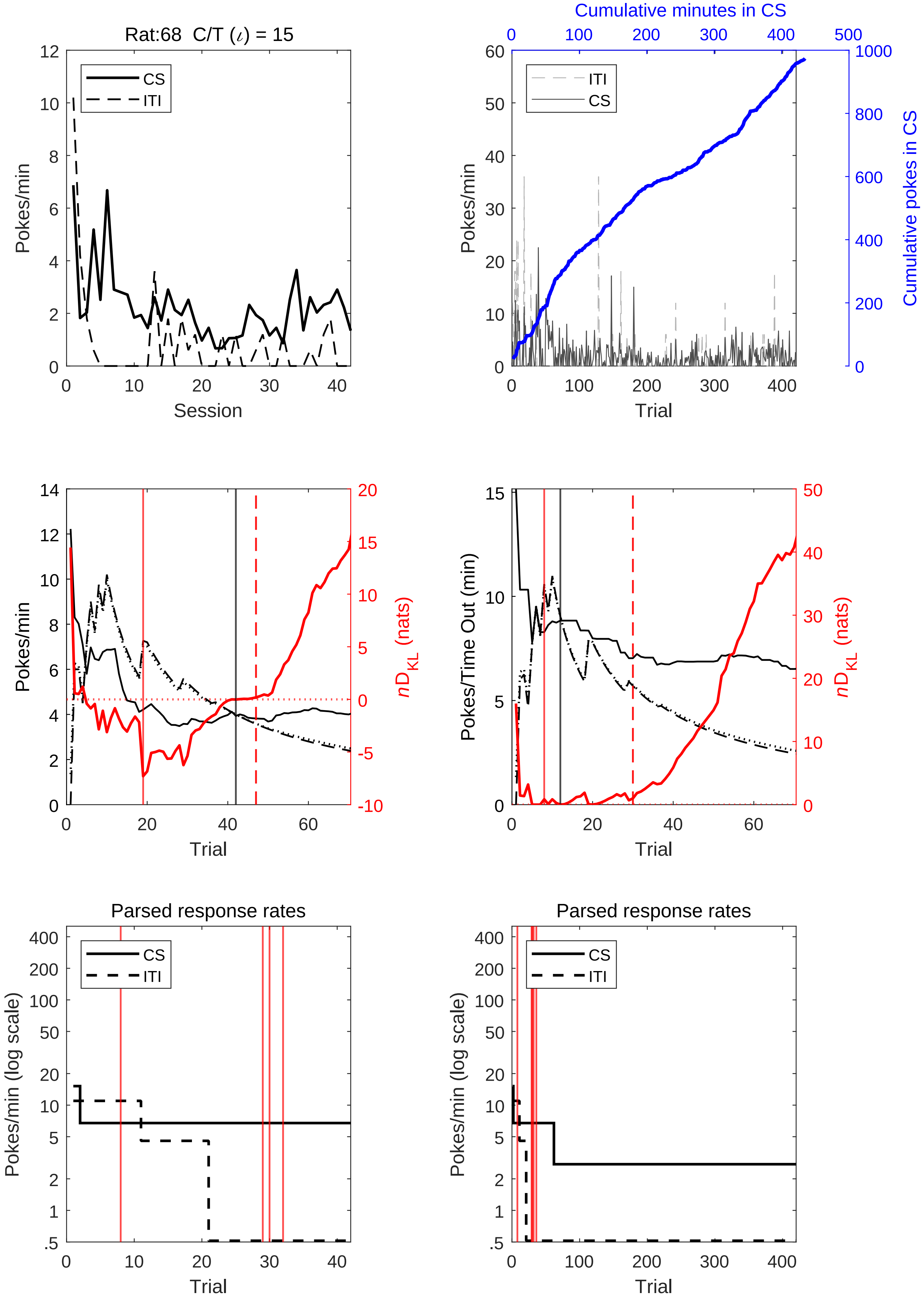
Rat:68 C/T (ι) = 15
Pokes/min
Session
CS
ITI
Cumulative minutes in CS
Cumulative pokes in CS
Trial
Pokes/min
Trial
nD_KL (nats)
Pokes/Time Out (min)
Parsed response rates
Pokes/min (log scale)

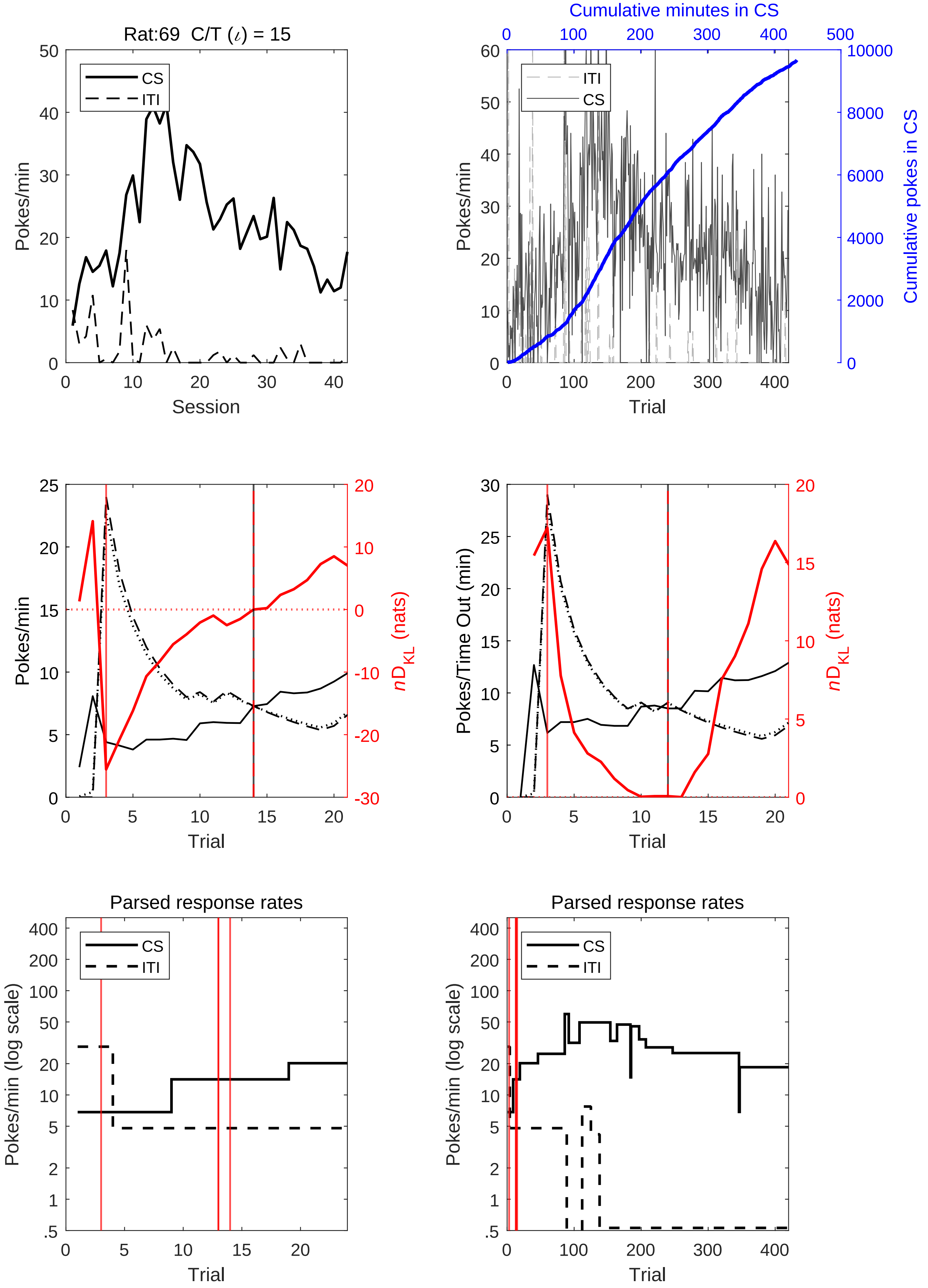

Rat:69 C/T (ι) = 15
CS
ITI
Pokes/min
Session
Cumulative minutes in CS
ITI
CS
Pokes/min
Cumulative pokes in CS
Trial
Pokes/min
nD_KL (nats)
Trial
Pokes/Time Out (min)
nD_KL (nats)
Trial
Parsed response rates
CS
ITI
Pokes/min (log scale)
Trial
Parsed response rates
CS
ITI
Pokes/min (log scale)
Trial

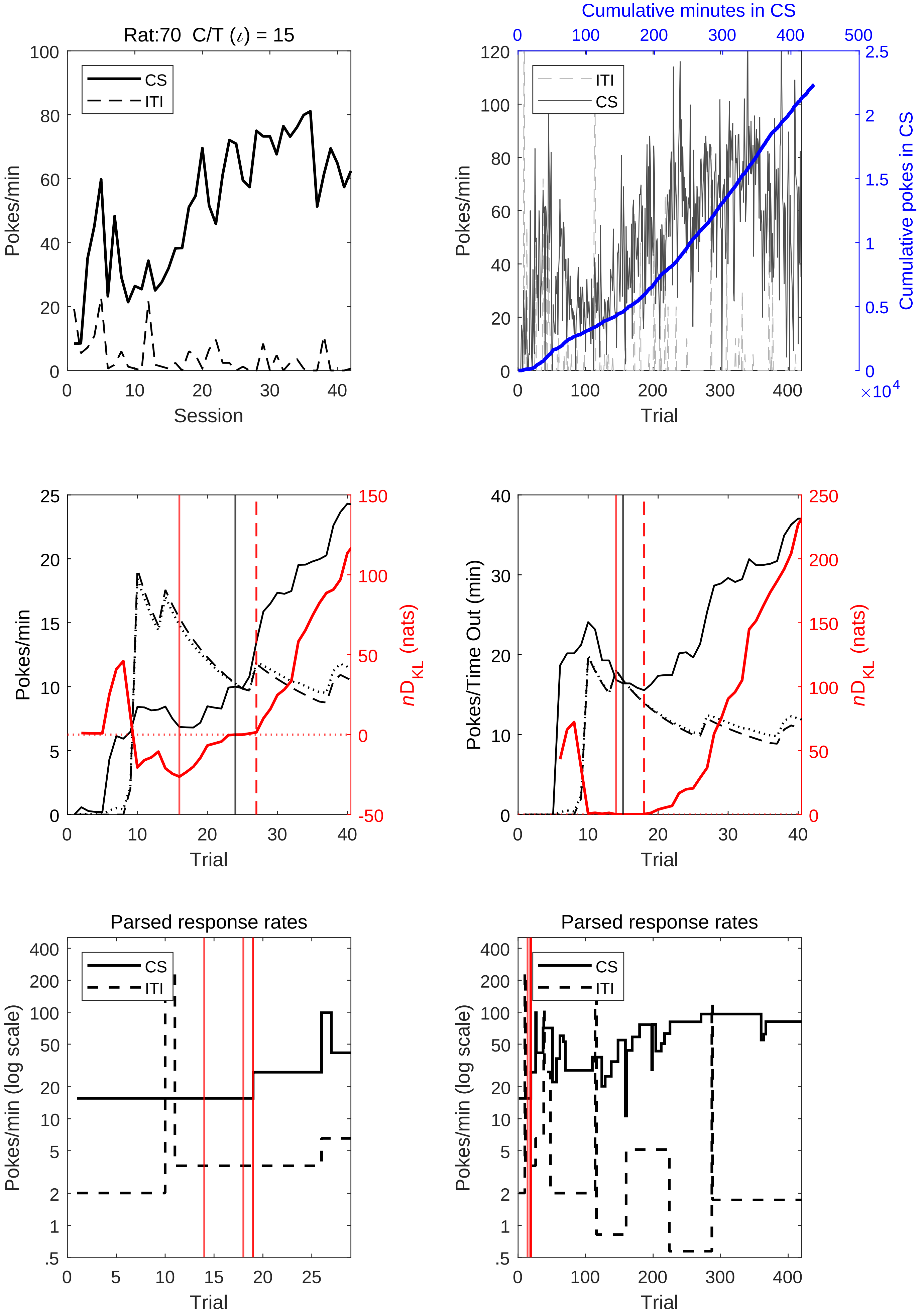
Rat:70 C/T (ι) = 15
CS
ITI
Pokes/min
Session
Cumulative minutes in CS
Cumulative pokes in CS
×10^4
Trial
Pokes/Time Out (min)
nD_KL (nats)
Parsed response rates
Pokes/min (log scale)

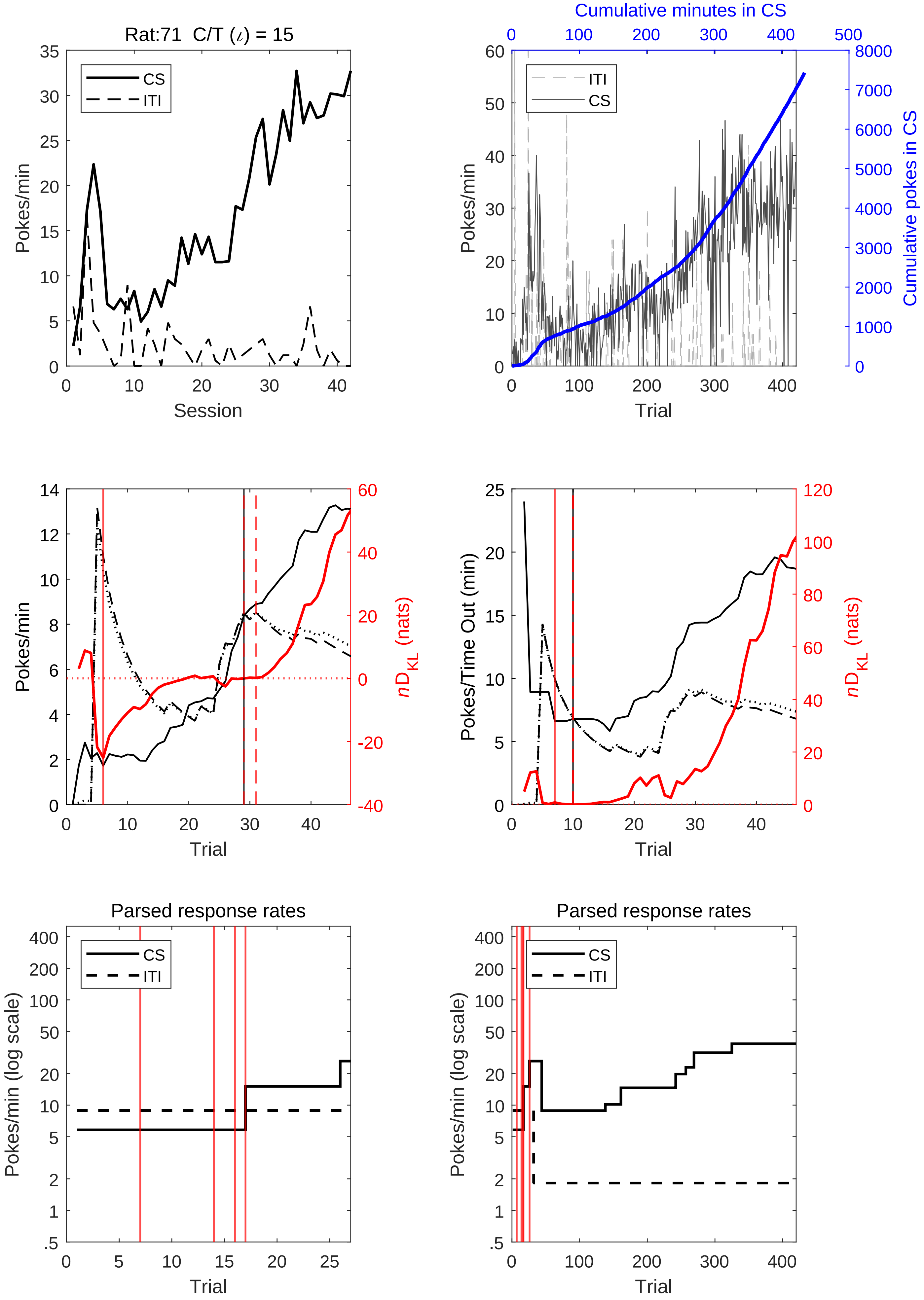
Rat:71 C/T (ι) = 15
Pokes/min
Session
CS
ITI
Cumulative minutes in CS
Cumulative pokes in CS
Trial
Pokes/Time Out (min)
nD_KL (nats)
Parsed response rates
Pokes/min (log scale)

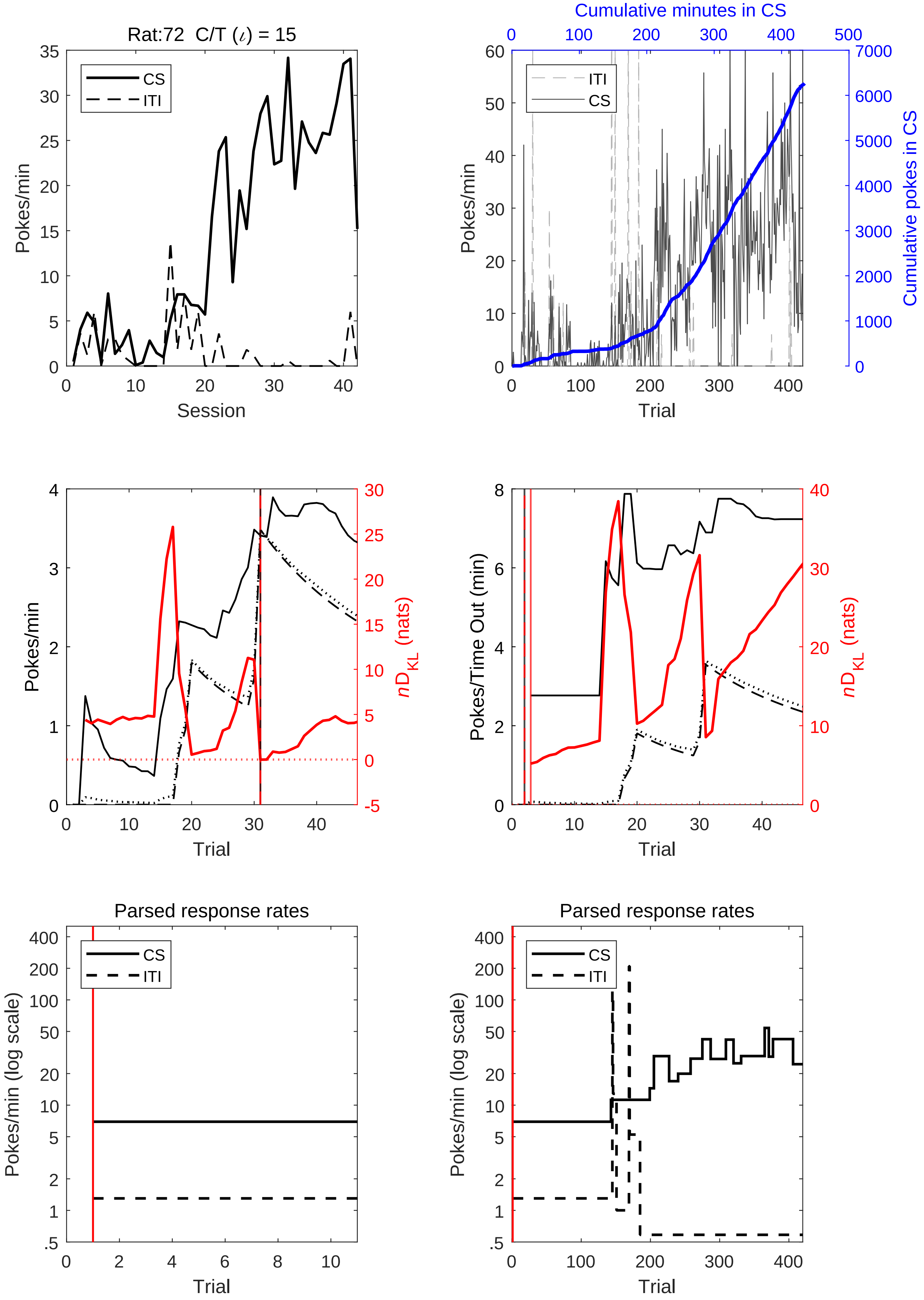
Rat:72 C/T (ι) = 15
CS
ITI
Pokes/min
Session
Cumulative minutes in CS
Cumulative pokes in CS
Trial
nD_KL (nats)
Pokes/Time Out (min)
Parsed response rates
Pokes/min (log scale)

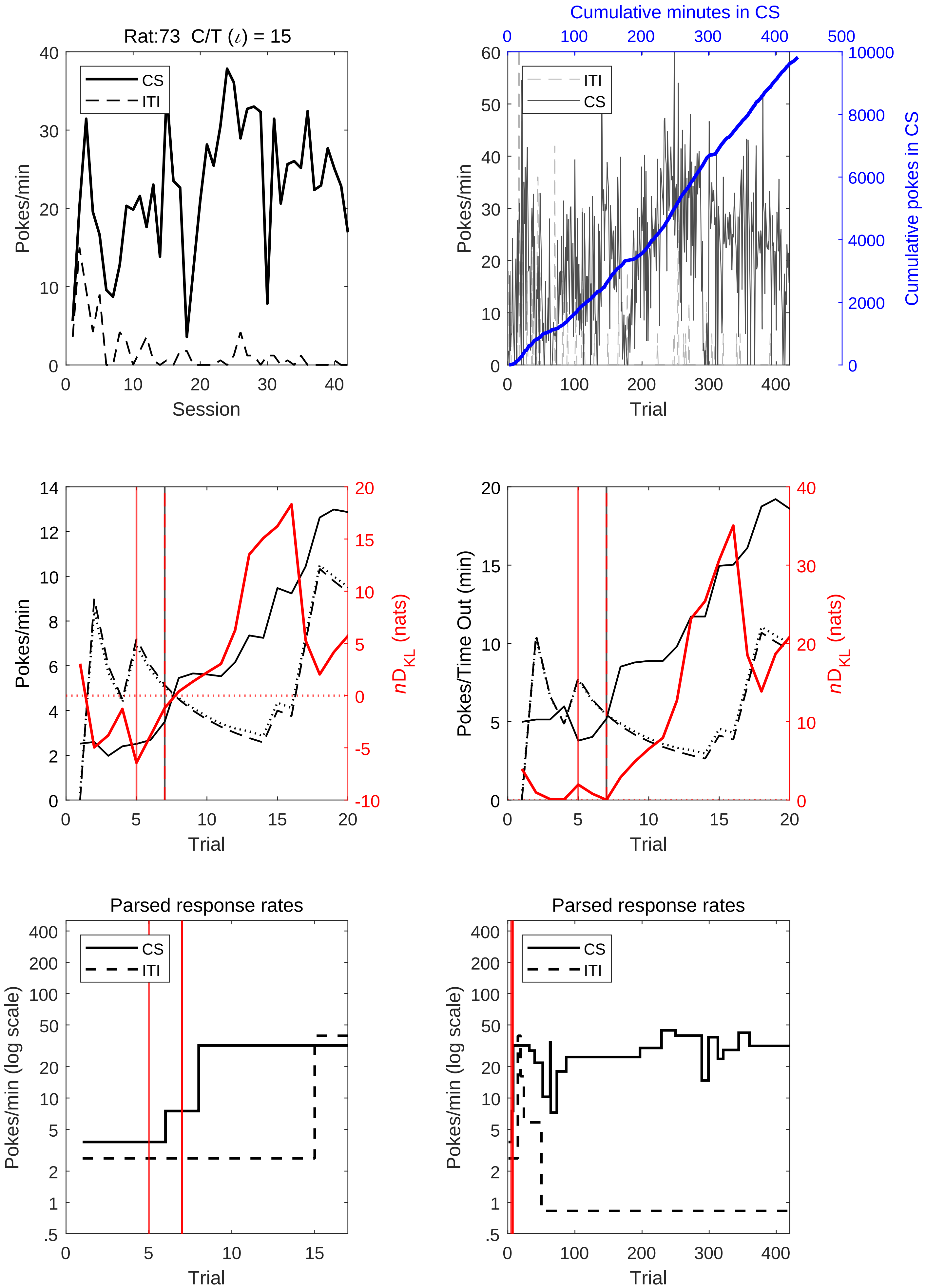

Rat:73 C/T (ι) = 15
CS
ITI
Pokes/min
Session
Cumulative minutes in CS
Cumulative pokes in CS
Trial
nD_KL (nats)
Pokes/Time Out (min)
Parsed response rates
Pokes/min (log scale)

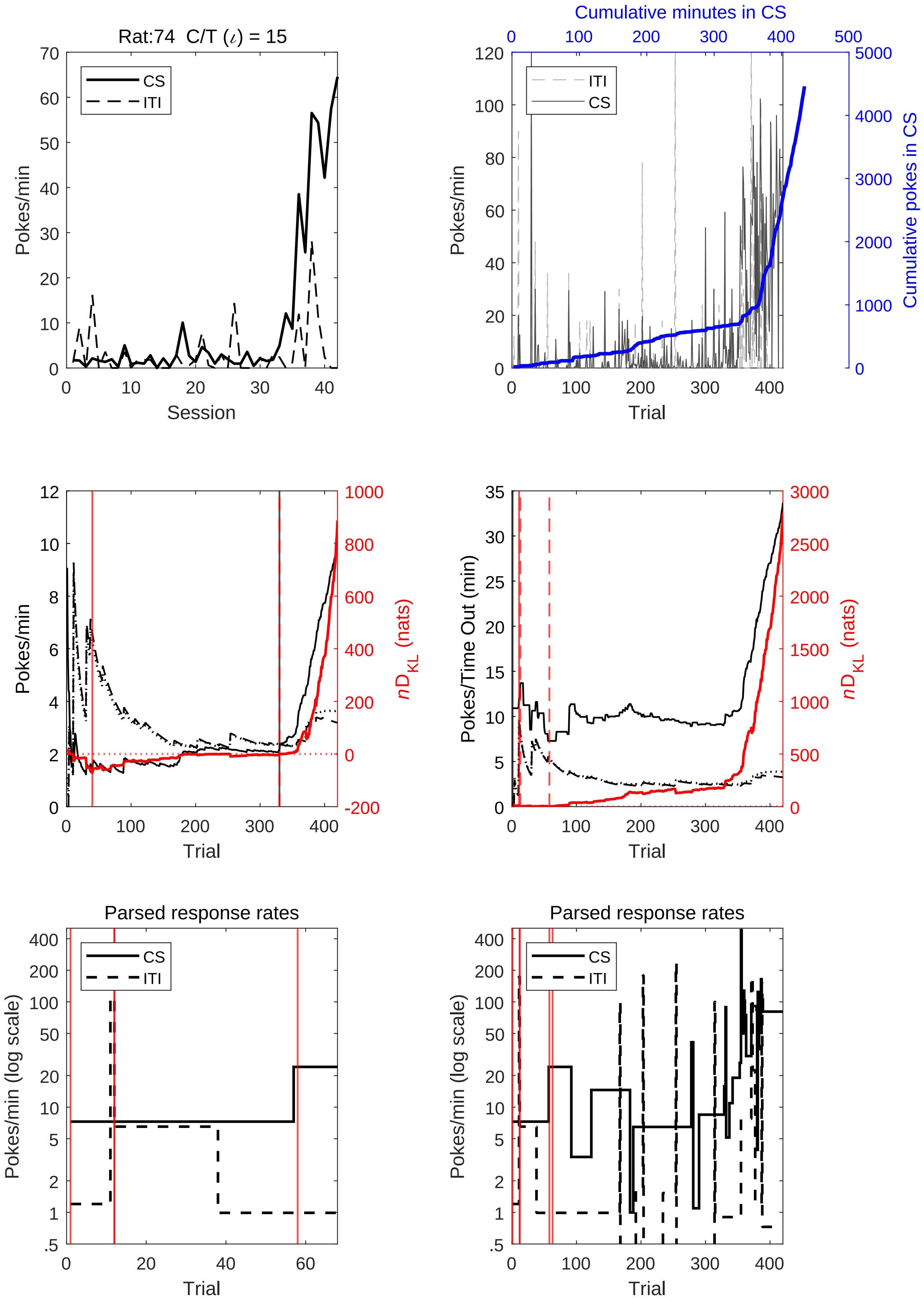
Rat:74 C/T (ι) = 15
CS
ITI
Pokes/min
Session
Cumulative minutes in CS
Cumulative pokes in CS
Trial
nD_KL (nats)
Pokes/Time Out (min)
Parsed response rates
Pokes/min (log scale)

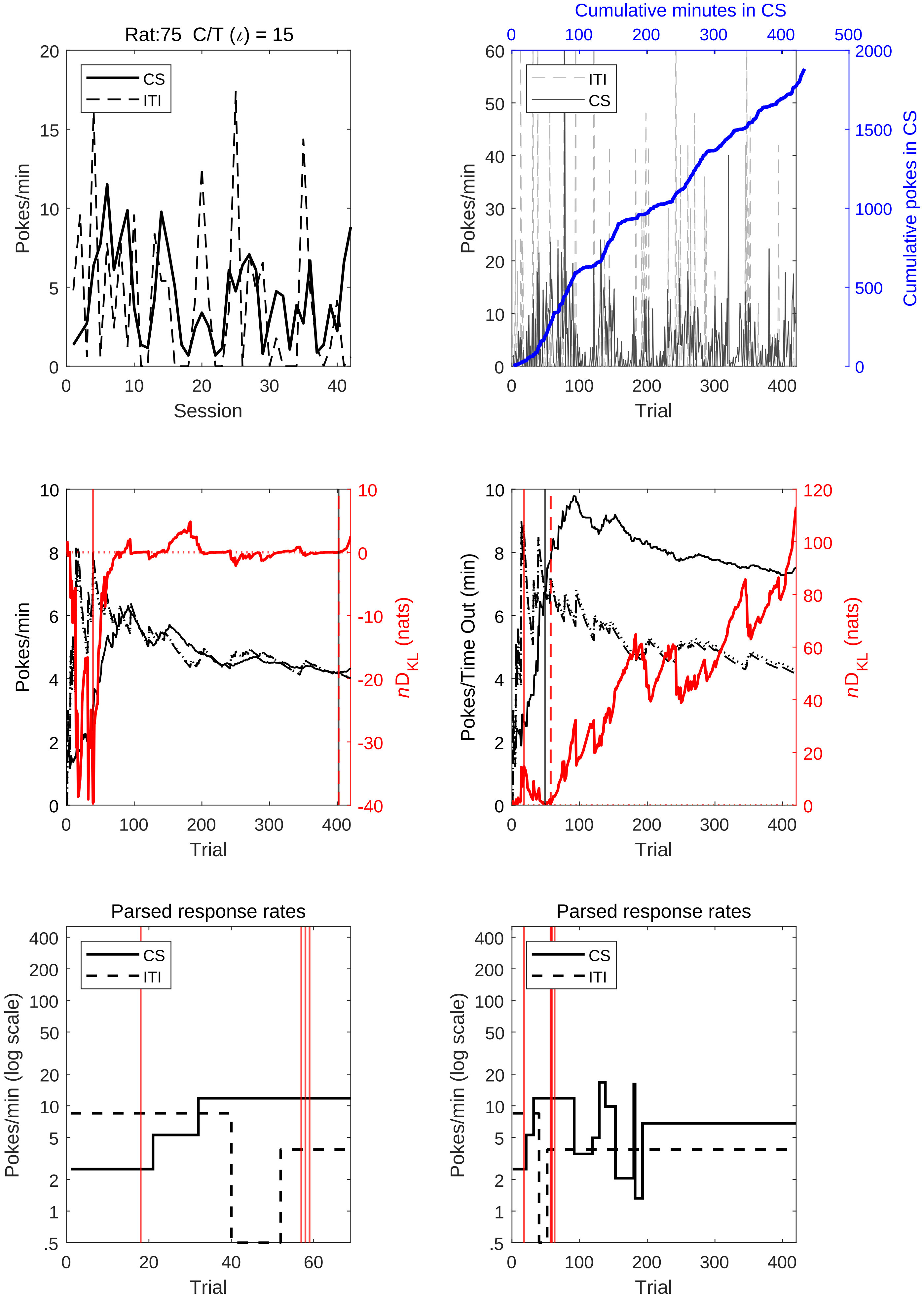

Rat:75 C/T (ι) = 15
CS
ITI
Pokes/min
Session
Cumulative minutes in CS
Cumulative pokes in CS
Trial
nDKL (nats)
Pokes/Time Out (min)
Parsed response rates
Pokes/min (log scale)

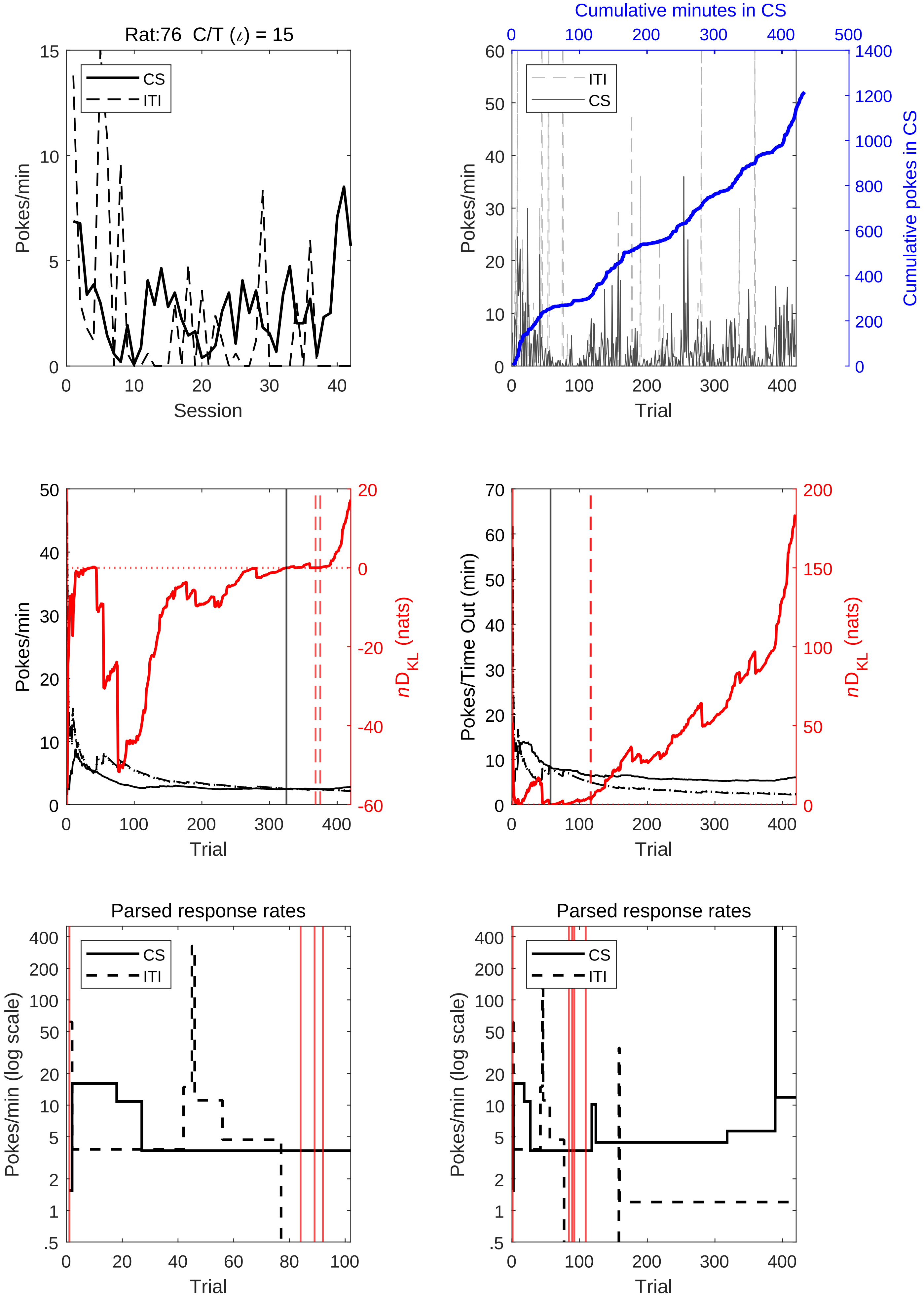
Rat:76 C/T (ι) = 15
CS
ITI
Pokes/min
Session
Cumulative minutes in CS
Cumulative pokes in CS
Trial
nD_KL (nats)
Pokes/Time Out (min)
Parsed response rates
Pokes/min (log scale)

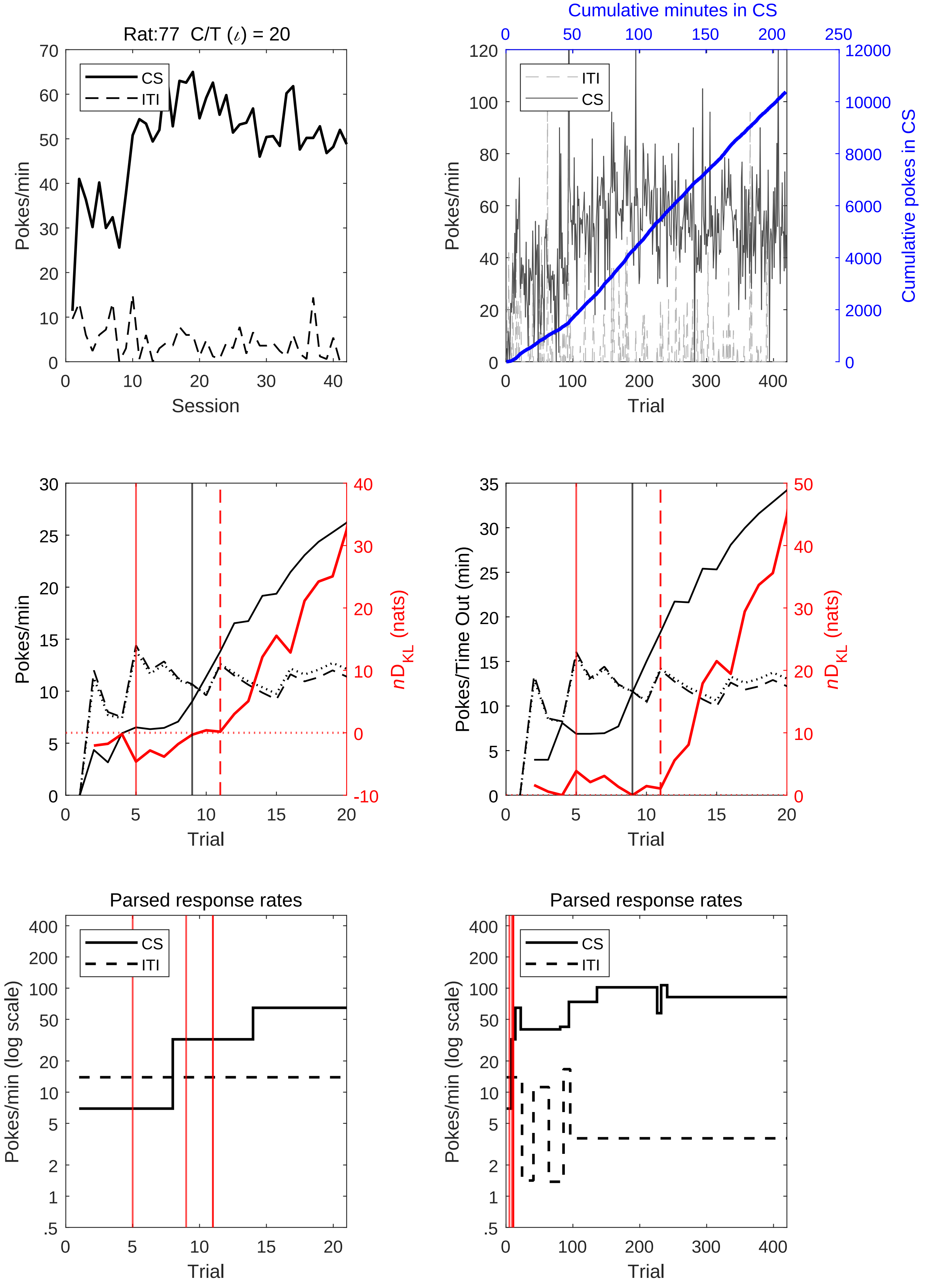
Rat:77 C/T (ι) = 20
Pokes/min
Session
CS
ITI
Cumulative minutes in CS
Cumulative pokes in CS
Trial
nD_KL (nats)
Pokes/Time Out (min)
Parsed response rates
Pokes/min (log scale)

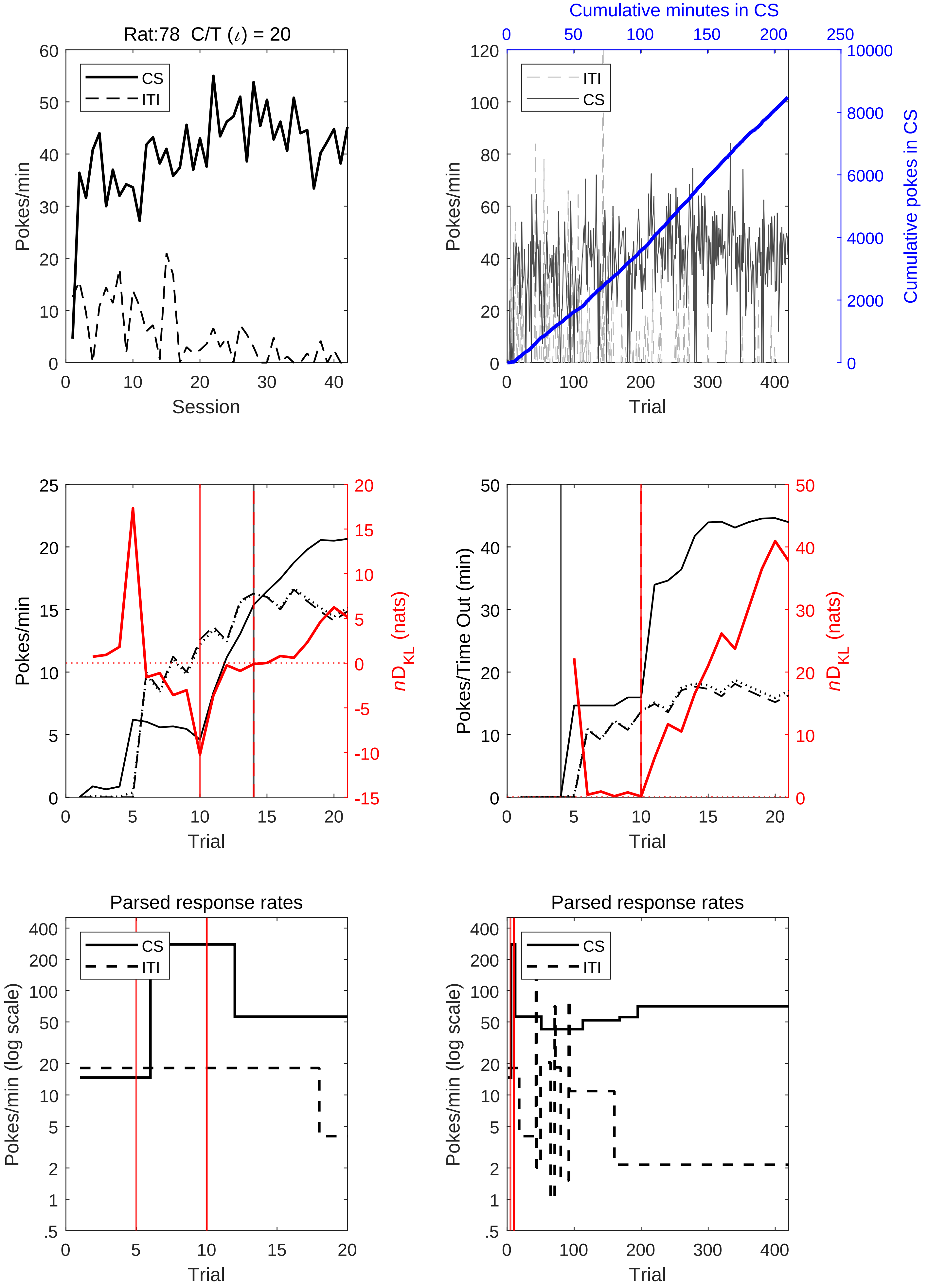
Rat:78 C/T ($\iota$) = 20
CS
ITI
Pokes/min
Session
Cumulative minutes in CS
Cumulative pokes in CS
Trial
$nD_{KL}$ (nats)
Pokes/Time Out (min)
Parsed response rates
Pokes/min (log scale)

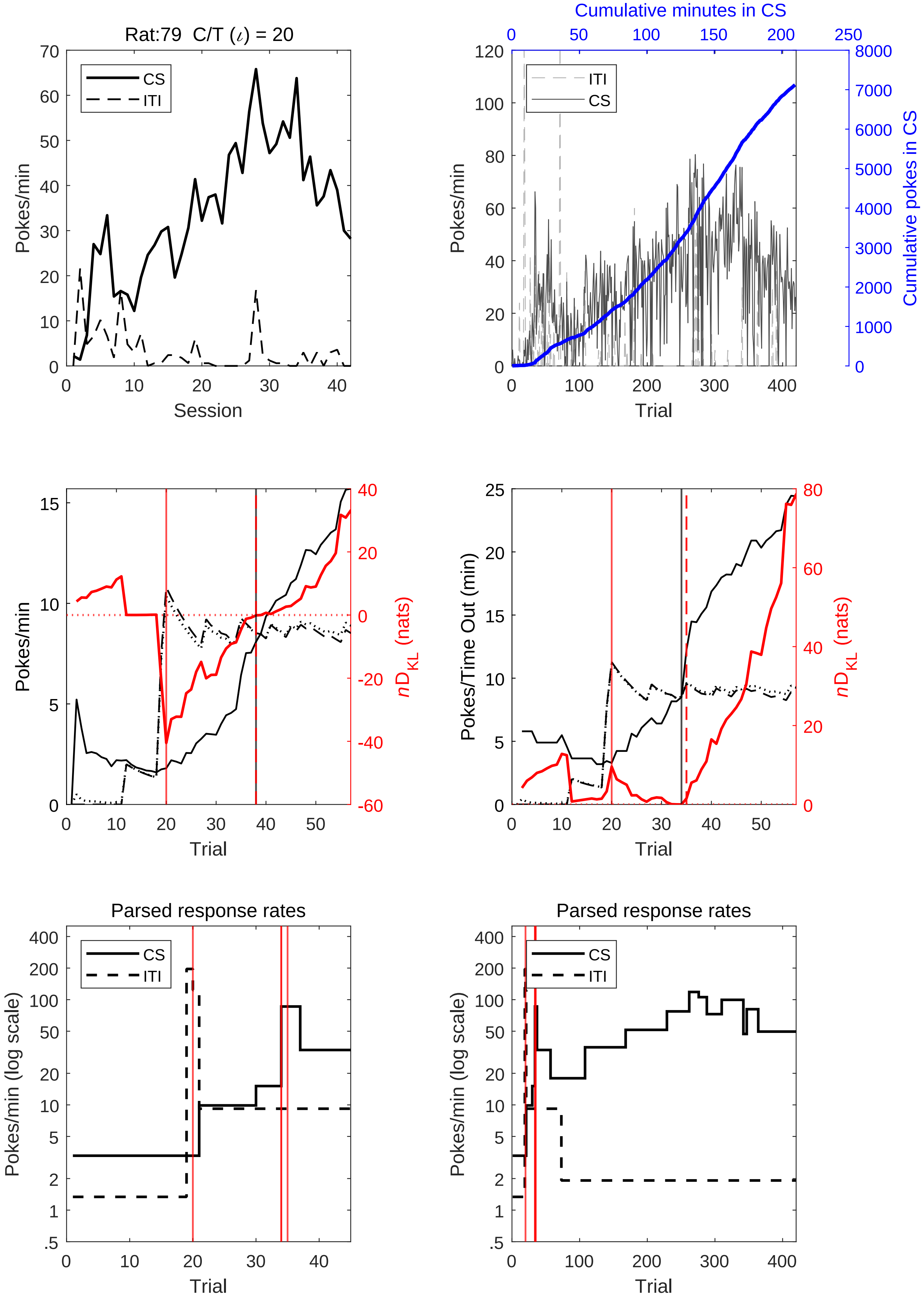

Rat:79 C/T (ι) = 20
CS
ITI
Pokes/min
Session
Cumulative minutes in CS
Cumulative pokes in CS
Trial
nD_KL (nats)
Pokes/Time Out (min)
Parsed response rates
Pokes/min (log scale)

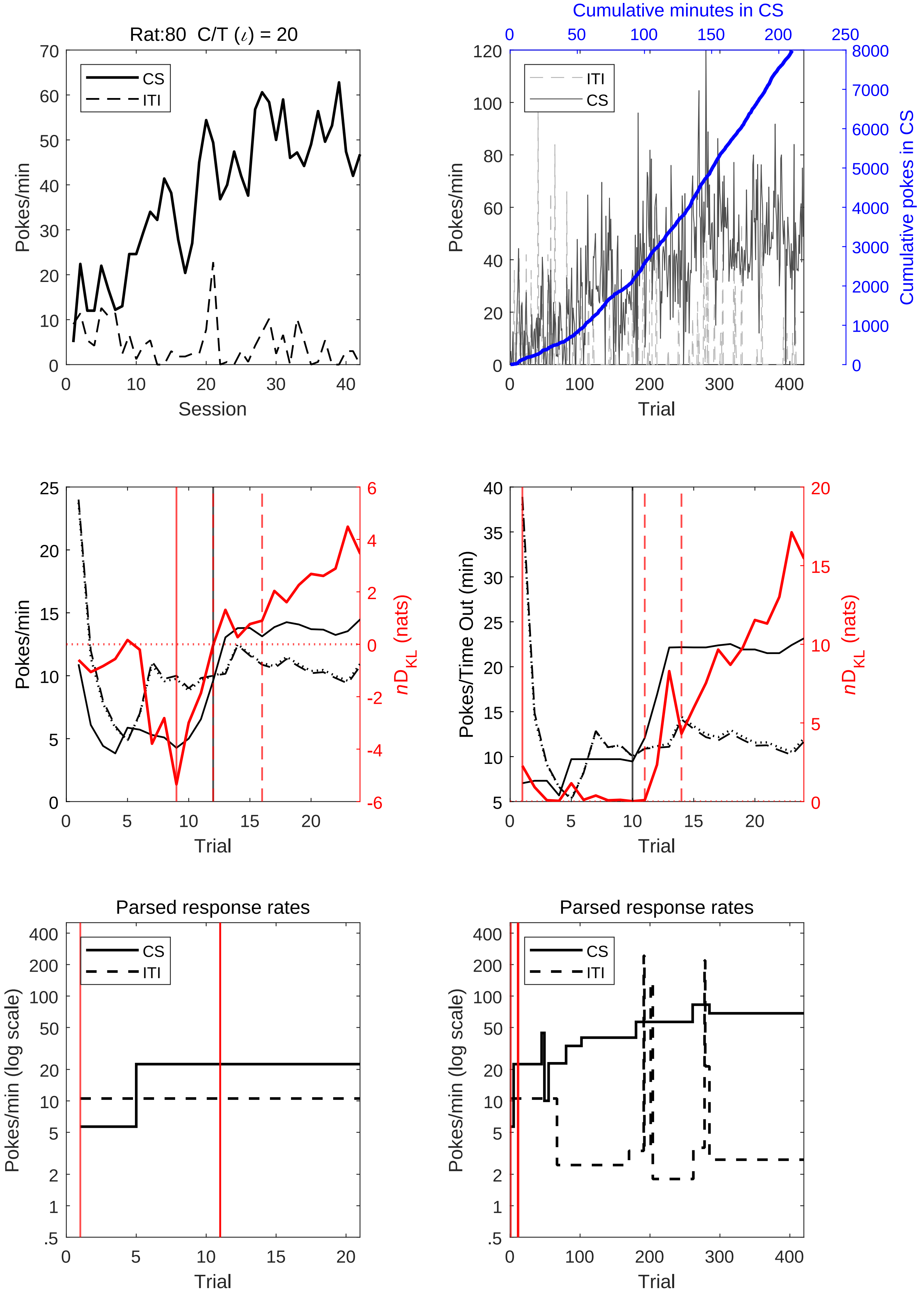

Rat:80 C/T (ι) = 20
CS
ITI
Pokes/min
Session
Cumulative minutes in CS
Cumulative pokes in CS
Trial
nD_KL (nats)
Pokes/Time Out (min)
Parsed response rates
Pokes/min (log scale)

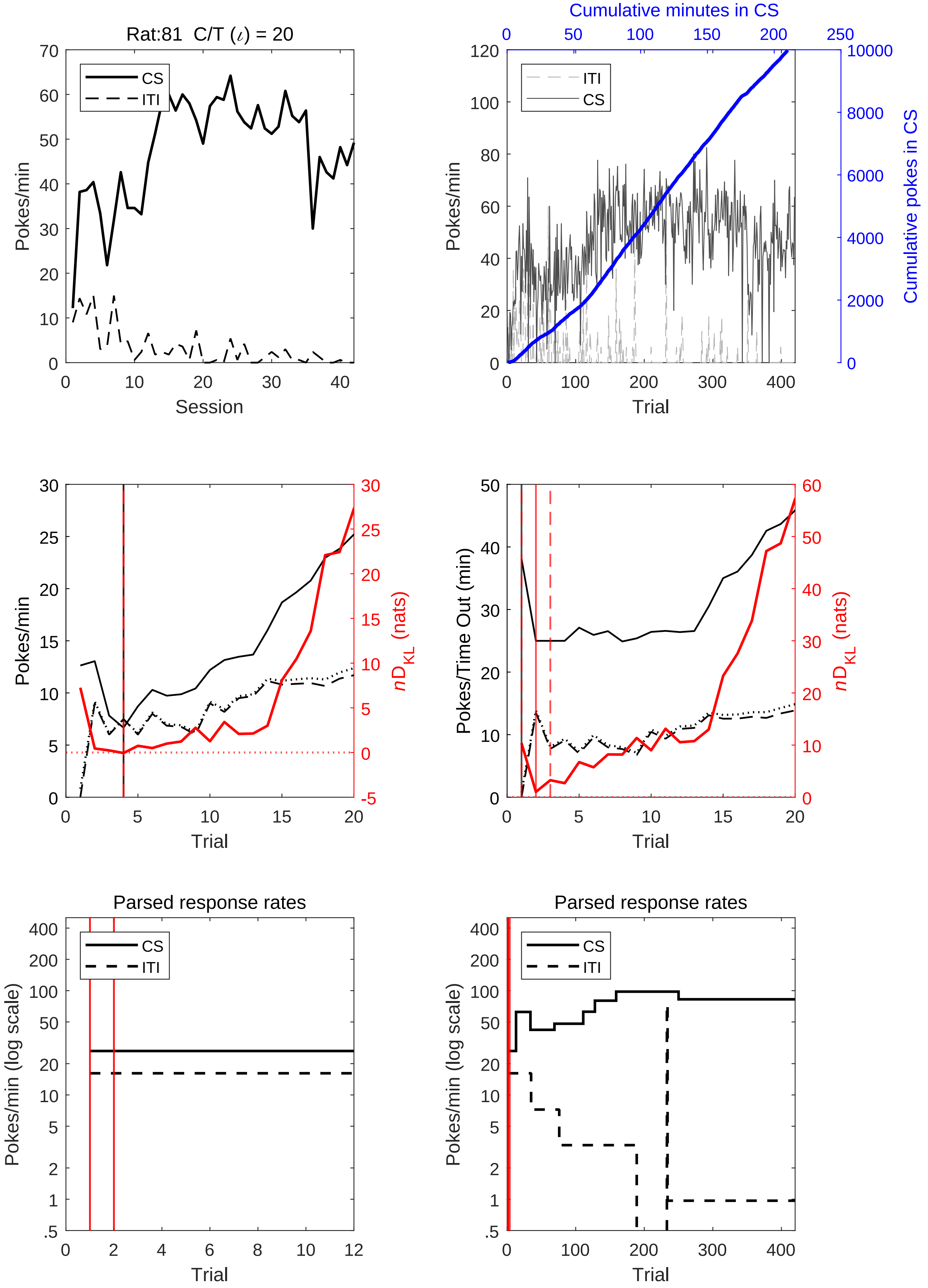
Rat:81 C/T (ι) = 20
Pokes/min
Session
CS
ITI
Cumulative minutes in CS
Cumulative pokes in CS
Trial
nD_KL (nats)
Pokes/Time Out (min)
Parsed response rates
Pokes/min (log scale)

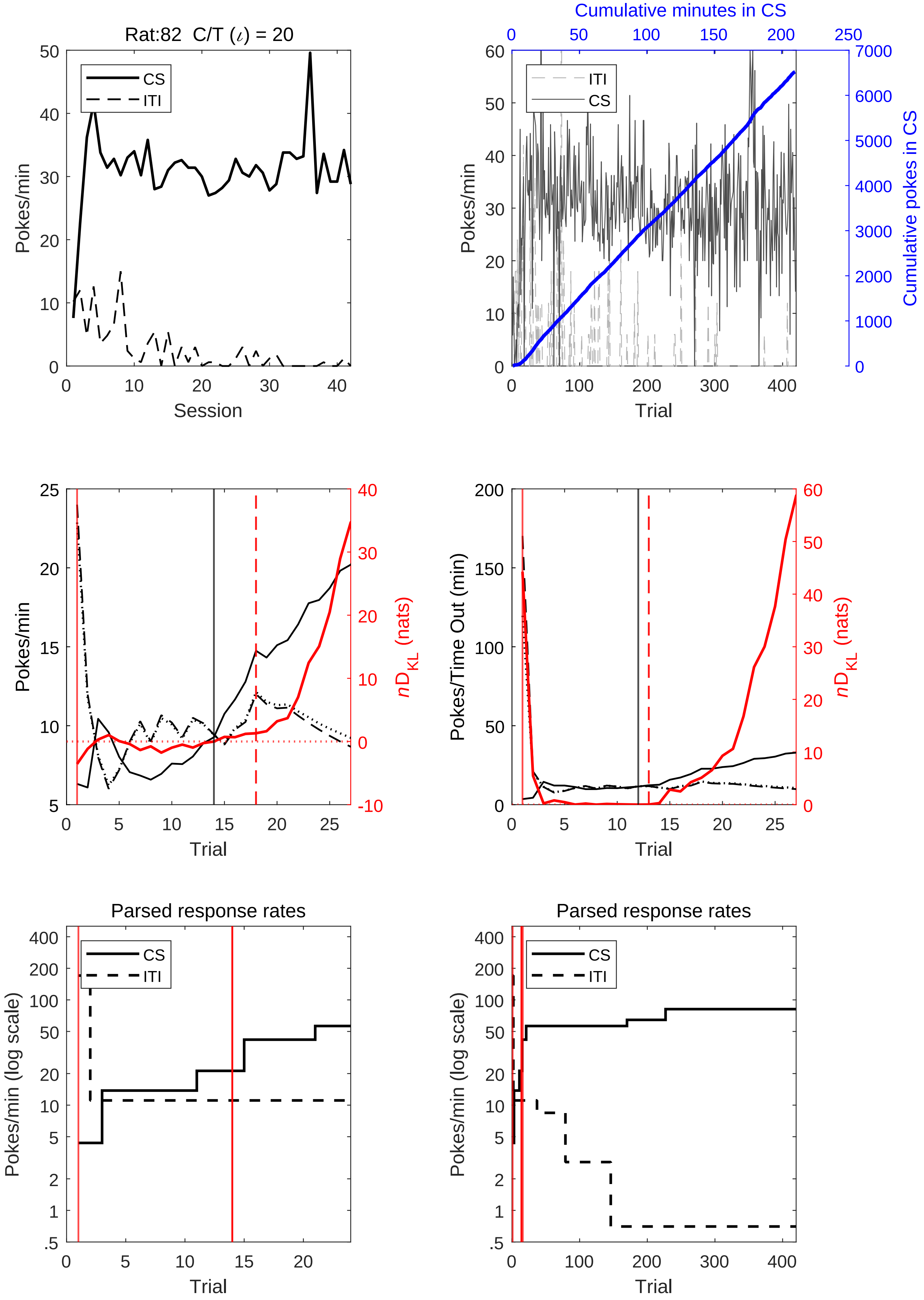

Rat:82 C/T (ι) = 20
CS
ITI
Pokes/min
Session
Cumulative minutes in CS
Cumulative pokes in CS
Trial
nD_KL (nats)
Pokes/Time Out (min)
Parsed response rates
Pokes/min (log scale)

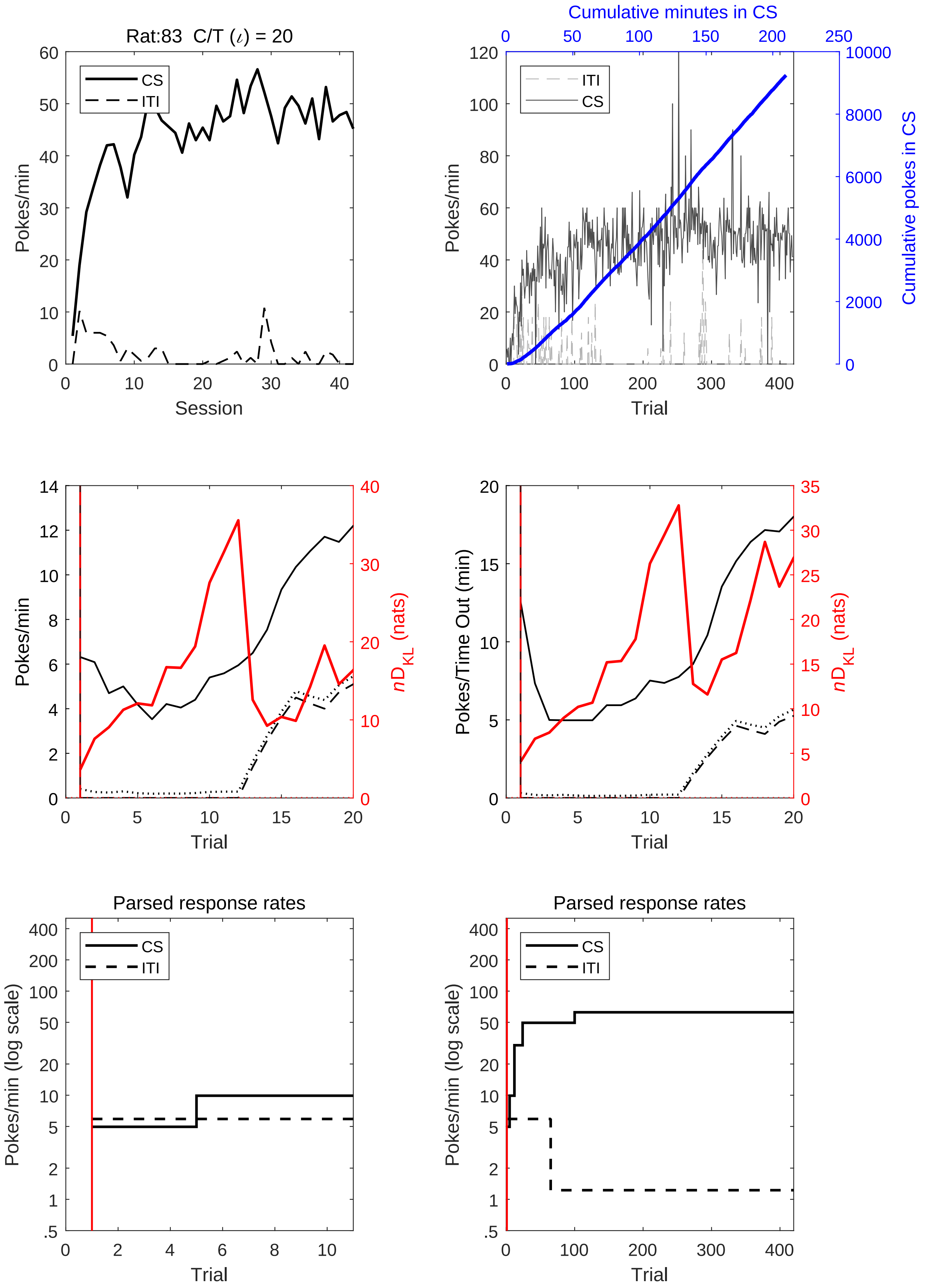
Rat:83 C/T (ι) = 20
Pokes/min
Session
CS
ITI
Cumulative minutes in CS
Cumulative pokes in CS
Trial
Pokes/Time Out (min)
nD_KL (nats)
Parsed response rates
Pokes/min (log scale)

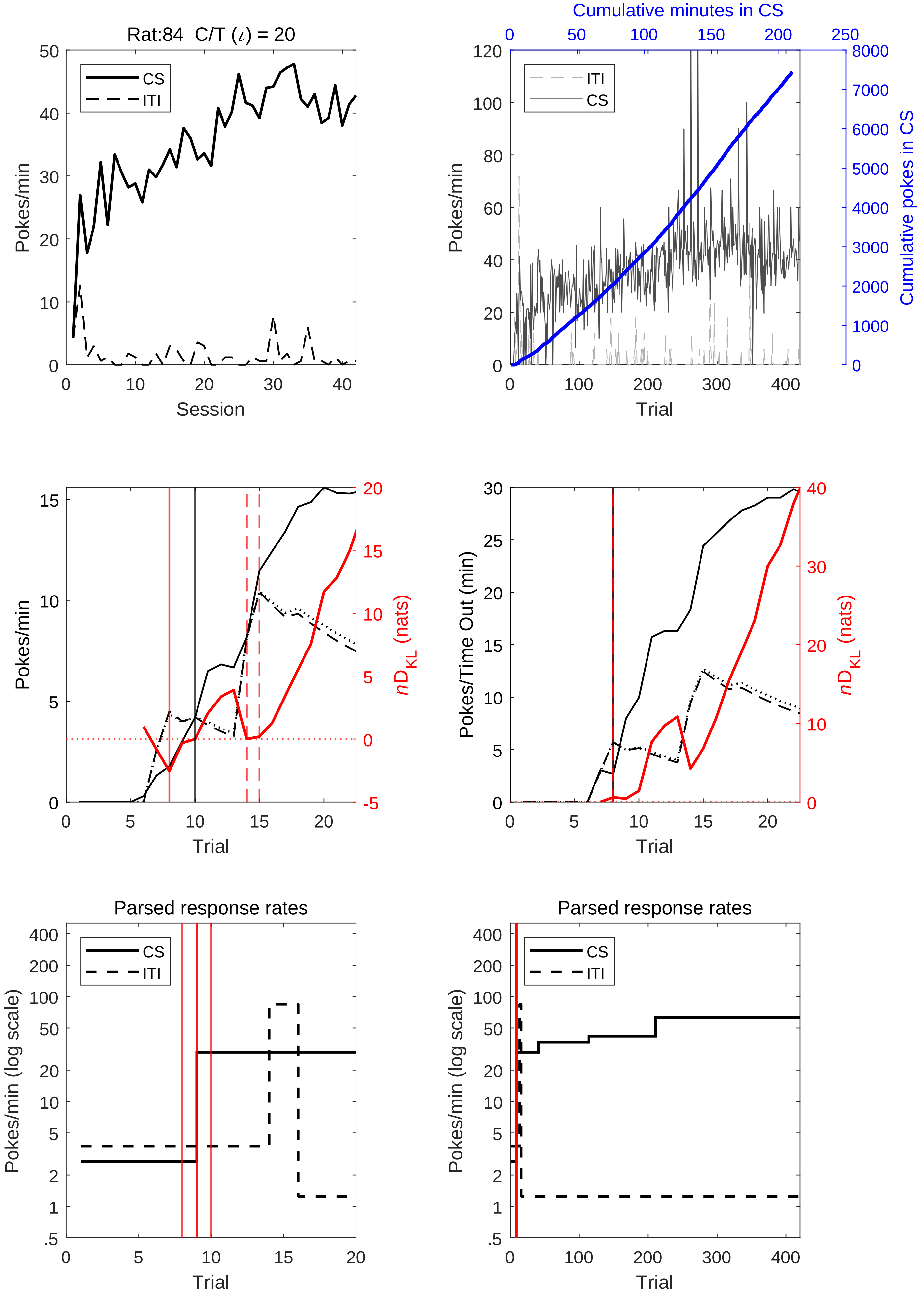

Rat:84  C/T (ι) = 20
CS
ITI
Pokes/min
Session
Cumulative minutes in CS
ITI
CS
Pokes/min
Cumulative pokes in CS
Trial
Pokes/min
nD_KL (nats)
Trial
Pokes/Time Out (min)
nD_KL (nats)
Trial
Parsed response rates
CS
ITI
Pokes/min (log scale)
Trial
Parsed response rates
CS
ITI
Pokes/min (log scale)
Trial

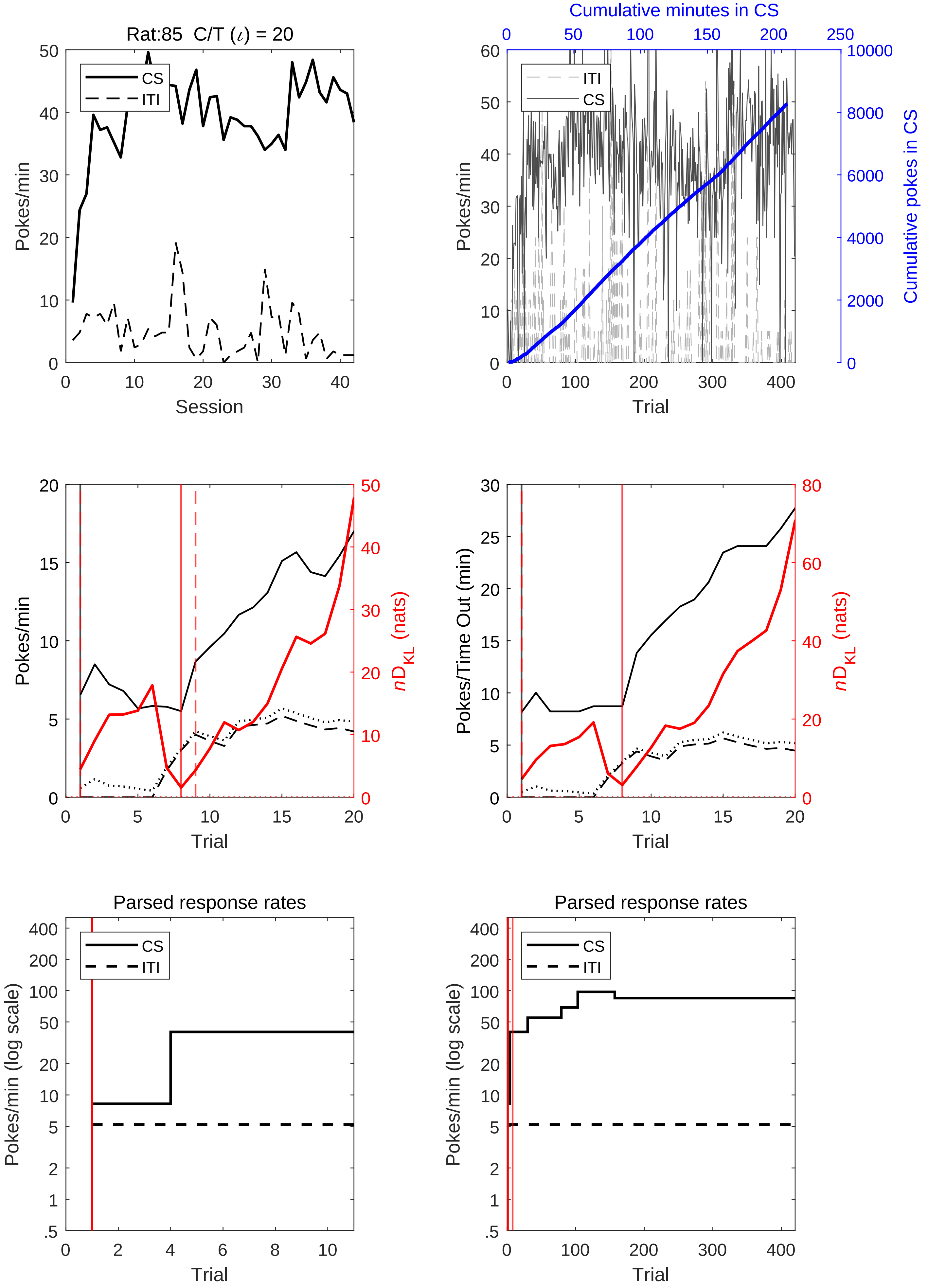
Rat:85 C/T (ι) = 20
CS
ITI
Pokes/min
Session
Cumulative minutes in CS
Cumulative pokes in CS
Trial
nD_KL (nats)
Pokes/Time Out (min)
Parsed response rates
Pokes/min (log scale)

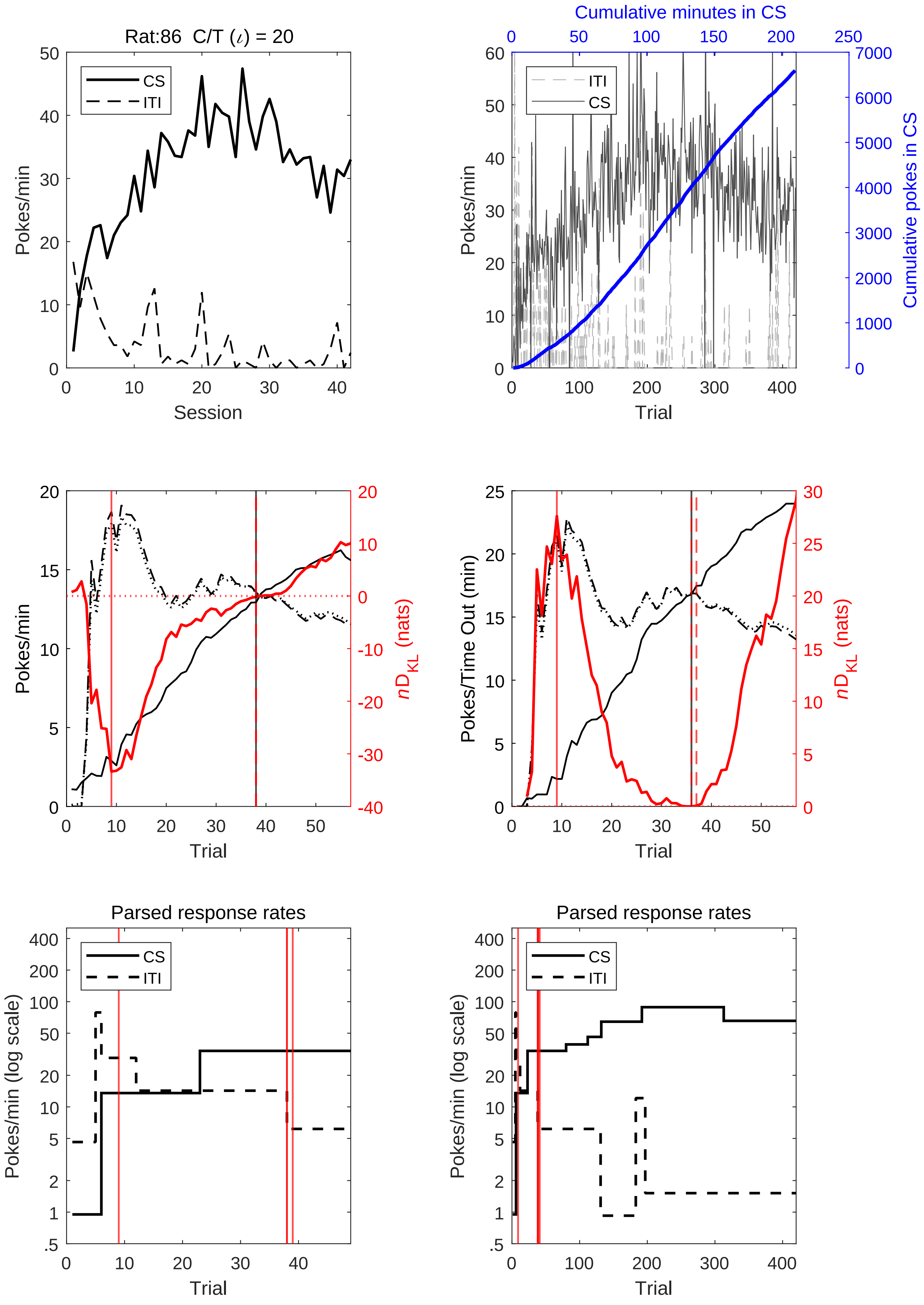
Rat:86 C/T (ι) = 20
CS
ITI
Pokes/min
Session
Cumulative minutes in CS
Cumulative pokes in CS
Trial
Pokes/Time Out (min)
nD_KL (nats)
Parsed response rates
Pokes/min (log scale)

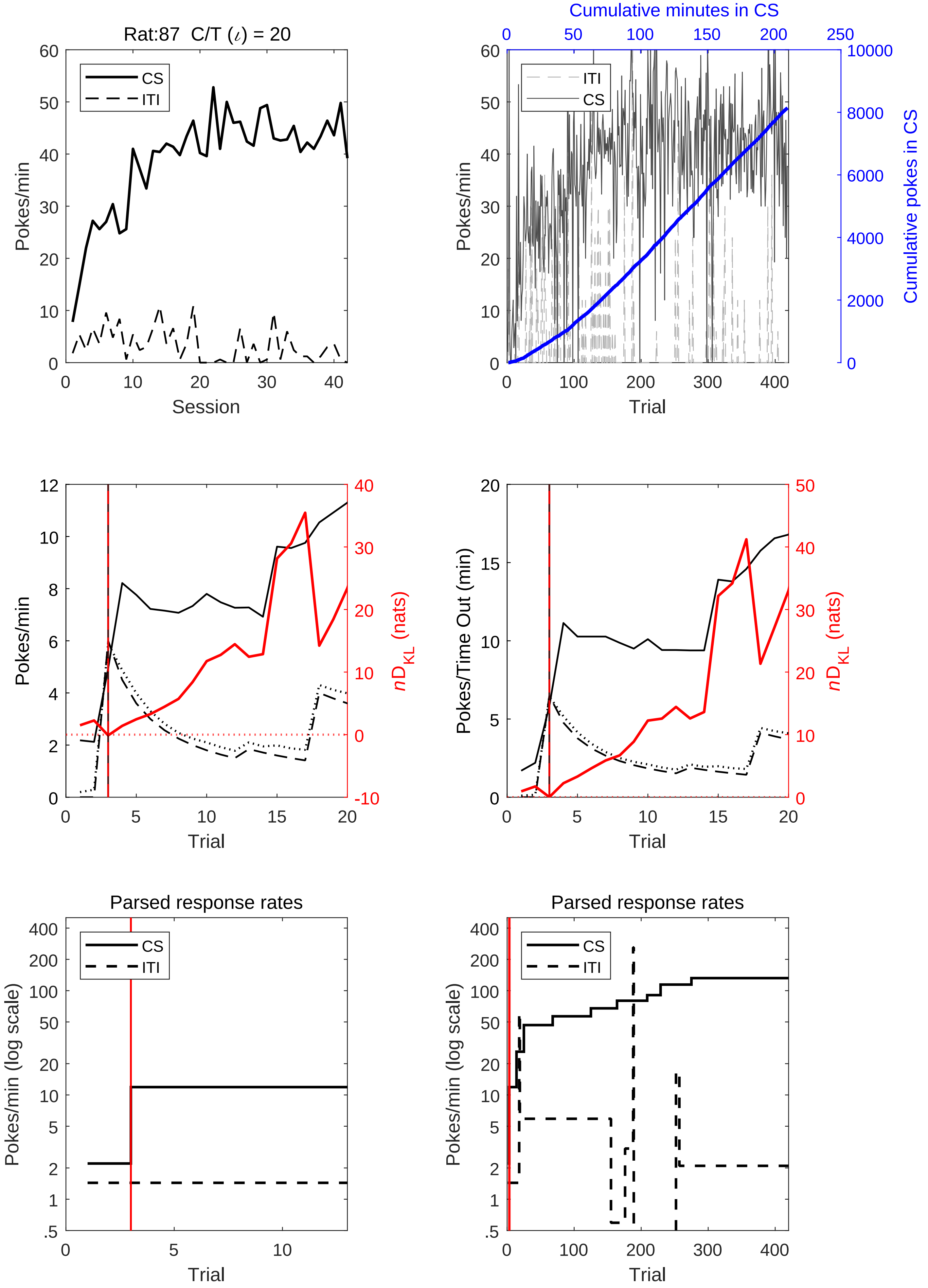
Rat:87 C/T (ι) = 20
CS
ITI
Pokes/min
Session
Cumulative minutes in CS
Cumulative pokes in CS
Trial
nD_KL (nats)
Pokes/Time Out (min)
Parsed response rates
Pokes/min (log scale)

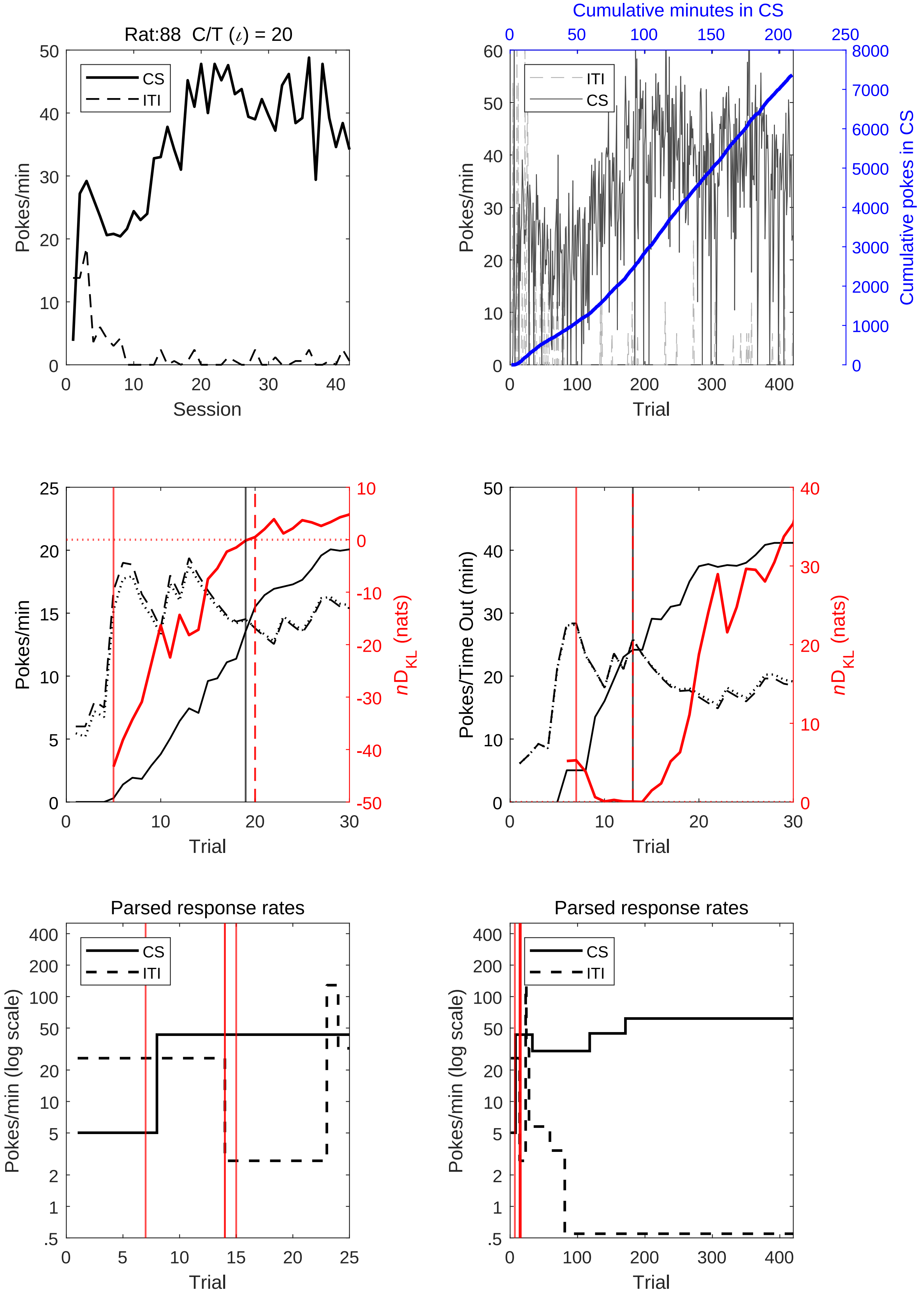
Rat:88 C/T (ι) = 20
CS
ITI
Pokes/min
Session
Cumulative minutes in CS
Cumulative pokes in CS
ITI
CS
Pokes/min
Trial
Pokes/min
$nD_{KL}$ (nats)
Trial
Pokes/Time Out (min)
$nD_{KL}$ (nats)
Trial
Parsed response rates
CS
ITI
Pokes/min (log scale)
Trial
Parsed response rates
CS
ITI
Pokes/min (log scale)
Trial

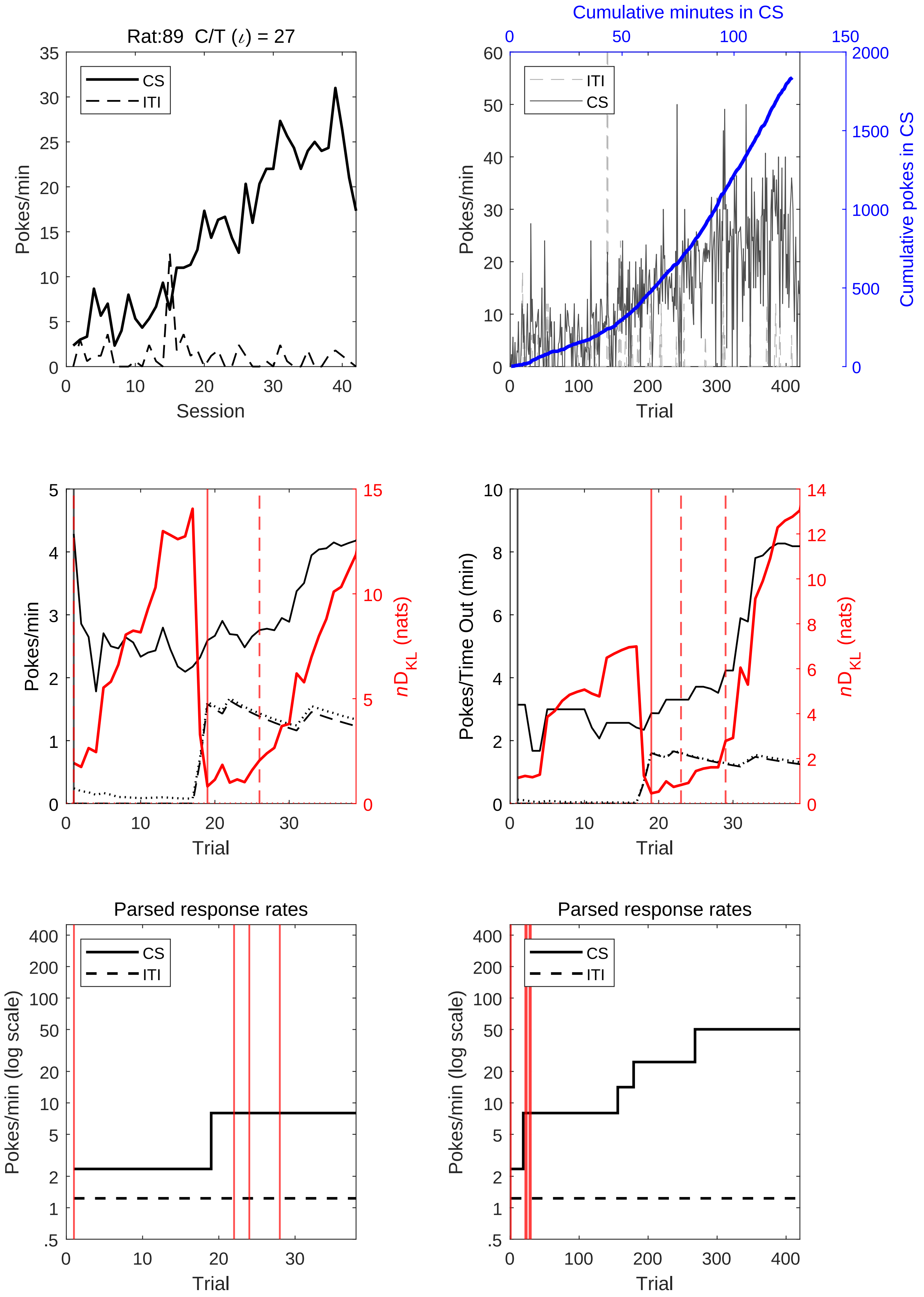

Rat:89 C/T ($\iota$) = 27
CS
ITI
Pokes/min
Session
Cumulative minutes in CS
Cumulative pokes in CS
Trial
$nD_{KL}$ (nats)
Pokes/Time Out (min)
Parsed response rates
Pokes/min (log scale)

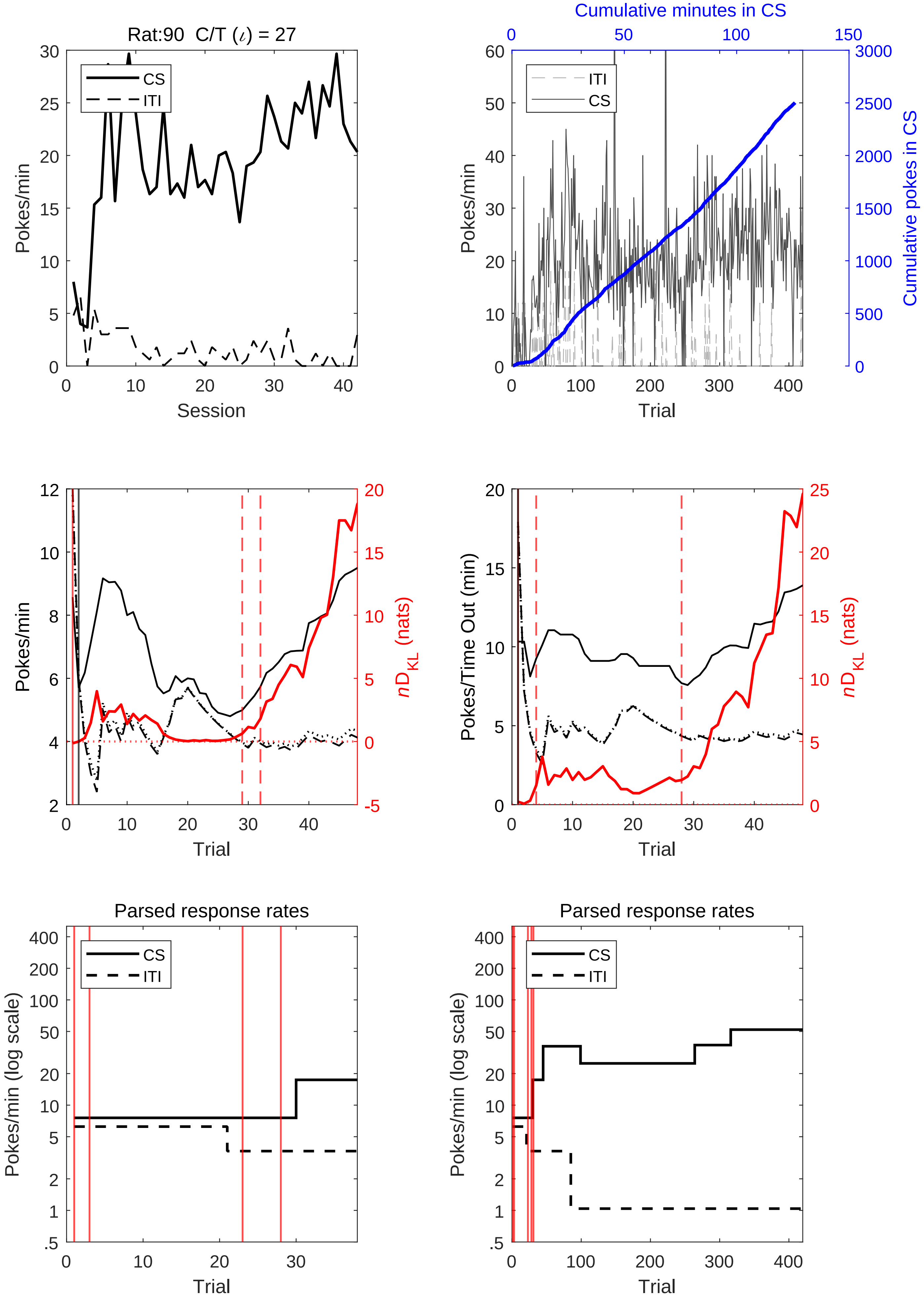
Rat:90 C/T (ι) = 27
CS
ITI
Pokes/min
Session
Cumulative minutes in CS
Cumulative pokes in CS
Trial
Pokes/Time Out (min)
nD_KL (nats)
Parsed response rates
Pokes/min (log scale)

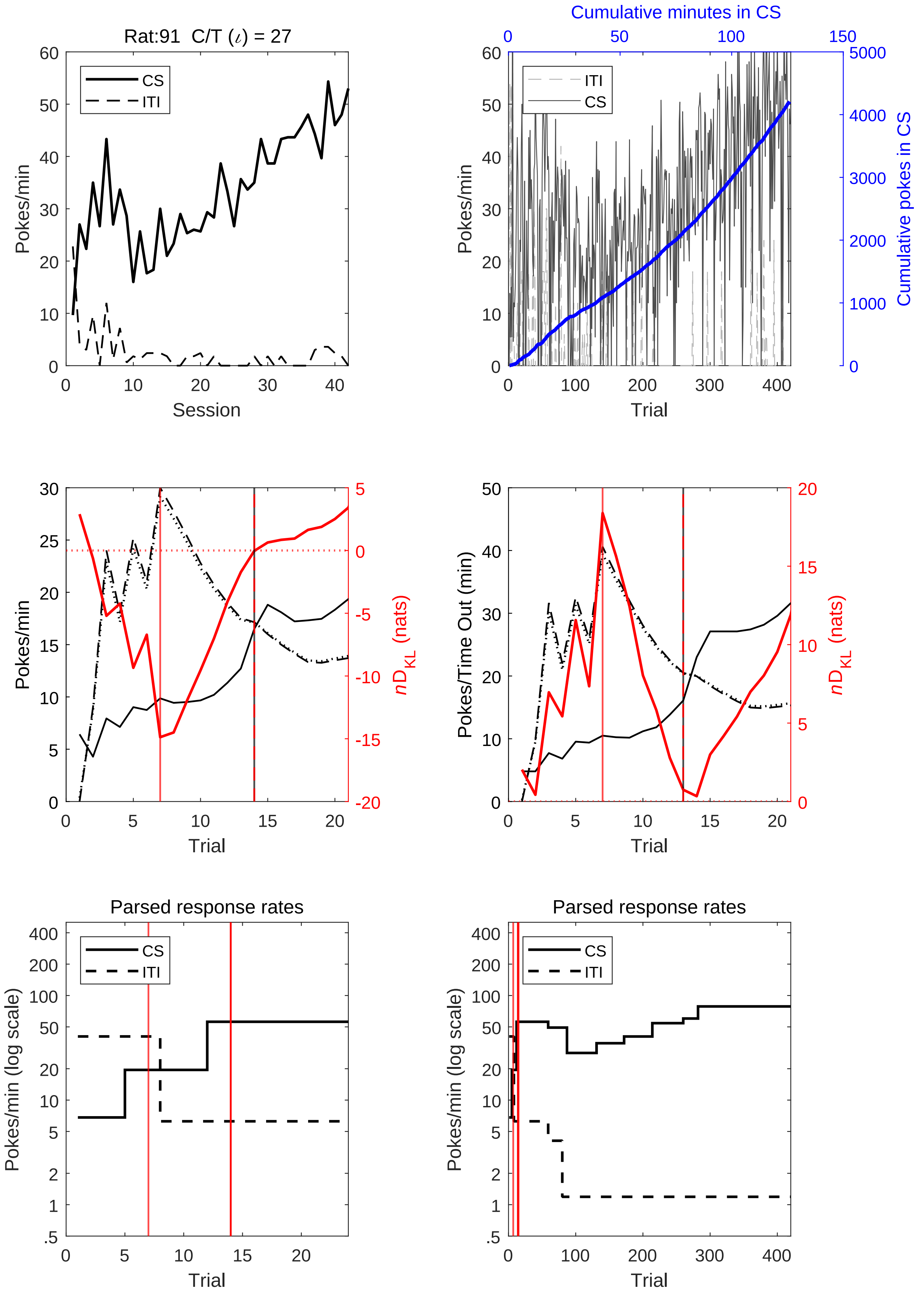

Rat:91 C/T (ι) = 27
Pokes/min
Session
CS
ITI
Cumulative minutes in CS
Cumulative pokes in CS
Trial
Pokes/Time Out (min)
$nD_{KL}$ (nats)
Parsed response rates
Pokes/min (log scale)

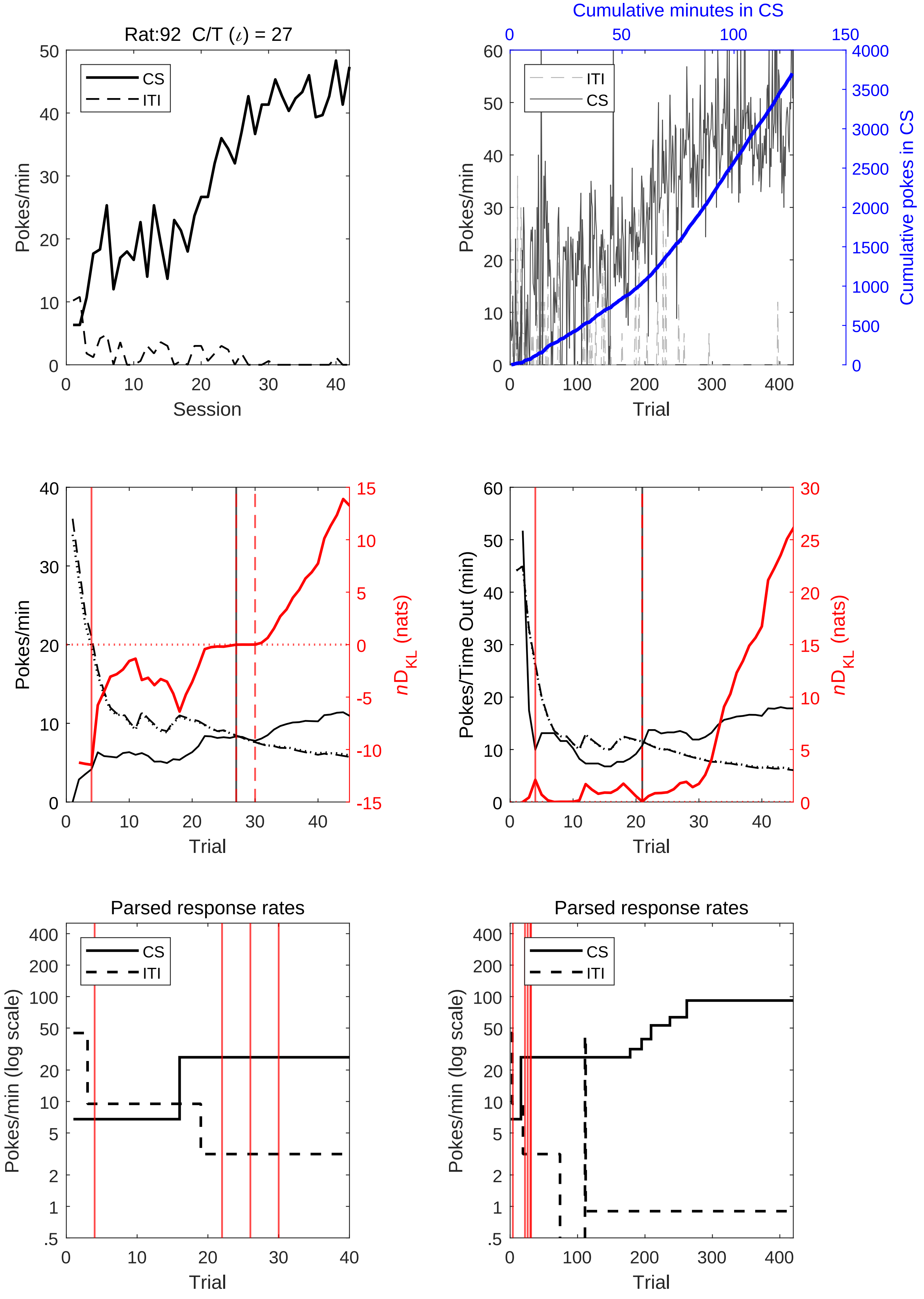

Rat:92 C/T (ι) = 27
CS
ITI
Pokes/min
Session
Cumulative minutes in CS
Cumulative pokes in CS
Trial
nD_KL (nats)
Pokes/Time Out (min)
Parsed response rates
Pokes/min (log scale)

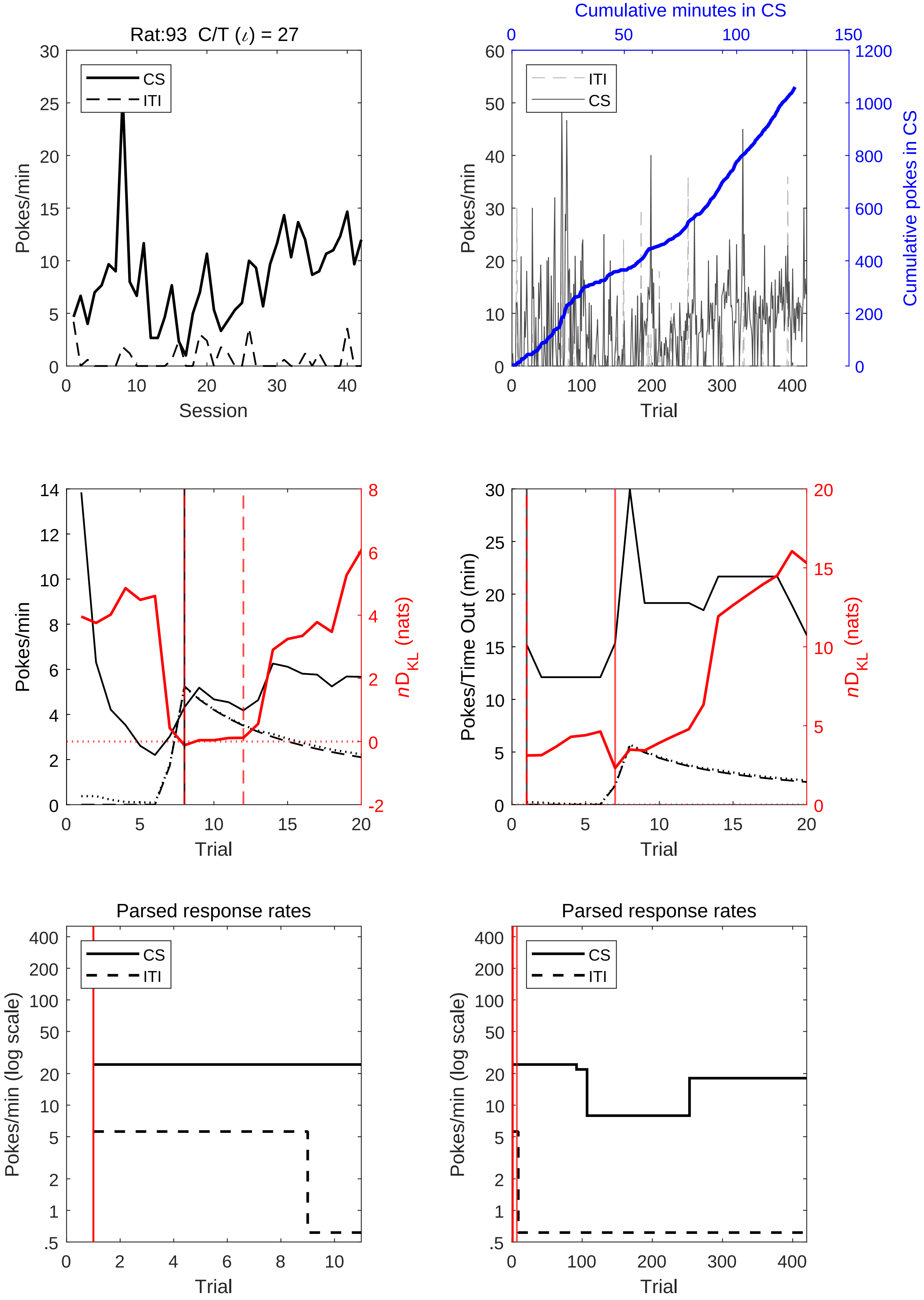

Rat:93  C/T (ι) = 27
CS
ITI
Pokes/min
Session
Cumulative minutes in CS
Cumulative pokes in CS
Trial
nD_KL (nats)
Pokes/Time Out (min)
Parsed response rates
Pokes/min (log scale)

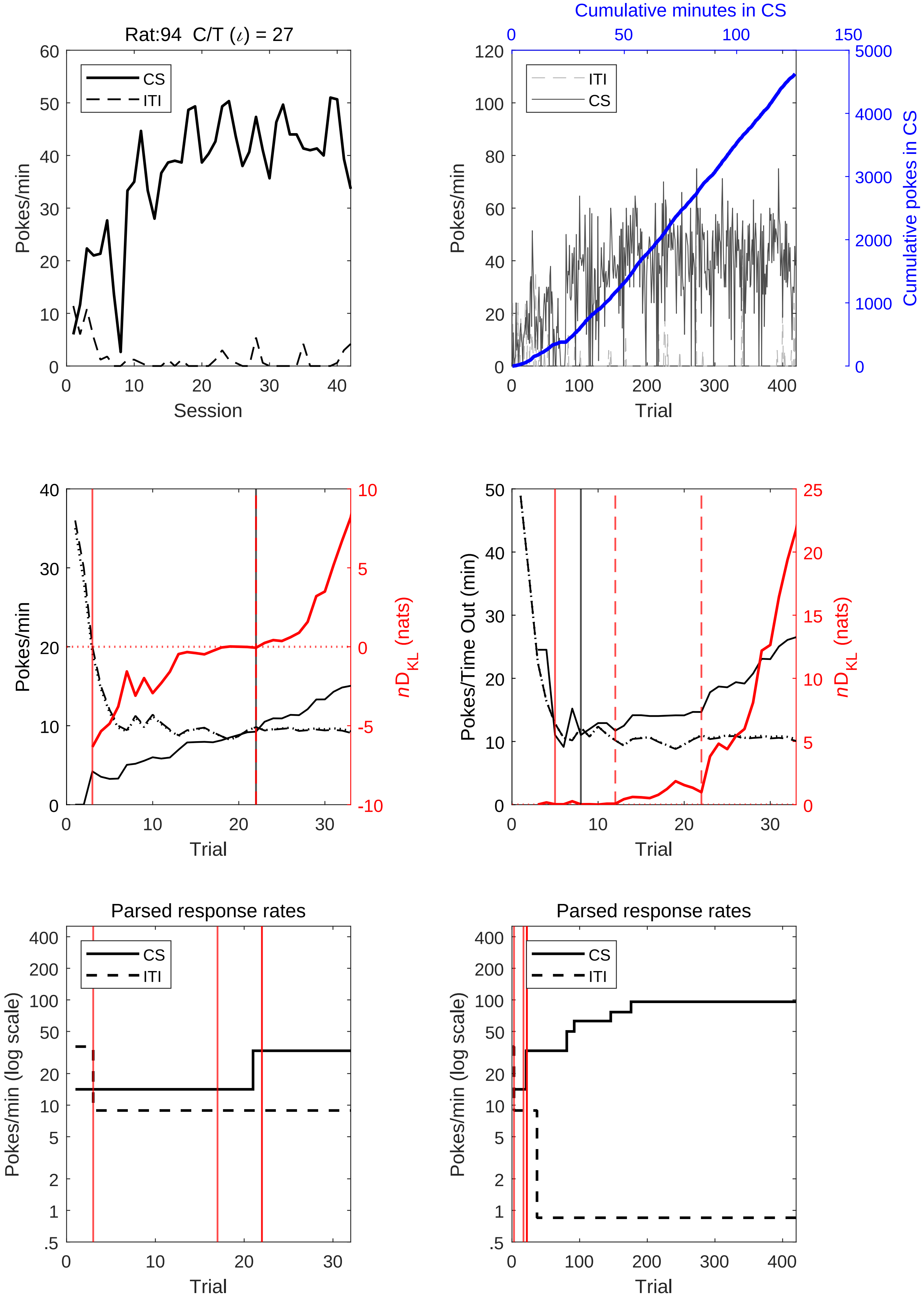
Rat:94 C/T (ι) = 27
CS
ITI
Pokes/min
Session
Cumulative minutes in CS
ITI
CS
Pokes/min
Cumulative pokes in CS
Trial
Pokes/min
nD_KL (nats)
Trial
Pokes/Time Out (min)
nD_KL (nats)
Trial
Parsed response rates
CS
ITI
Pokes/min (log scale)
Trial
Parsed response rates
CS
ITI
Pokes/min (log scale)
Trial

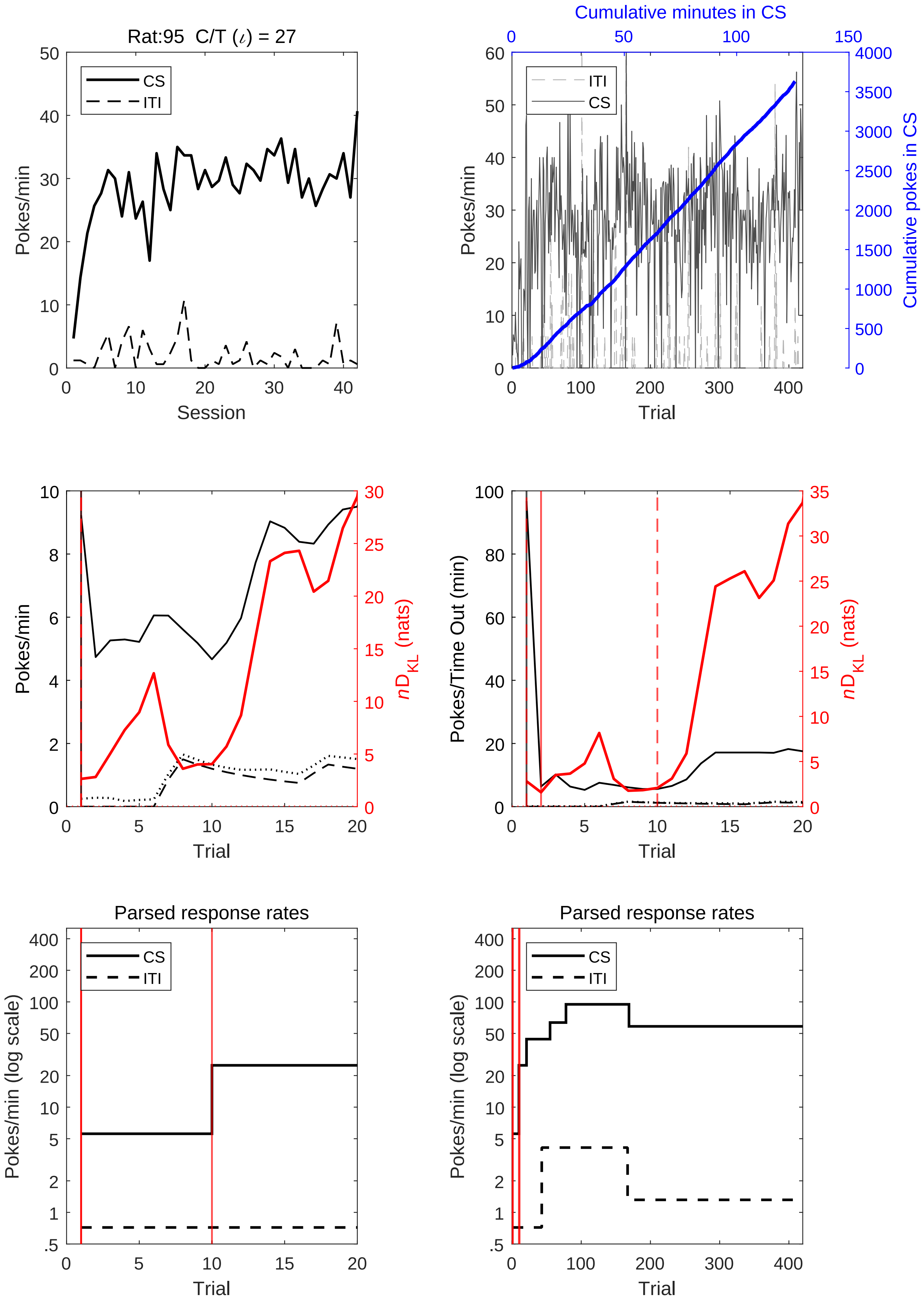
Rat:95 C/T (ι) = 27
CS
ITI
Pokes/min
Session
Cumulative minutes in CS
Cumulative pokes in CS
Trial
nD_KL (nats)
Pokes/Time Out (min)
Parsed response rates
Pokes/min (log scale)

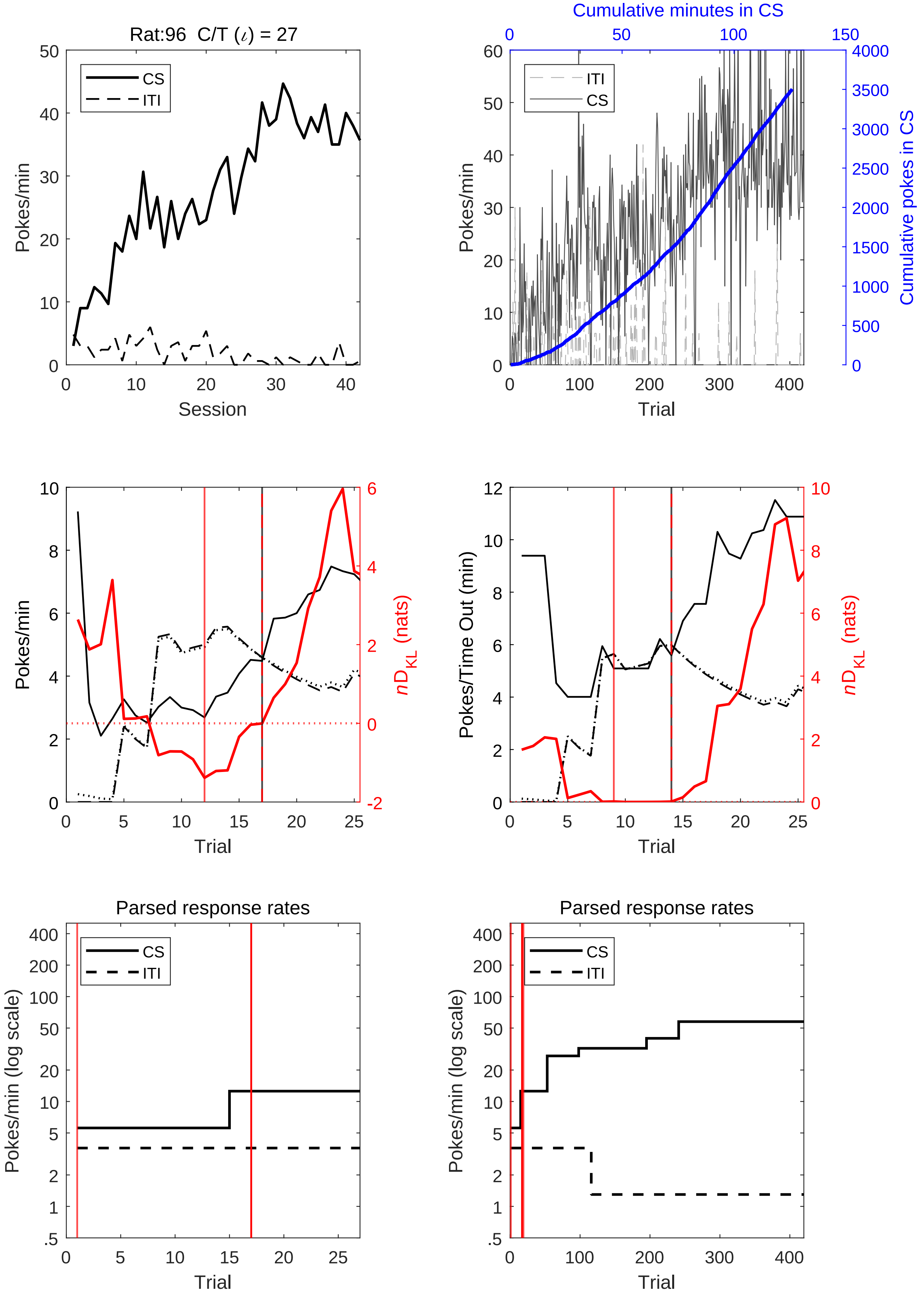

Rat:96 C/T (ι) = 27
CS
ITI
Pokes/min
Session
Cumulative minutes in CS
Cumulative pokes in CS
Trial
nD_KL (nats)
Pokes/Time Out (min)
Parsed response rates
Pokes/min (log scale)

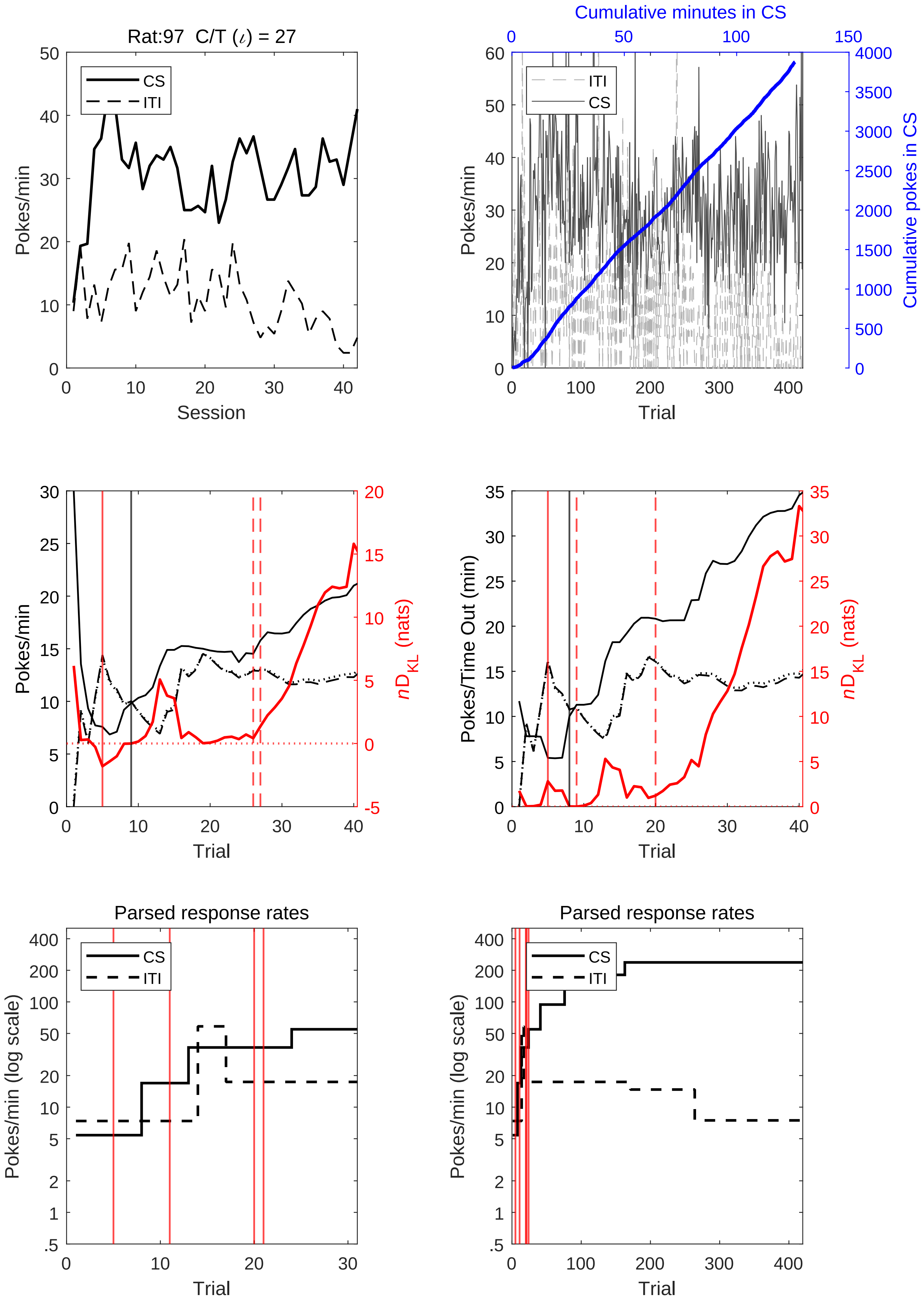
Rat:97 C/T (ι) = 27
CS
ITI
Pokes/min
Session
Cumulative minutes in CS
Cumulative pokes in CS
Trial
nD_KL (nats)
Pokes/Time Out (min)
Parsed response rates
Pokes/min (log scale)

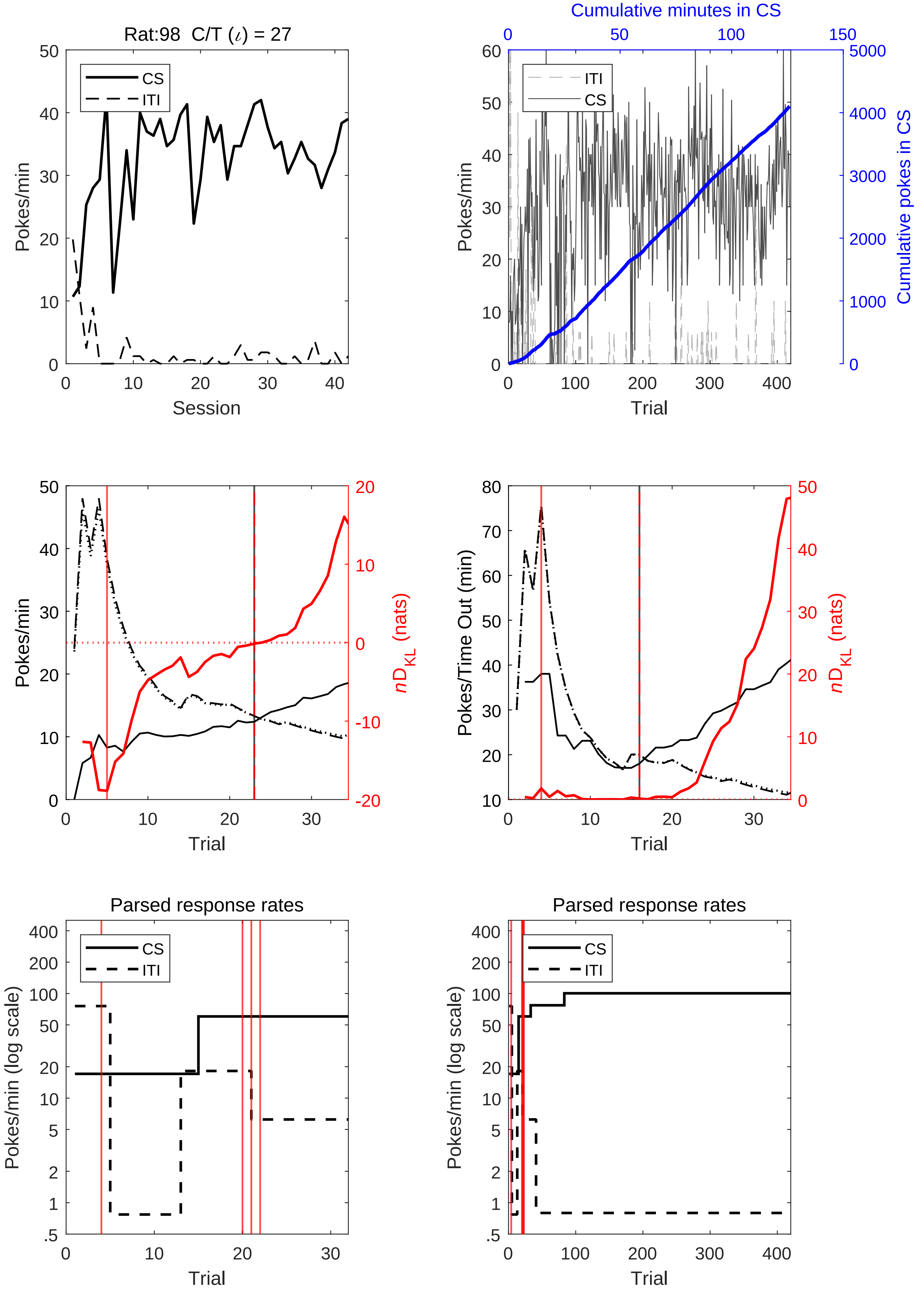

Rat:98 C/T ($\iota$) = 27
CS
ITI
Pokes/min
Session
Cumulative minutes in CS
Cumulative pokes in CS
Trial
$nD_{KL}$ (nats)
Pokes/Time Out (min)
Parsed response rates
Pokes/min (log scale)

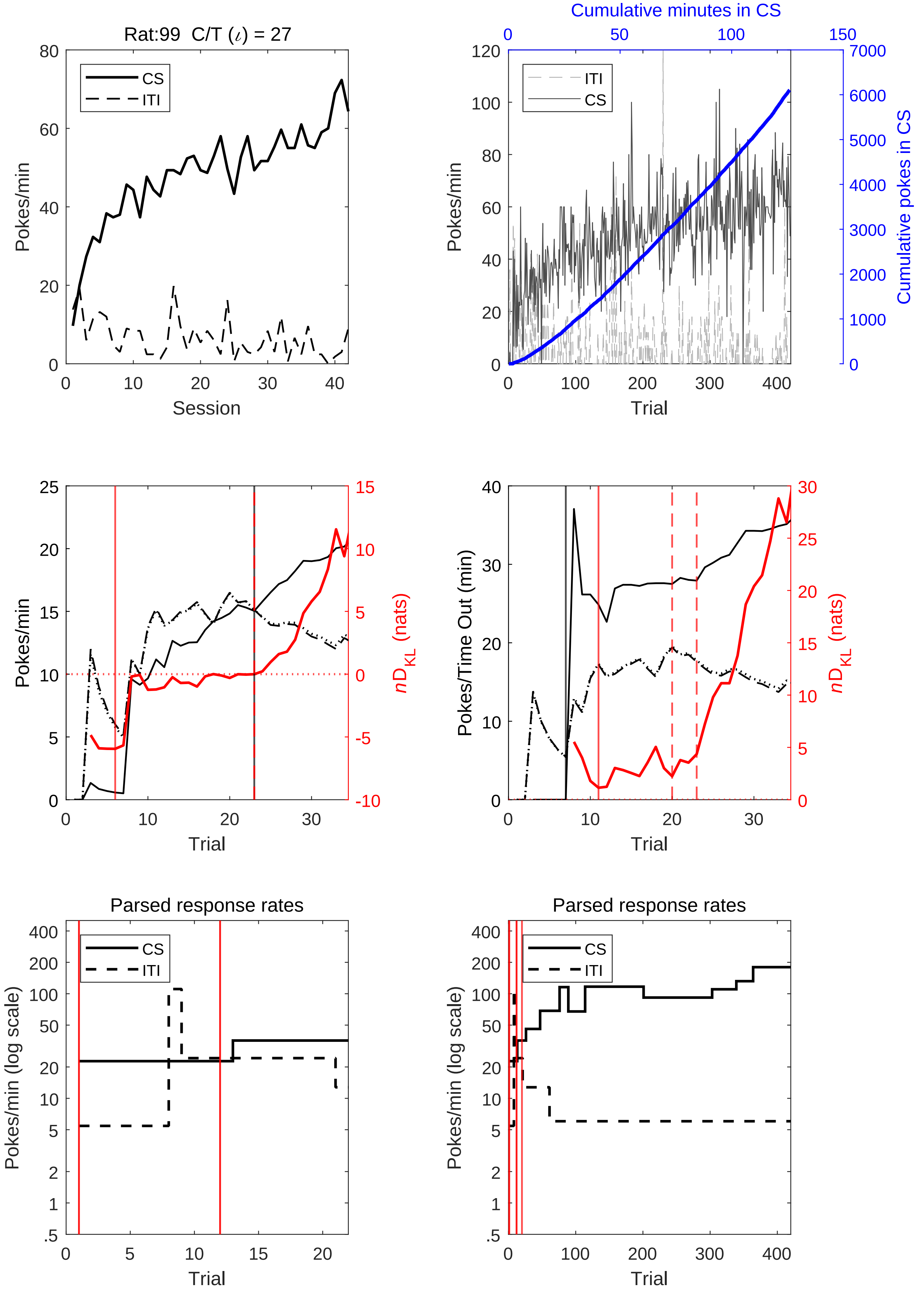
Rat:99 C/T (ι) = 27
CS
ITI
Pokes/min
Session
Cumulative minutes in CS
Cumulative pokes in CS
Trial
nD_KL (nats)
Pokes/Time Out (min)
Parsed response rates
Pokes/min (log scale)

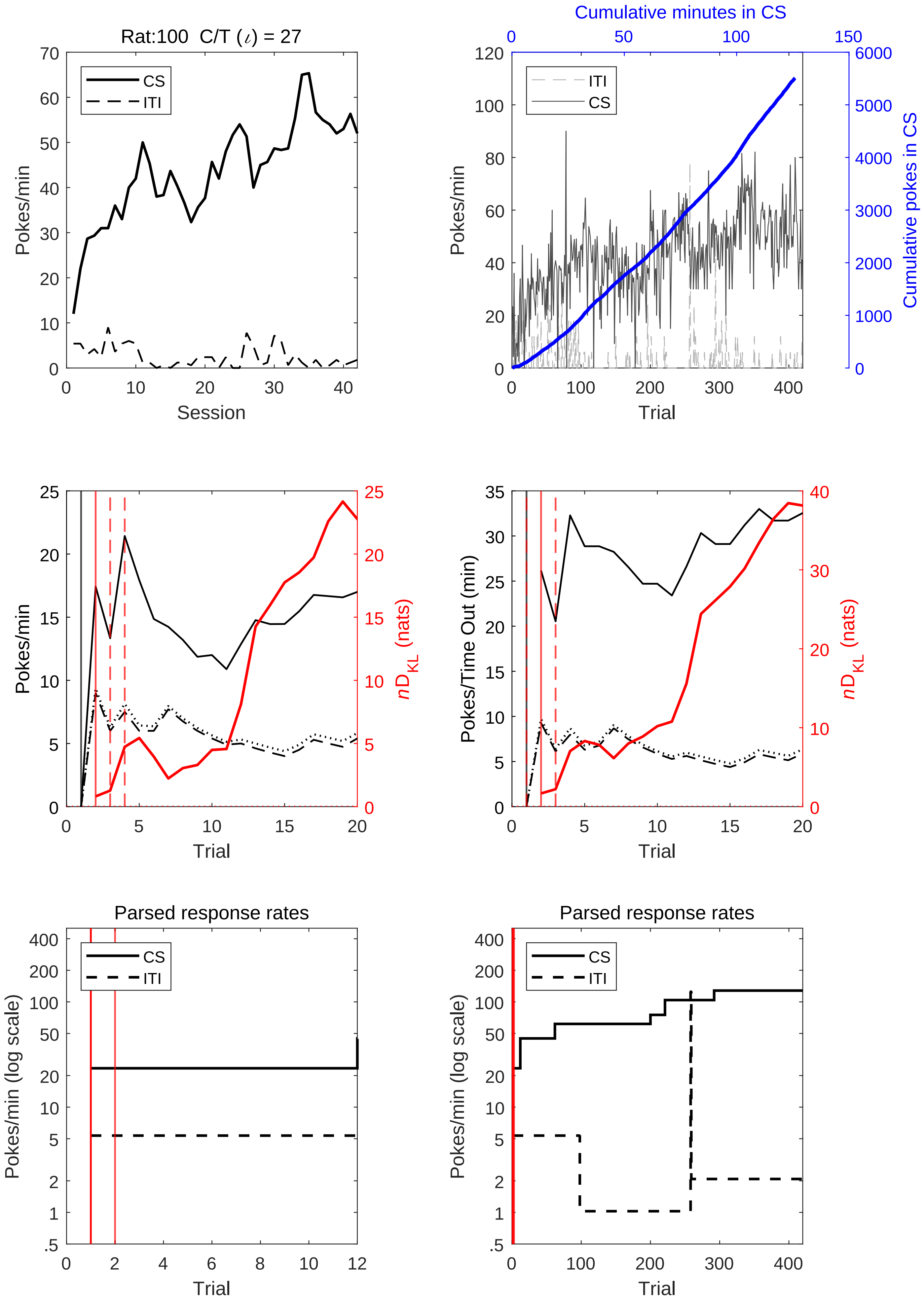

Rat:100 C/T (ι) = 27
CS
ITI
Pokes/min
Session
Cumulative minutes in CS
Cumulative pokes in CS
Trial
$n\mathrm{D}_{\mathrm{KL}}$ (nats)
Pokes/Time Out (min)
Parsed response rates
Pokes/min (log scale)

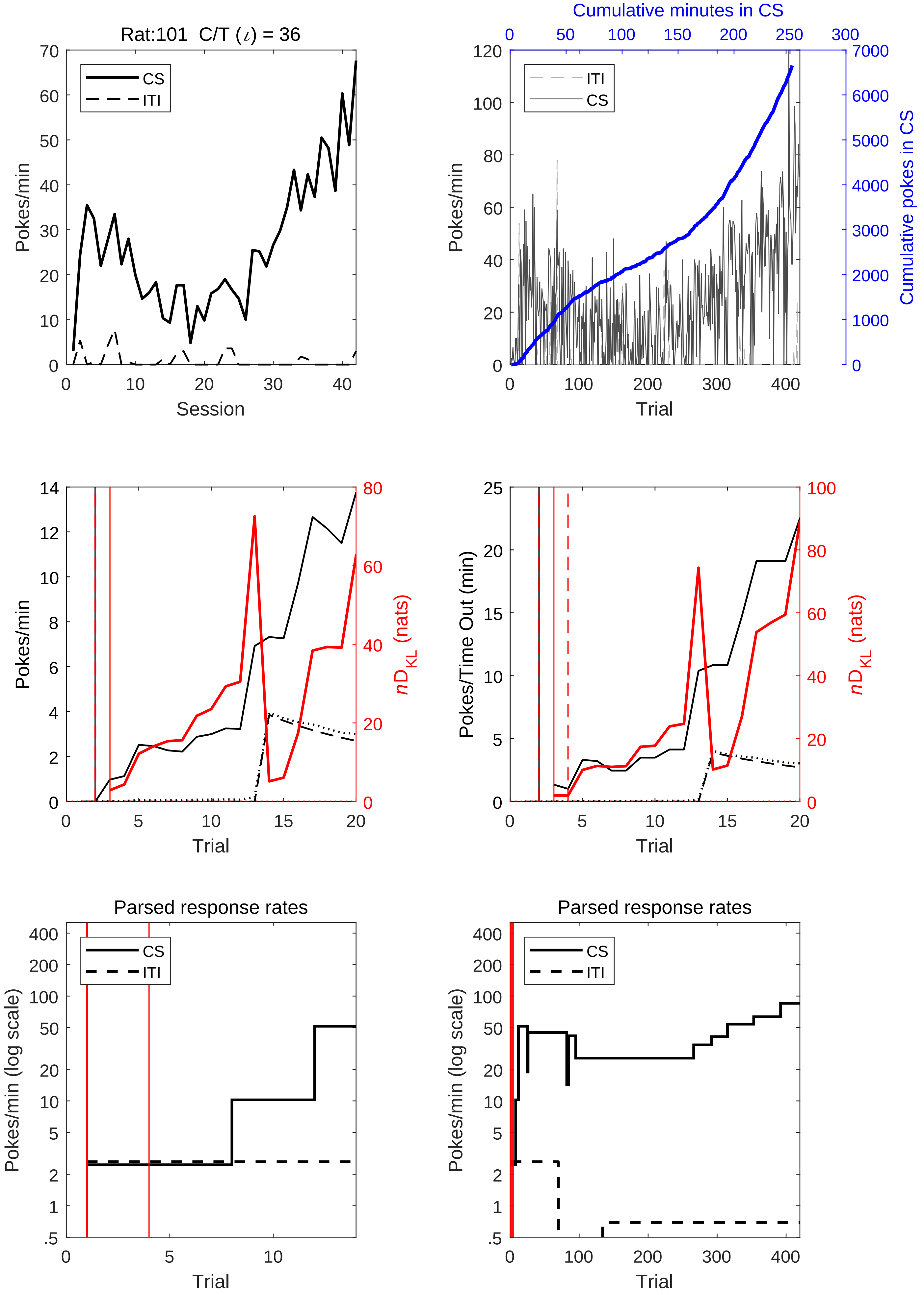
Rat:101 C/T (ι) = 36
CS
ITI
Pokes/min
Session
Cumulative minutes in CS
Cumulative pokes in CS
Trial
Pokes/Time Out (min)
$nD_{KL}$ (nats)
Parsed response rates
Pokes/min (log scale)

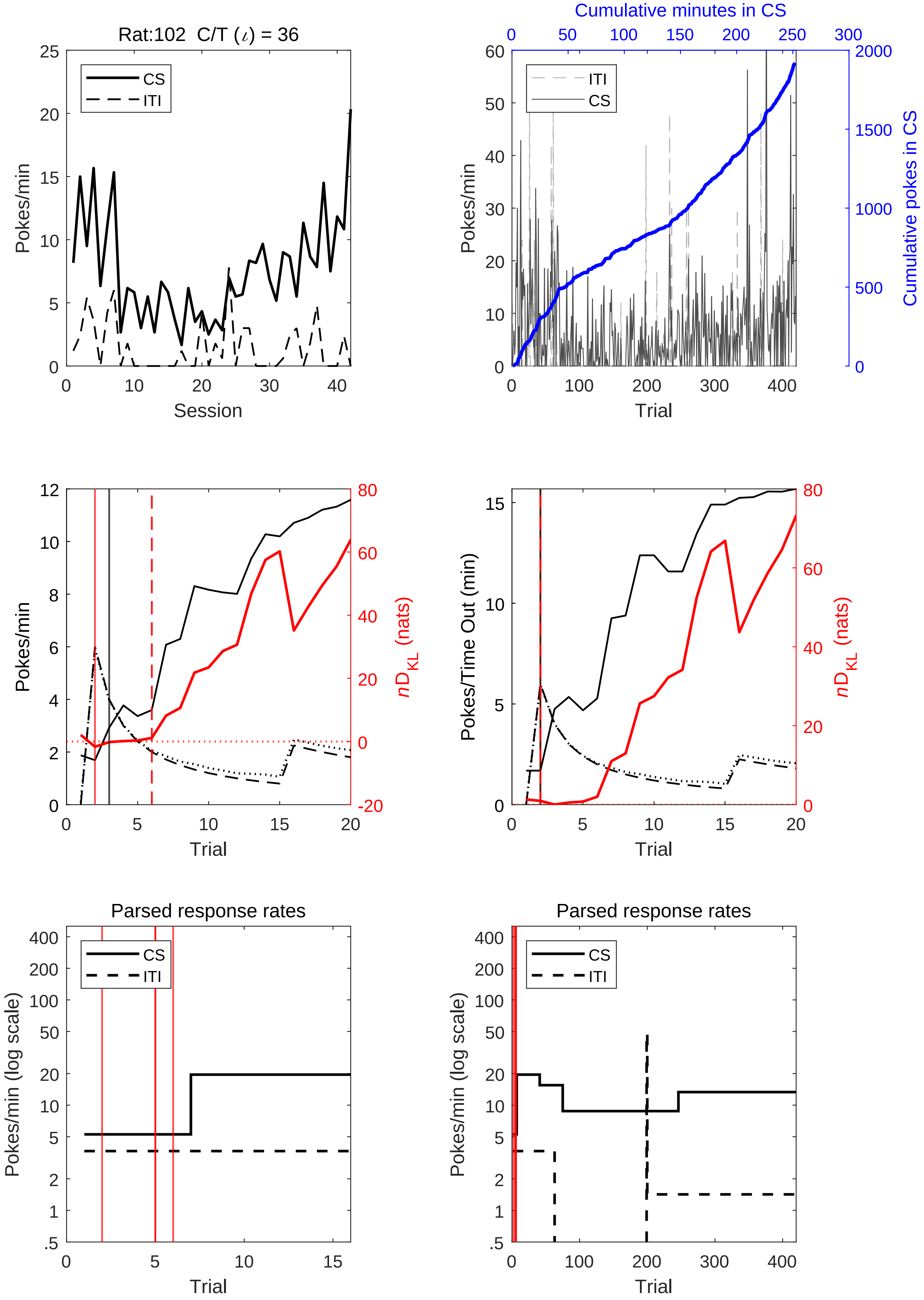

Rat:102 C/T (ι) = 36
CS
ITI
Pokes/min
Session
Cumulative minutes in CS
Cumulative pokes in CS
Trial
Pokes/Time Out (min)
nD_KL (nats)
Parsed response rates
Pokes/min (log scale)

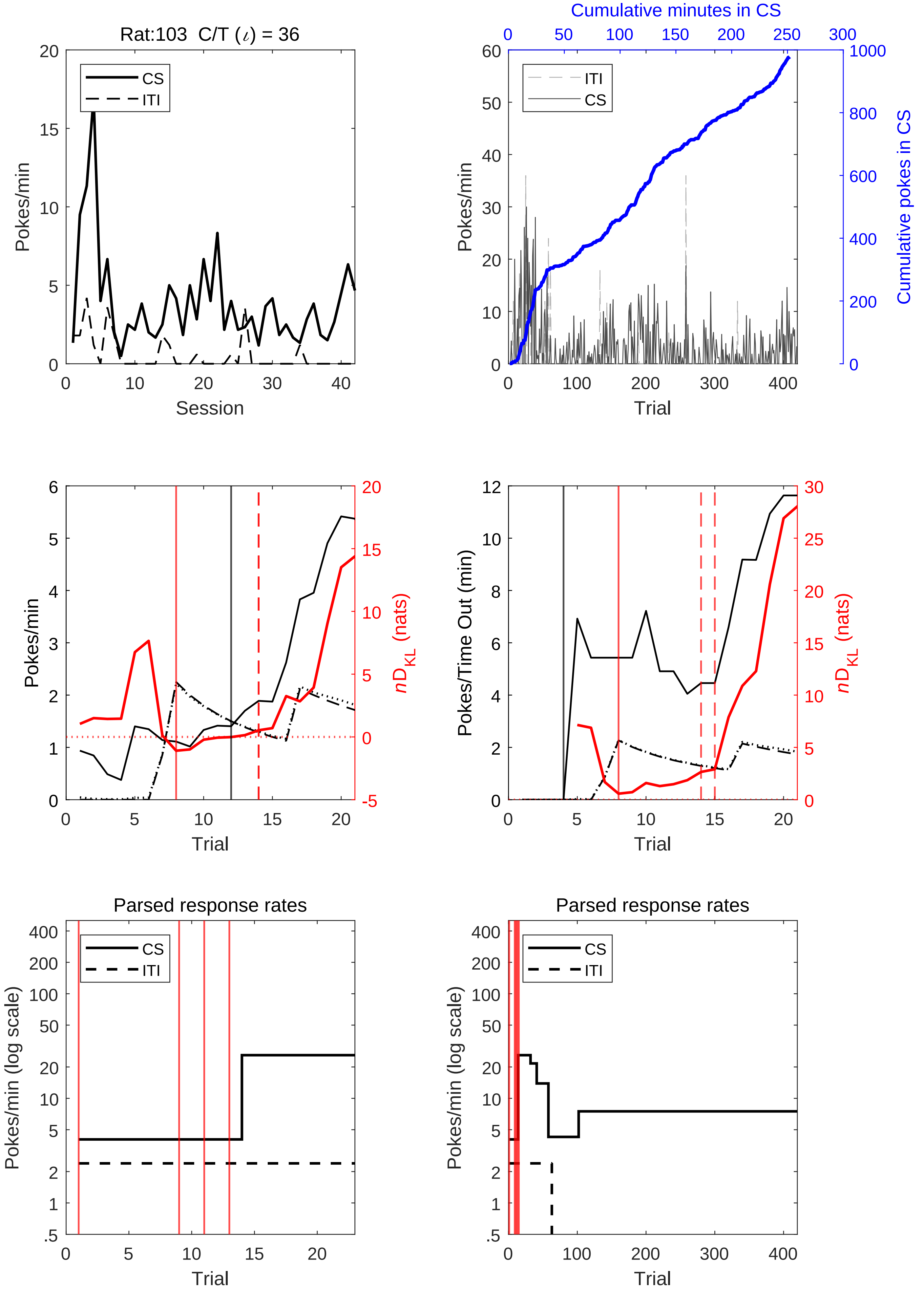

Rat:103 C/T (ι) = 36
CS
ITI
Pokes/min
Session
Cumulative minutes in CS
Cumulative pokes in CS
Trial
nD_KL (nats)
Pokes/Time Out (min)
Parsed response rates
Pokes/min (log scale)

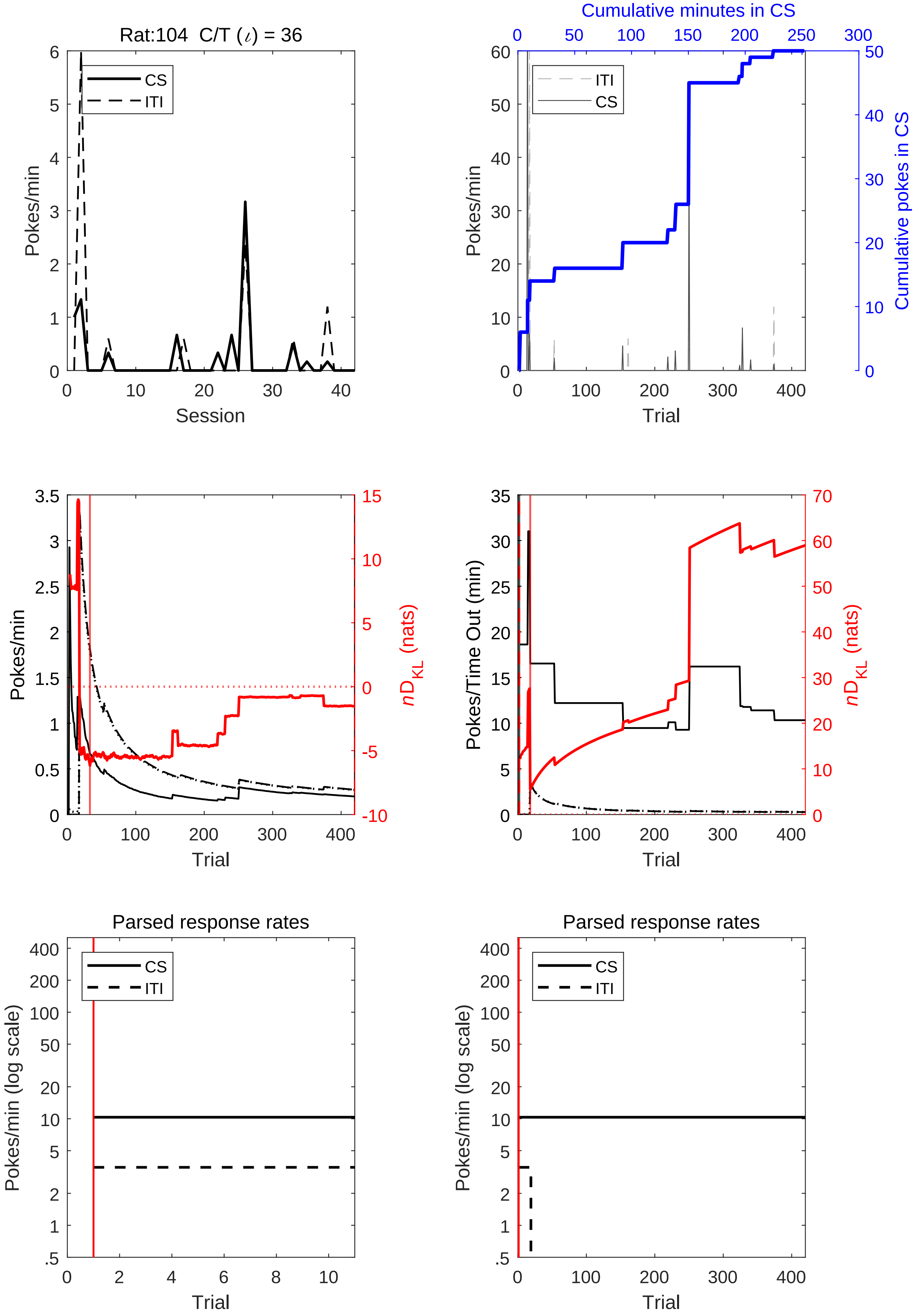

Rat:104 C/T (ι) = 36
CS
ITI
Pokes/min
Session
Cumulative minutes in CS
Cumulative pokes in CS
Trial
nD_KL (nats)
Pokes/Time Out (min)
Parsed response rates
Pokes/min (log scale)

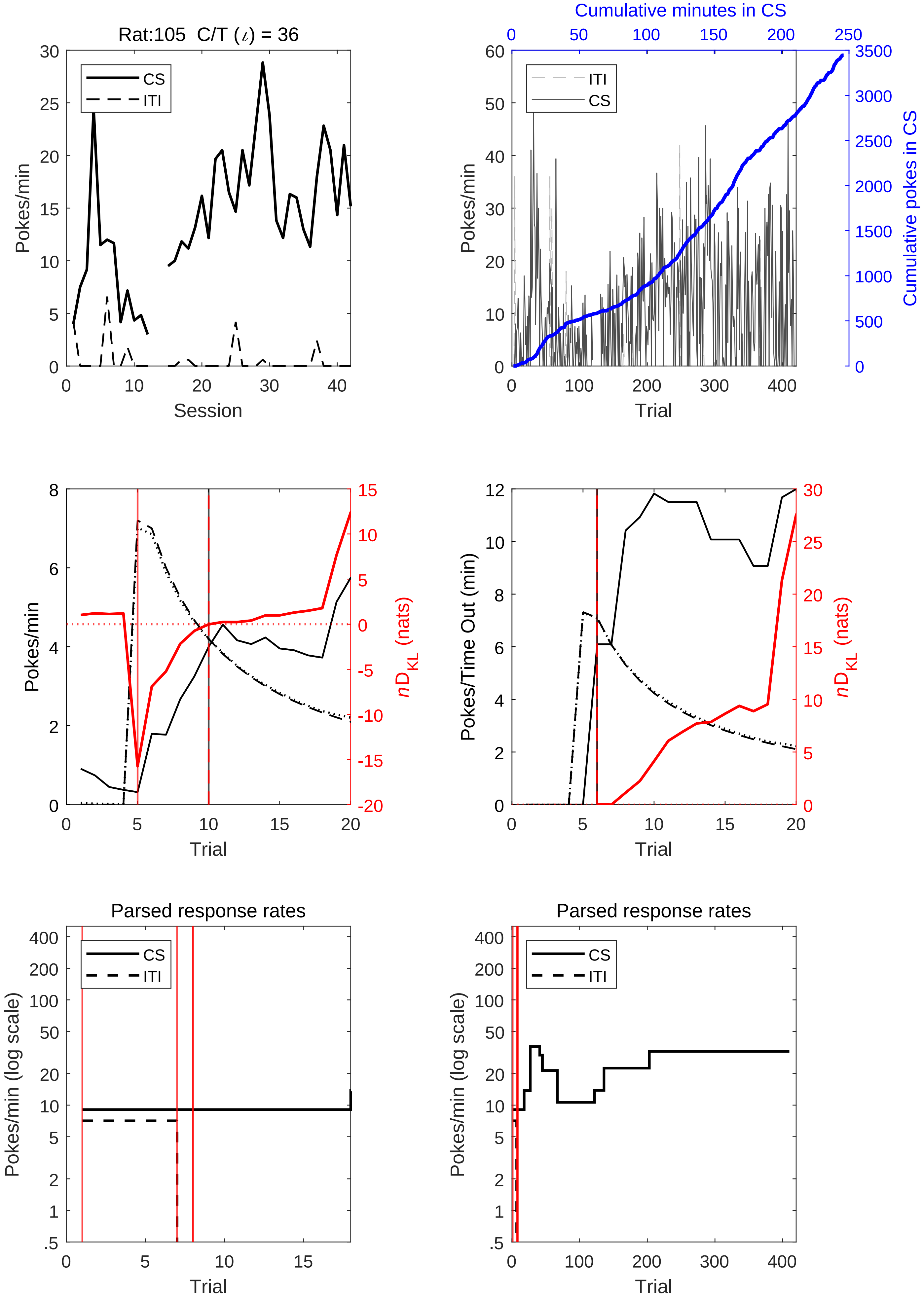

Rat:105 C/T (ι) = 36
CS
ITI
Pokes/min
Session
Cumulative minutes in CS
ITI
CS
Pokes/min
Cumulative pokes in CS
Trial
Pokes/min
nD_KL (nats)
Trial
Pokes/Time Out (min)
nD_KL (nats)
Trial
Parsed response rates
CS
ITI
Pokes/min (log scale)
Trial
Parsed response rates
CS
ITI
Pokes/min (log scale)
Trial

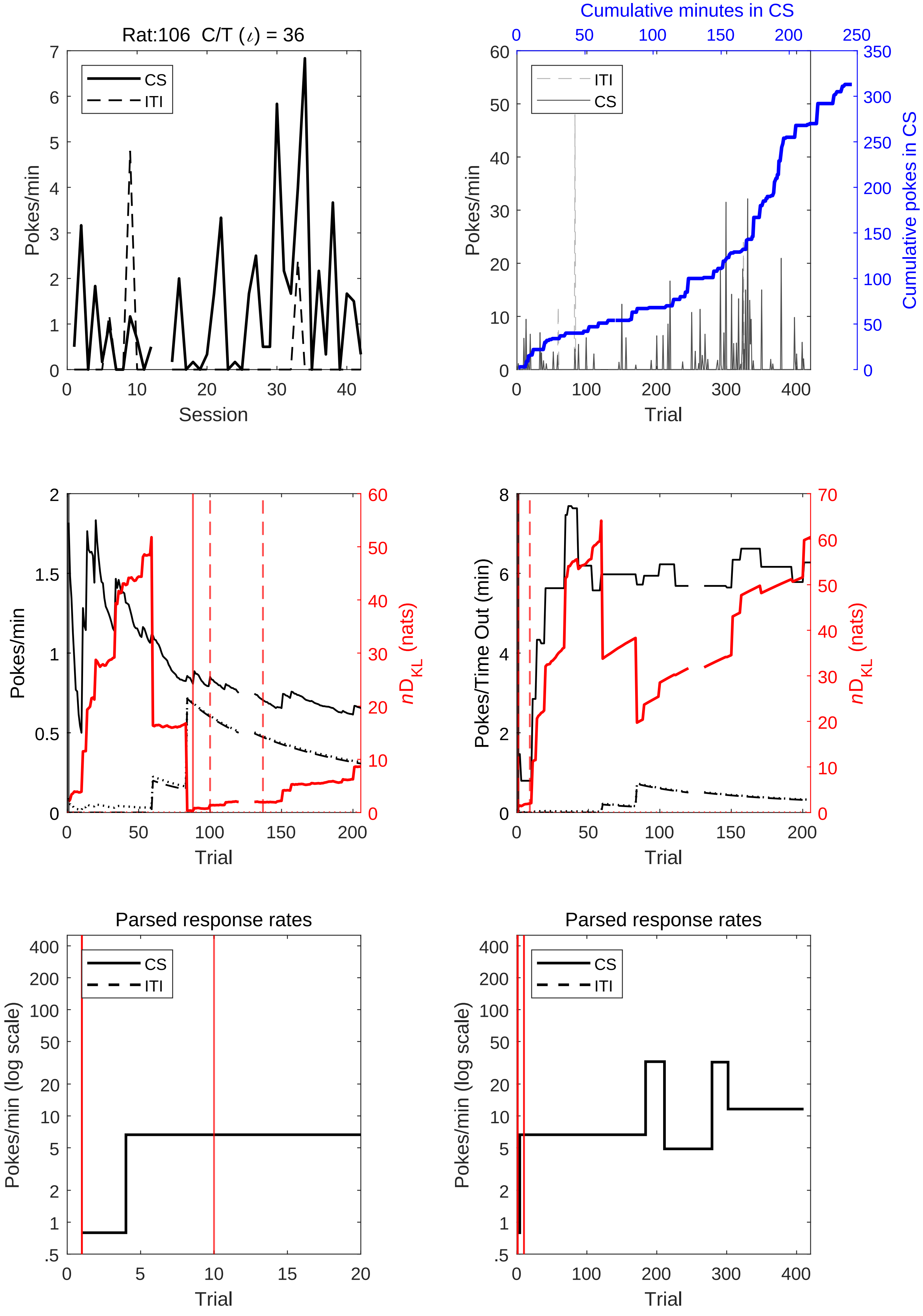
Rat:106 C/T (ι) = 36
CS
ITI
Pokes/min
Session
Cumulative minutes in CS
Cumulative pokes in CS
ITI
CS
Pokes/min
Trial
Pokes/min
$nD_{KL}$ (nats)
Trial
Pokes/Time Out (min)
$nD_{KL}$ (nats)
Trial
Parsed response rates
CS
ITI
Pokes/min (log scale)
Trial
Parsed response rates
CS
ITI
Pokes/min (log scale)
Trial

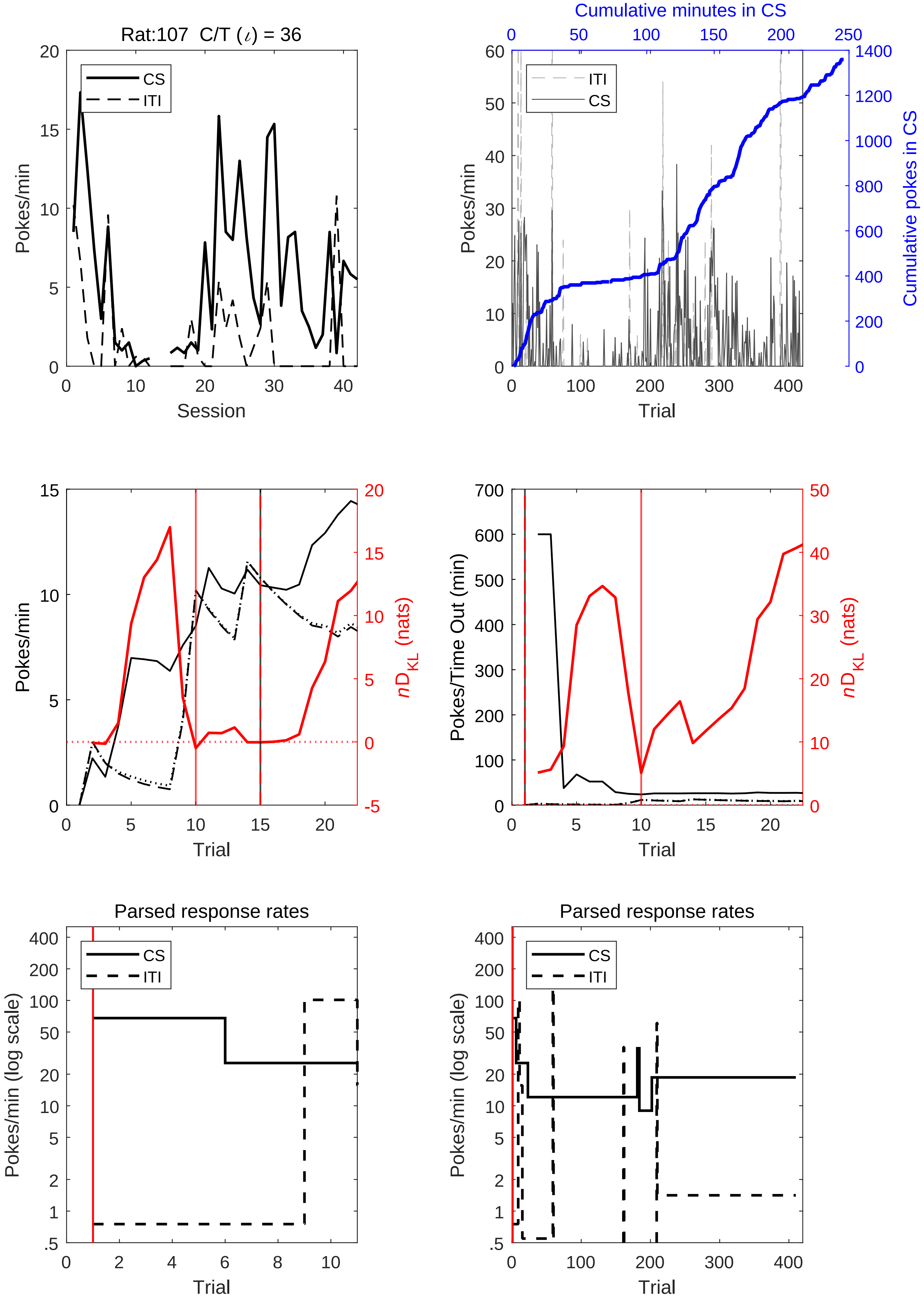
Rat:107 C/T (ι) = 36
CS
ITI
Pokes/min
Session
Cumulative minutes in CS
Cumulative pokes in CS
Trial
nD_KL (nats)
Pokes/Time Out (min)
Parsed response rates
Pokes/min (log scale)

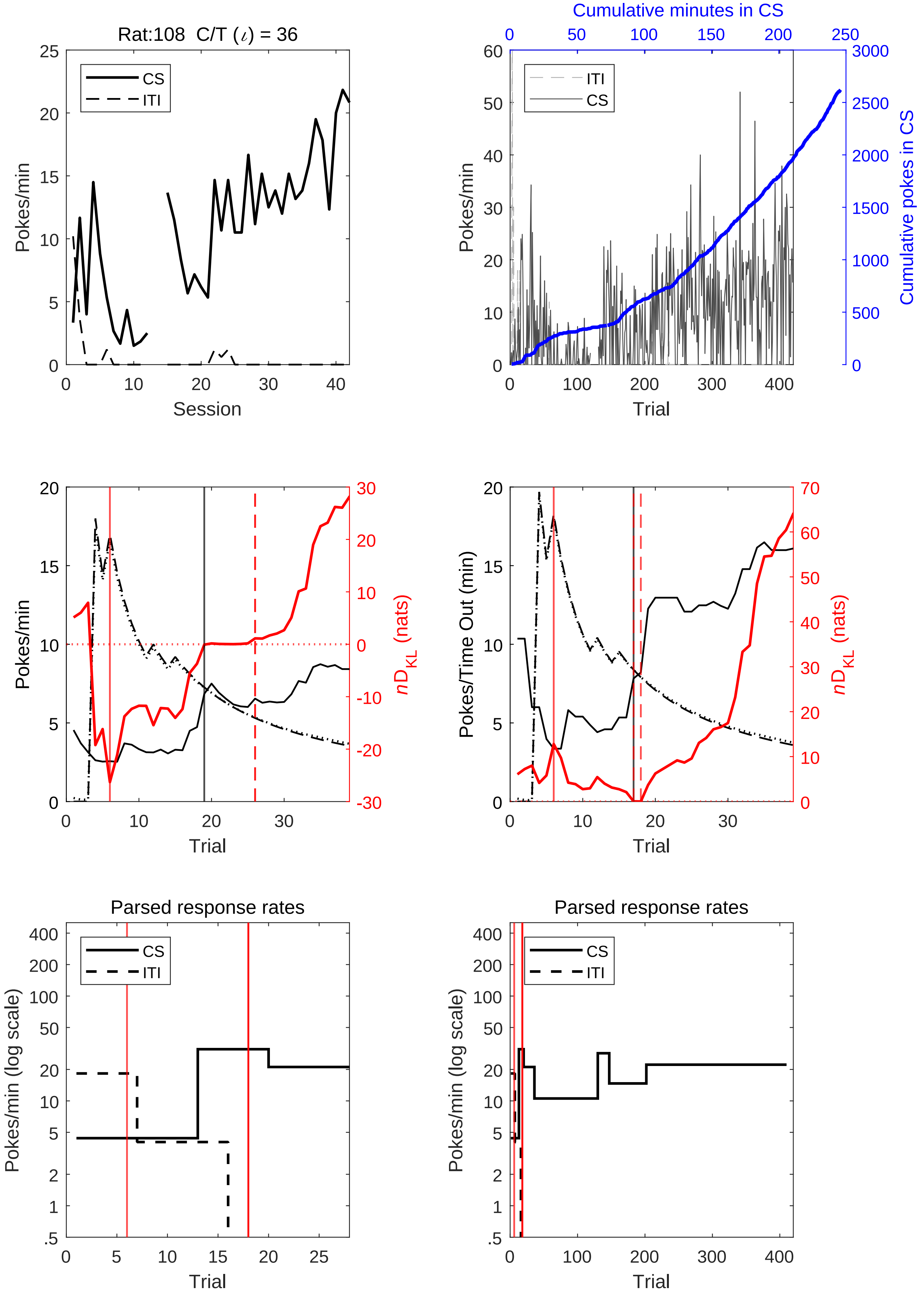
Rat:108 C/T (ι) = 36
CS
ITI
Pokes/min
Session
Cumulative minutes in CS
Cumulative pokes in CS
Trial
Pokes/Time Out (min)
nD_KL (nats)
Parsed response rates
Pokes/min (log scale)

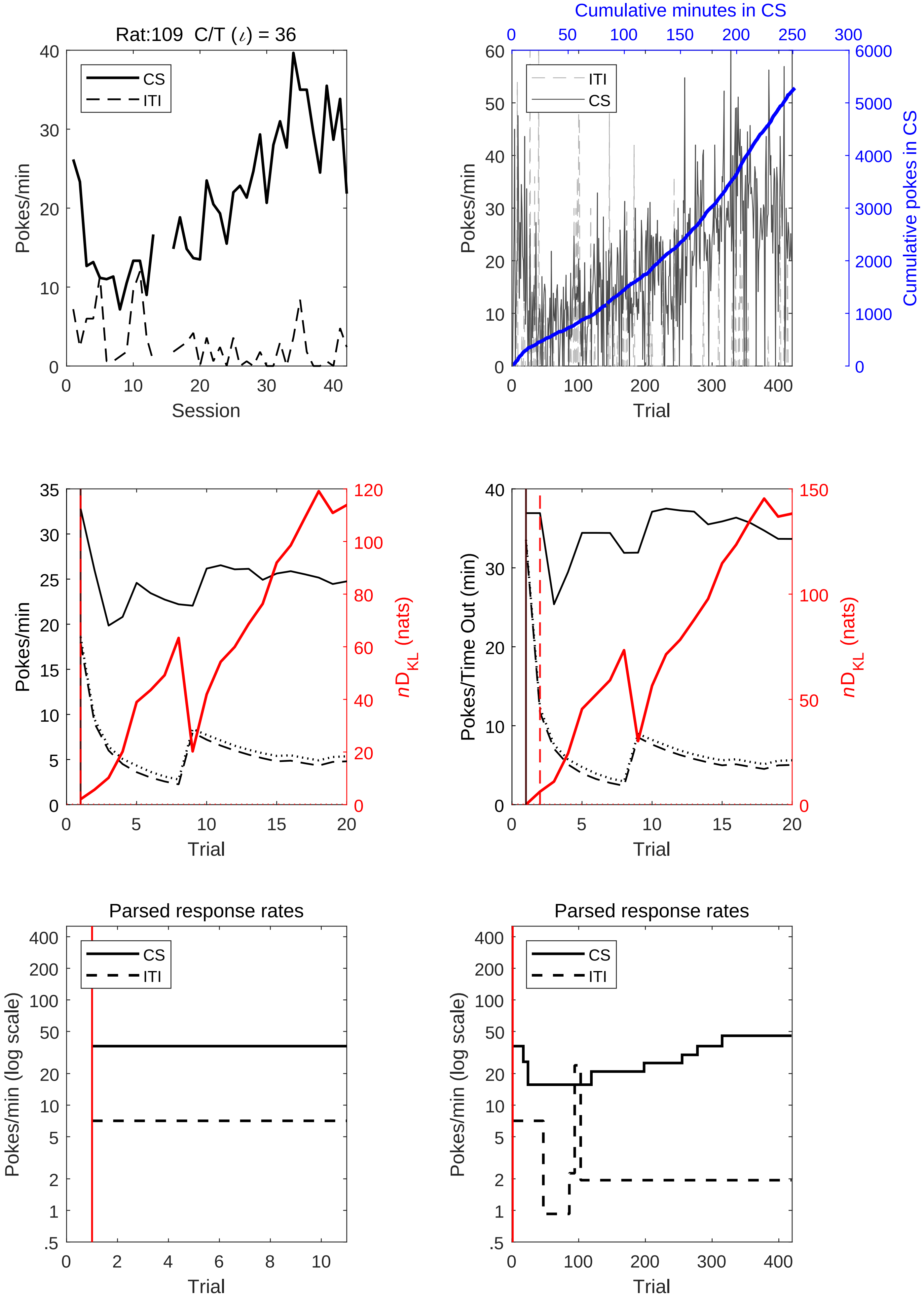

Rat:109 C/T (ι) = 36
CS
ITI
Pokes/min
Session
Cumulative minutes in CS
ITI
CS
Pokes/min
Cumulative pokes in CS
Trial
Pokes/min
nD_KL (nats)
Trial
Pokes/Time Out (min)
nD_KL (nats)
Trial
Parsed response rates
CS
ITI
Pokes/min (log scale)
Trial
Parsed response rates
CS
ITI
Pokes/min (log scale)
Trial

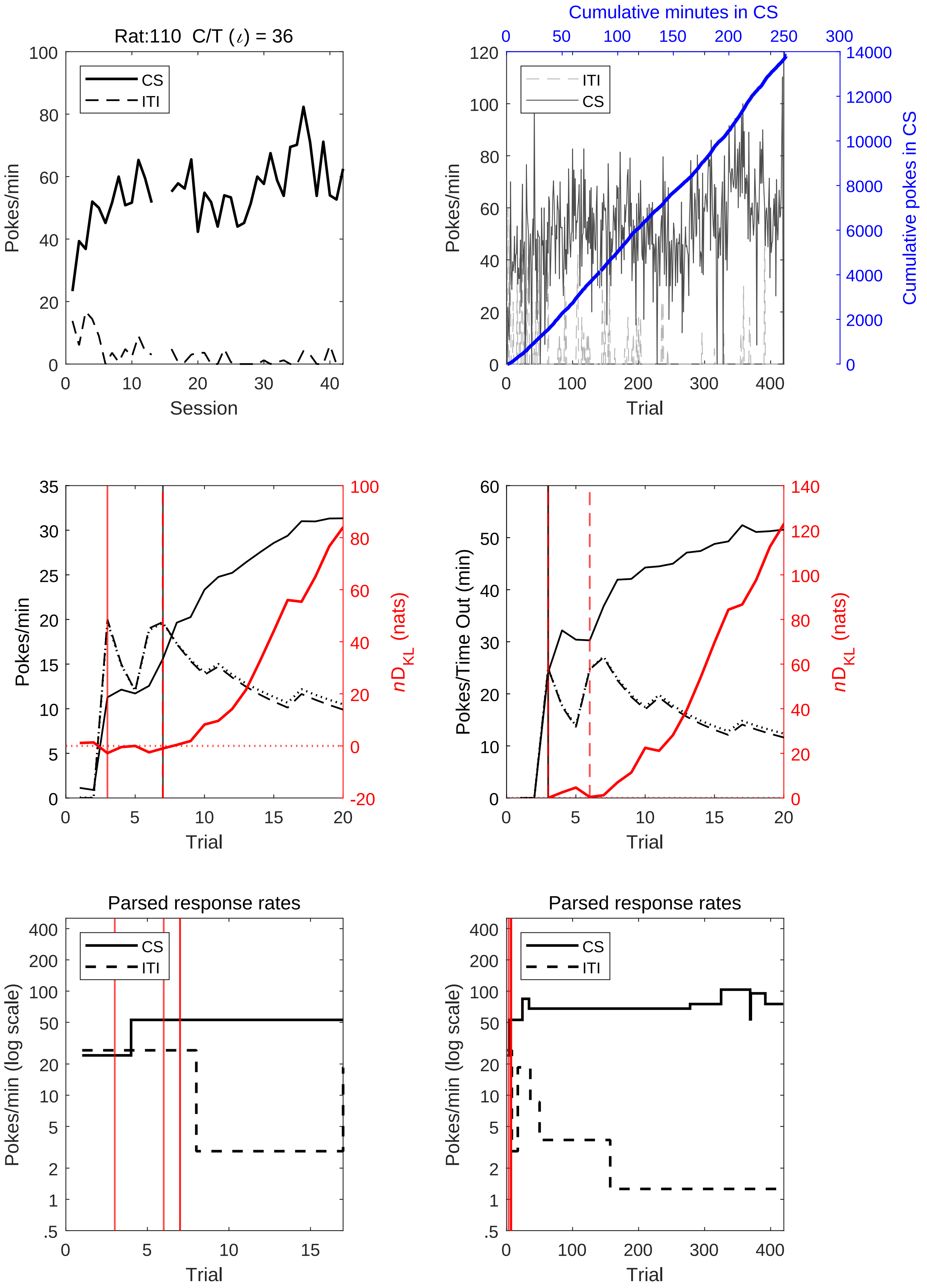
Rat:110 C/T (ι) = 36
CS
ITI
Pokes/min
Session
Cumulative minutes in CS
ITI
CS
Pokes/min
Cumulative pokes in CS
Trial
Pokes/min
nD_KL (nats)
Trial
Pokes/Time Out (min)
nD_KL (nats)
Trial
Parsed response rates
CS
ITI
Pokes/min (log scale)
Trial
Parsed response rates
CS
ITI
Pokes/min (log scale)
Trial

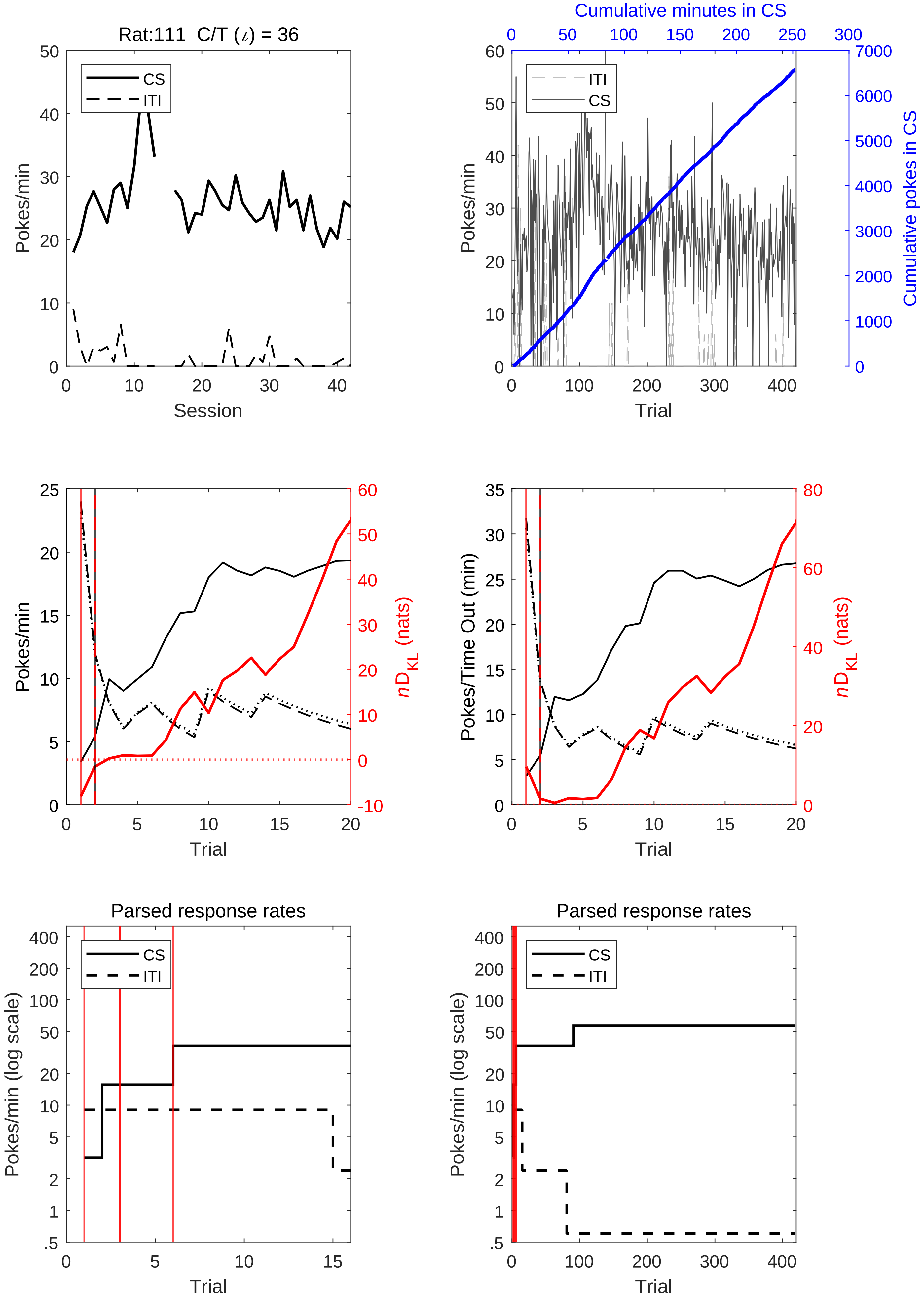
Rat:111 C/T (ι) = 36
CS
ITI
Pokes/min
Session
Cumulative minutes in CS
ITI
CS
Pokes/min
Cumulative pokes in CS
Trial
Pokes/min
$nD_{KL}$ (nats)
Trial
Pokes/Time Out (min)
$nD_{KL}$ (nats)
Trial
Parsed response rates
CS
ITI
Pokes/min (log scale)
Trial
Parsed response rates
CS
ITI
Pokes/min (log scale)
Trial

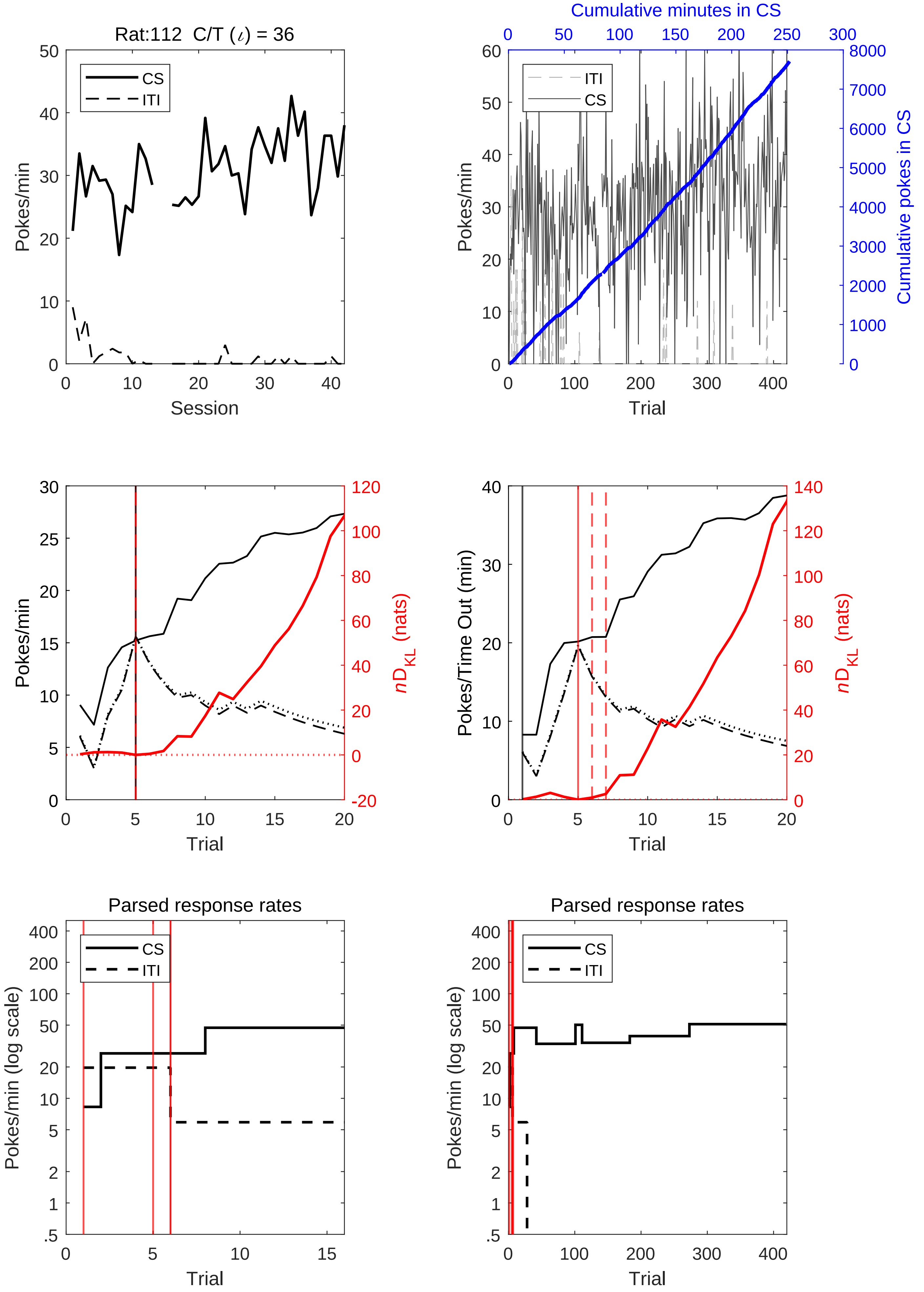
Rat:112 C/T ($\iota$) = 36
CS
ITI
Pokes/min
Session
Cumulative minutes in CS
Cumulative pokes in CS
Trial
nD_KL (nats)
Pokes/Time Out (min)
Parsed response rates
Pokes/min (log scale)

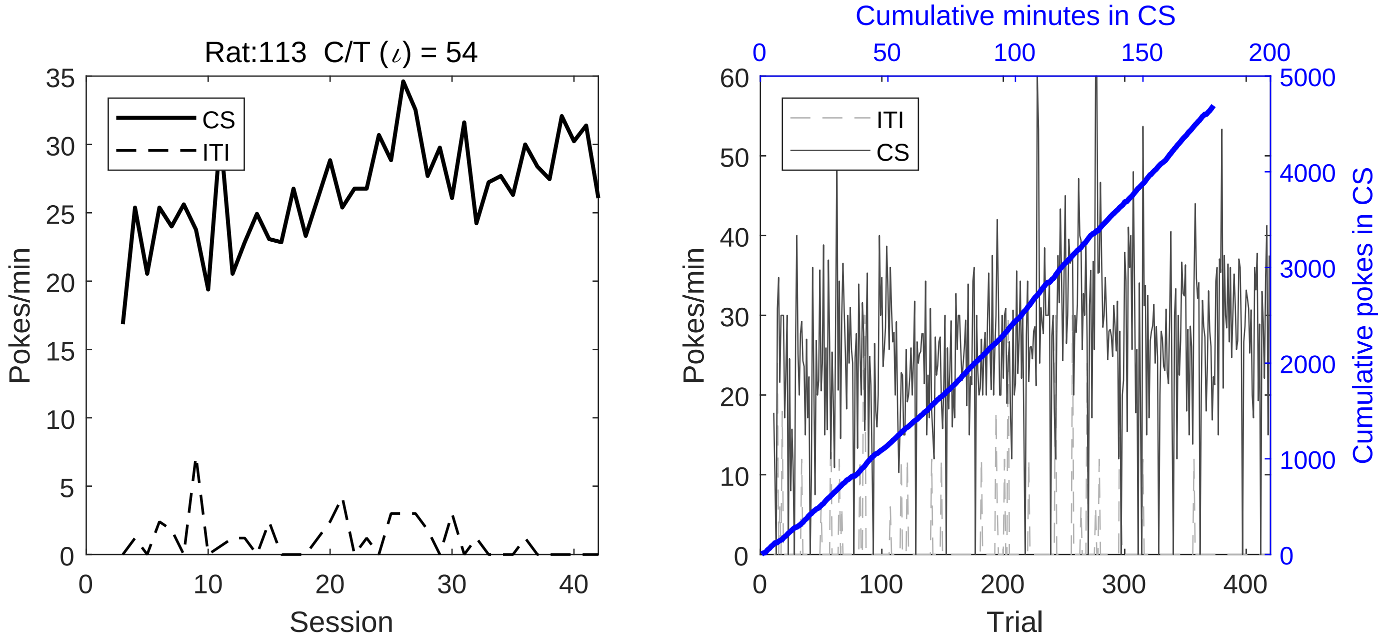

Rat:113 C/T (ι) = 54
CS
ITI
Pokes/min
Session
Cumulative minutes in CS
Pokes/min
Cumulative pokes in CS
Trial
ITI
CS

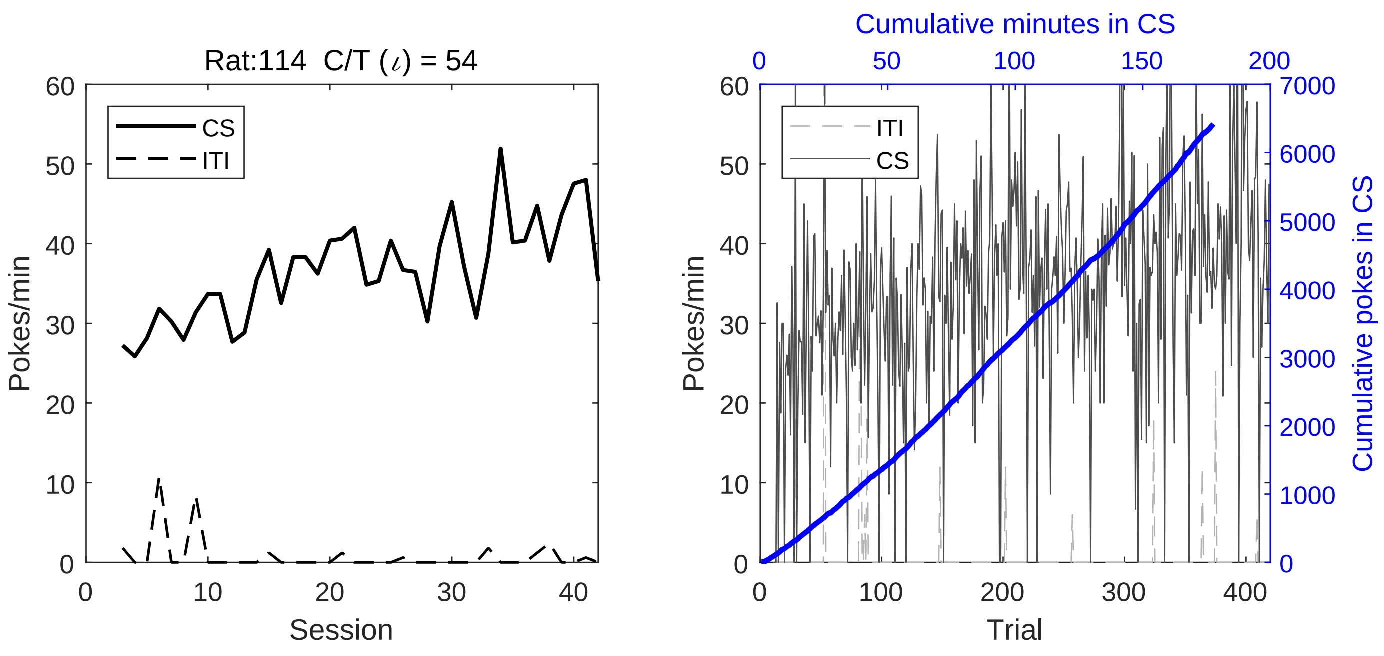
Rat:114 C/T ($\iota$) = 54
CS
ITI
Pokes/min
Session
Cumulative minutes in CS
ITI
CS
Pokes/min
Cumulative pokes in CS
Trial

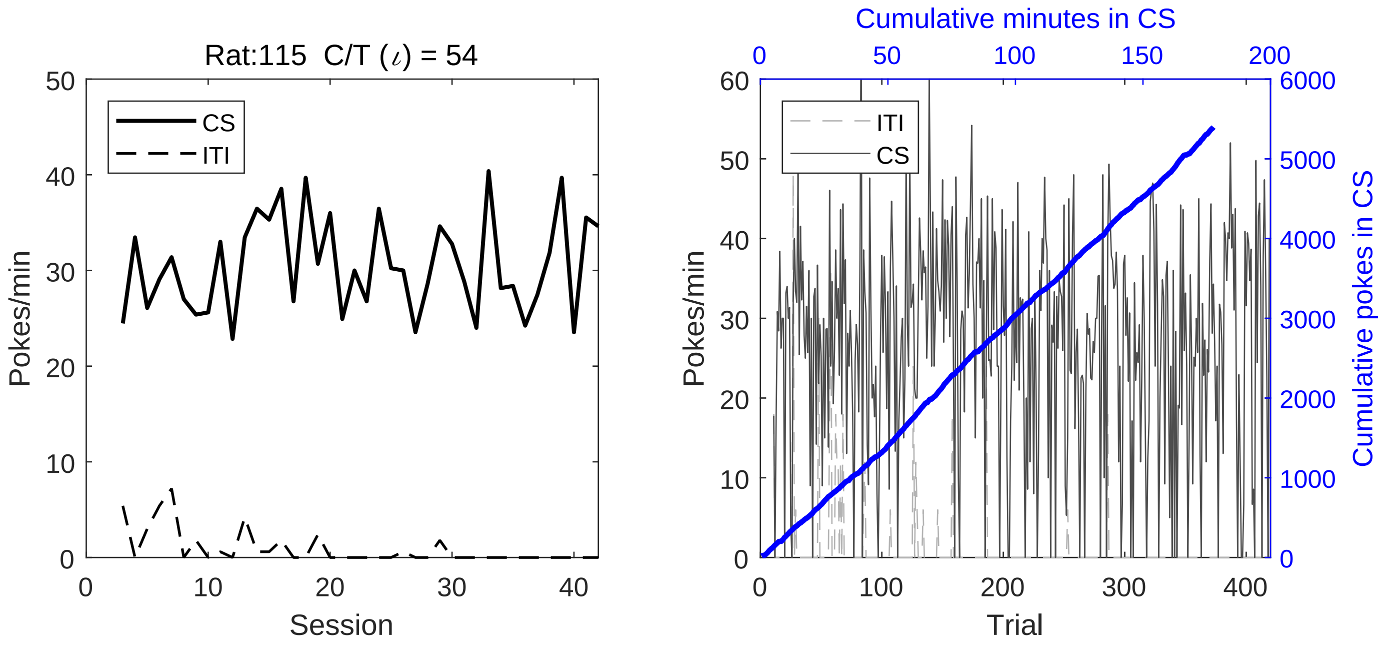
Rat:115 C/T (ι) = 54
CS
ITI
Pokes/min
Session
Cumulative minutes in CS
ITI
CS
Pokes/min
Trial
Cumulative pokes in CS

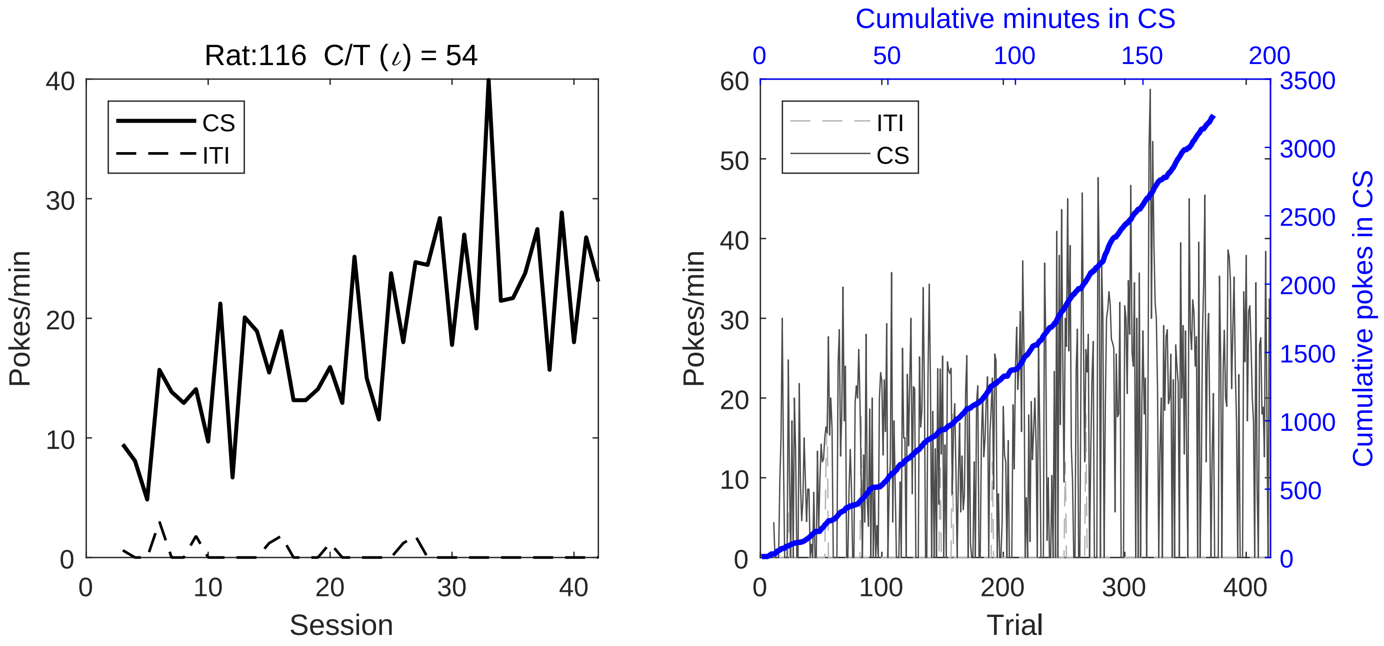
Rat:116  C/T (ι) = 54
CS
ITI
Pokes/min
Session
0
10
20
30
40
Cumulative minutes in CS
ITI
CS
Pokes/min
Trial
Cumulative pokes in CS
0
50
100
150
200
500
1000
1500
2000
2500
3000
3500
60
300
400

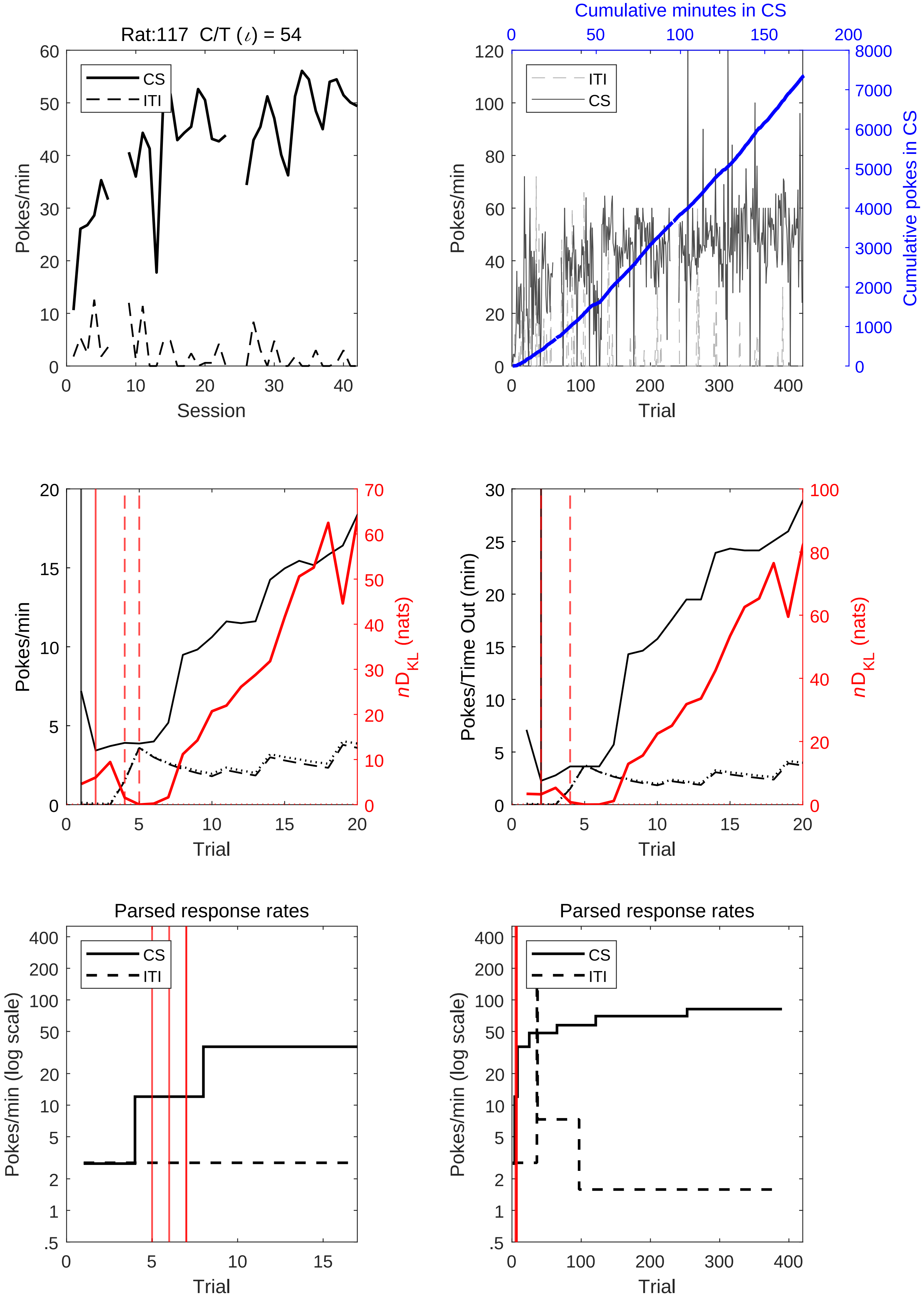
Rat:117 C/T (ι) = 54
CS
ITI
Pokes/min
Session
Cumulative minutes in CS
Cumulative pokes in CS
Trial
Pokes/min
nD_KL (nats)
Trial
Pokes/Time Out (min)
nD_KL (nats)
Trial
Parsed response rates
Pokes/min (log scale)
Trial
Parsed response rates
Pokes/min (log scale)
Trial

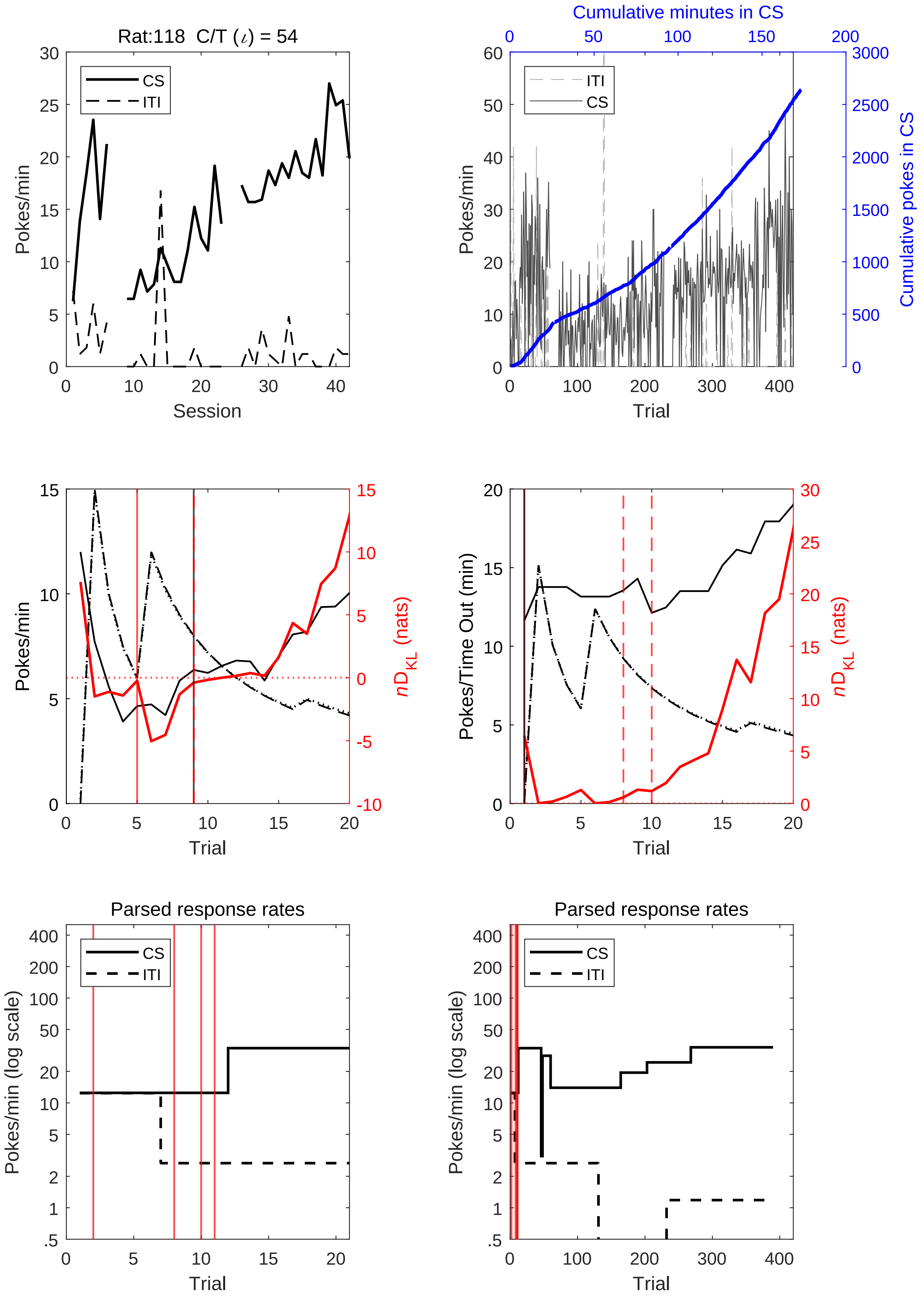
Rat:118 C/T (ι) = 54
Pokes/min
Session
CS
ITI
Cumulative minutes in CS
Cumulative pokes in CS
Trial
Pokes/Time Out (min)
nD_KL (nats)
Parsed response rates
Pokes/min (log scale)

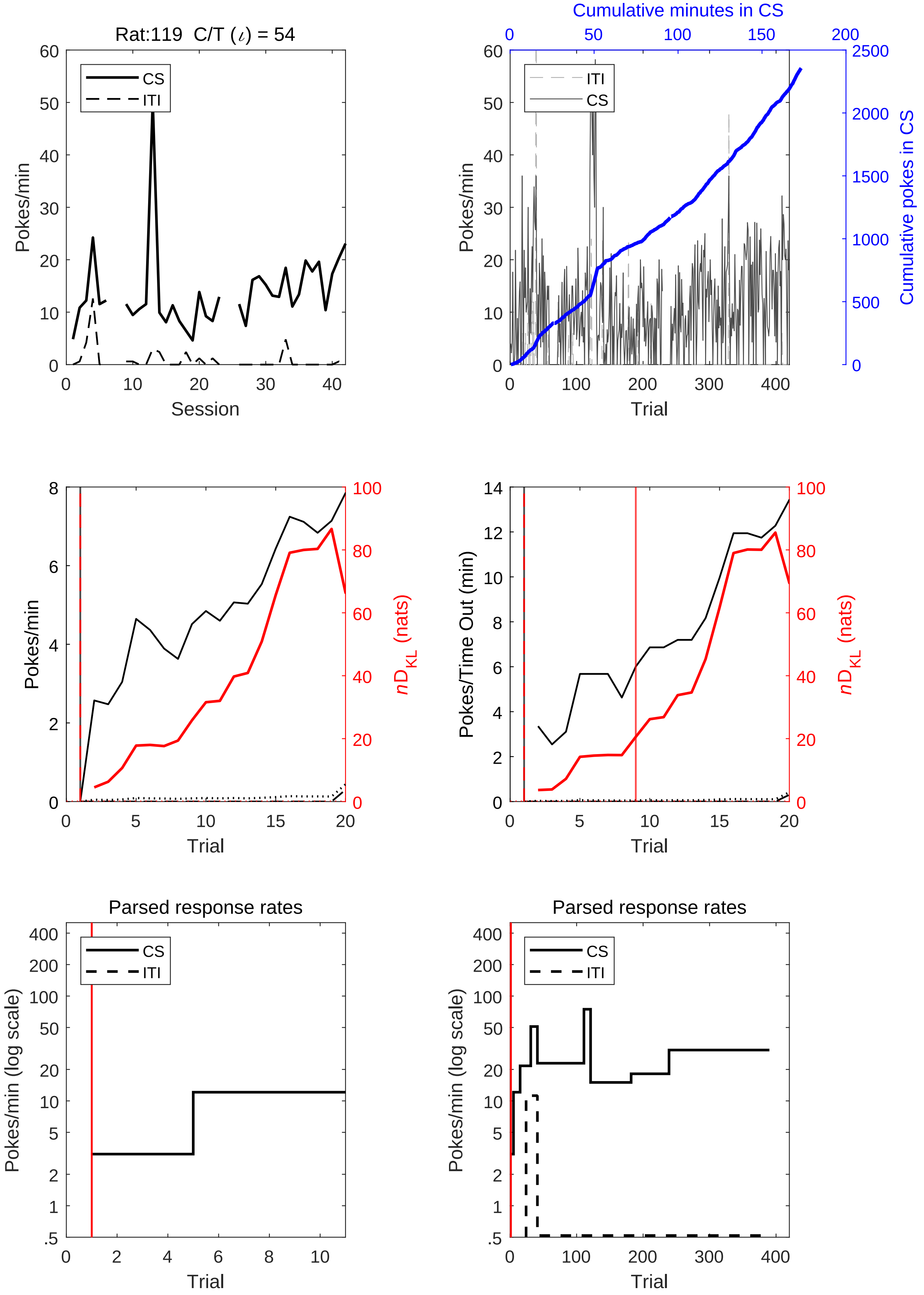

Rat:119 C/T ($\iota$) = 54
CS
ITI
Pokes/min
Session
Cumulative minutes in CS
Cumulative pokes in CS
Trial
$nD_{KL}$ (nats)
Pokes/Time Out (min)
Parsed response rates
Pokes/min (log scale)

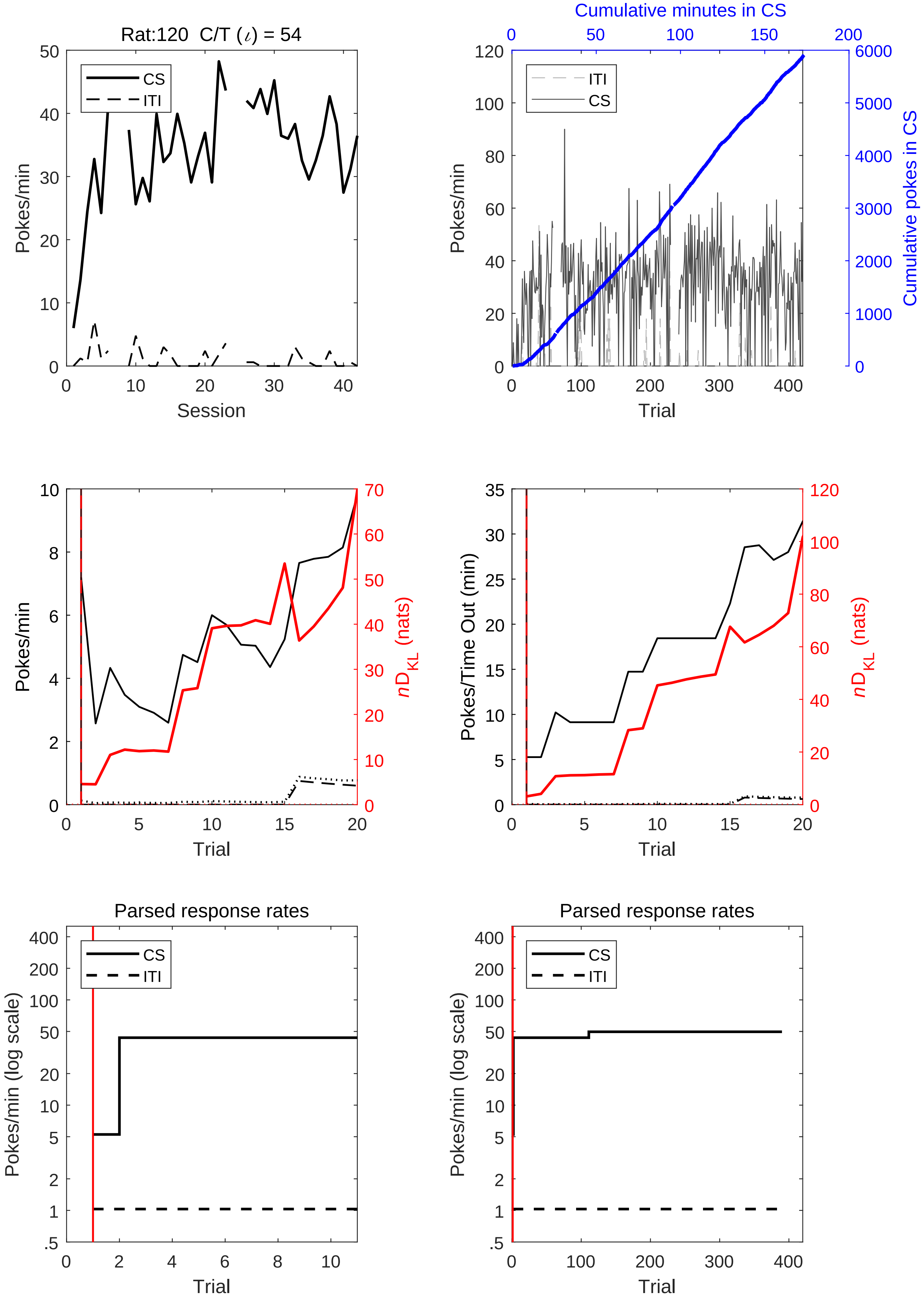

Rat:120 C/T (ι) = 54
CS
ITI
Pokes/min
Session
Cumulative minutes in CS
Cumulative pokes in CS
Trial
nD_KL (nats)
Pokes/Time Out (min)
Parsed response rates
Pokes/min (log scale)

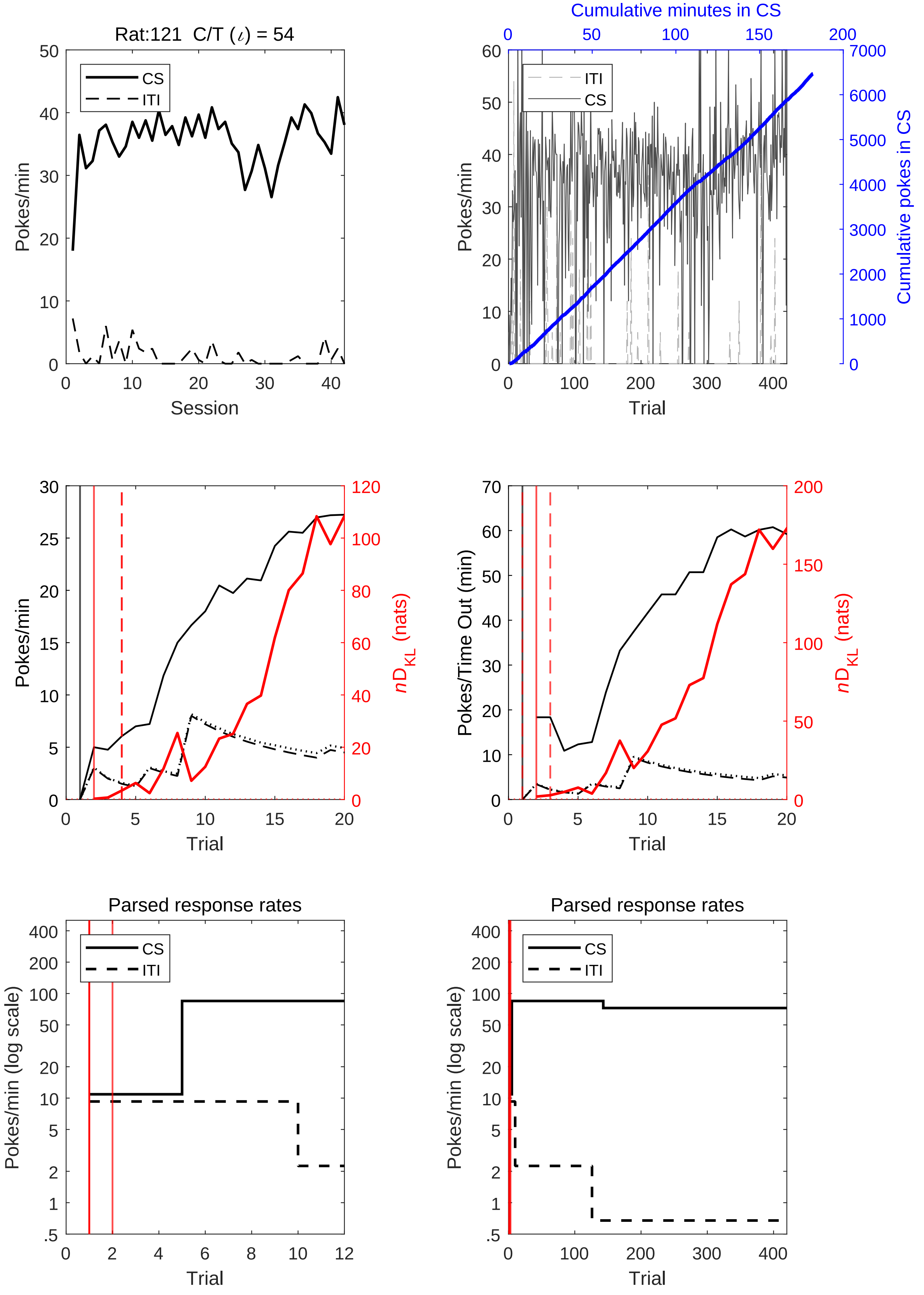
Rat:121 C/T (ι) = 54
CS
ITI
Pokes/min
Session
Cumulative minutes in CS
Cumulative pokes in CS
Trial
nD_KL (nats)
Pokes/Time Out (min)
Parsed response rates
Pokes/min (log scale)

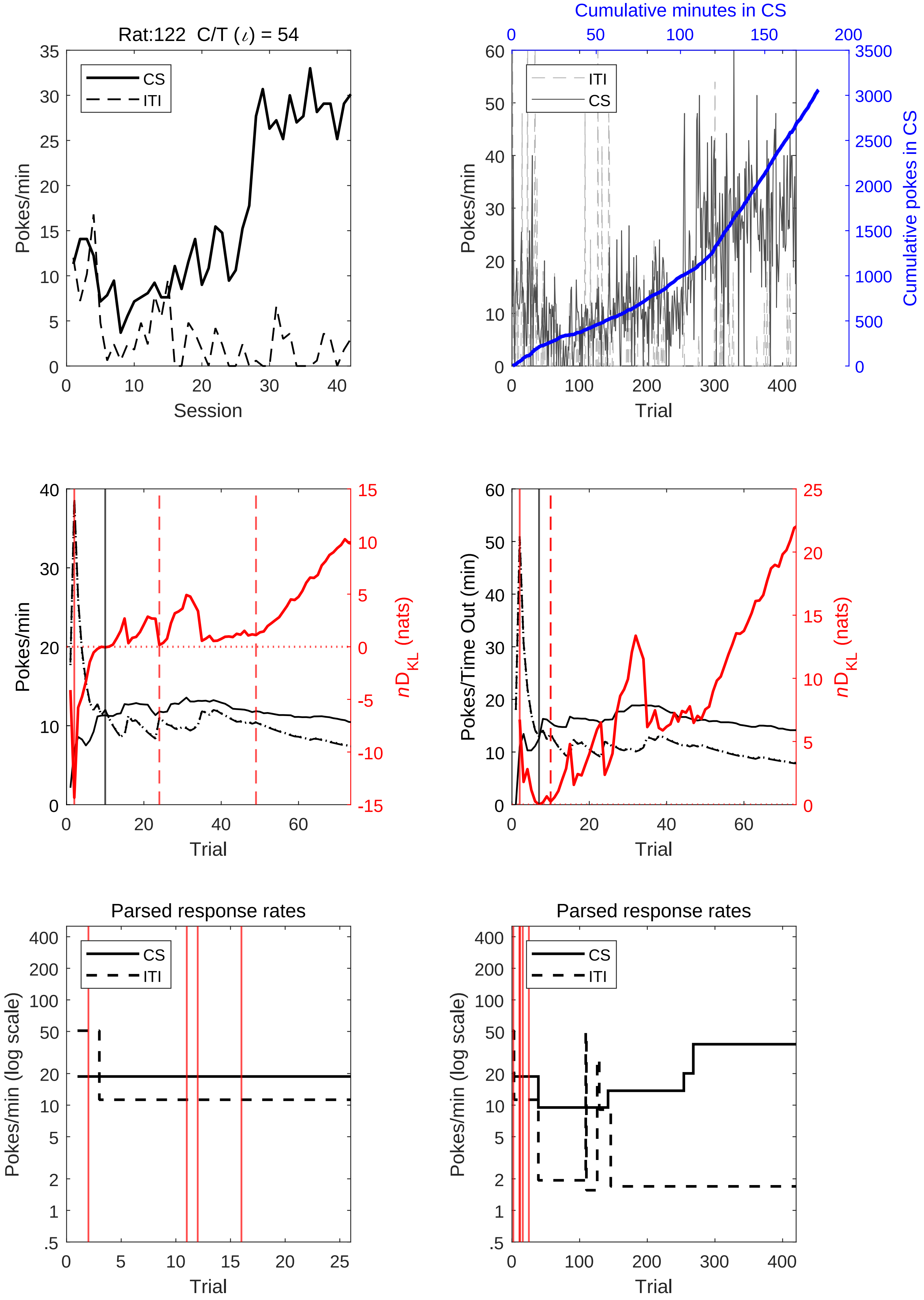
Rat:122 C/T ($\iota$) = 54
CS
ITI
Pokes/min
Session
Cumulative minutes in CS
Cumulative pokes in CS
Trial
$nD_{KL}$ (nats)
Pokes/Time Out (min)
Parsed response rates
Pokes/min (log scale)

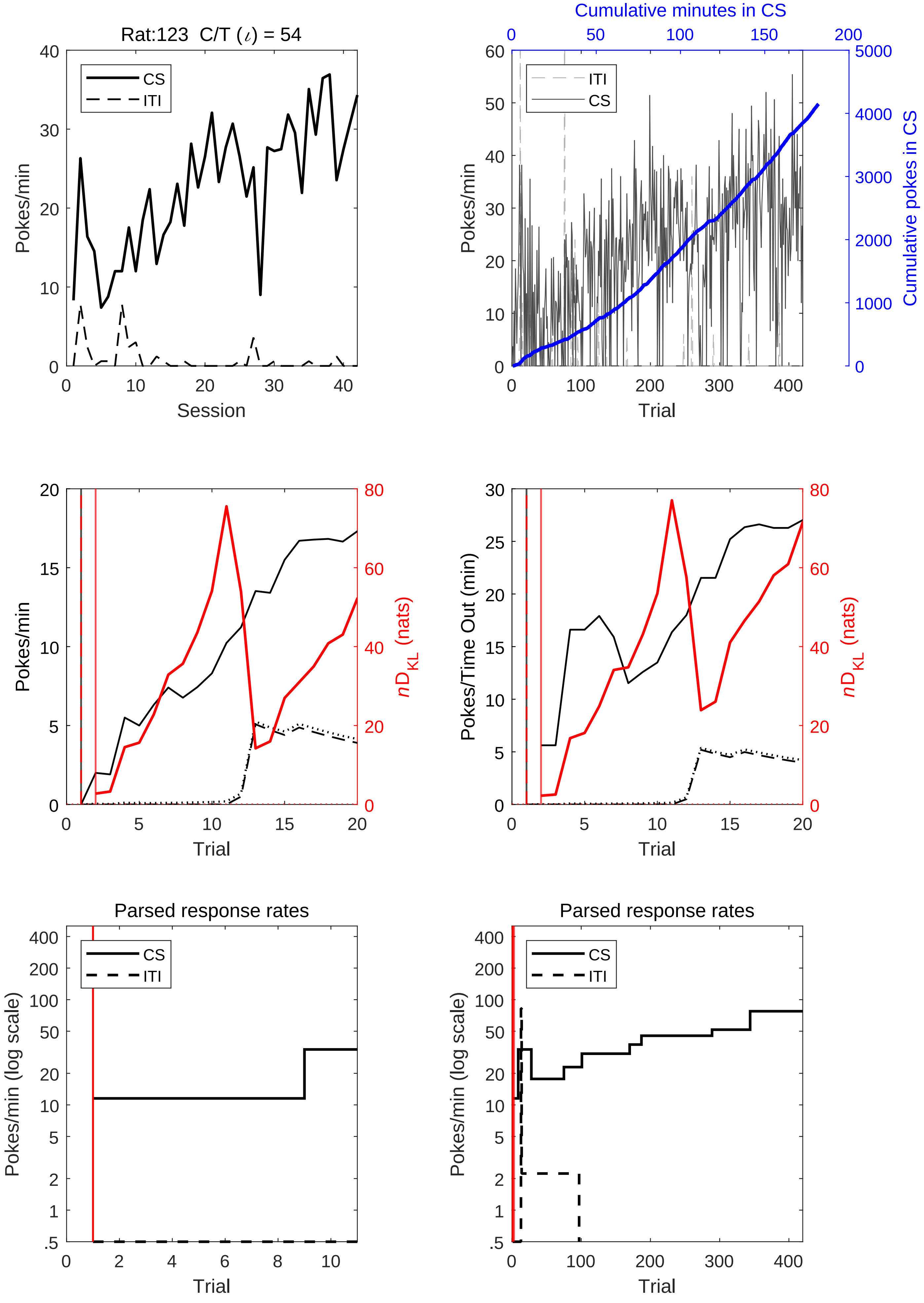

Rat:123 C/T (ι) = 54
Pokes/min
Session
CS
ITI
Cumulative minutes in CS
Cumulative pokes in CS
Trial
Pokes/Time Out (min)
$nD_{KL}$ (nats)
Parsed response rates
Pokes/min (log scale)

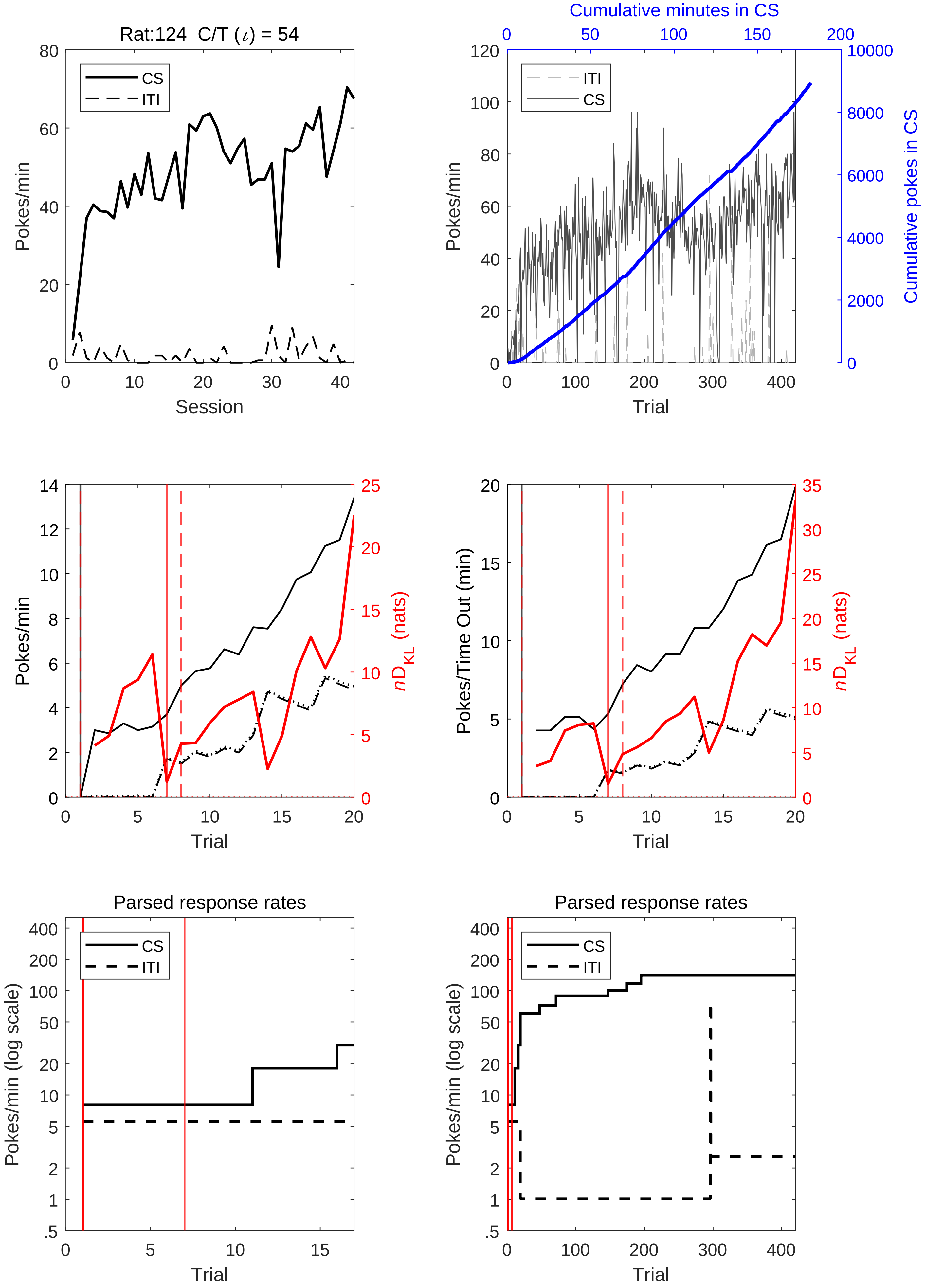

Rat:124 C/T (ι) = 54
CS
ITI
Pokes/min
Session
Cumulative minutes in CS
Cumulative pokes in CS
Trial
Pokes/Time Out (min)
nD_KL (nats)
Parsed response rates
Pokes/min (log scale)

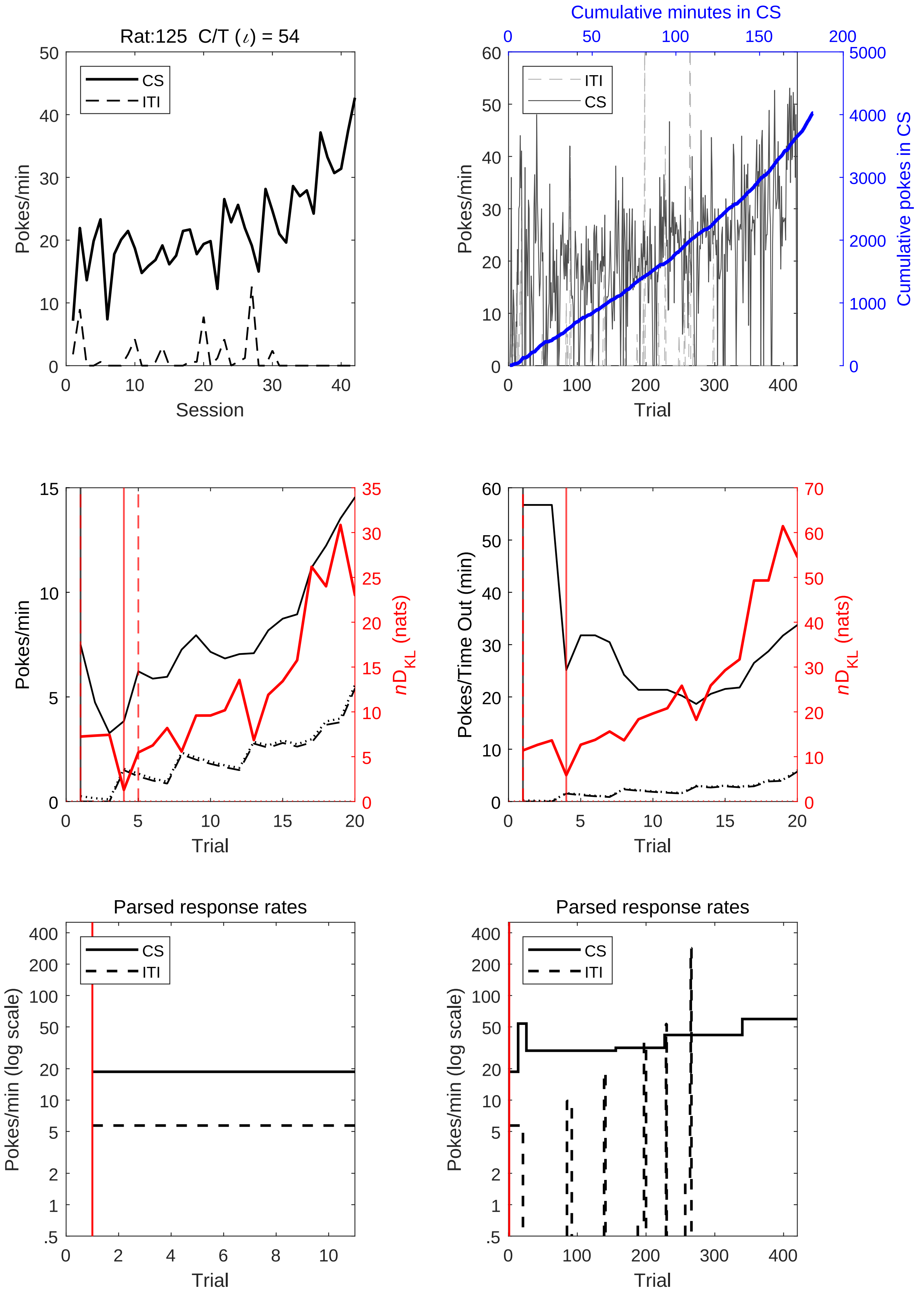

Rat:125 C/T ($\iota$) = 54
CS
ITI
Pokes/min
Session
Cumulative minutes in CS
Cumulative pokes in CS
Trial
Pokes/min
$nD_{KL}$ (nats)
Pokes/Time Out (min)
Parsed response rates
Pokes/min (log scale)

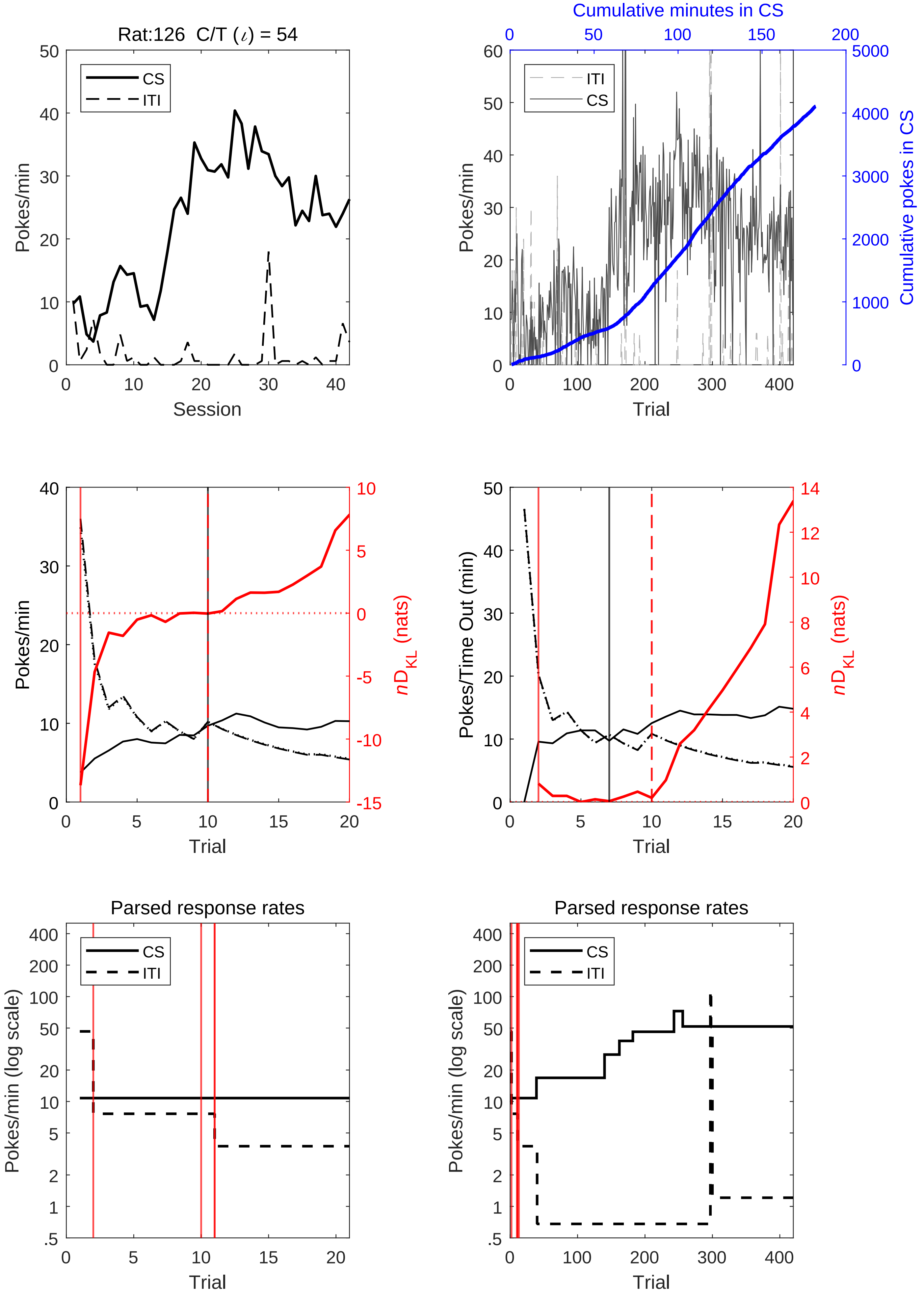

Rat:126 C/T (ι) = 54
CS
ITI
Pokes/min
Session
Cumulative minutes in CS
Cumulative pokes in CS
Trial
nD_KL (nats)
Pokes/Time Out (min)
Parsed response rates
Pokes/min (log scale)

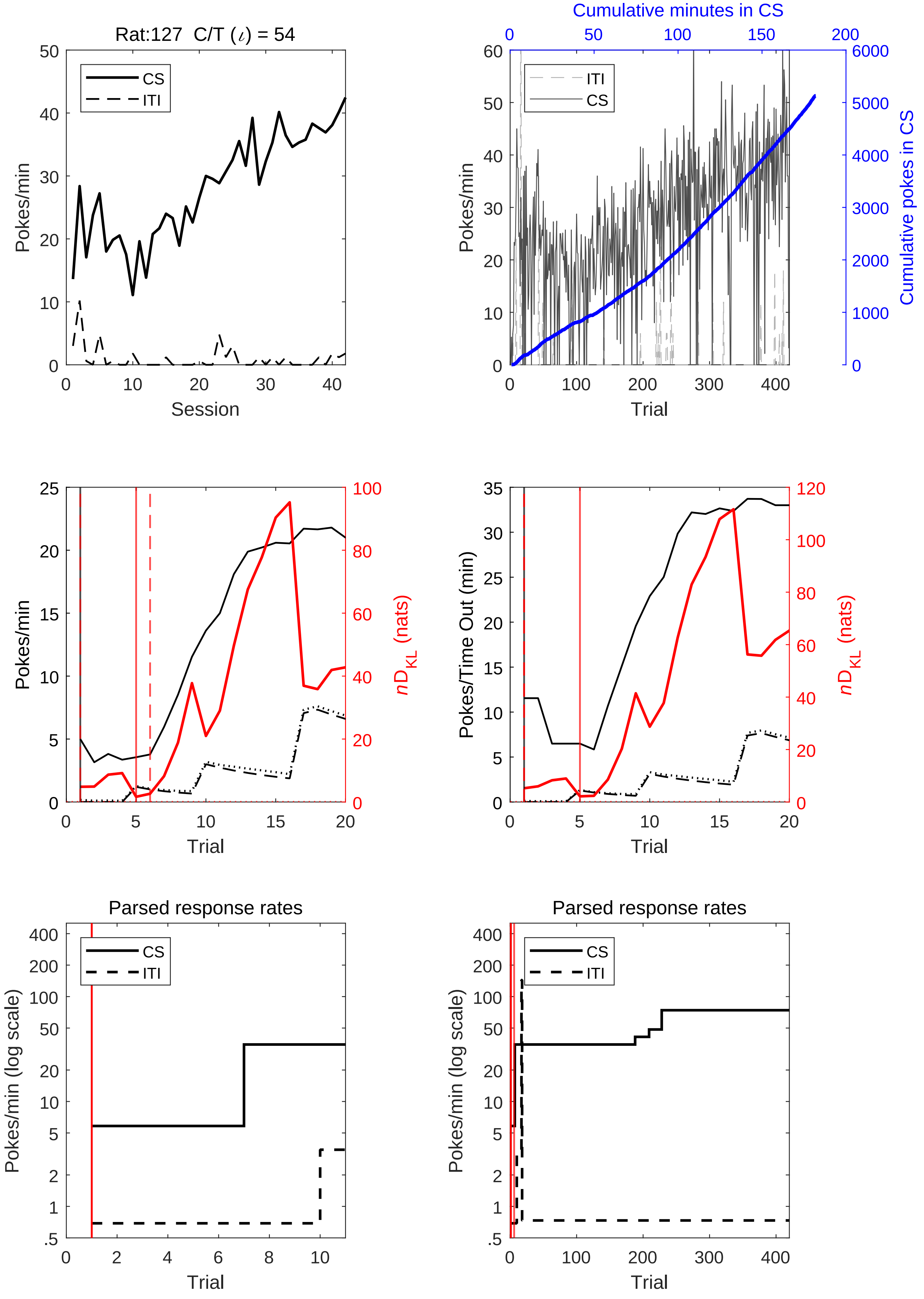

Rat:127 C/T (ι) = 54
CS
ITI
Pokes/min
Session
Cumulative minutes in CS
Cumulative pokes in CS
Trial
nD_KL (nats)
Pokes/Time Out (min)
Parsed response rates
Pokes/min (log scale)

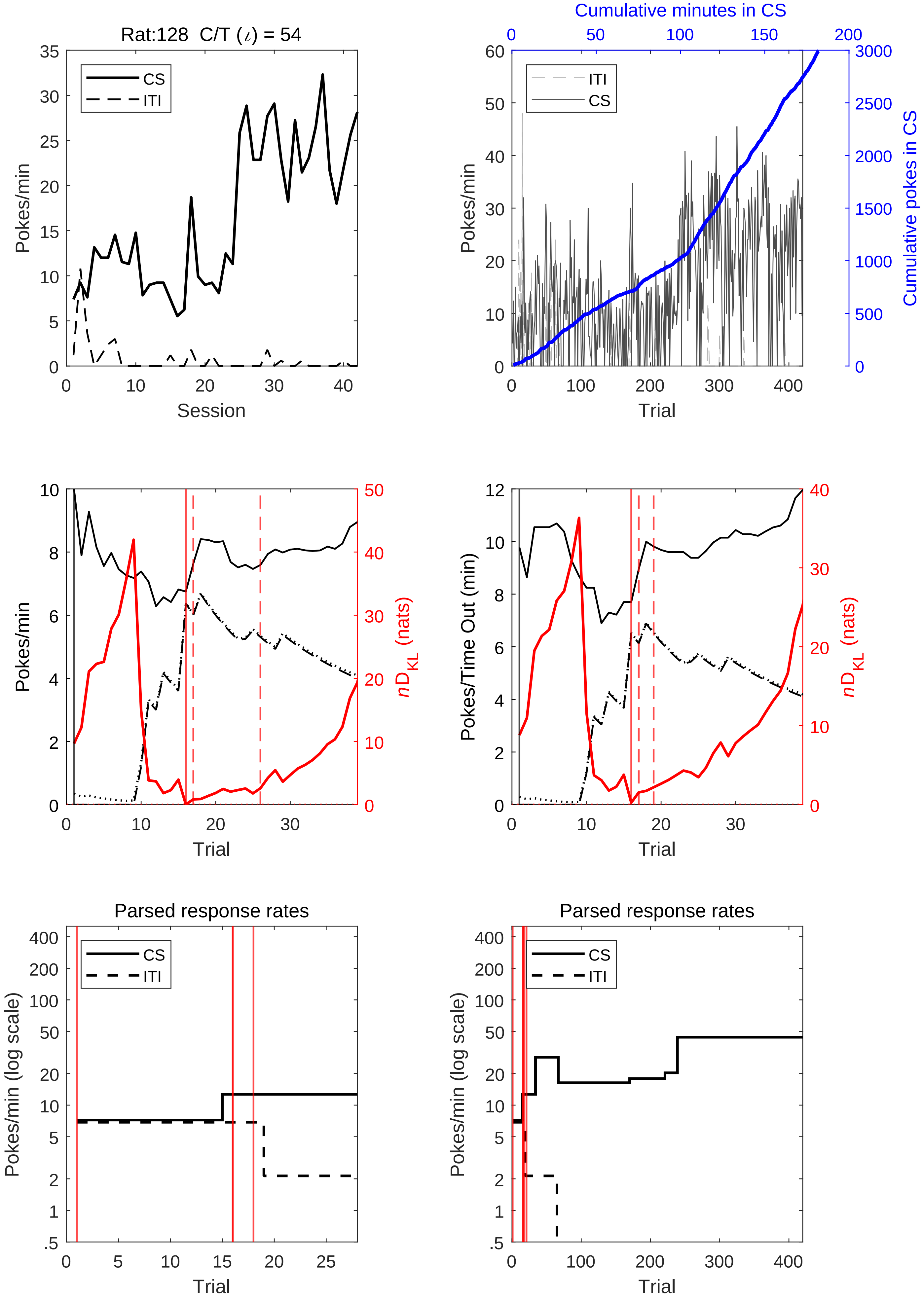
Rat:128 C/T (ι) = 54
CS
ITI
Pokes/min
Session
Cumulative minutes in CS
Cumulative pokes in CS
Trial
Pokes/Time Out (min)
$nD_{KL}$ (nats)
Parsed response rates
Pokes/min (log scale)

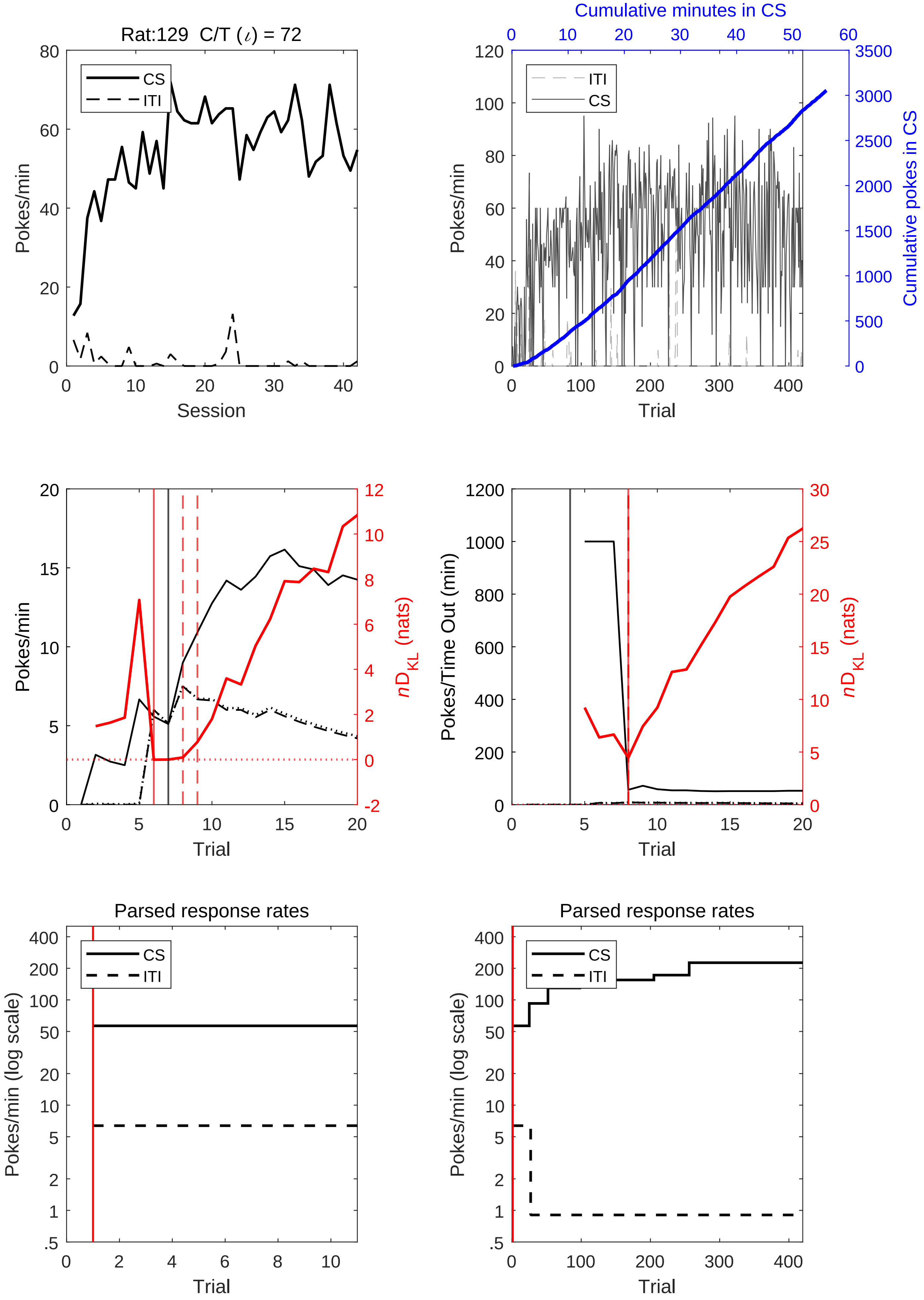

Rat:129 C/T (ι) = 72
CS
ITI
Pokes/min
Session
Cumulative minutes in CS
Cumulative pokes in CS
Pokes/min
Trial
Pokes/min
Trial
nD_KL (nats)
Pokes/Time Out (min)
Trial
nD_KL (nats)
Parsed response rates
Pokes/min (log scale)
Trial
Parsed response rates
Pokes/min (log scale)
Trial

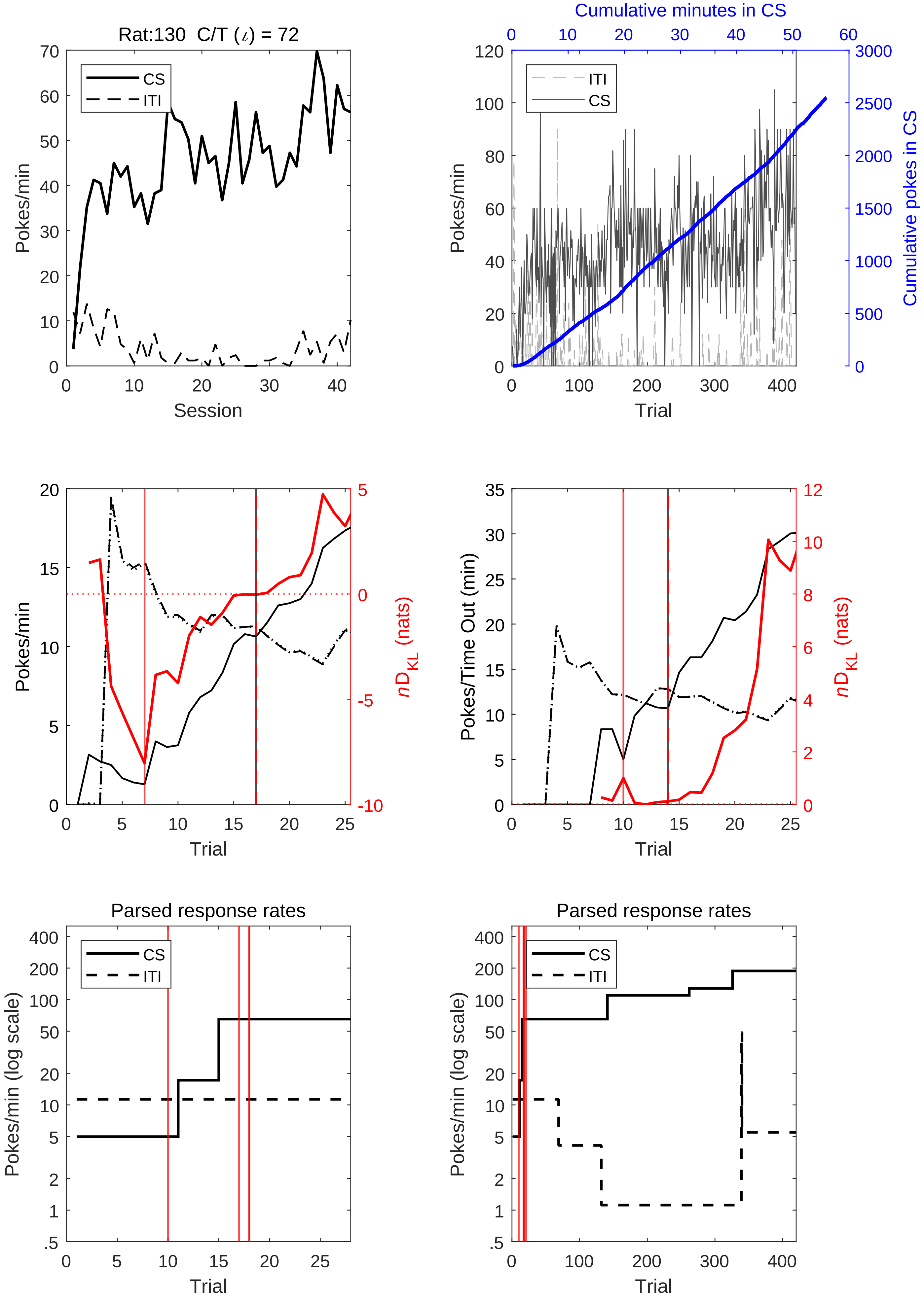

Rat:130 C/T (ι) = 72
CS
ITI
Pokes/min
Session
Cumulative minutes in CS
ITI
CS
Pokes/min
Cumulative pokes in CS
Trial
Pokes/min
nD_KL (nats)
Trial
Pokes/Time Out (min)
nD_KL (nats)
Trial
Parsed response rates
CS
ITI
Pokes/min (log scale)
Trial
Parsed response rates
CS
ITI
Pokes/min (log scale)
Trial

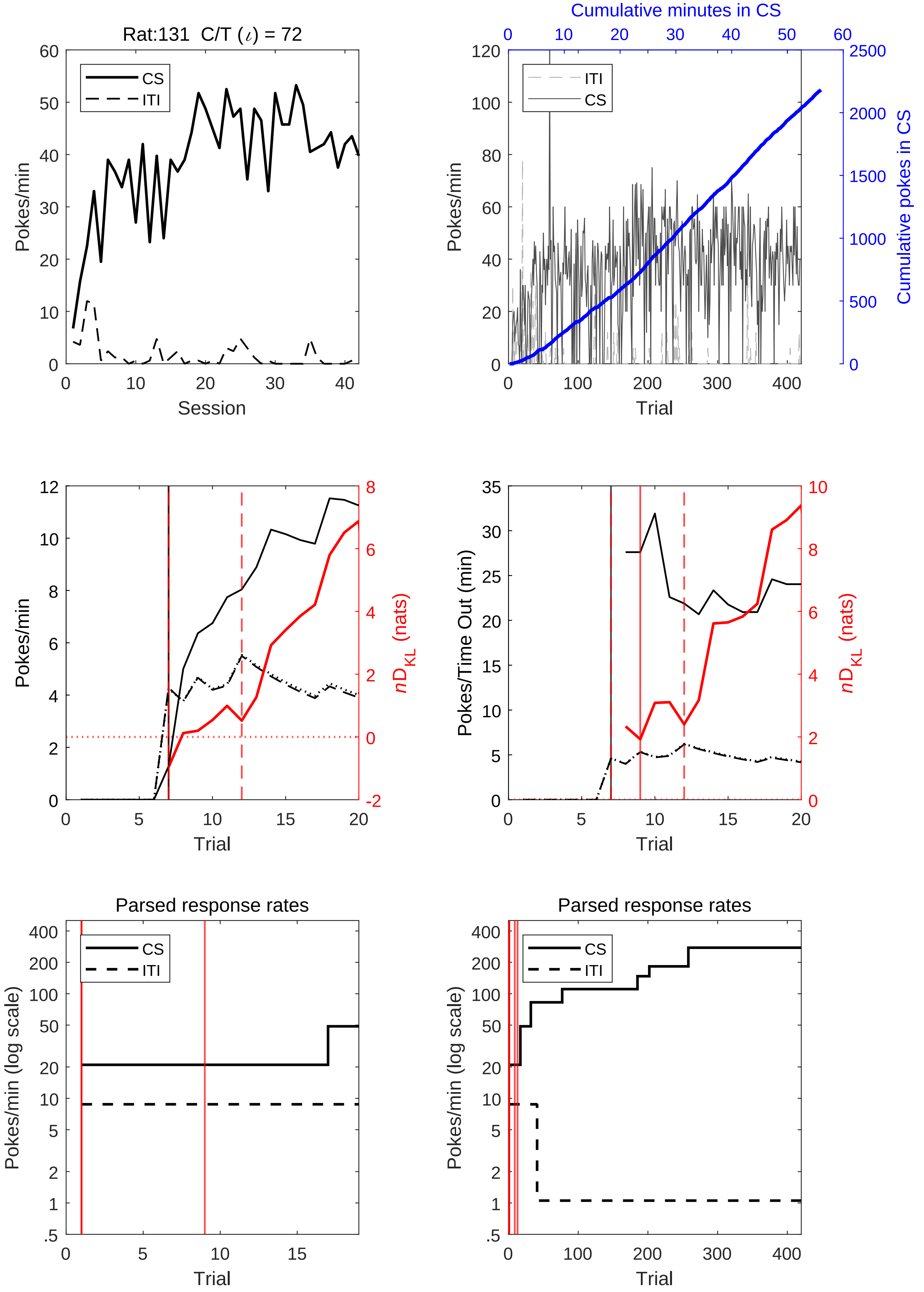

Rat:131 C/T (ι) = 72
CS
ITI
Pokes/min
Session
Cumulative minutes in CS
Cumulative pokes in CS
Trial
nD_KL (nats)
Pokes/Time Out (min)
Parsed response rates
Pokes/min (log scale)

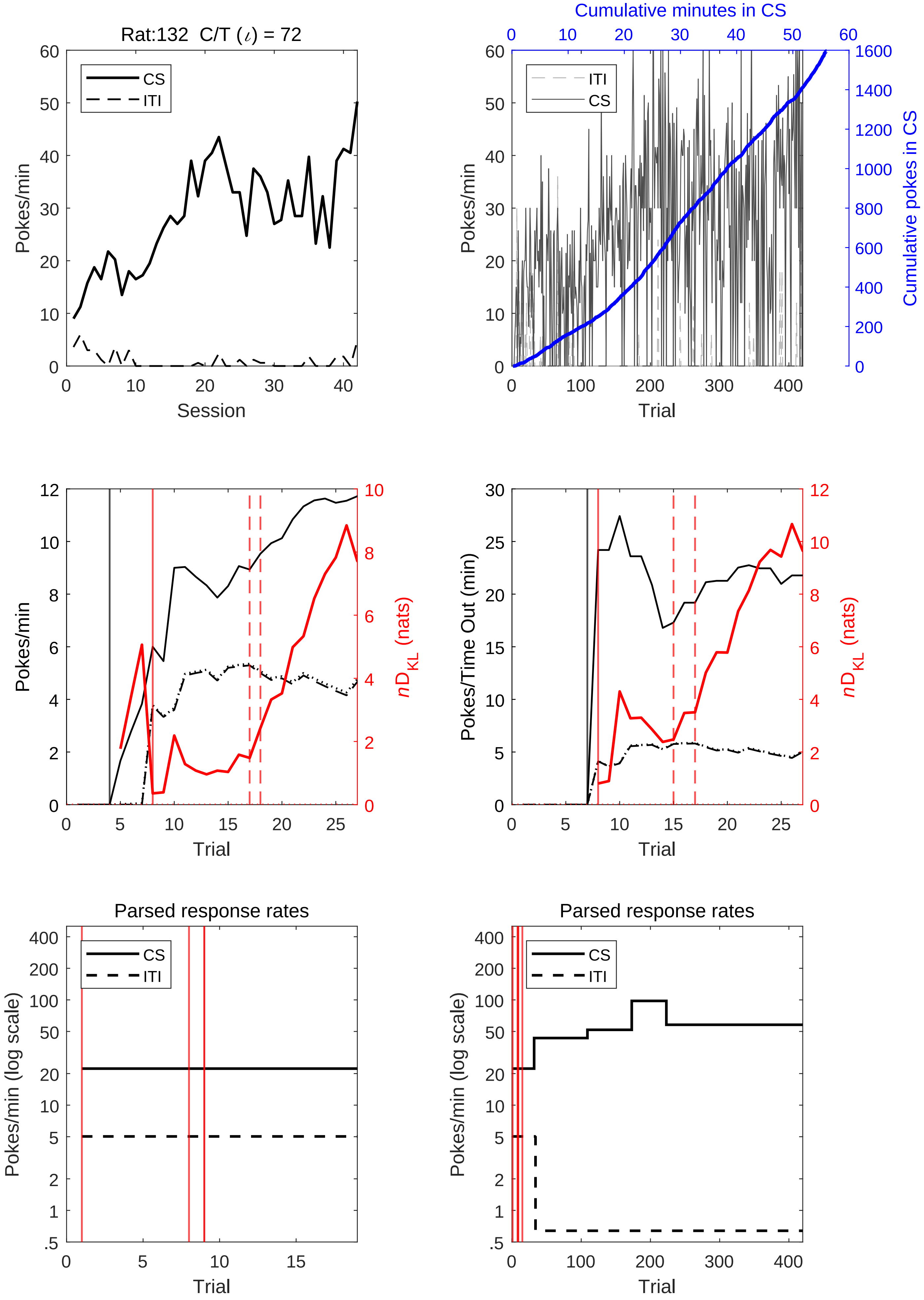
Rat:132 C/T (ι) = 72
CS
ITI
Pokes/min
Session
Cumulative minutes in CS
ITI
CS
Pokes/min
Cumulative pokes in CS
Trial
Pokes/min
$nD_{KL}$ (nats)
Trial
Pokes/Time Out (min)
$nD_{KL}$ (nats)
Trial
Parsed response rates
CS
ITI
Pokes/min (log scale)
Trial
Parsed response rates
CS
ITI
Pokes/min (log scale)
Trial

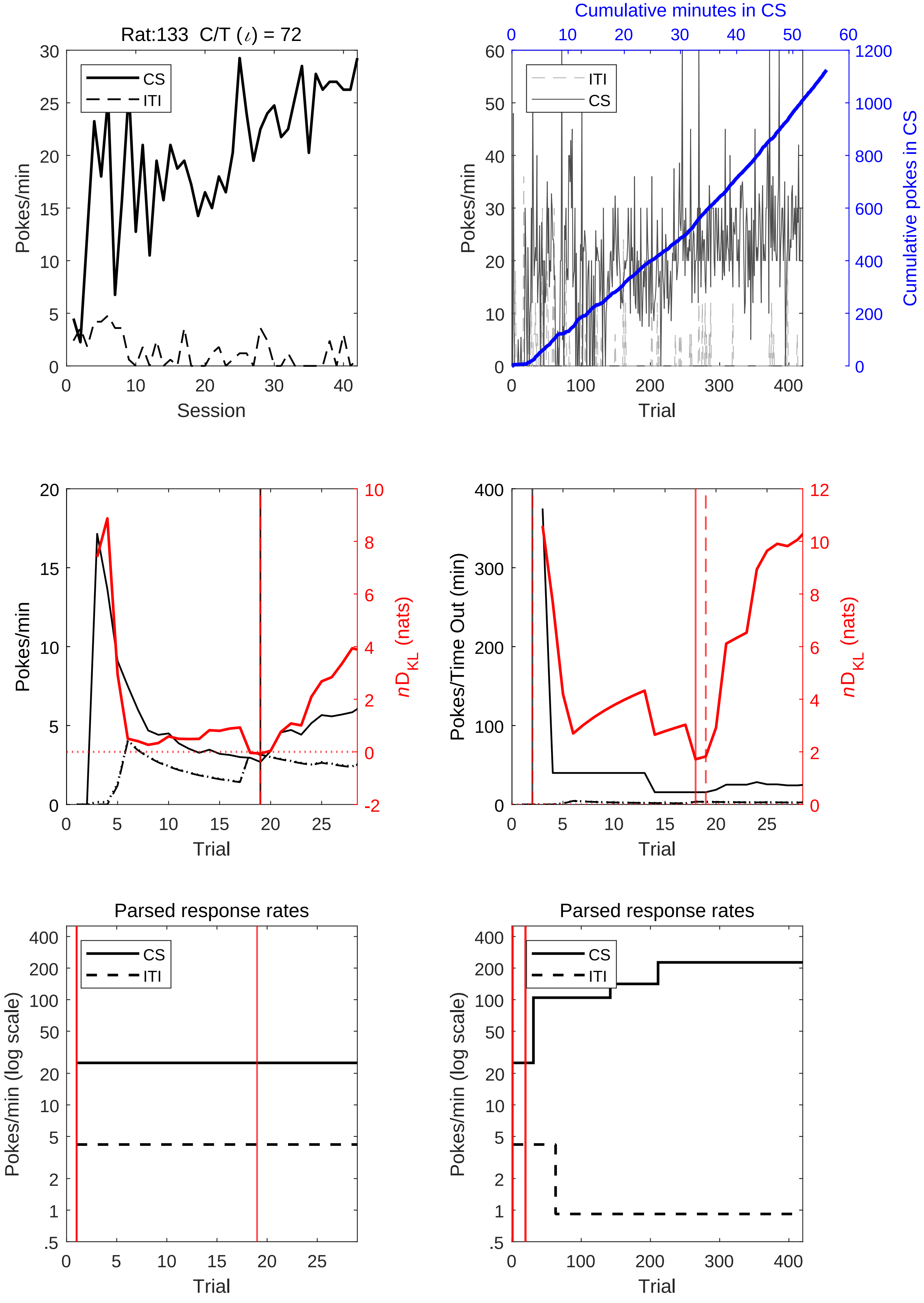
Rat:133 C/T (ι) = 72
CS
ITI
Pokes/min
Session
Cumulative minutes in CS
Cumulative pokes in CS
Trial
nD_KL (nats)
Pokes/Time Out (min)
Parsed response rates
Pokes/min (log scale)

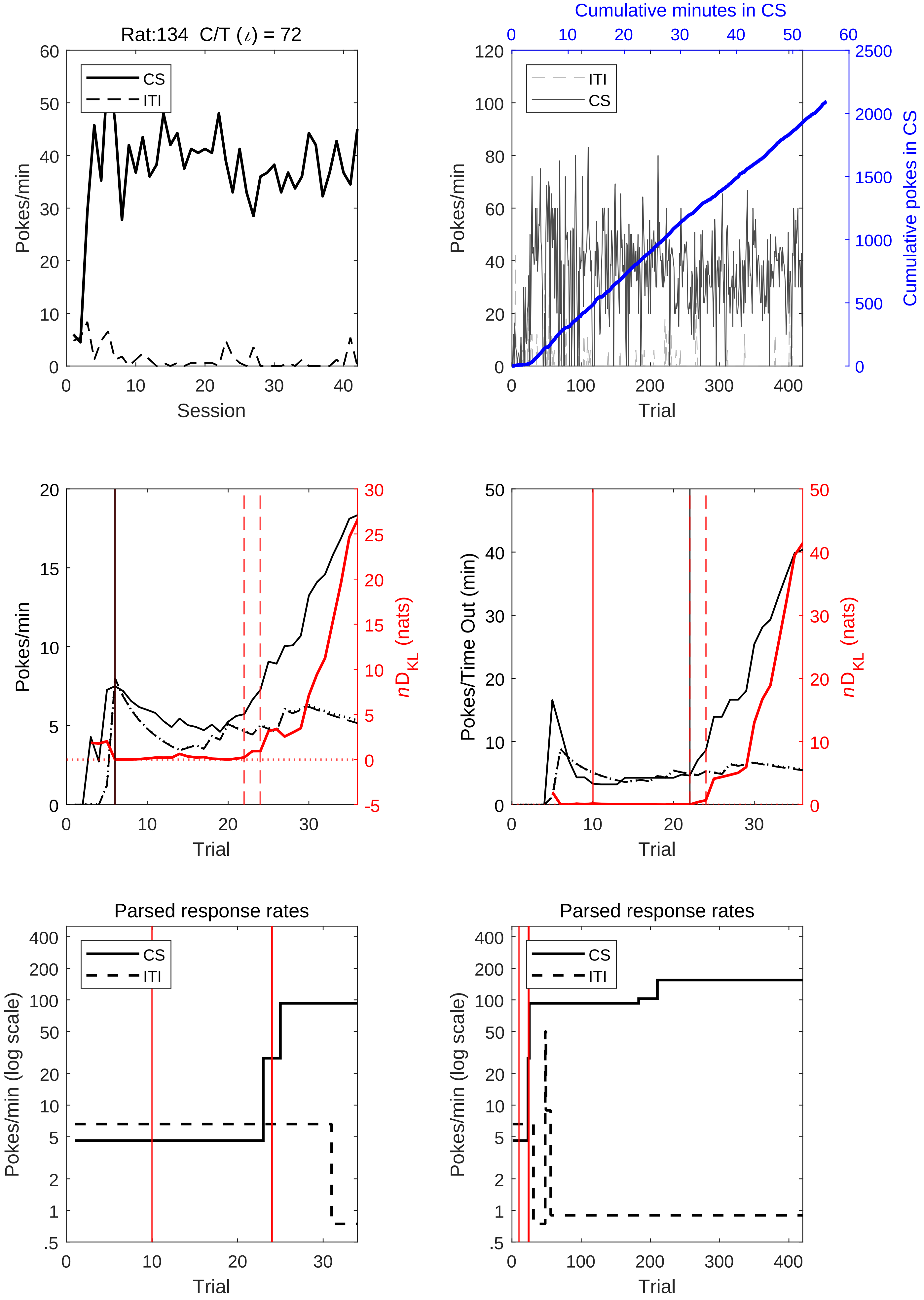

Rat:134 C/T (ι) = 72
CS
ITI
Pokes/min
Session
Cumulative minutes in CS
Cumulative pokes in CS
Trial
Pokes/Time Out (min)
nD_KL (nats)
Parsed response rates
Pokes/min (log scale)

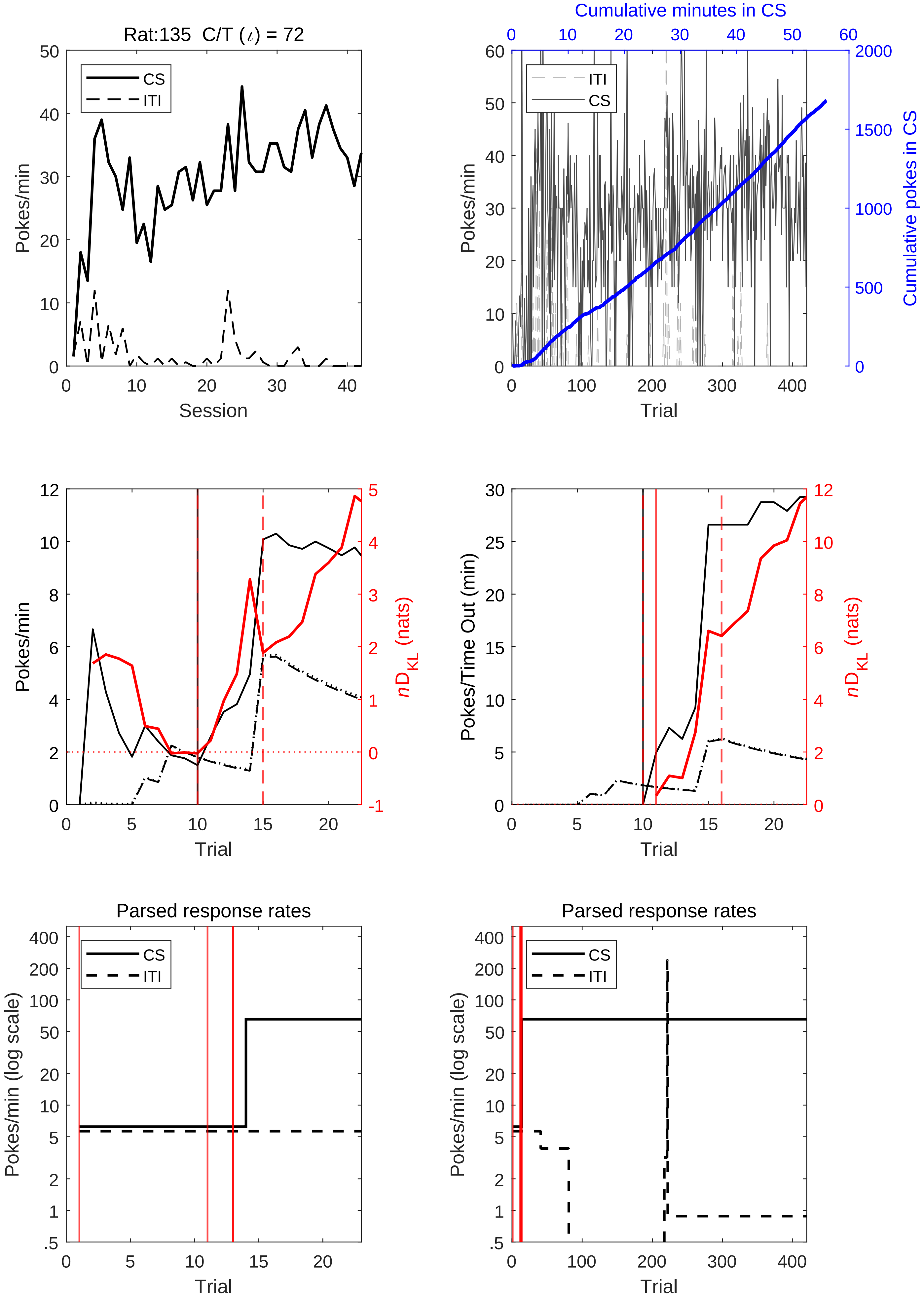
Rat:135 C/T (ι) = 72
CS
ITI
Pokes/min
Session
Cumulative minutes in CS
Cumulative pokes in CS
Trial
nD_KL (nats)
Pokes/Time Out (min)
Parsed response rates
Pokes/min (log scale)

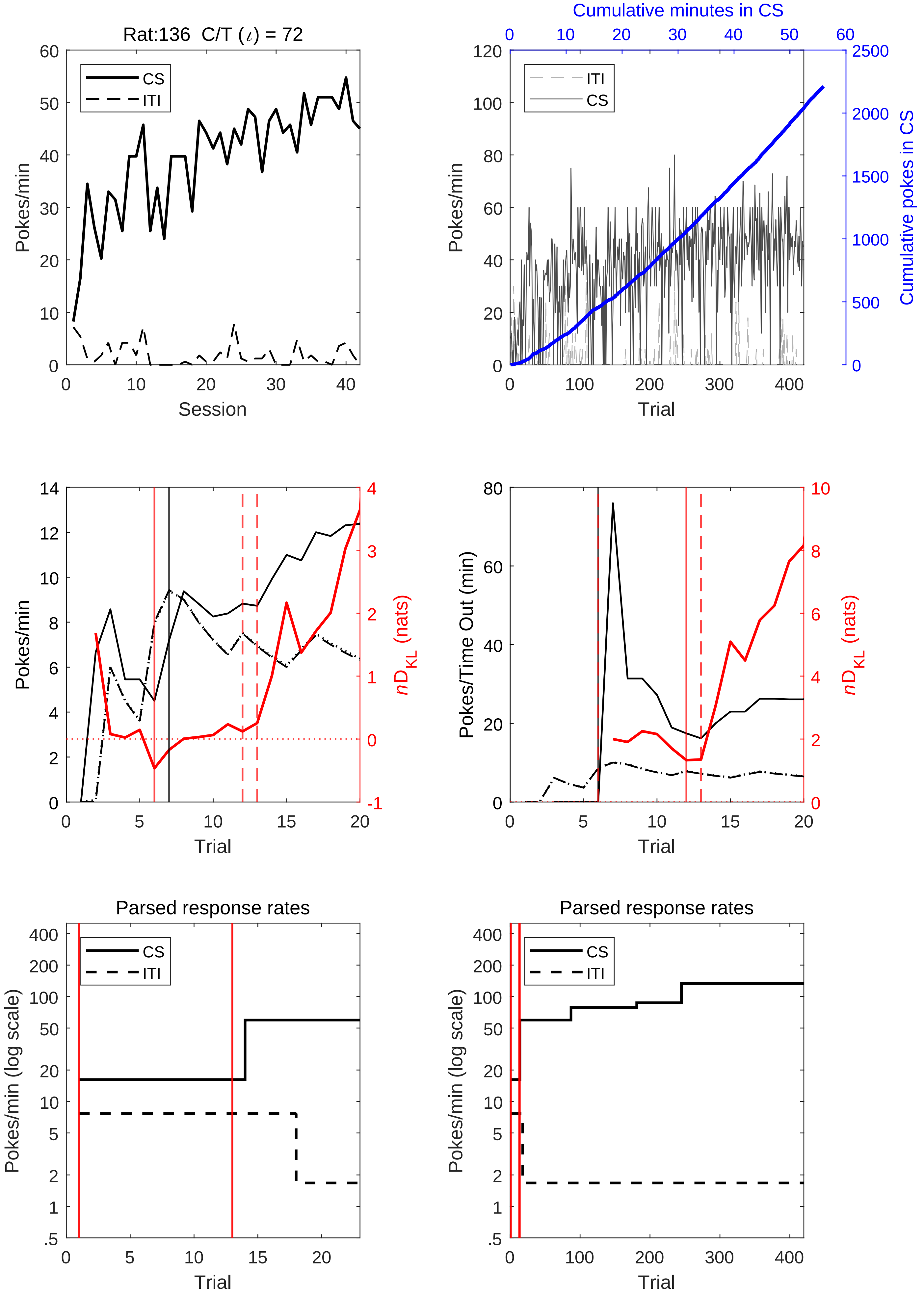

Rat:136 C/T (ι) = 72
CS
ITI
Pokes/min
Session
Cumulative minutes in CS
Cumulative pokes in CS
Trial
nD_KL (nats)
Pokes/Time Out (min)
Parsed response rates
Pokes/min (log scale)

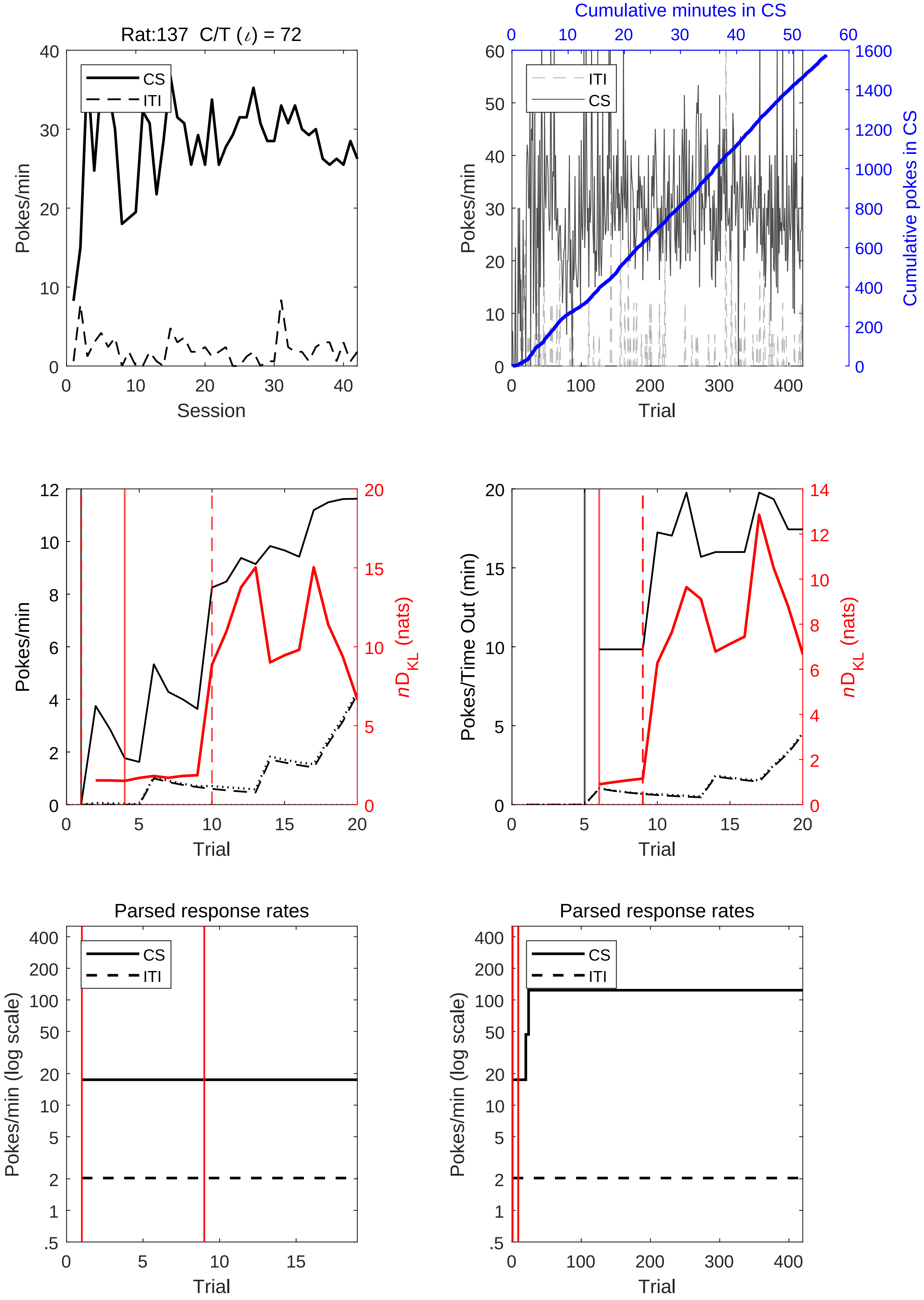
Rat:137 C/T (ι) = 72
CS
ITI
Pokes/min
Session
Cumulative minutes in CS
Cumulative pokes in CS
Trial
Pokes/Time Out (min)
nD_KL (nats)
Parsed response rates
Pokes/min (log scale)

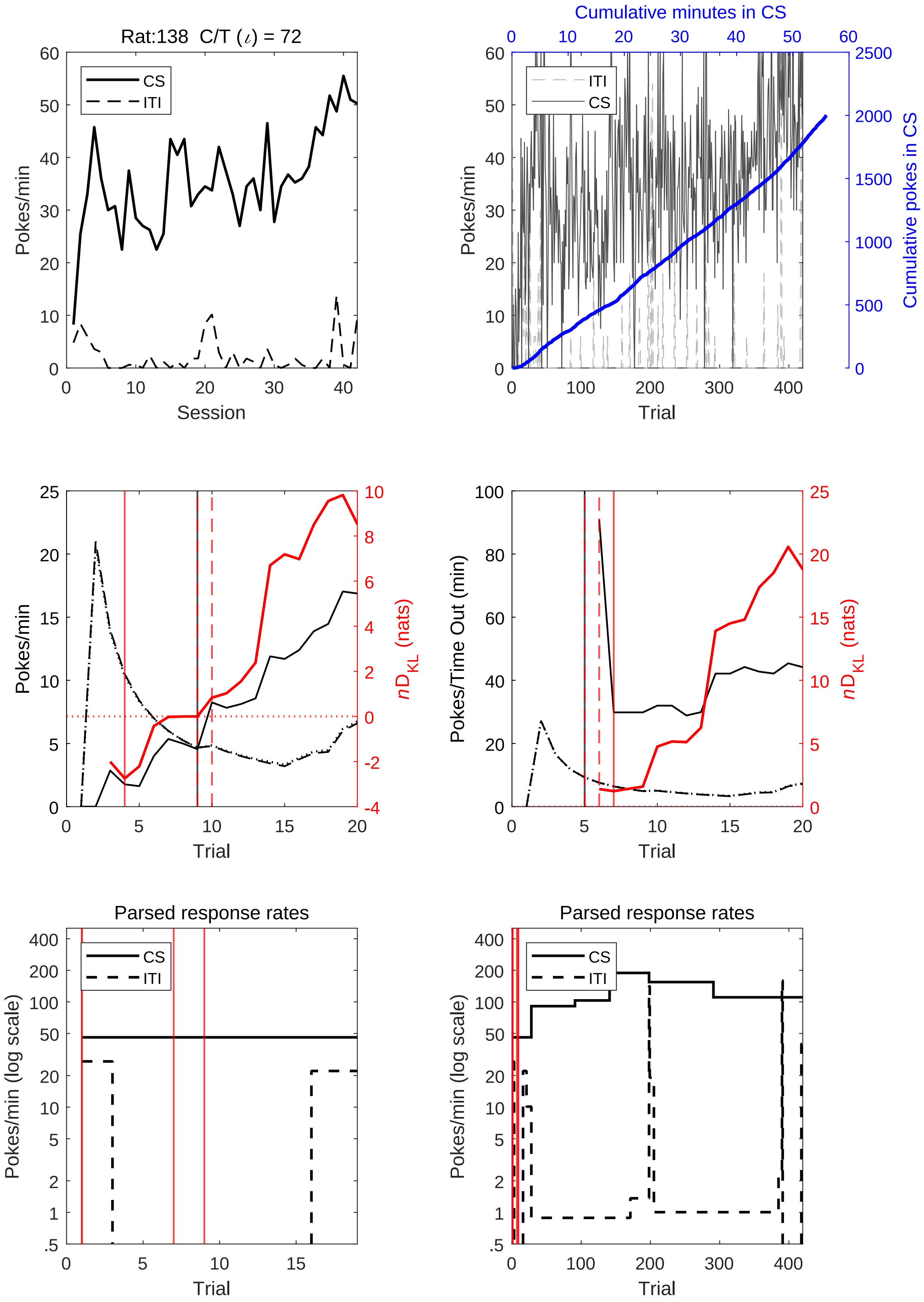

Rat:138 C/T (ι) = 72
CS
ITI
Pokes/min
Session
Cumulative minutes in CS
Cumulative pokes in CS
Trial
nD_KL (nats)
Pokes/Time Out (min)
Parsed response rates
Pokes/min (log scale)

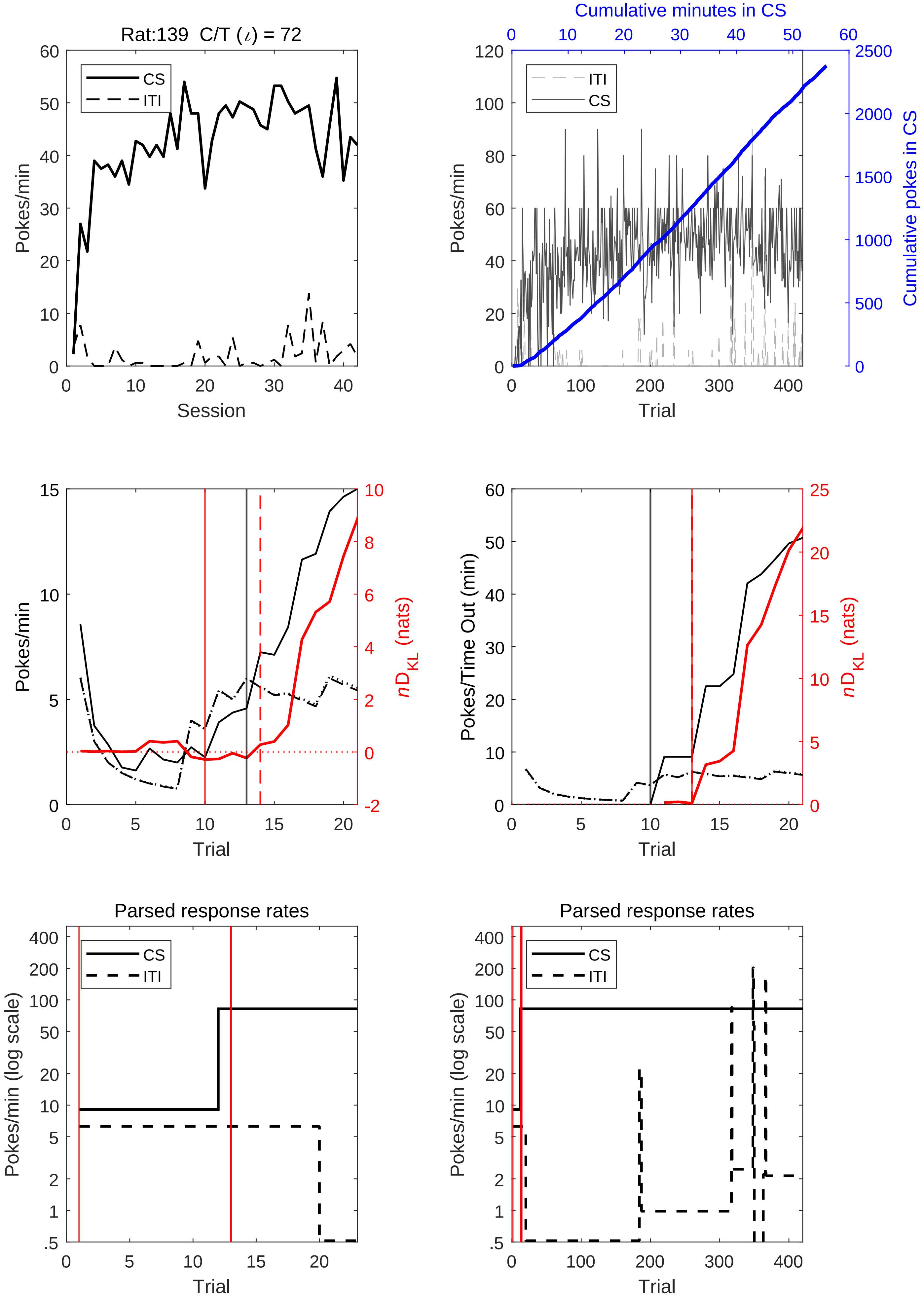

Rat:139 C/T (ι) = 72
CS
ITI
Pokes/min
Session
Cumulative minutes in CS
Cumulative pokes in CS
Trial
nD_KL (nats)
Pokes/Time Out (min)
Parsed response rates
Pokes/min (log scale)

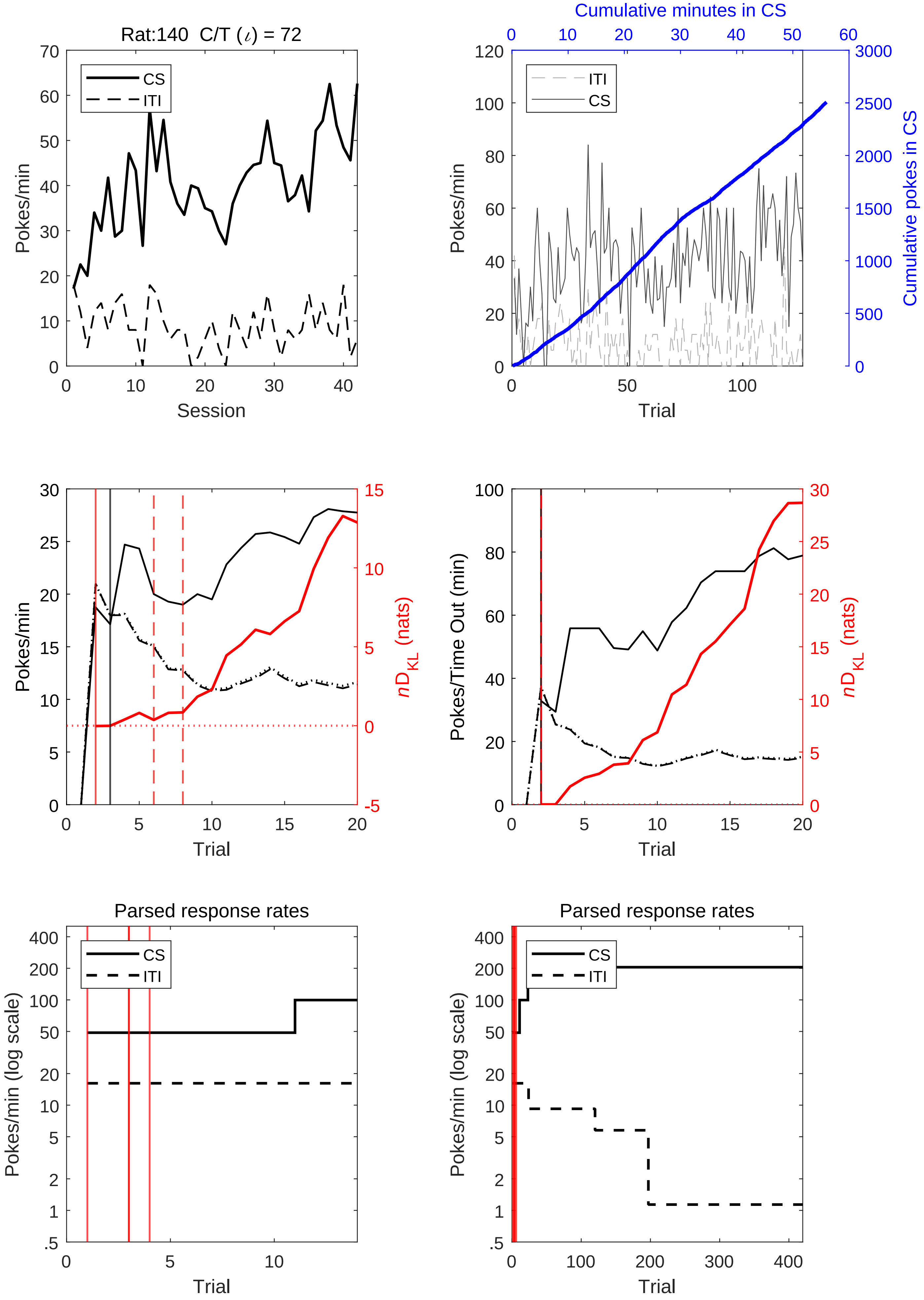

Rat:140 C/T ($\iota$) = 72
CS
ITI
Pokes/min
Session
Cumulative minutes in CS
Cumulative pokes in CS
Trial
Pokes/Time Out (min)
$nD_{KL}$ (nats)
Parsed response rates
Pokes/min (log scale)

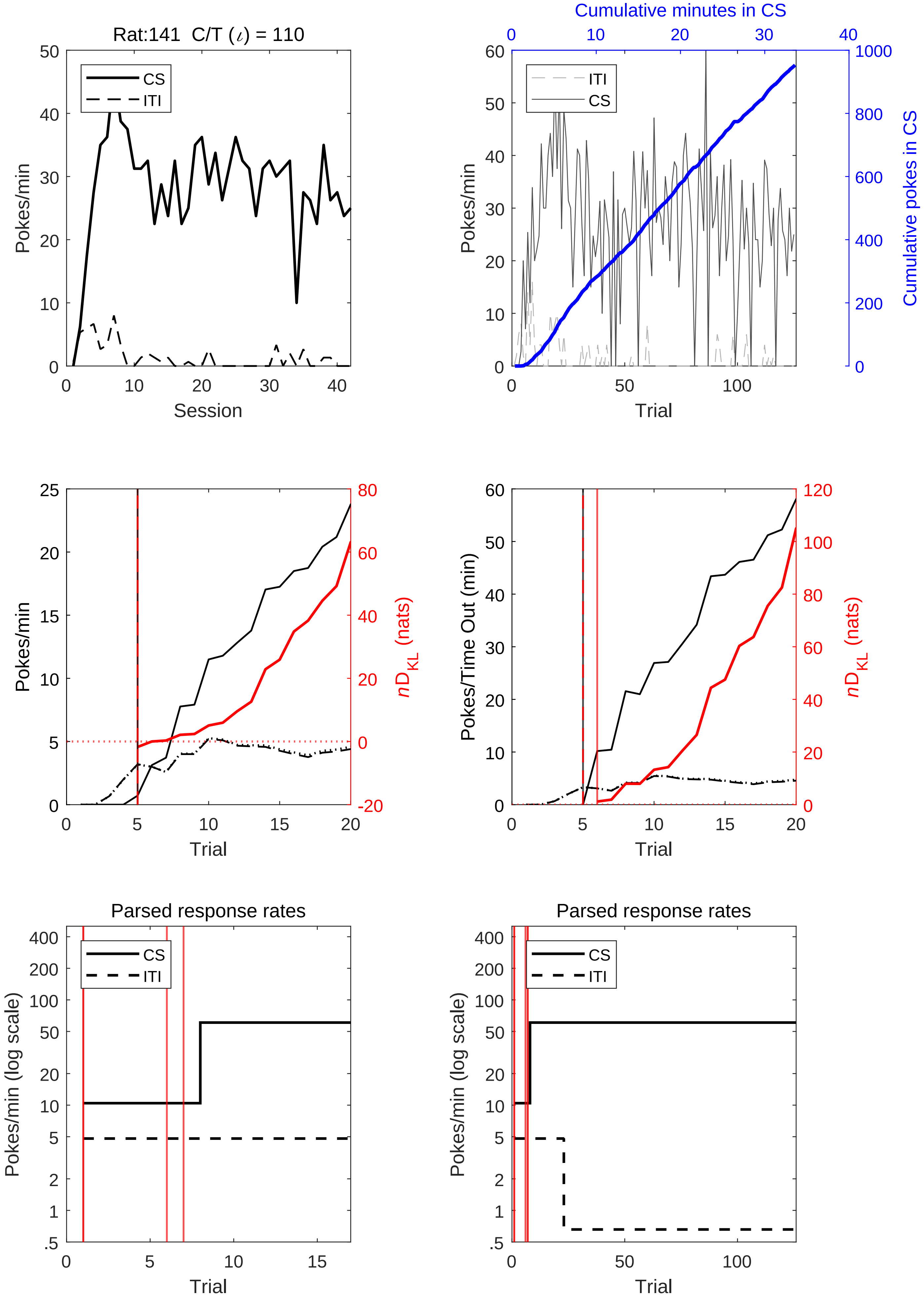

Rat:141 C/T (ι) = 110
CS
ITI
Pokes/min
Session
Cumulative minutes in CS
ITI
CS
Pokes/min
Cumulative pokes in CS
Trial
Pokes/min
nD_KL (nats)
Trial
Pokes/Time Out (min)
nD_KL (nats)
Trial
Parsed response rates
CS
ITI
Pokes/min (log scale)
Trial
Parsed response rates
CS
ITI
Pokes/min (log scale)
Trial

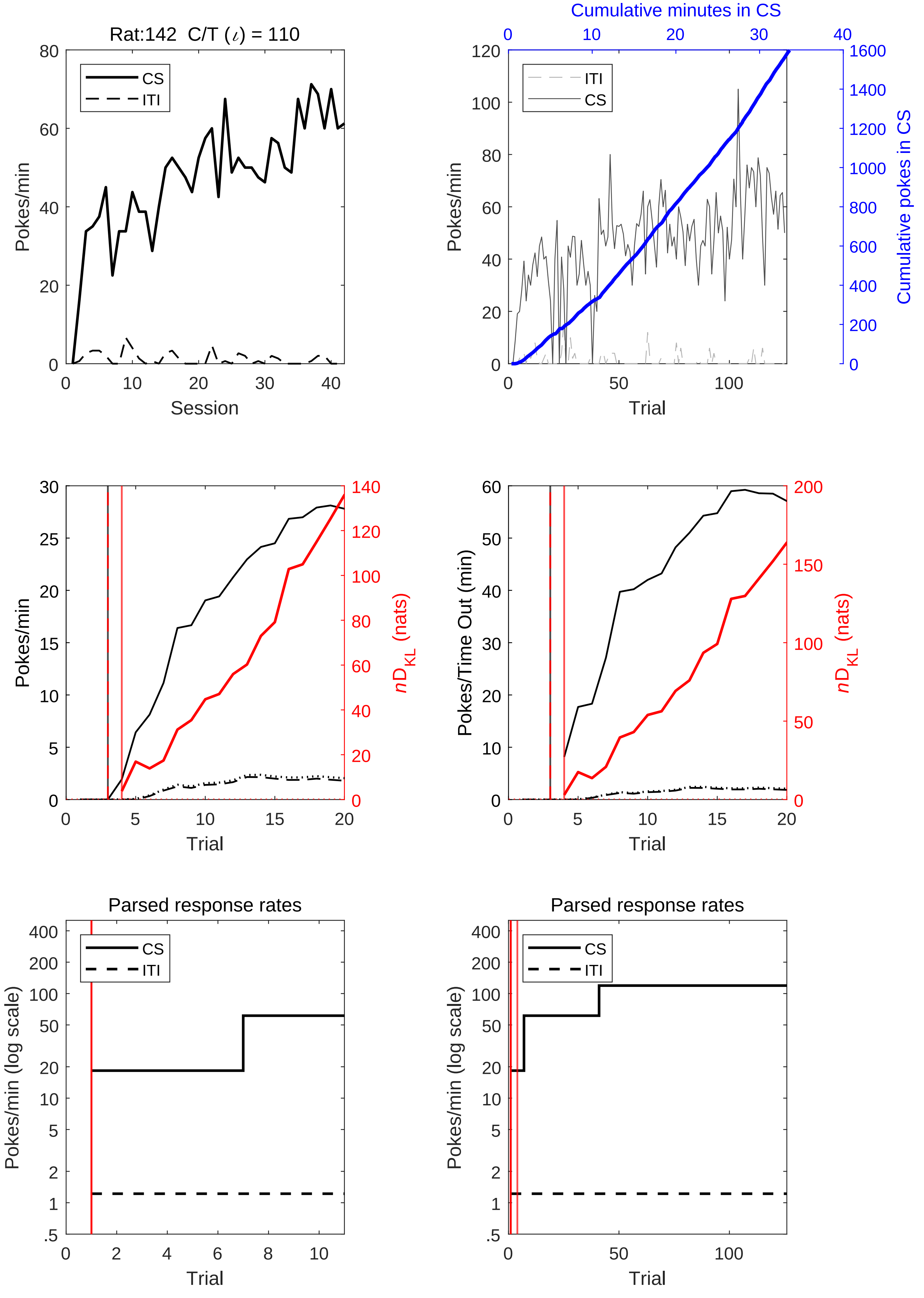

Rat:142 C/T ($\iota$) = 110
CS
ITI
Pokes/min
Session
Cumulative minutes in CS
Cumulative pokes in CS
Trial
$nD_{KL}$ (nats)
Pokes/Time Out (min)
Parsed response rates
Pokes/min (log scale)

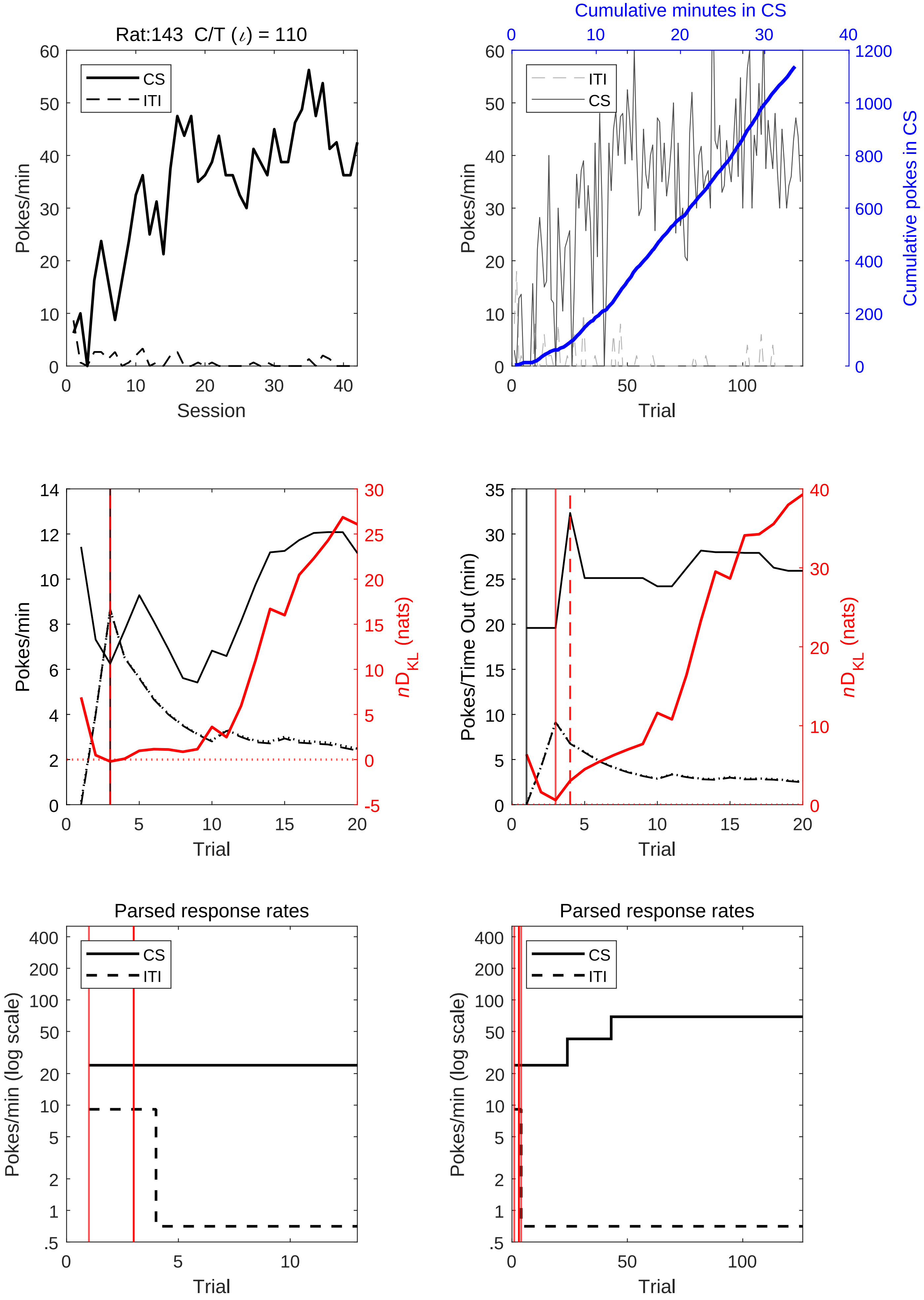
Rat:143 C/T (ι) = 110
CS
ITI
Pokes/min
Session
Cumulative minutes in CS
Cumulative pokes in CS
Trial
Pokes/Time Out (min)
nD_KL (nats)
Parsed response rates
Pokes/min (log scale)

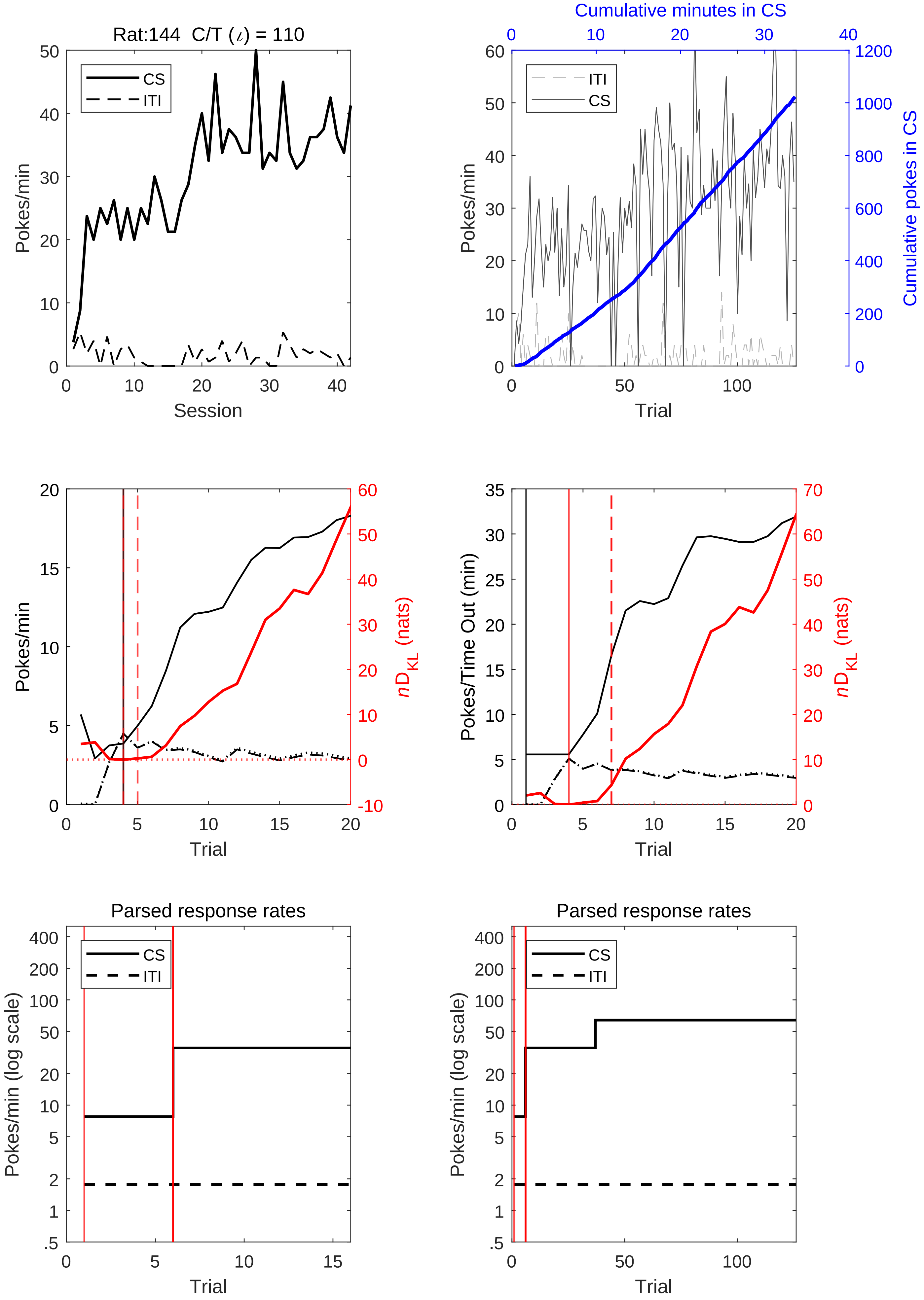
Rat:144 C/T (ι) = 110
CS
ITI
Pokes/min
Session
Cumulative minutes in CS
Cumulative pokes in CS
Trial
nD_KL (nats)
Pokes/Time Out (min)
Parsed response rates
Parsed response rates
Pokes/min (log scale)
Pokes/min (log scale)

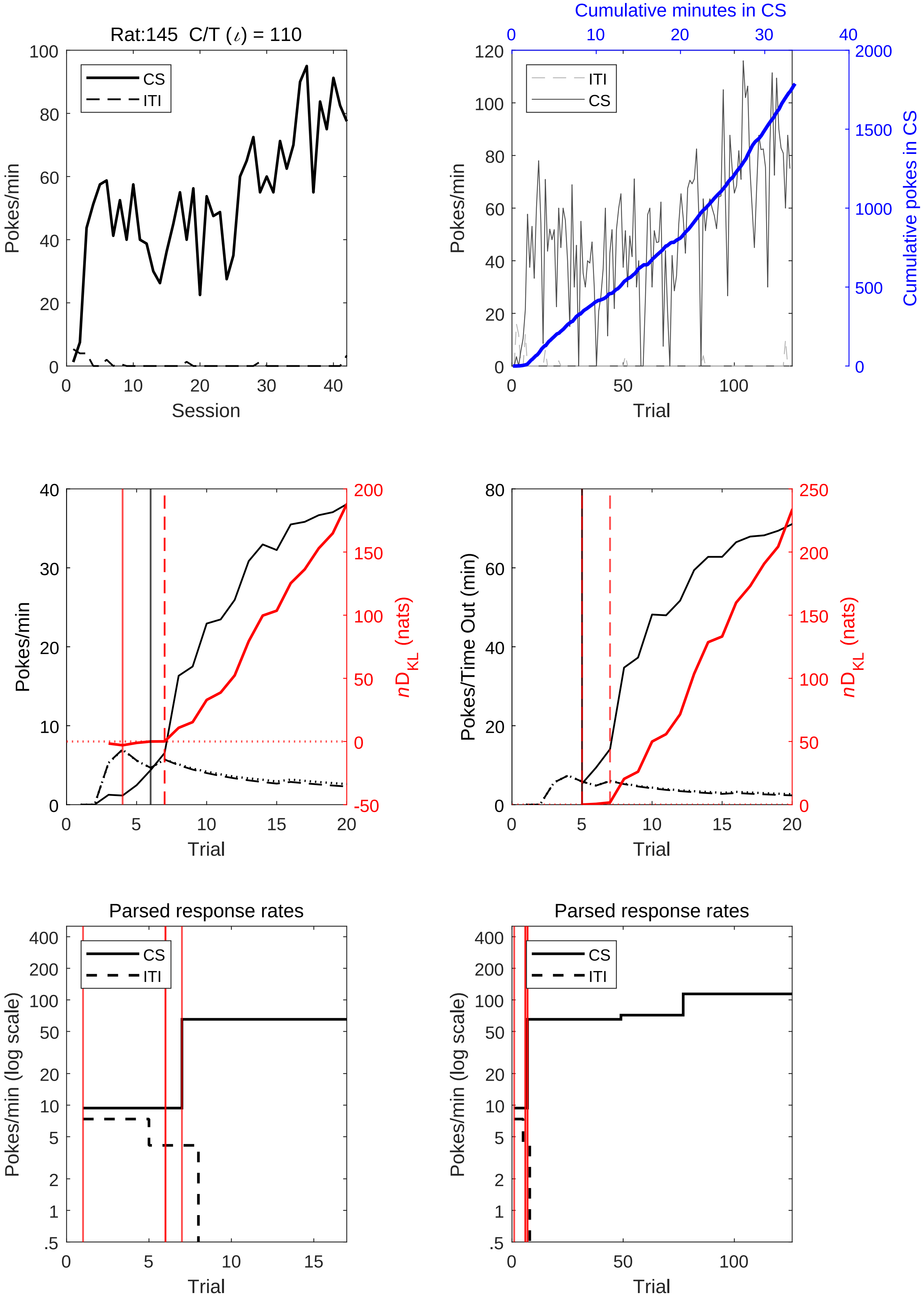
Rat:145 C/T (ι) = 110
CS
ITI
Pokes/min
Session
Cumulative minutes in CS
Cumulative pokes in CS
Trial
nD_KL (nats)
Pokes/Time Out (min)
Parsed response rates
Pokes/min (log scale)

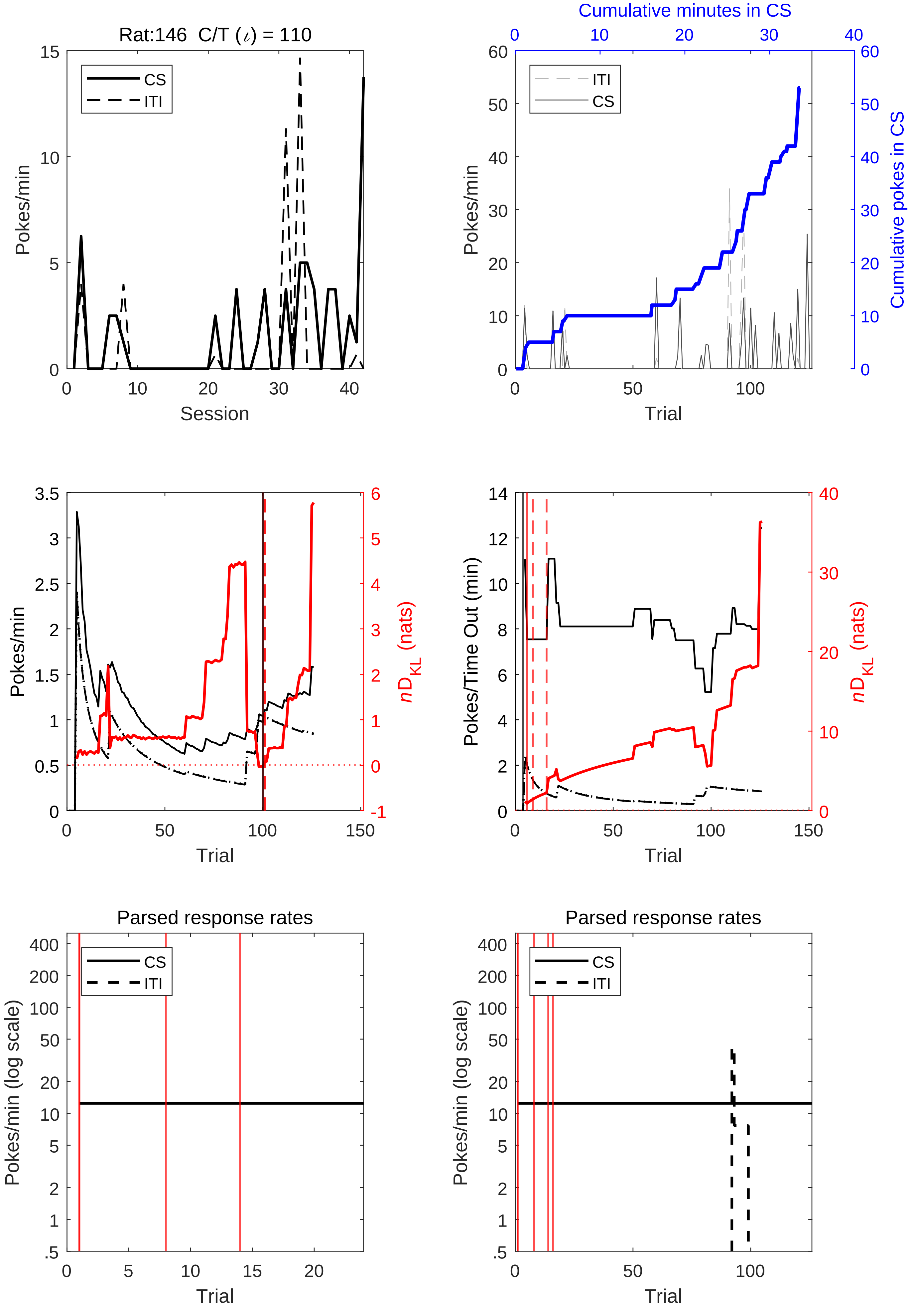
Rat:146 C/T (ι) = 110
CS
ITI
Pokes/min
Session
Cumulative minutes in CS
Pokes/min
Cumulative pokes in CS
Trial
Pokes/min
nD_KL (nats)
Trial
Pokes/Time Out (min)
nD_KL (nats)
Trial
Parsed response rates
Pokes/min (log scale)
Trial
Parsed response rates
Pokes/min (log scale)
Trial

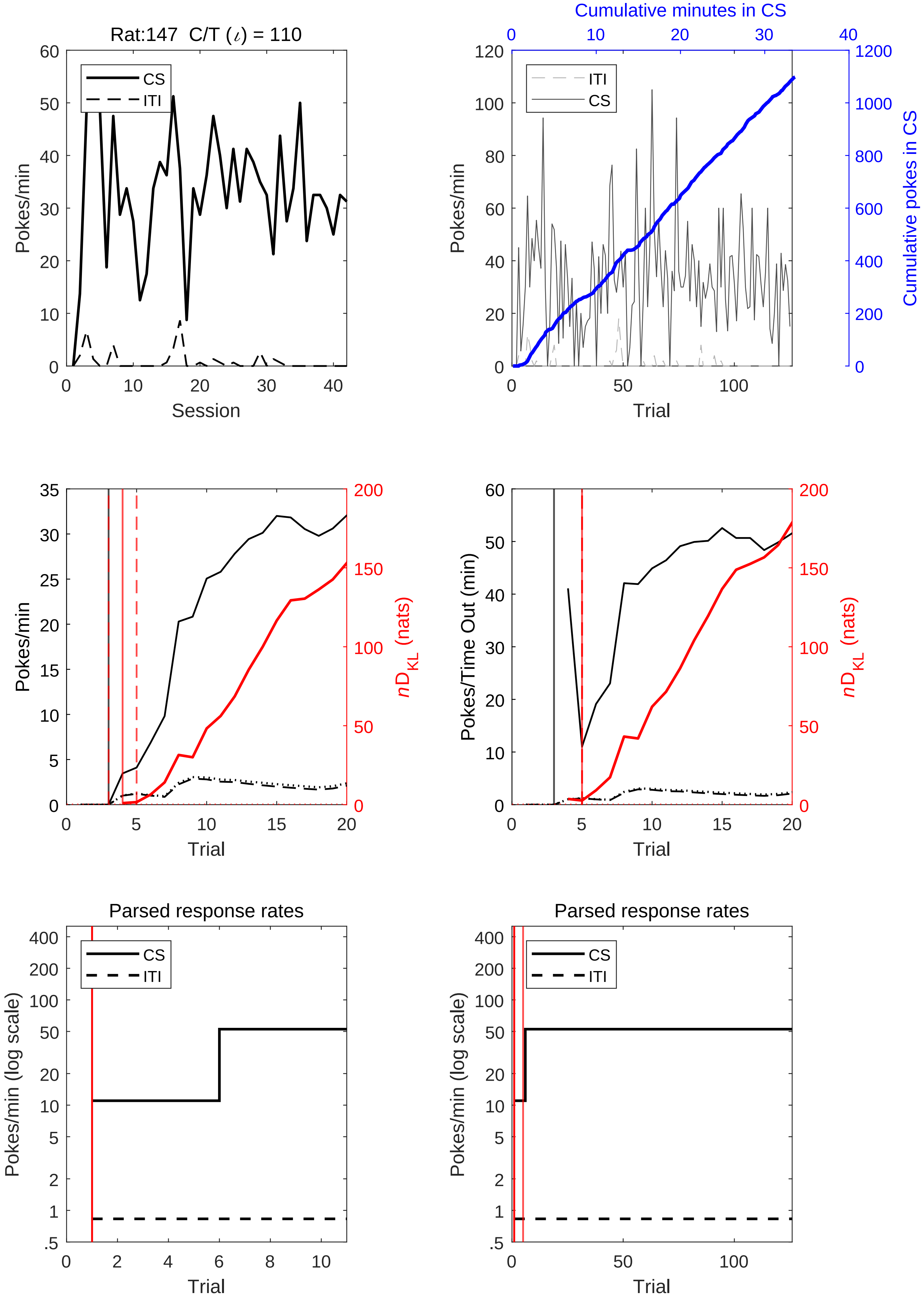

Rat:147 C/T (ι) = 110
CS
ITI
Pokes/min
Session
Cumulative minutes in CS
Cumulative pokes in CS
Trial
Pokes/Time Out (min)
nD_KL (nats)
Parsed response rates
Pokes/min (log scale)

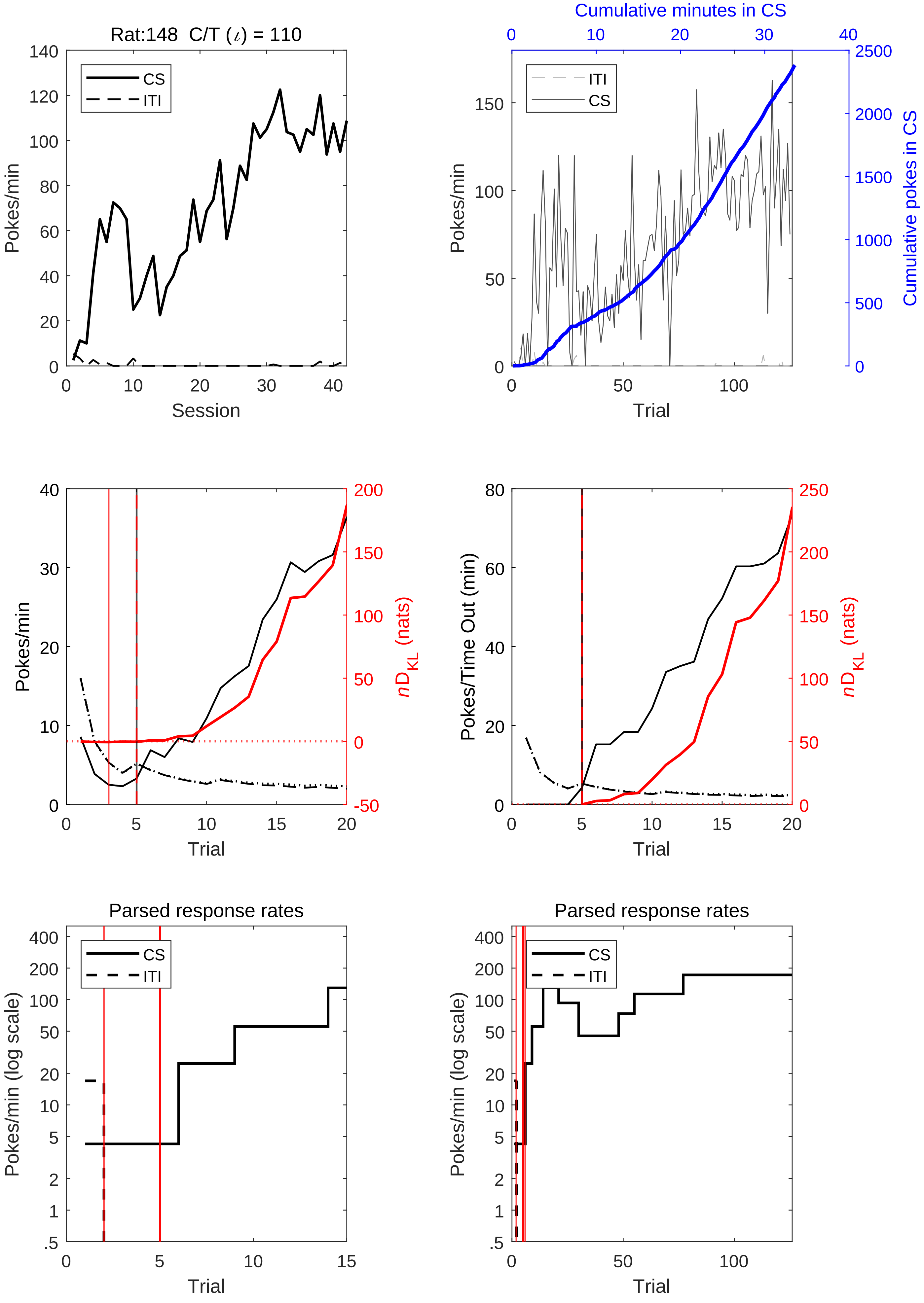

Rat:148 C/T (ι) = 110
CS
ITI
Pokes/min
Session
Cumulative minutes in CS
Cumulative pokes in CS
Trial
nD_KL (nats)
Pokes/Time Out (min)
Parsed response rates
Pokes/min (log scale)

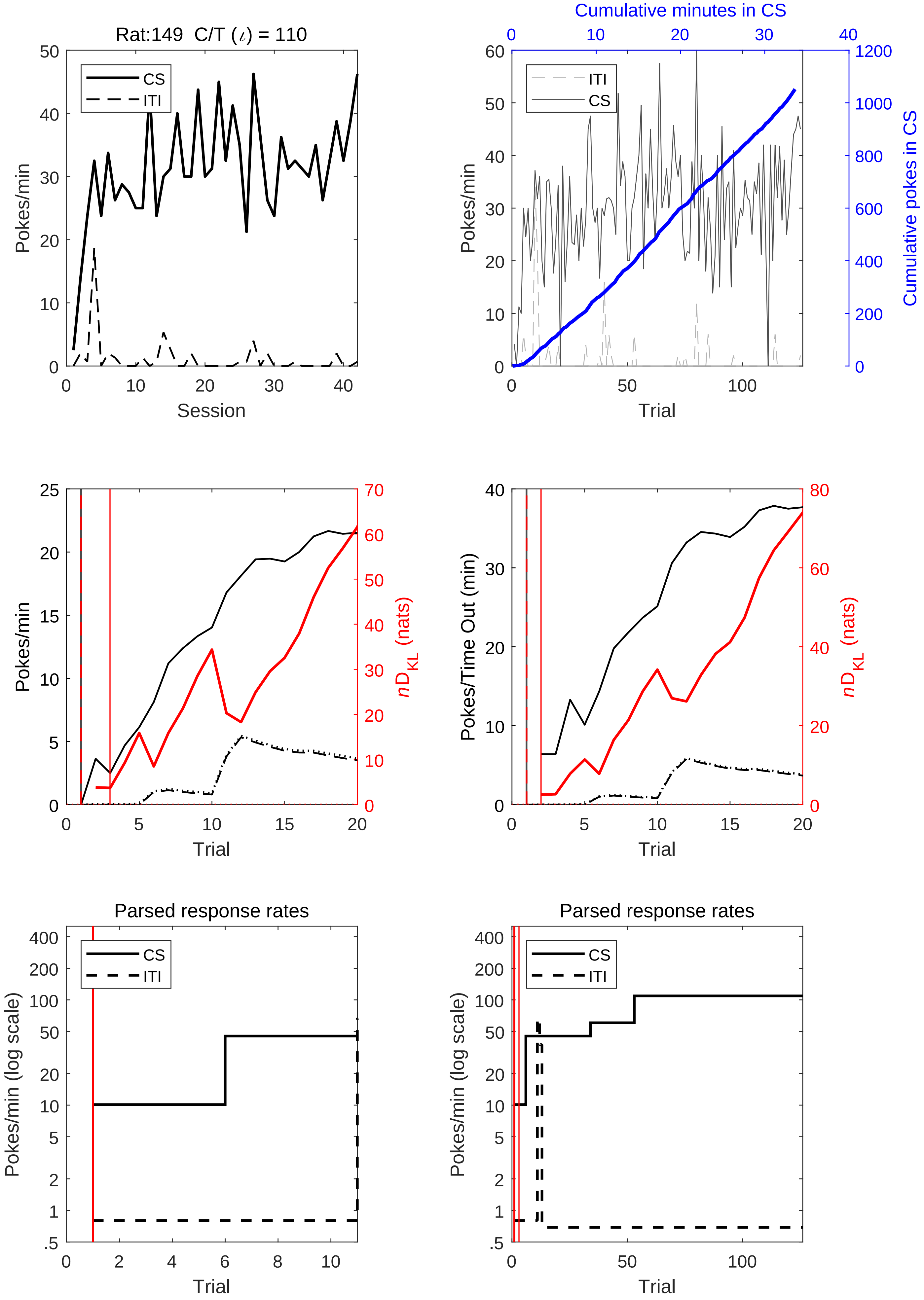
Rat:149 C/T (ι) = 110
CS
ITI
Pokes/min
Session
Cumulative minutes in CS
ITI
CS
Pokes/min
Cumulative pokes in CS
Trial
Pokes/min
$nD_{KL}$ (nats)
Trial
Pokes/Time Out (min)
$nD_{KL}$ (nats)
Trial
Parsed response rates
CS
ITI
Pokes/min (log scale)
Trial
Parsed response rates
CS
ITI
Pokes/min (log scale)
Trial

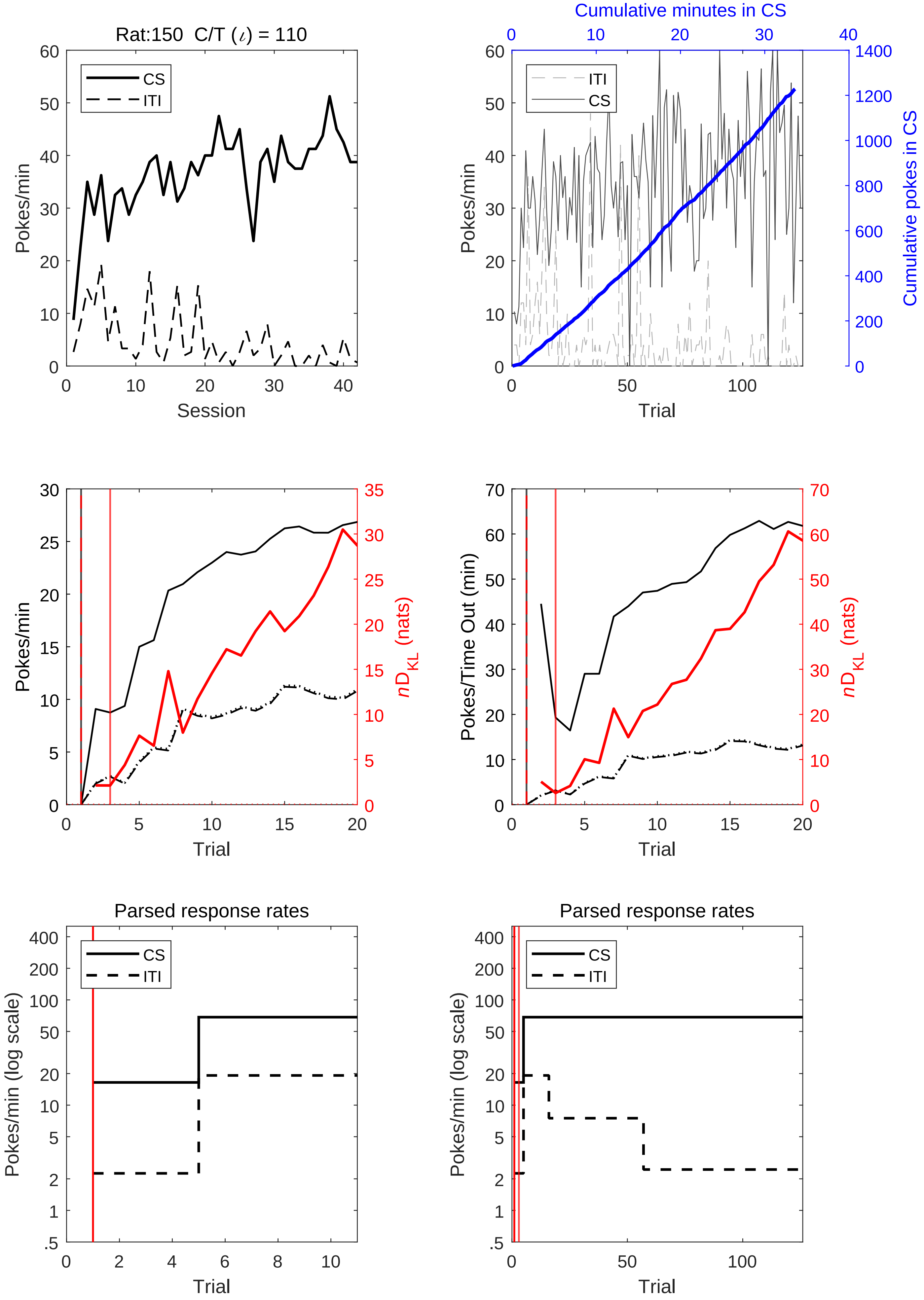

Rat:150 C/T (ι) = 110
CS
ITI
Pokes/min
Session
Cumulative minutes in CS
Cumulative pokes in CS
Trial
nD_KL (nats)
Pokes/Time Out (min)
Parsed response rates
Pokes/min (log scale)

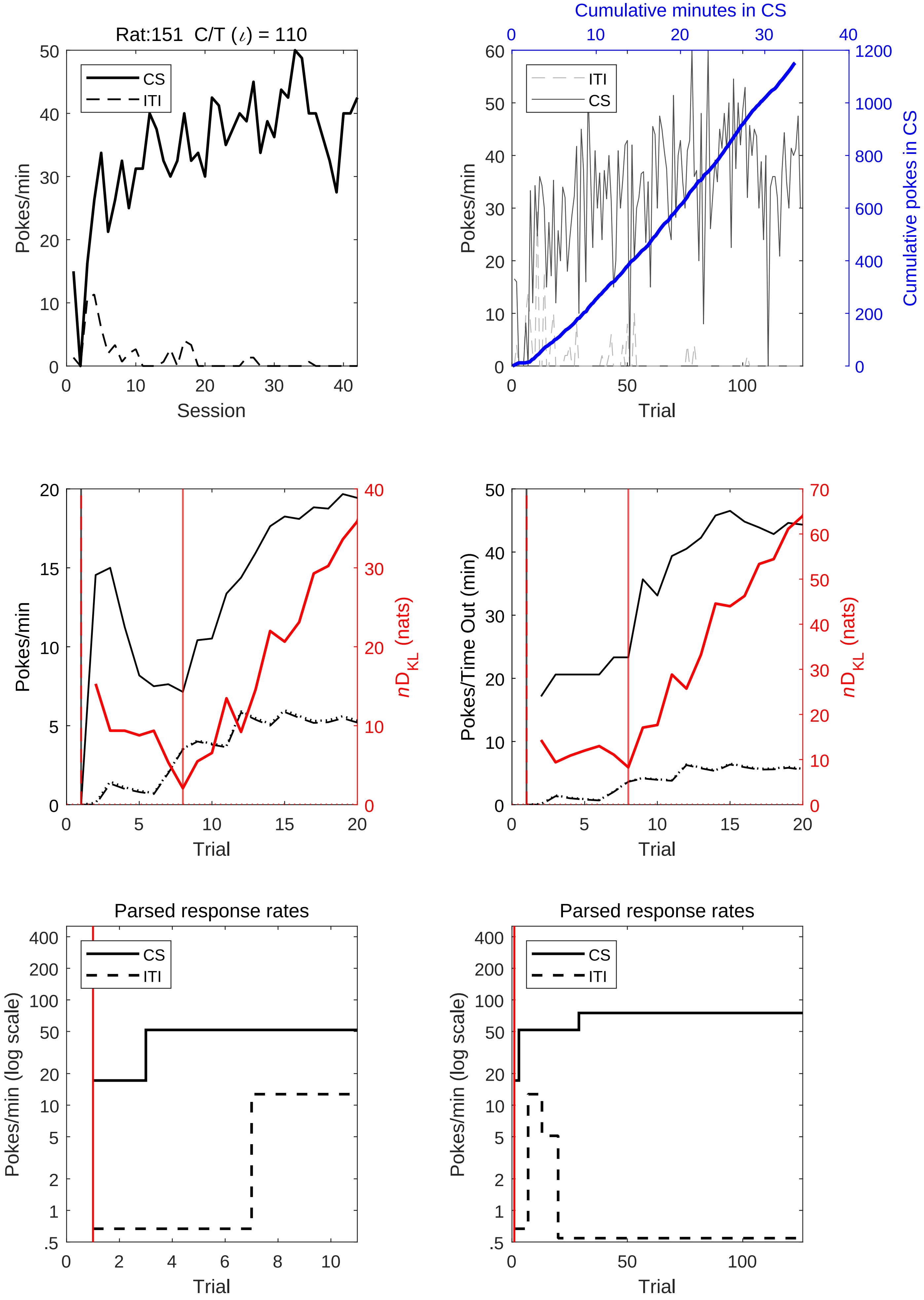
Rat:151 C/T (ι) = 110
CS
ITI
Pokes/min
Session
Cumulative minutes in CS
Cumulative pokes in CS
Trial
nD_KL (nats)
Pokes/Time Out (min)
Parsed response rates
Pokes/min (log scale)

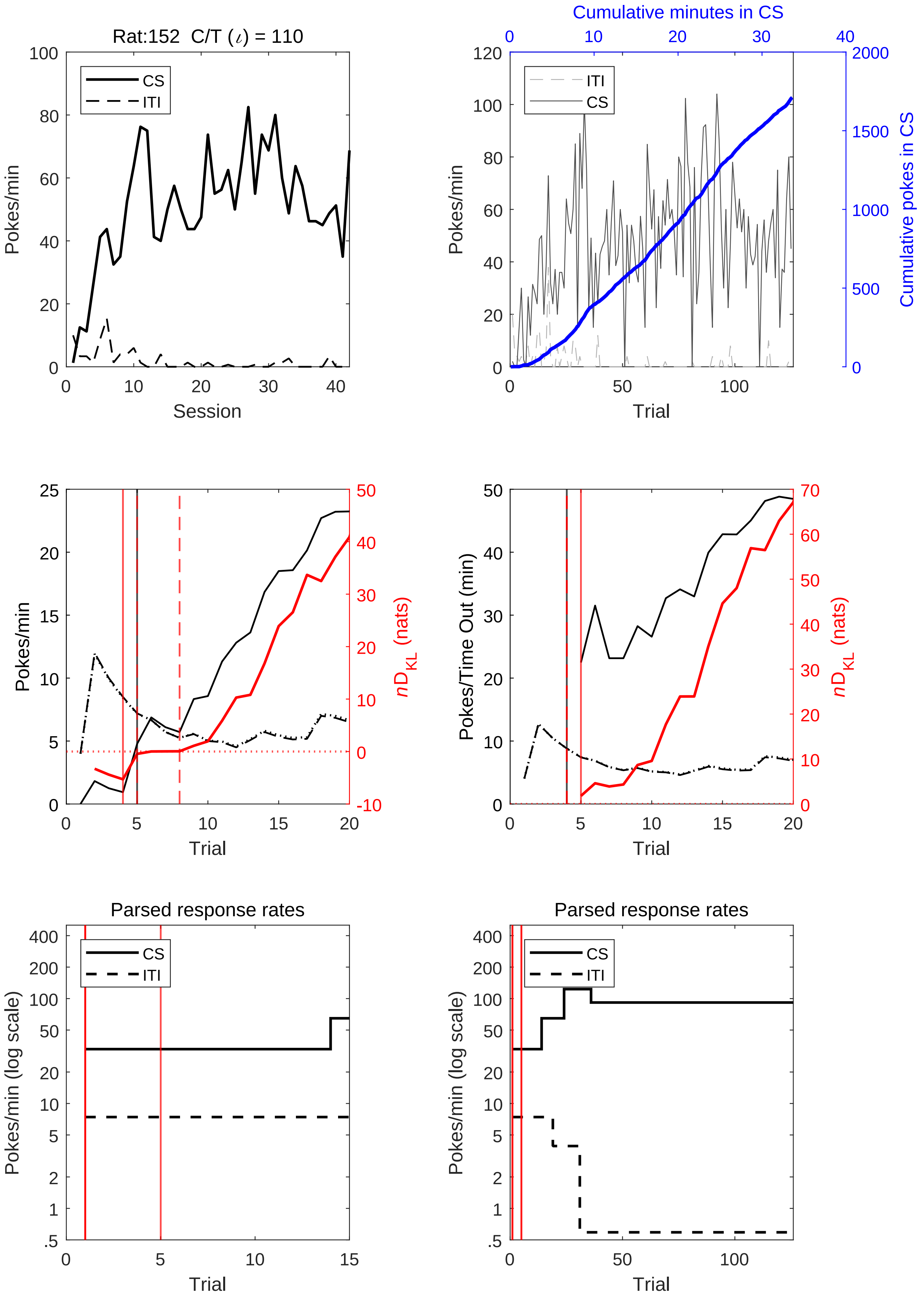

Rat:152 C/T (ι) = 110
Pokes/min
Session
CS
ITI
Cumulative minutes in CS
Cumulative pokes in CS
Pokes/min
Trial
ITI
CS
Pokes/min
Trial
nD_KL (nats)
Pokes/Time Out (min)
Trial
nD_KL (nats)
Parsed response rates
Pokes/min (log scale)
Trial
CS
ITI
Parsed response rates
Pokes/min (log scale)
Trial
CS
ITI

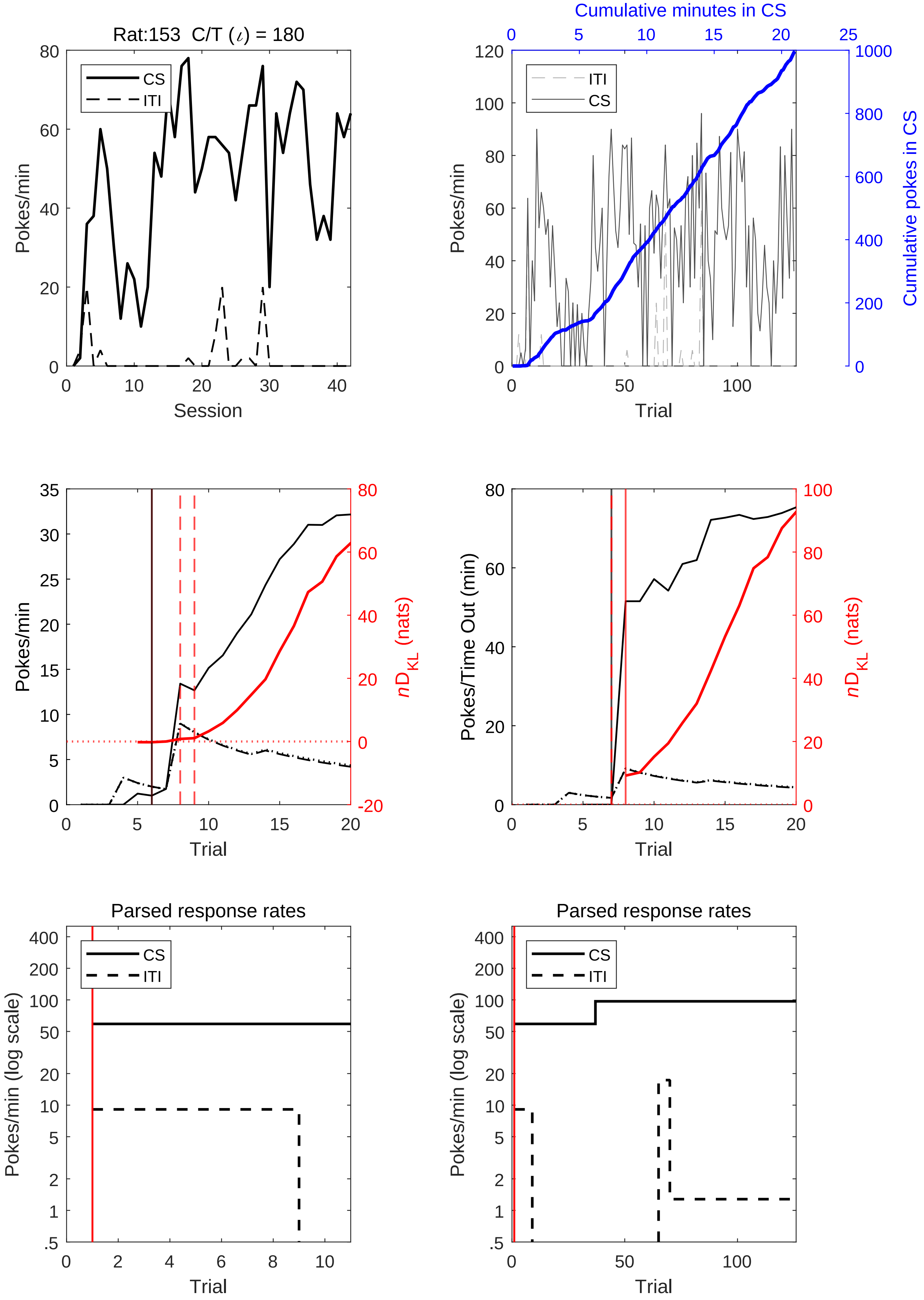
Rat:153 C/T (ι) = 180
CS
ITI
Pokes/min
Session
Cumulative minutes in CS
Cumulative pokes in CS
Trial
nD_KL (nats)
Pokes/Time Out (min)
Parsed response rates
Pokes/min (log scale)

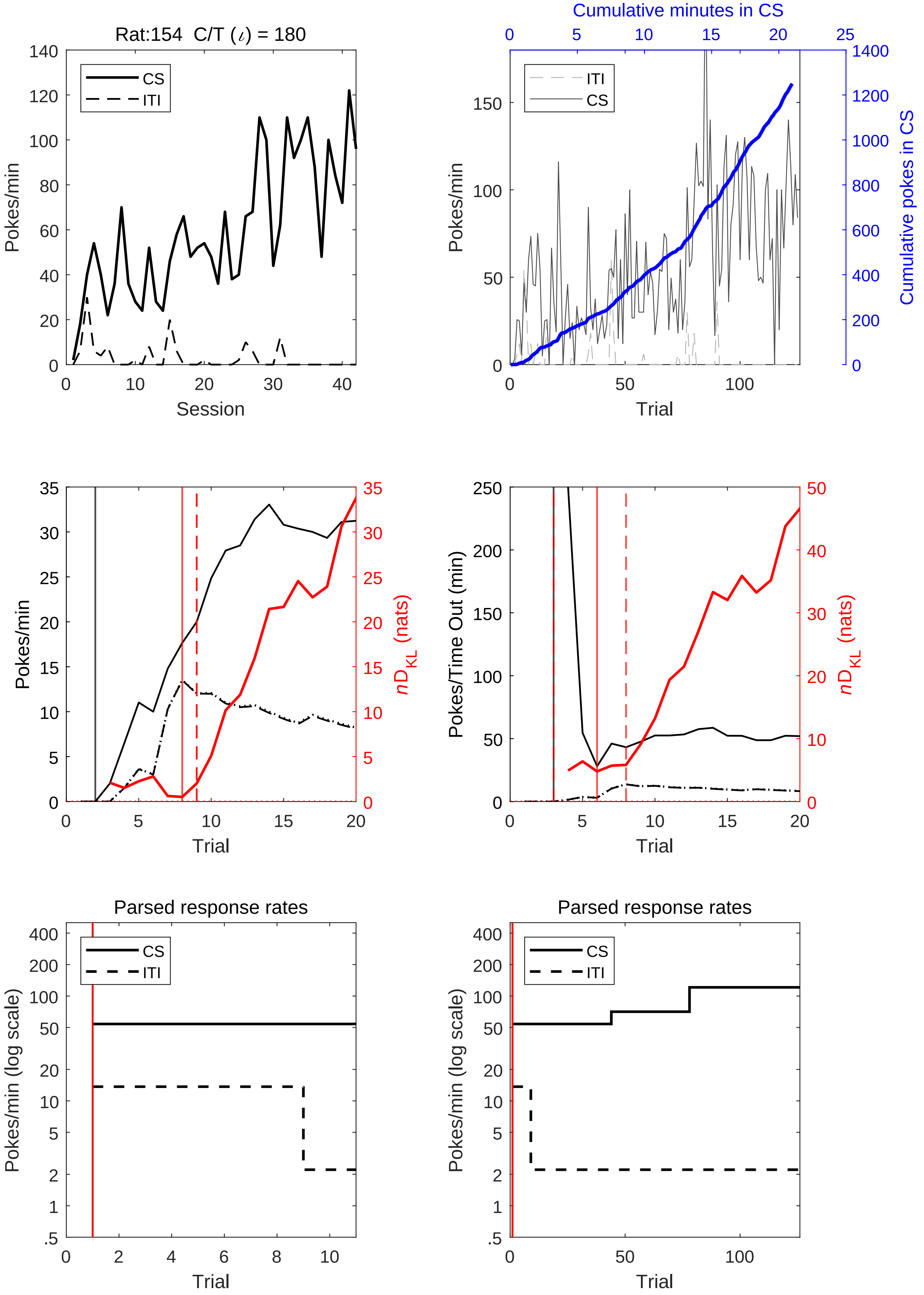
Rat:154 C/T (ι) = 180
CS
ITI
Pokes/min
Session
Cumulative minutes in CS
ITI
CS
Pokes/min
Cumulative pokes in CS
Trial
Pokes/min
nD_KL (nats)
Trial
Pokes/Time Out (min)
nD_KL (nats)
Trial
Parsed response rates
CS
ITI
Pokes/min (log scale)
Trial
Parsed response rates
CS
ITI
Pokes/min (log scale)
Trial

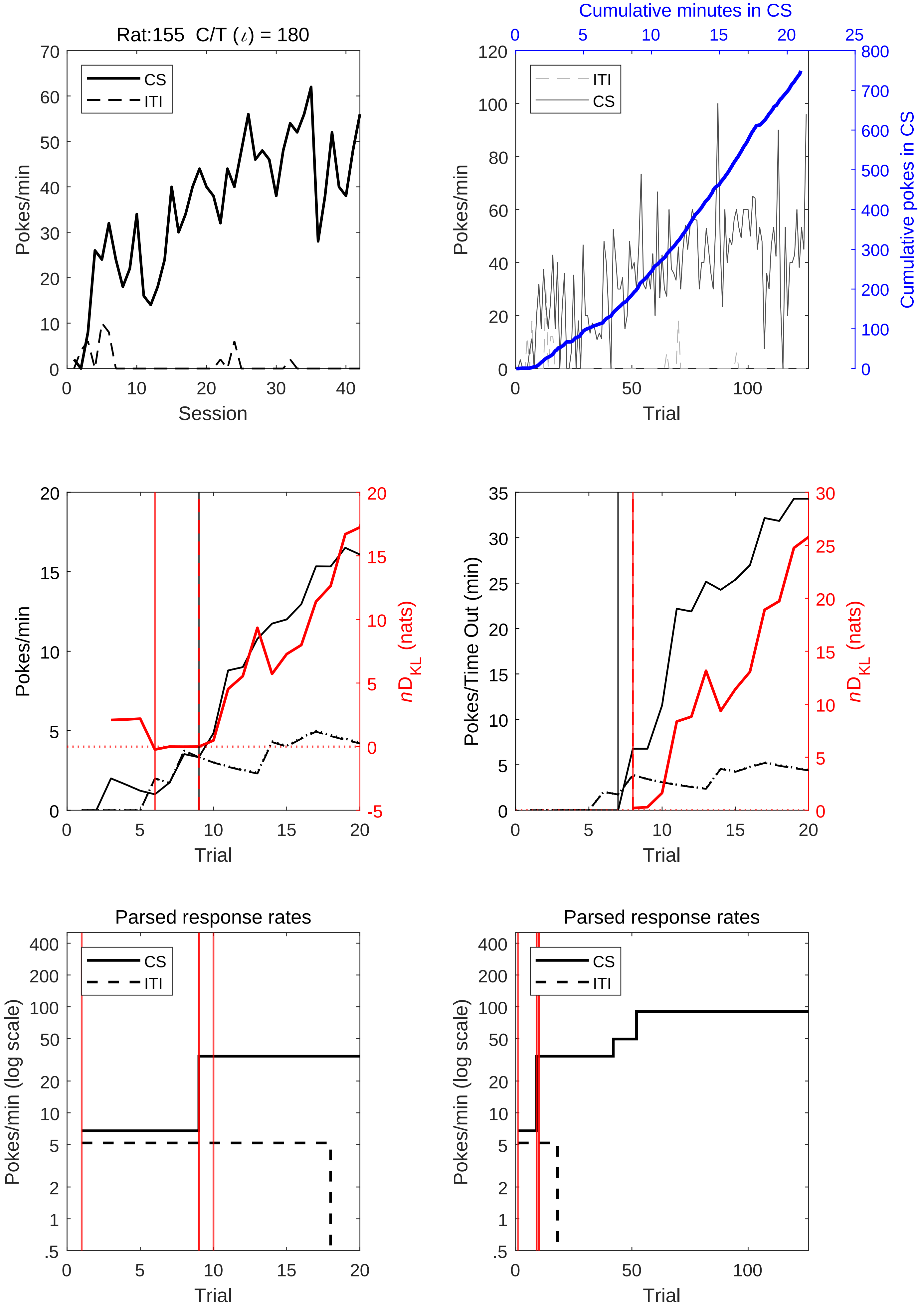
Rat:155 C/T (ι) = 180
CS
ITI
Pokes/min
Session
Cumulative minutes in CS
Cumulative pokes in CS
Trial
nD_KL (nats)
Pokes/Time Out (min)
Parsed response rates
Pokes/min (log scale)
Parsed response rates
Pokes/min (log scale)

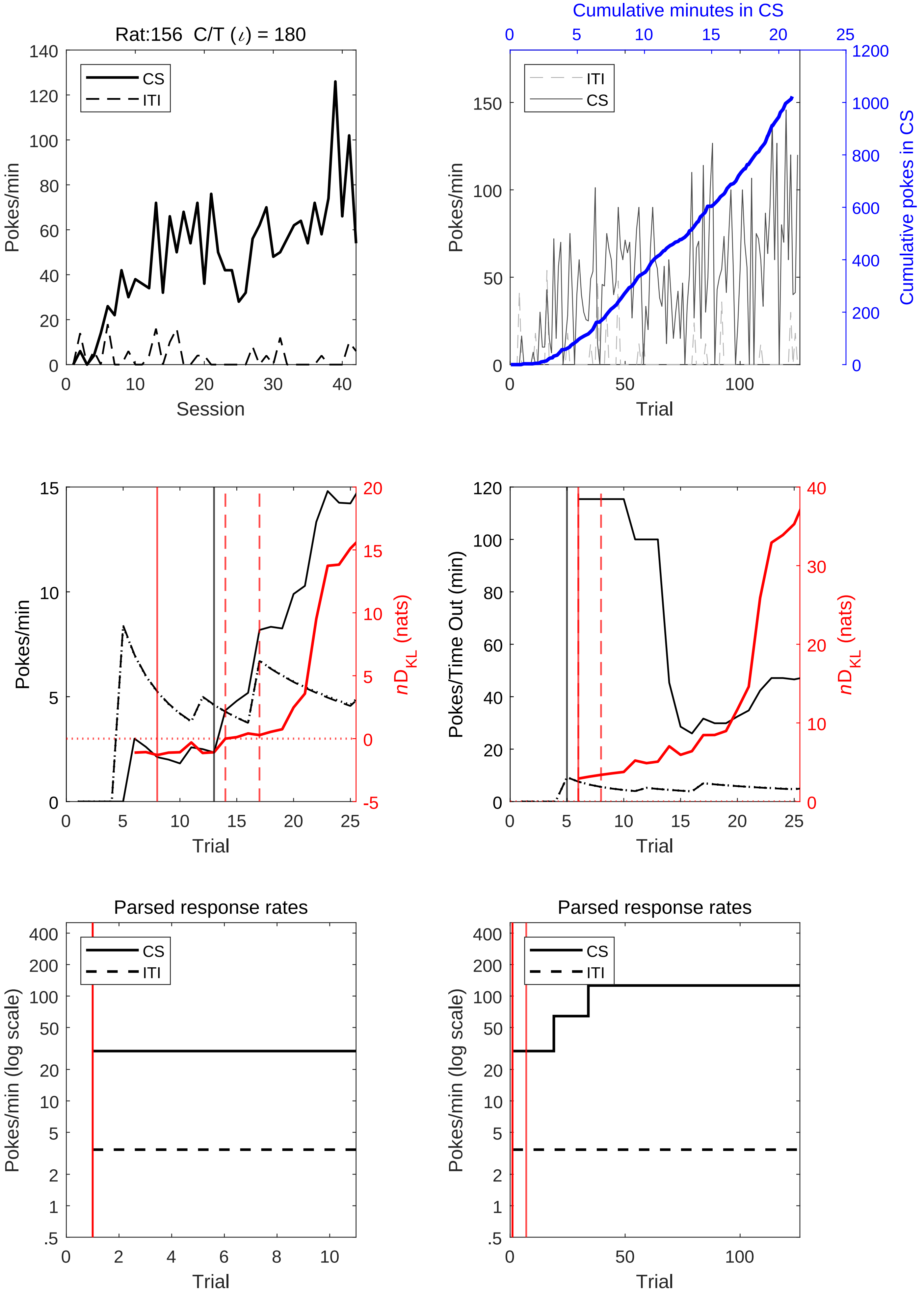

Rat:156  C/T ($\iota$) = 180
CS
ITI
Pokes/min
Session
Cumulative minutes in CS
Cumulative pokes in CS
Trial
nD$_{KL}$ (nats)
Pokes/Time Out (min)
Parsed response rates
Pokes/min (log scale)

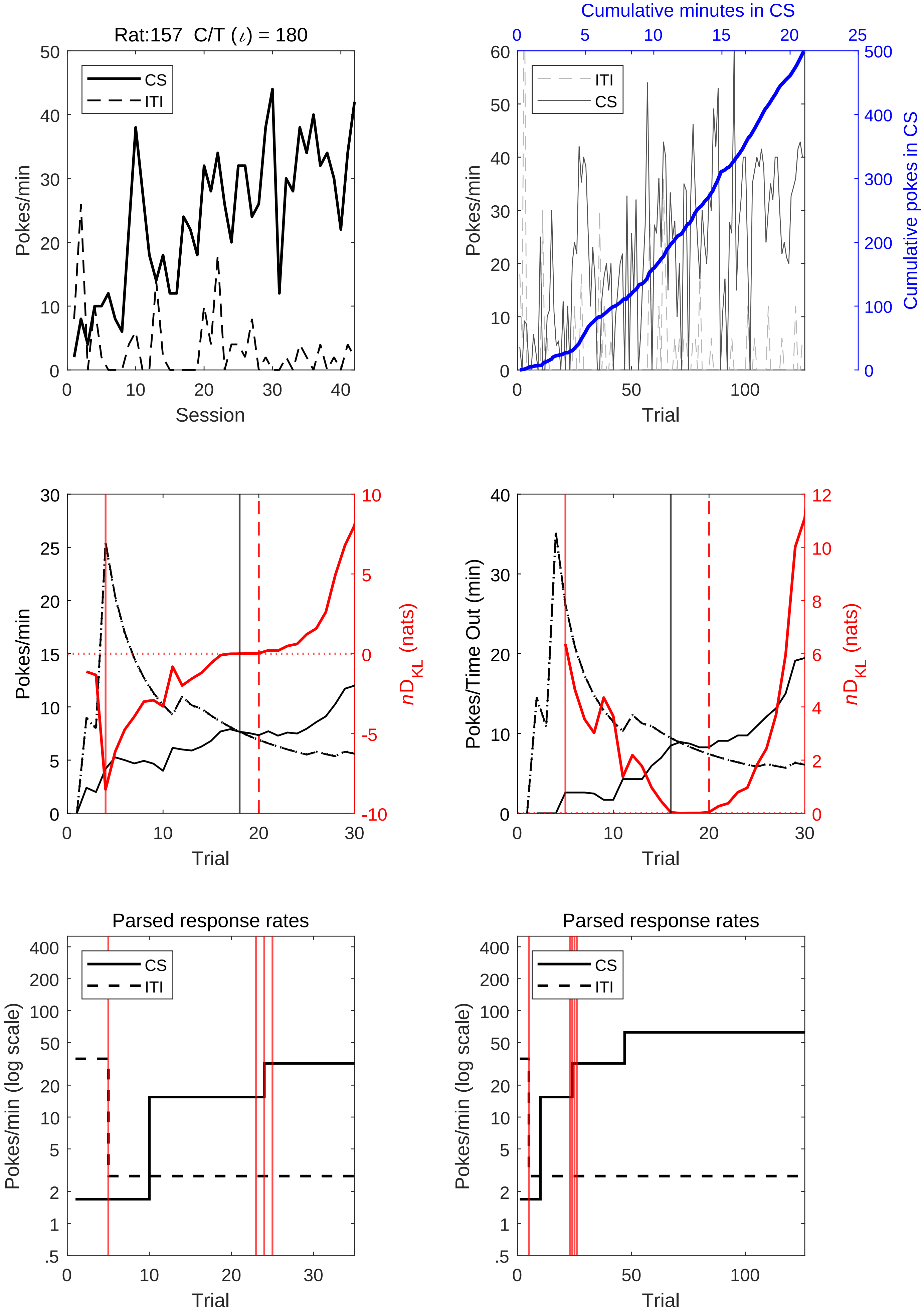

Rat:157 C/T (ι) = 180
CS
ITI
Pokes/min
Session
Cumulative minutes in CS
Cumulative pokes in CS
Trial
nD_KL (nats)
Pokes/Time Out (min)
Parsed response rates
Pokes/min (log scale)

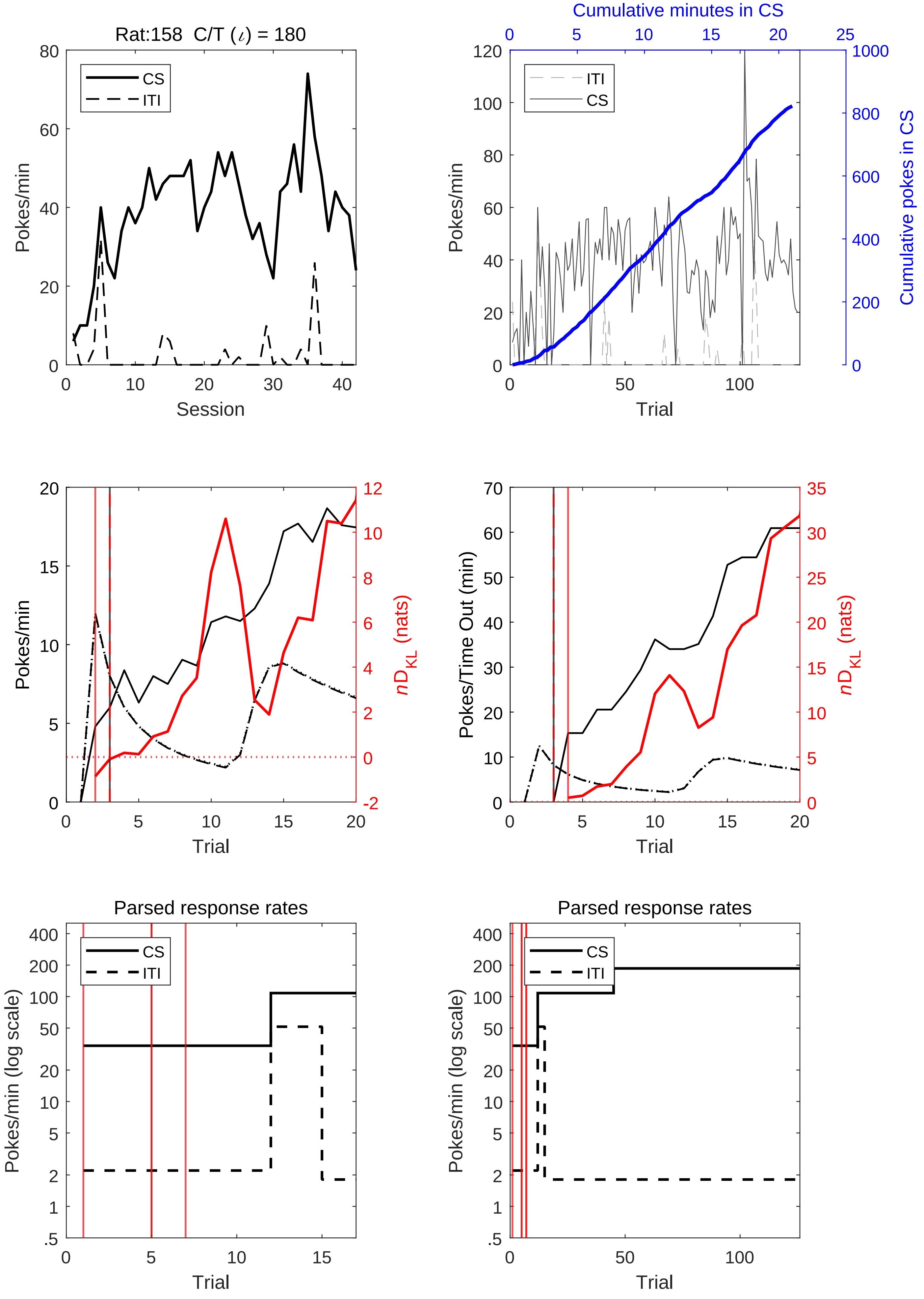

Rat:158 C/T ($\iota$) = 180
CS
ITI
Pokes/min
Session
Cumulative minutes in CS
ITI
CS
Pokes/min
Cumulative pokes in CS
Trial
Pokes/min
$nD_{KL}$ (nats)
Trial
Pokes/Time Out (min)
$nD_{KL}$ (nats)
Trial
Parsed response rates
CS
ITI
Pokes/min (log scale)
Trial
Parsed response rates
CS
ITI
Pokes/min (log scale)
Trial

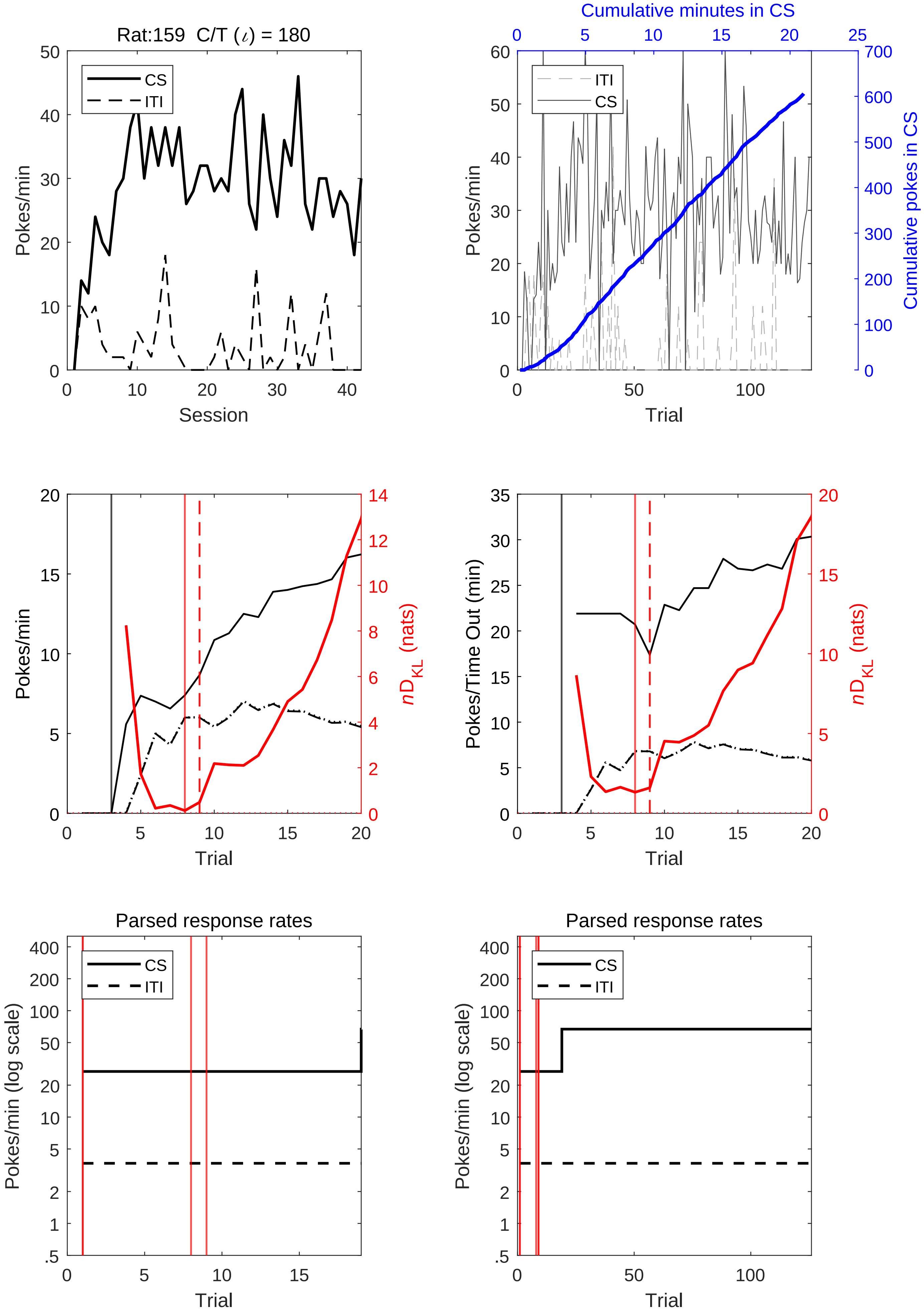
Rat:159 C/T (ι) = 180
CS
ITI
Pokes/min
Session
Cumulative minutes in CS
Cumulative pokes in CS
ITI
CS
Pokes/min
Trial
Pokes/min
Trial
nD_KL (nats)
Pokes/Time Out (min)
Trial
nD_KL (nats)
Parsed response rates
CS
ITI
Pokes/min (log scale)
Trial
Parsed response rates
CS
ITI
Pokes/min (log scale)
Trial

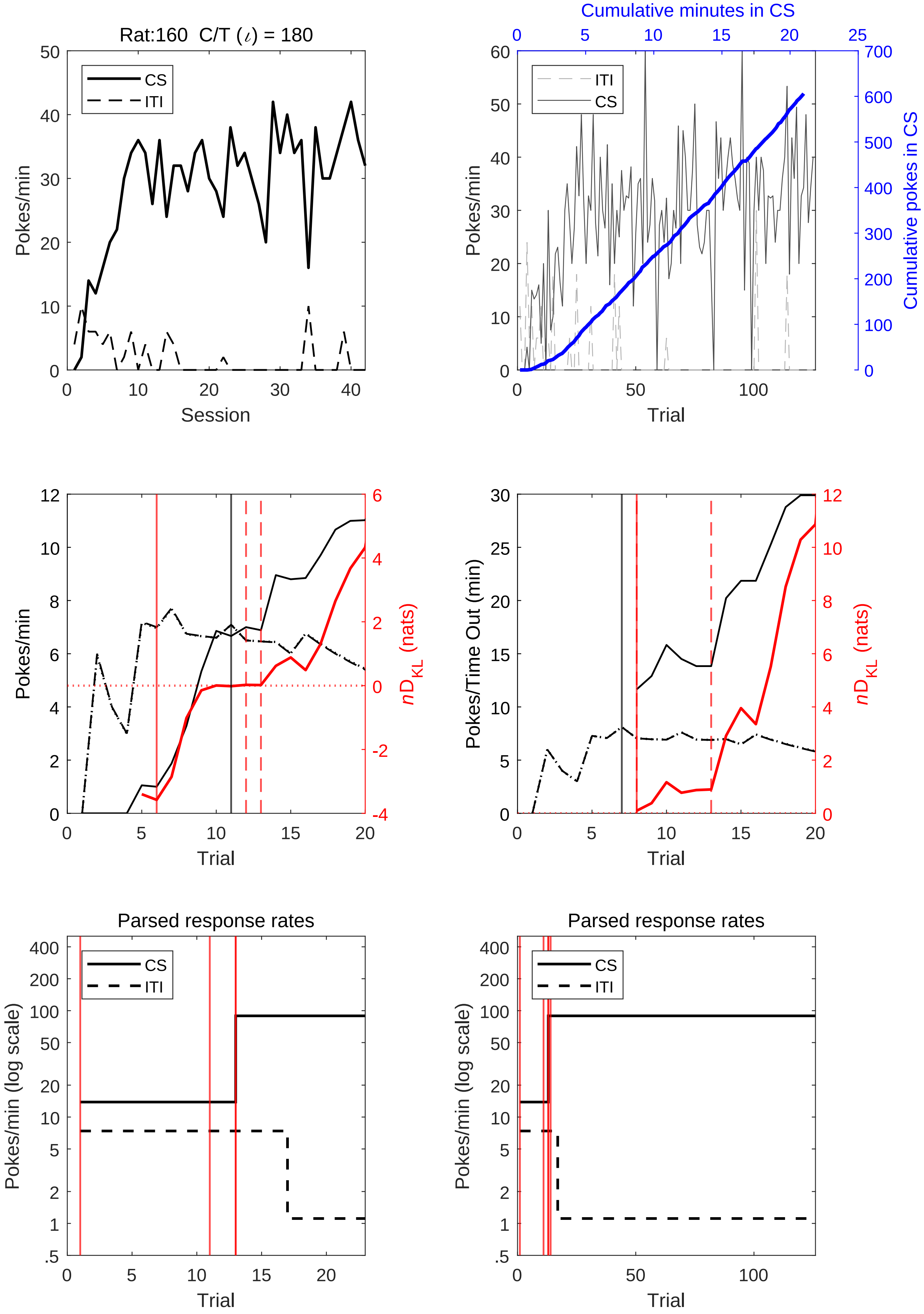

Rat:160 C/T (ι) = 180
Pokes/min
Session
CS
ITI
Cumulative minutes in CS
Cumulative pokes in CS
Trial
nD_KL (nats)
Pokes/Time Out (min)
Parsed response rates
Pokes/min (log scale)

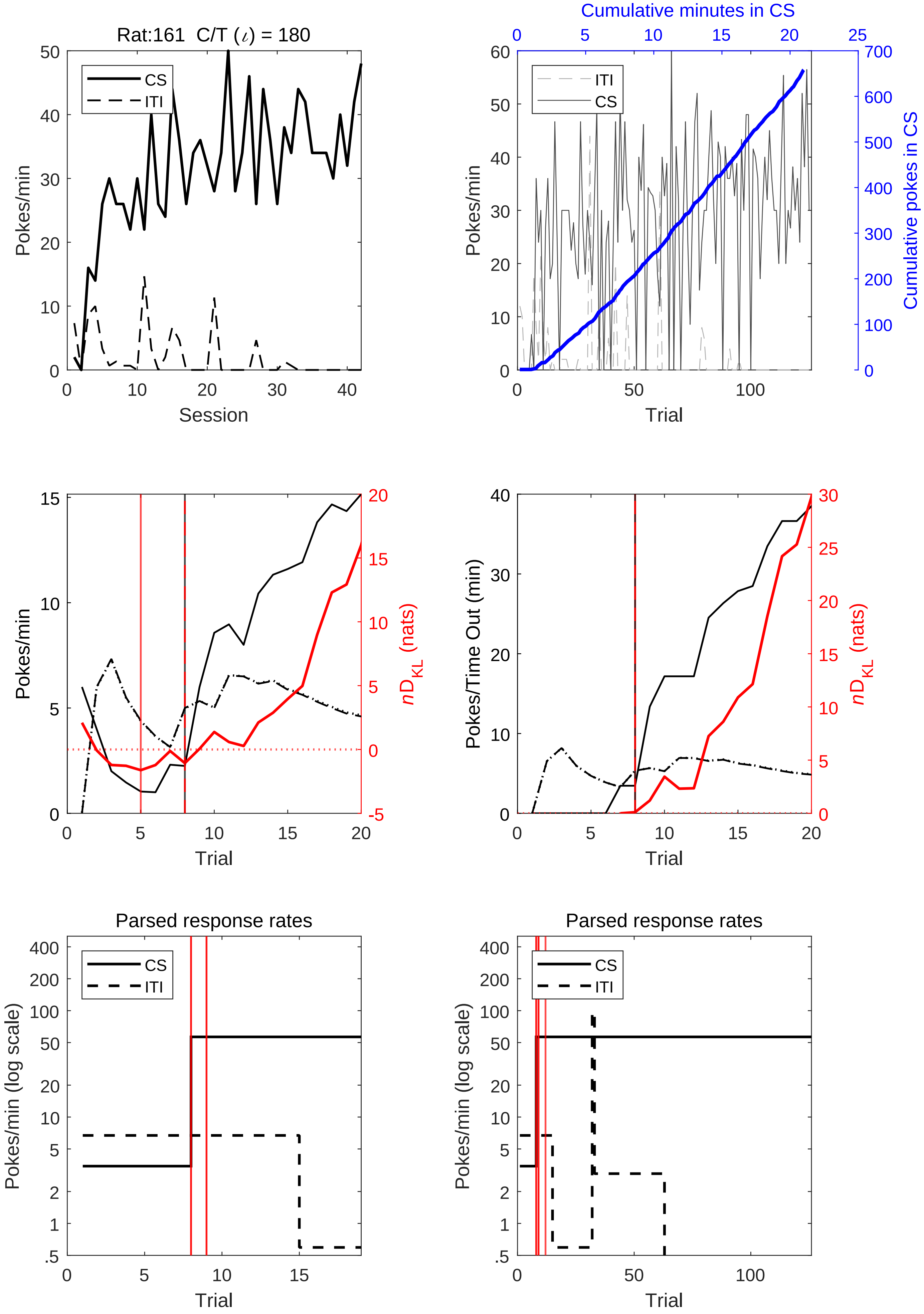

Rat:161 C/T ($\iota$) = 180
CS
ITI
Pokes/min
Session
Cumulative minutes in CS
Cumulative pokes in CS
Trial
$nD_{KL}$ (nats)
Pokes/Time Out (min)
Parsed response rates
Pokes/min (log scale)

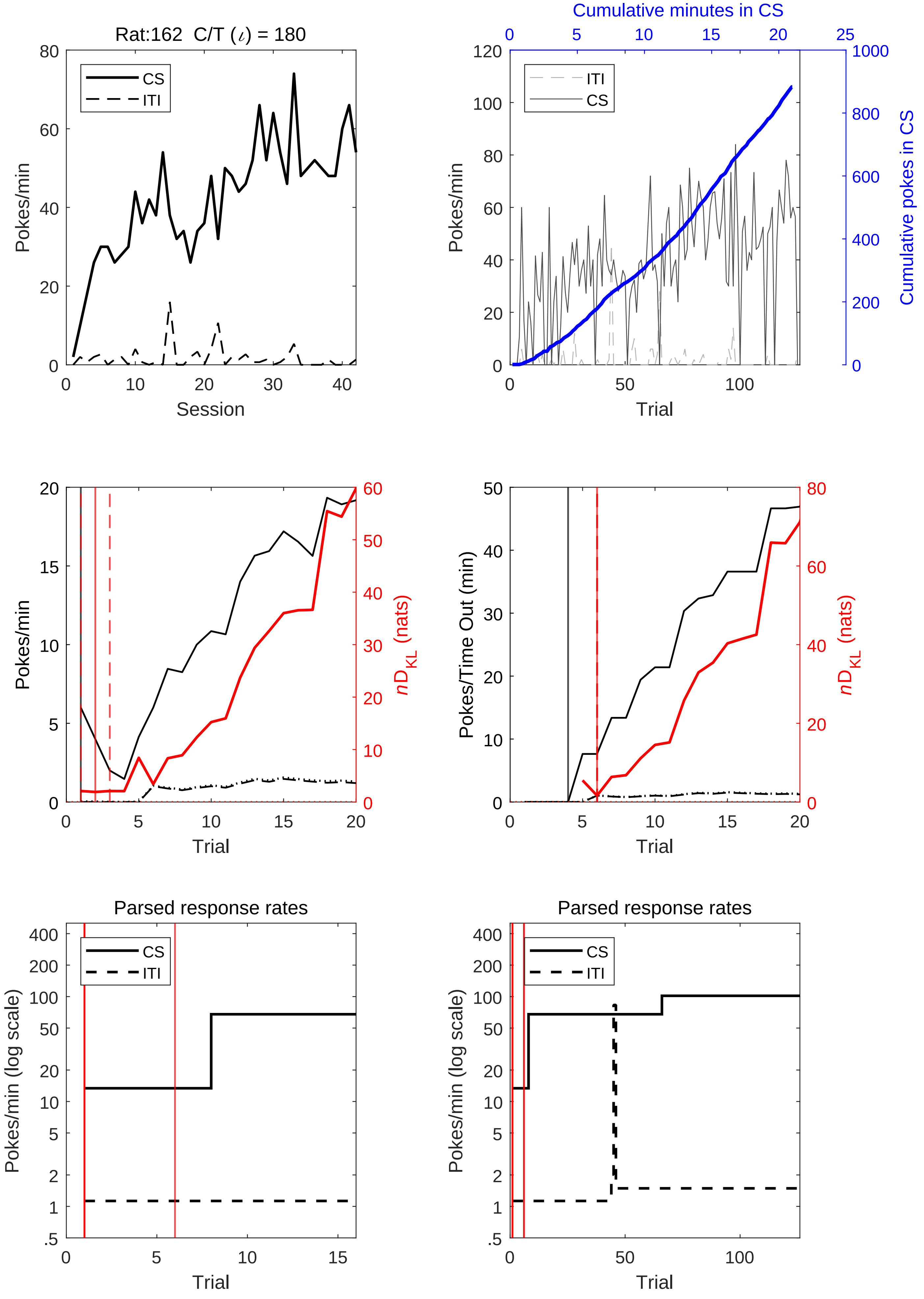

Rat:162 C/T (ι) = 180
CS
ITI
Pokes/min
Session
Cumulative minutes in CS
Cumulative pokes in CS
Trial
nD_KL (nats)
Pokes/Time Out (min)
Parsed response rates
Pokes/min (log scale)

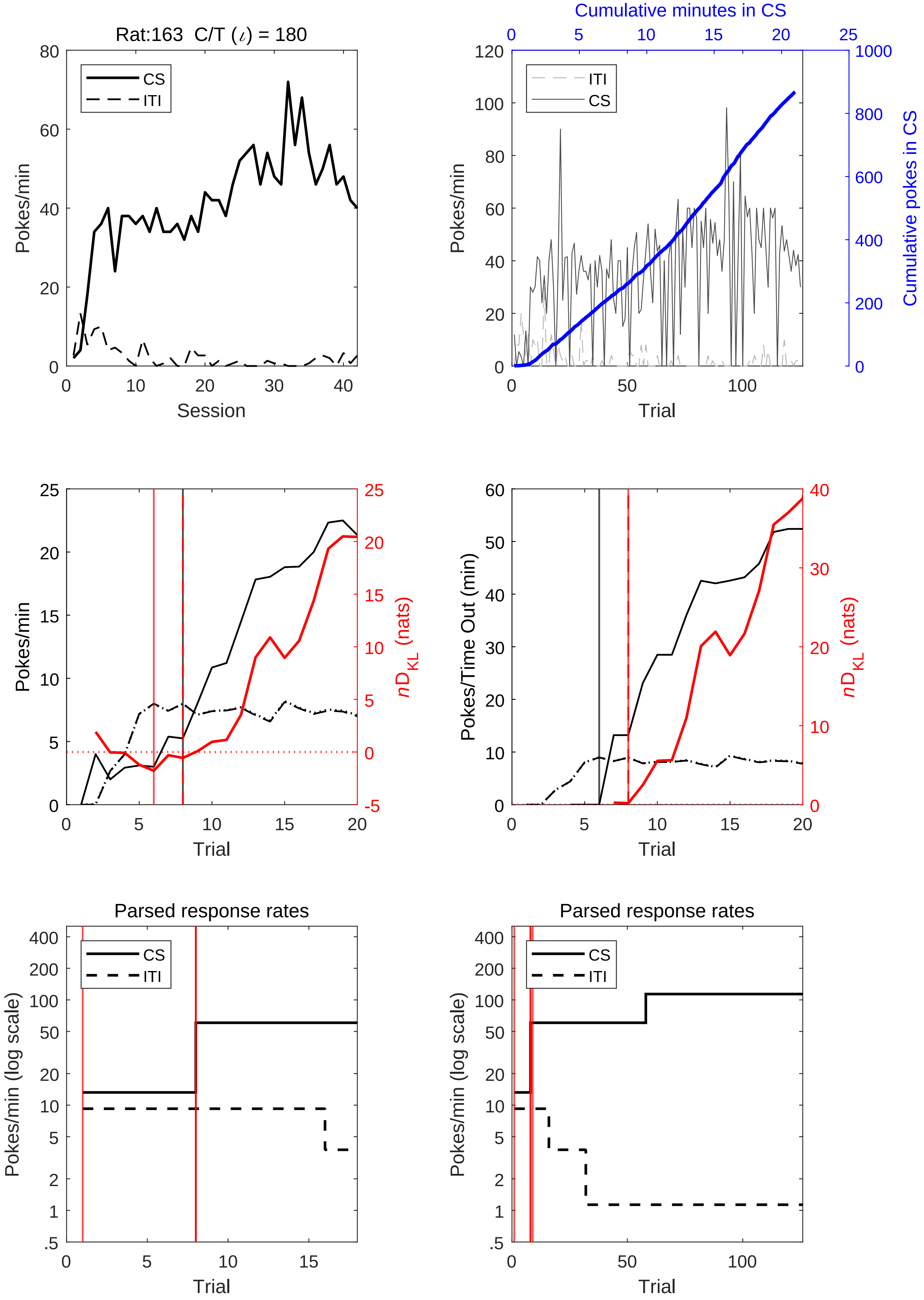

Rat:163 C/T (ι) = 180
CS
ITI
Pokes/min
Session
Cumulative minutes in CS
Cumulative pokes in CS
Trial
Pokes/Time Out (min)
nD_KL (nats)
Parsed response rates
Pokes/min (log scale)

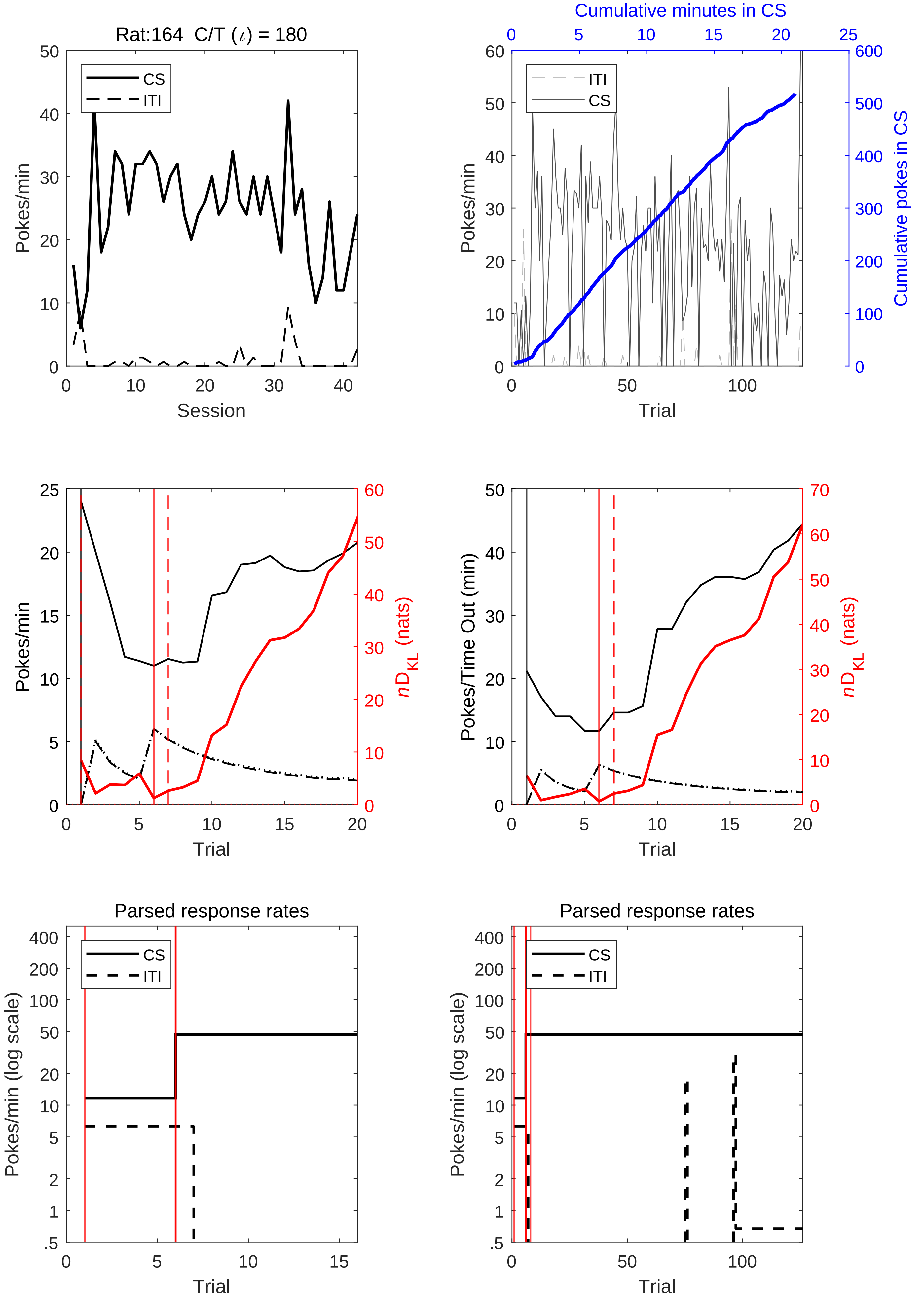

Rat:164 C/T (ι) = 180
CS
ITI
Pokes/min
Session
Cumulative minutes in CS
Cumulative pokes in CS
Trial
nD_KL (nats)
Pokes/Time Out (min)
Parsed response rates
Pokes/min (log scale)

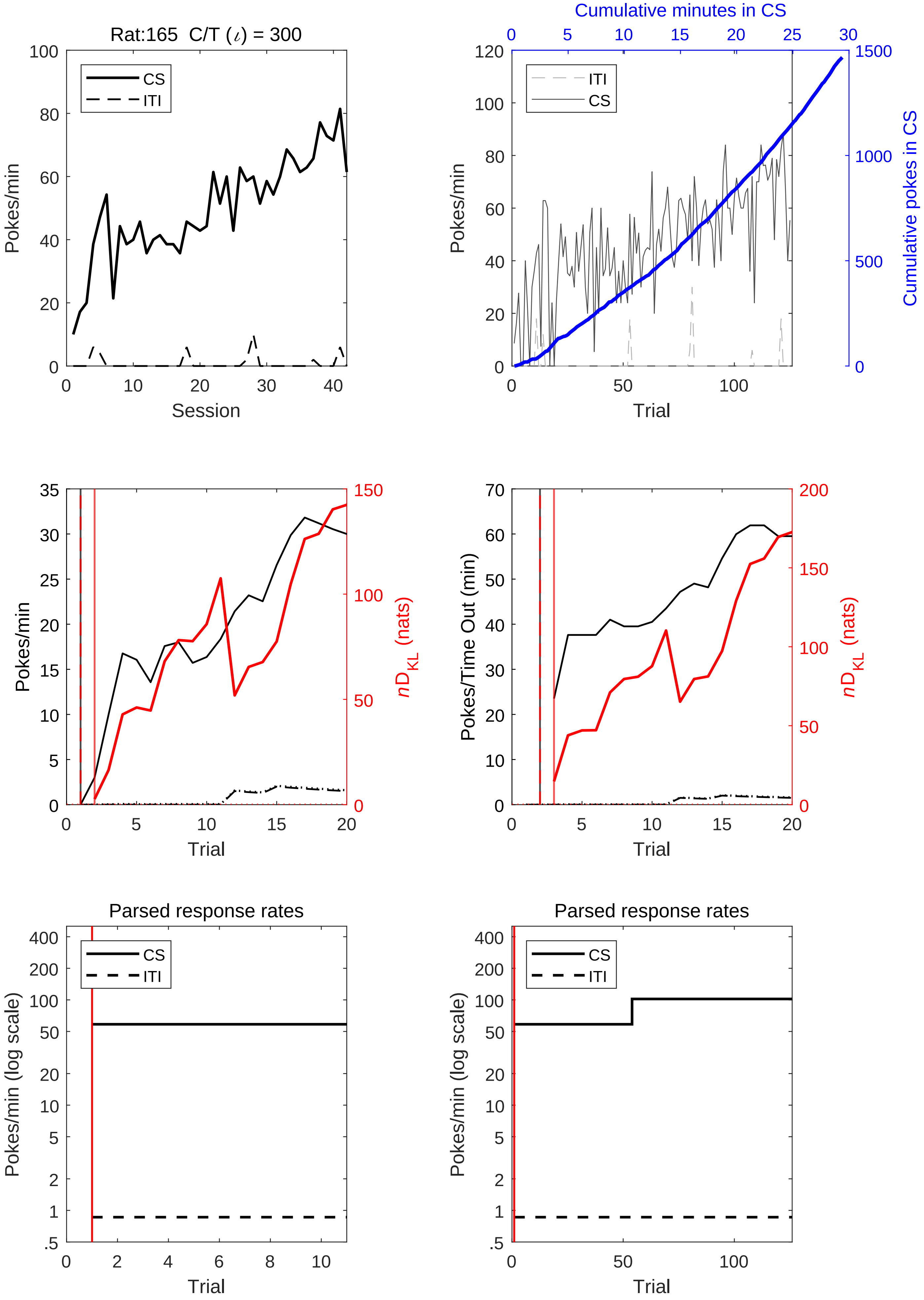
Rat:165 C/T (ι) = 300
Pokes/min
Session
CS
ITI
Cumulative minutes in CS
Cumulative pokes in CS
Trial
Pokes/Time Out (min)
nD_KL (nats)
Parsed response rates
Pokes/min (log scale)

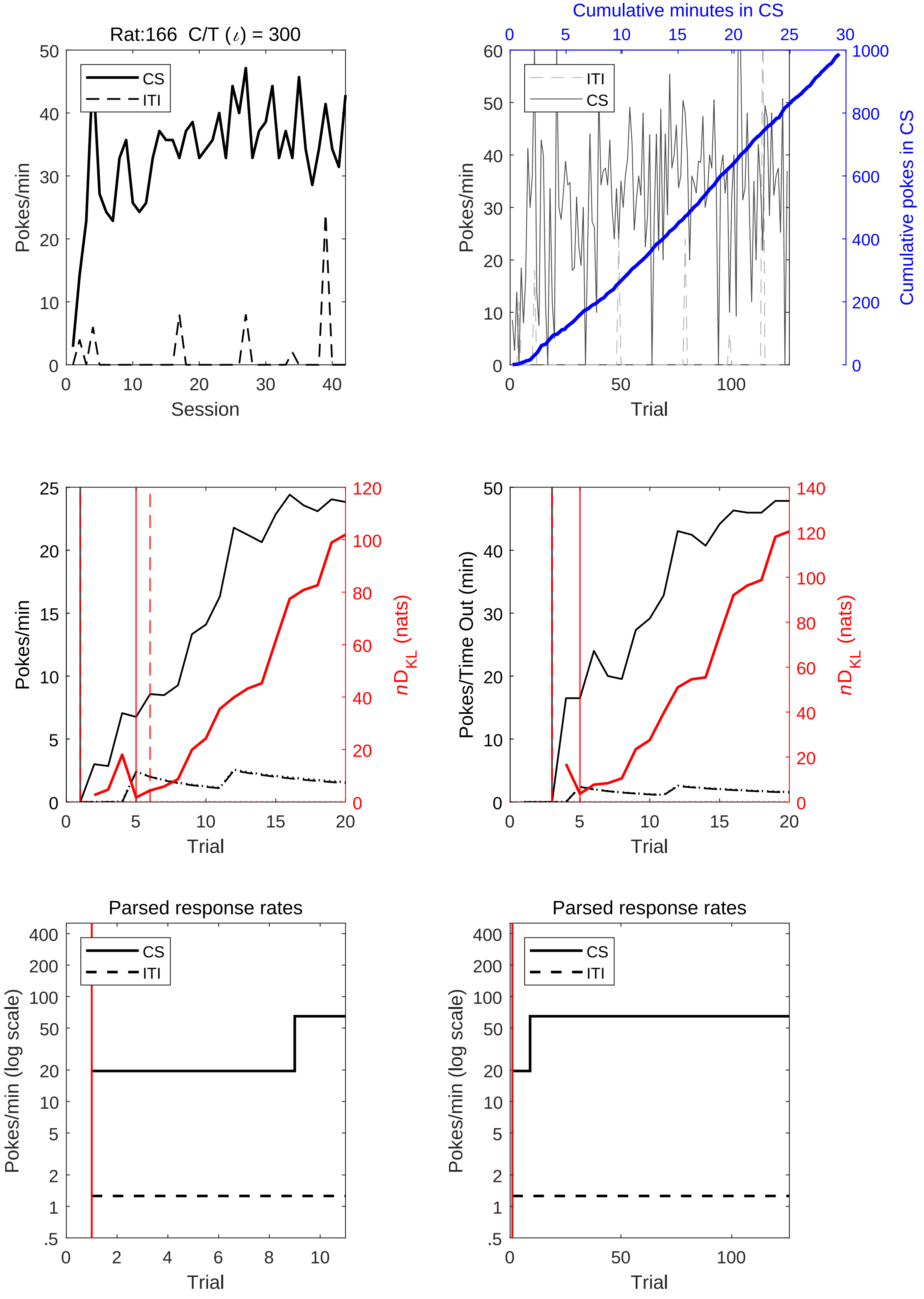

Rat:166 C/T (ι) = 300
CS
ITI
Pokes/min
Session
Cumulative minutes in CS
Cumulative pokes in CS
Trial
nD_KL (nats)
Pokes/Time Out (min)
Parsed response rates
Pokes/min (log scale)

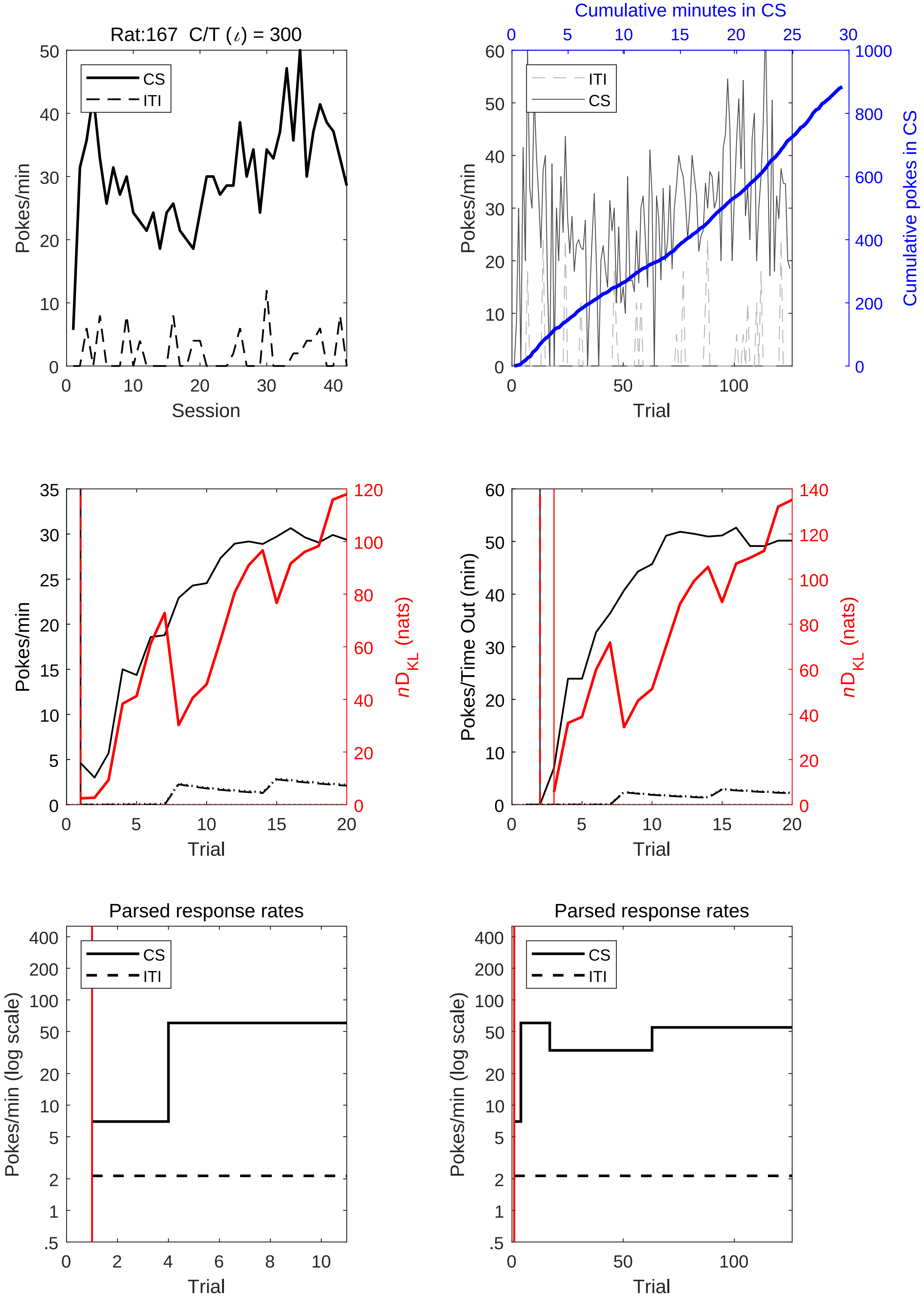
Rat:167 C/T (ι) = 300
CS
ITI
Pokes/min
Session
Cumulative minutes in CS
Pokes/min
Cumulative pokes in CS
Trial
Pokes/min
$nD_{KL}$ (nats)
Trial
Pokes/Time Out (min)
$nD_{KL}$ (nats)
Trial
Parsed response rates
Pokes/min (log scale)
Trial
Parsed response rates
Pokes/min (log scale)
Trial

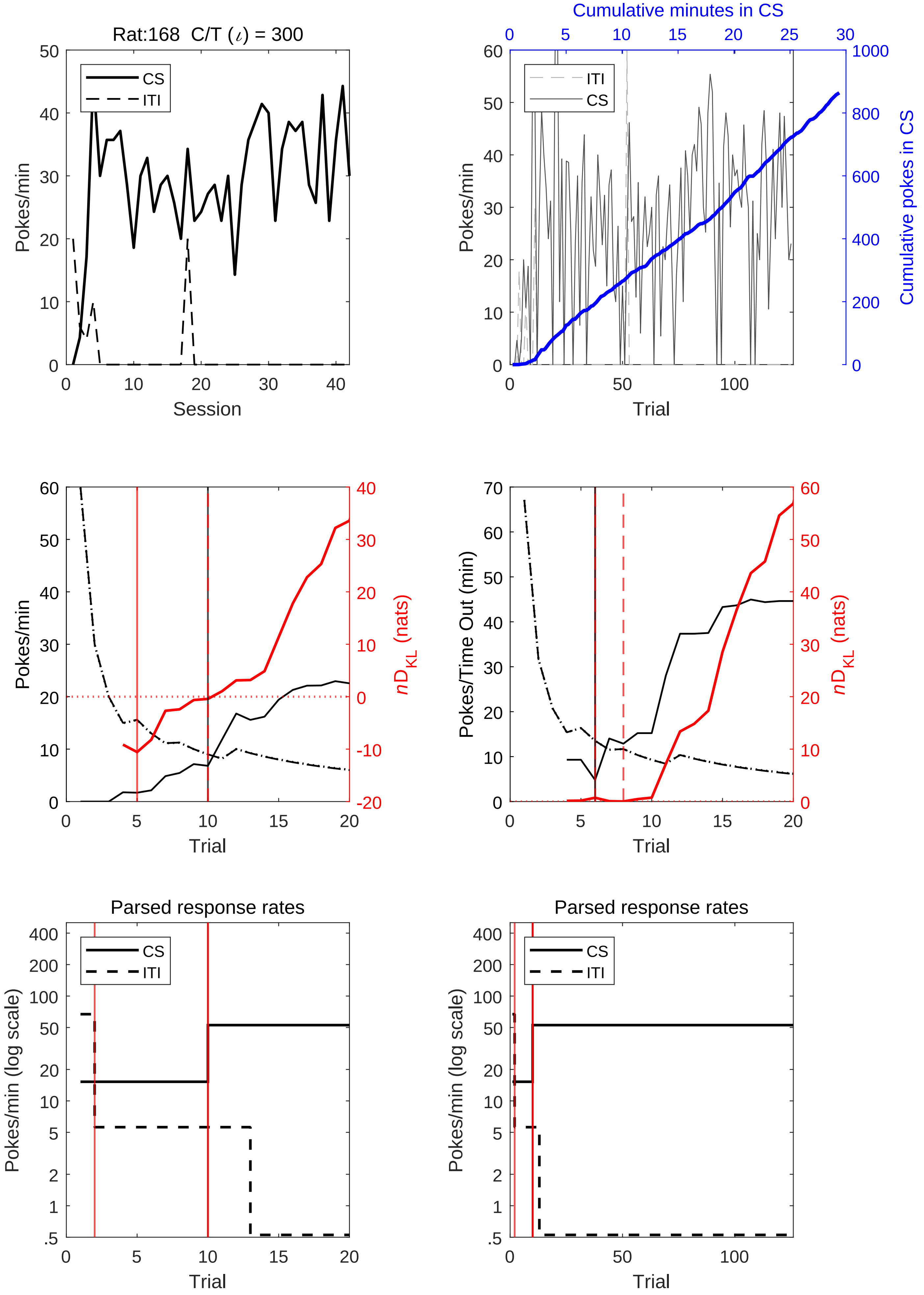
Rat:168 C/T (ι) = 300
CS
ITI
Pokes/min
Session
Cumulative minutes in CS
Cumulative pokes in CS
Trial
nD_KL (nats)
Pokes/Time Out (min)
Parsed response rates
Pokes/min (log scale)

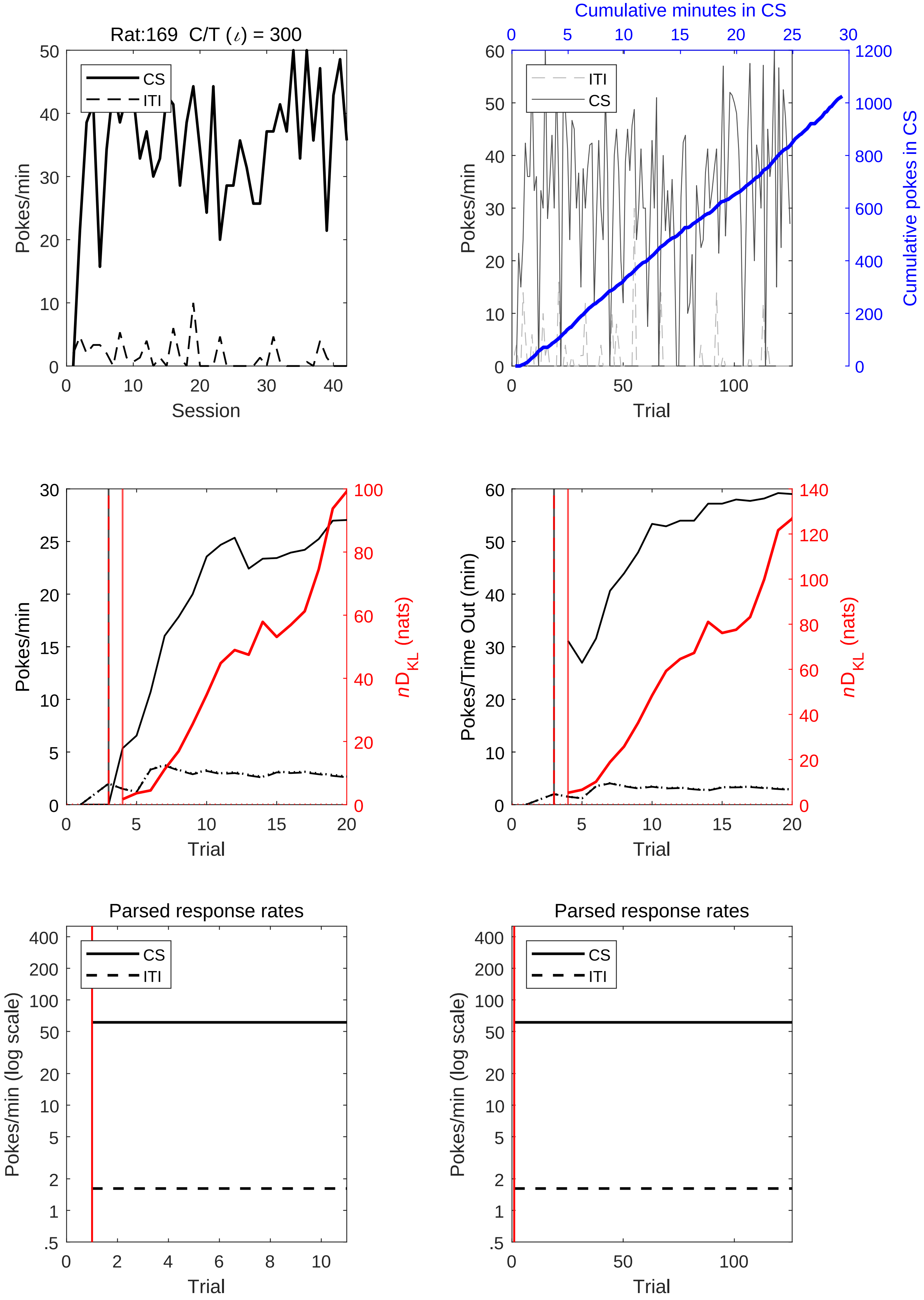
Rat:169 C/T (ι) = 300
CS
ITI
Pokes/min
Session
Cumulative minutes in CS
Pokes/min
Cumulative pokes in CS
Trial
Pokes/min
nD_KL (nats)
Trial
Pokes/Time Out (min)
nD_KL (nats)
Trial
Parsed response rates
Pokes/min (log scale)
Trial
Parsed response rates
Pokes/min (log scale)
Trial

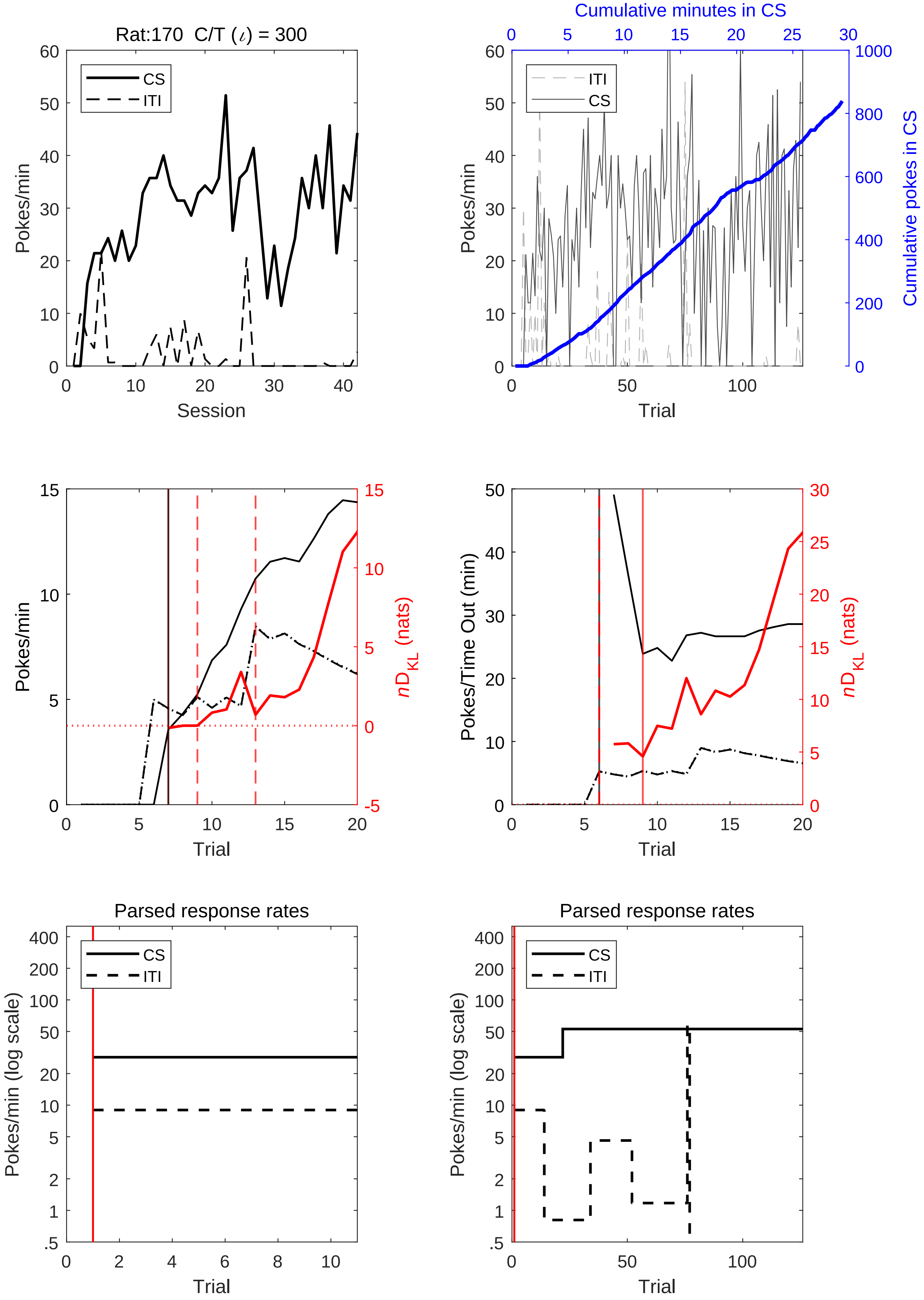
Rat:170 C/T (ι) = 300
CS
ITI
Pokes/min
Session
Cumulative minutes in CS
Cumulative pokes in CS
Trial
nD_KL (nats)
Pokes/Time Out (min)
Parsed response rates
Pokes/min (log scale)

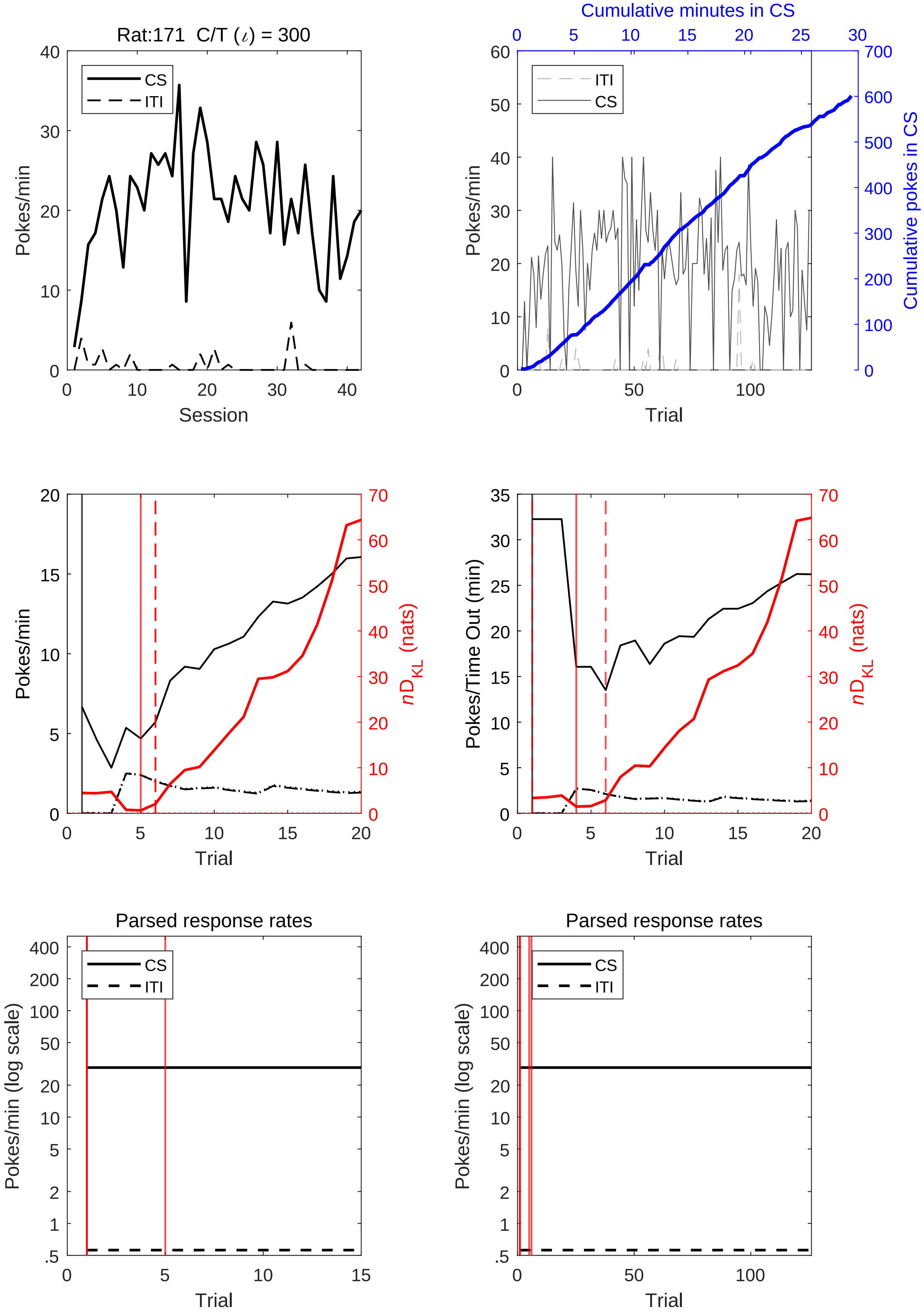

Rat:171 C/T ($\iota$) = 300
CS
ITI
Pokes/min
Session
Cumulative minutes in CS
ITI
CS
Pokes/min
Cumulative pokes in CS
Trial
Pokes/min
$nD_{KL}$ (nats)
Trial
Pokes/Time Out (min)
$nD_{KL}$ (nats)
Trial
Parsed response rates
CS
ITI
Pokes/min (log scale)
Trial
Parsed response rates
CS
ITI
Pokes/min (log scale)
Trial

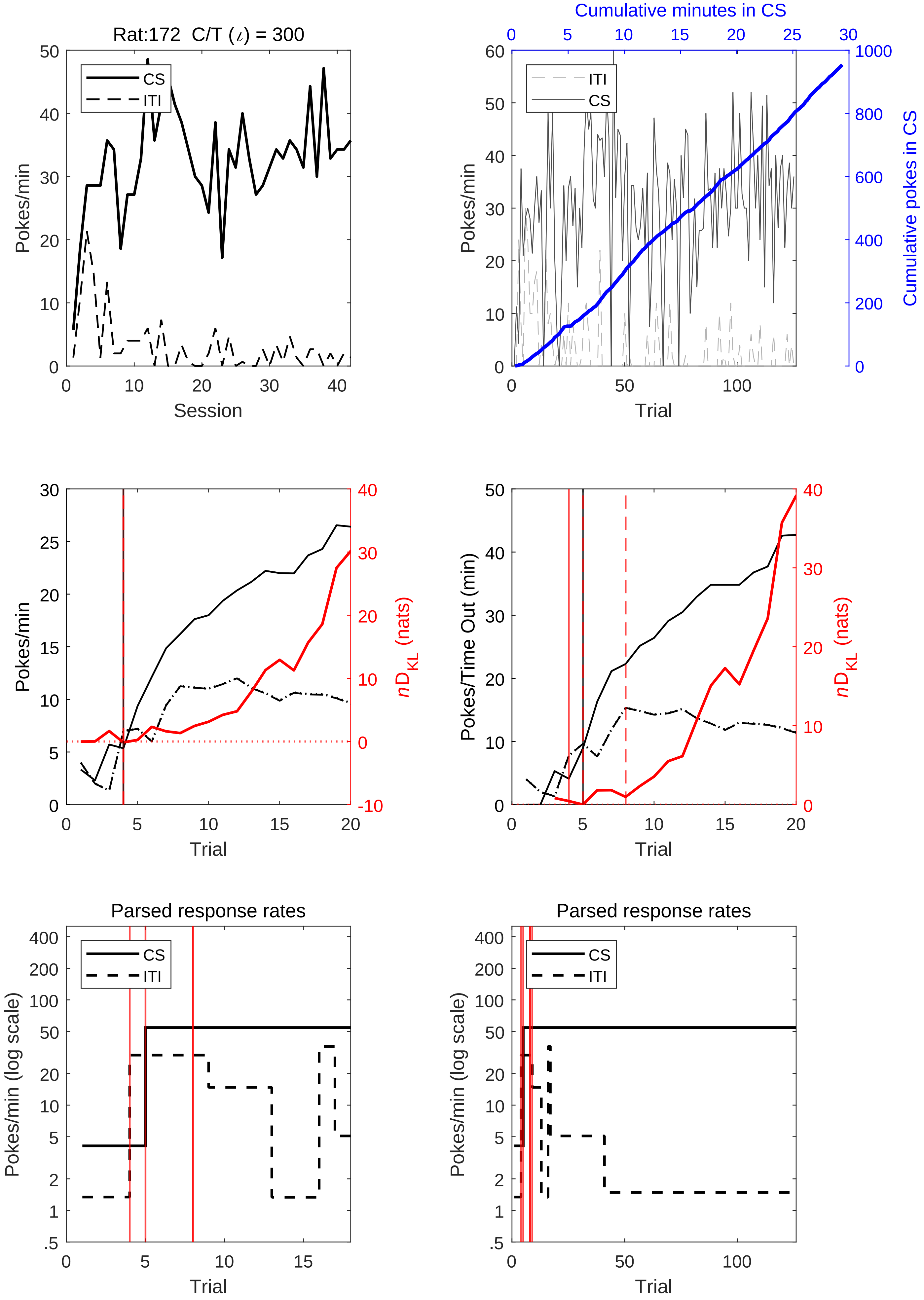
Rat:172 C/T (ι) = 300
Pokes/min
Session
CS
ITI
Cumulative minutes in CS
Cumulative pokes in CS
Trial
Pokes/Time Out (min)
nD_KL (nats)
Parsed response rates
Pokes/min (log scale)

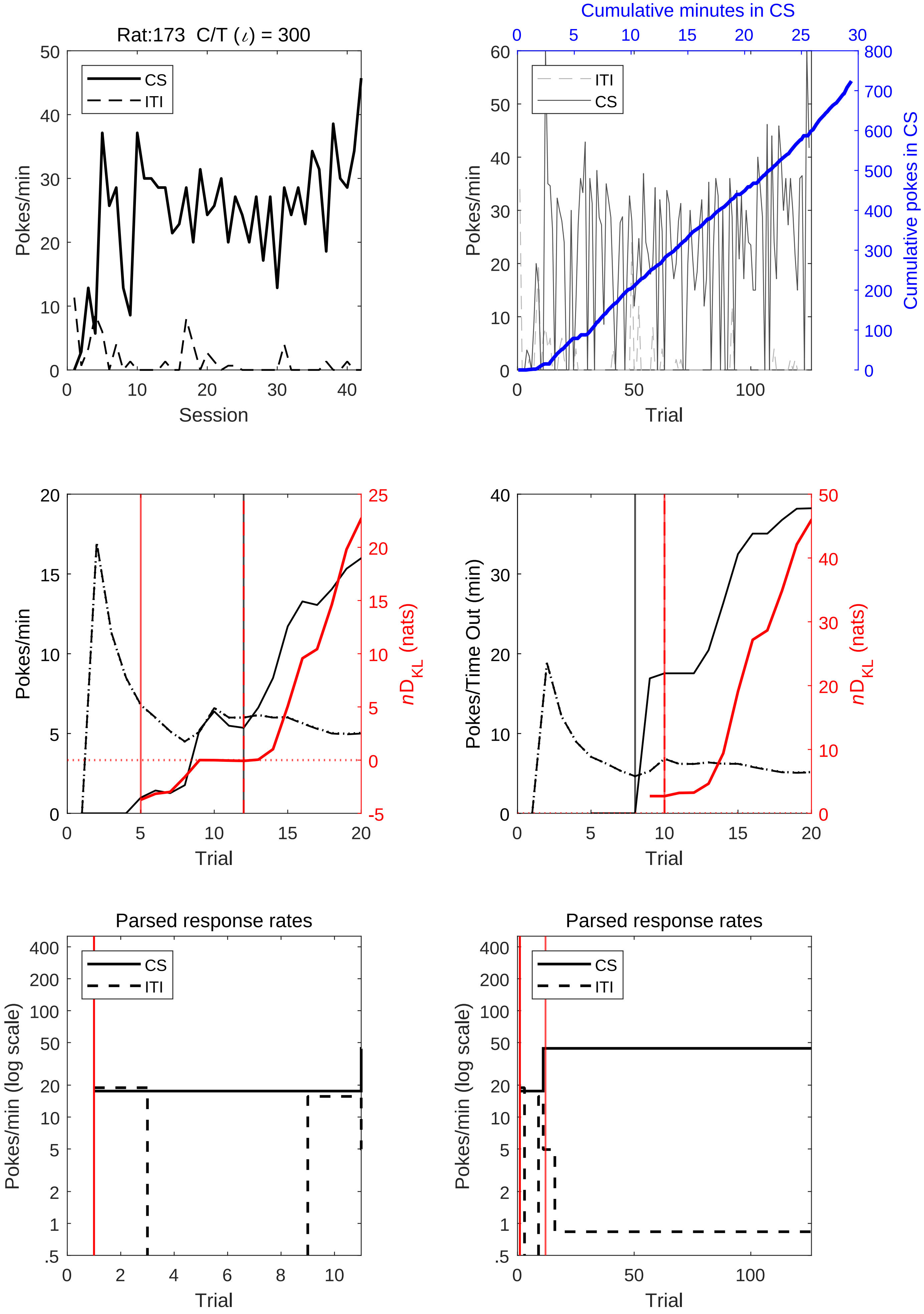
Rat:173 C/T (ι) = 300
CS
ITI
Pokes/min
Session
Cumulative minutes in CS
Cumulative pokes in CS
Trial
nD_KL (nats)
Pokes/Time Out (min)
Parsed response rates
Pokes/min (log scale)

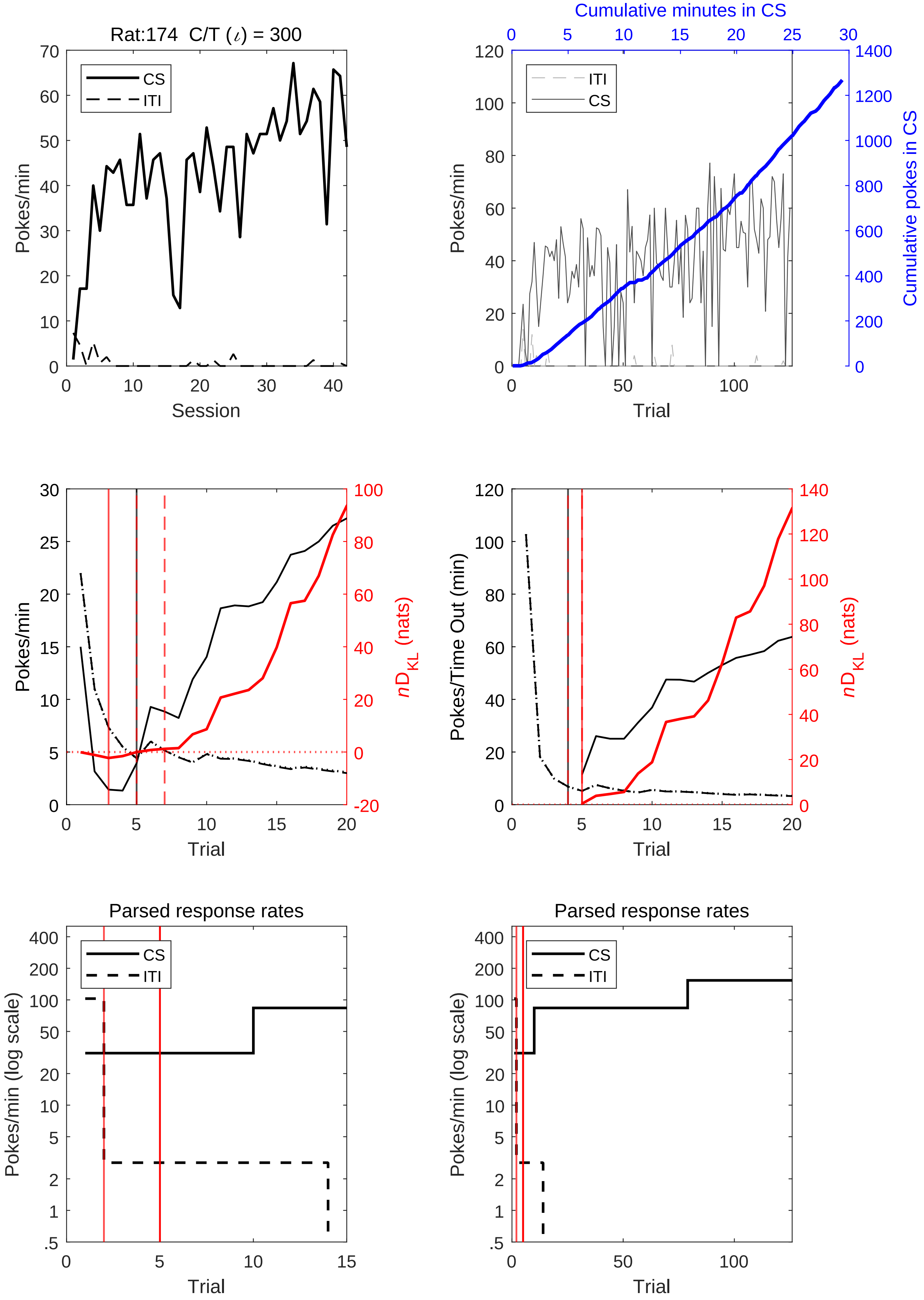
Rat:174 C/T (ι) = 300
CS
ITI
Pokes/min
Session
Cumulative minutes in CS
Cumulative pokes in CS
Trial
Pokes/Time Out (min)
nD_KL (nats)
Parsed response rates
Pokes/min (log scale)

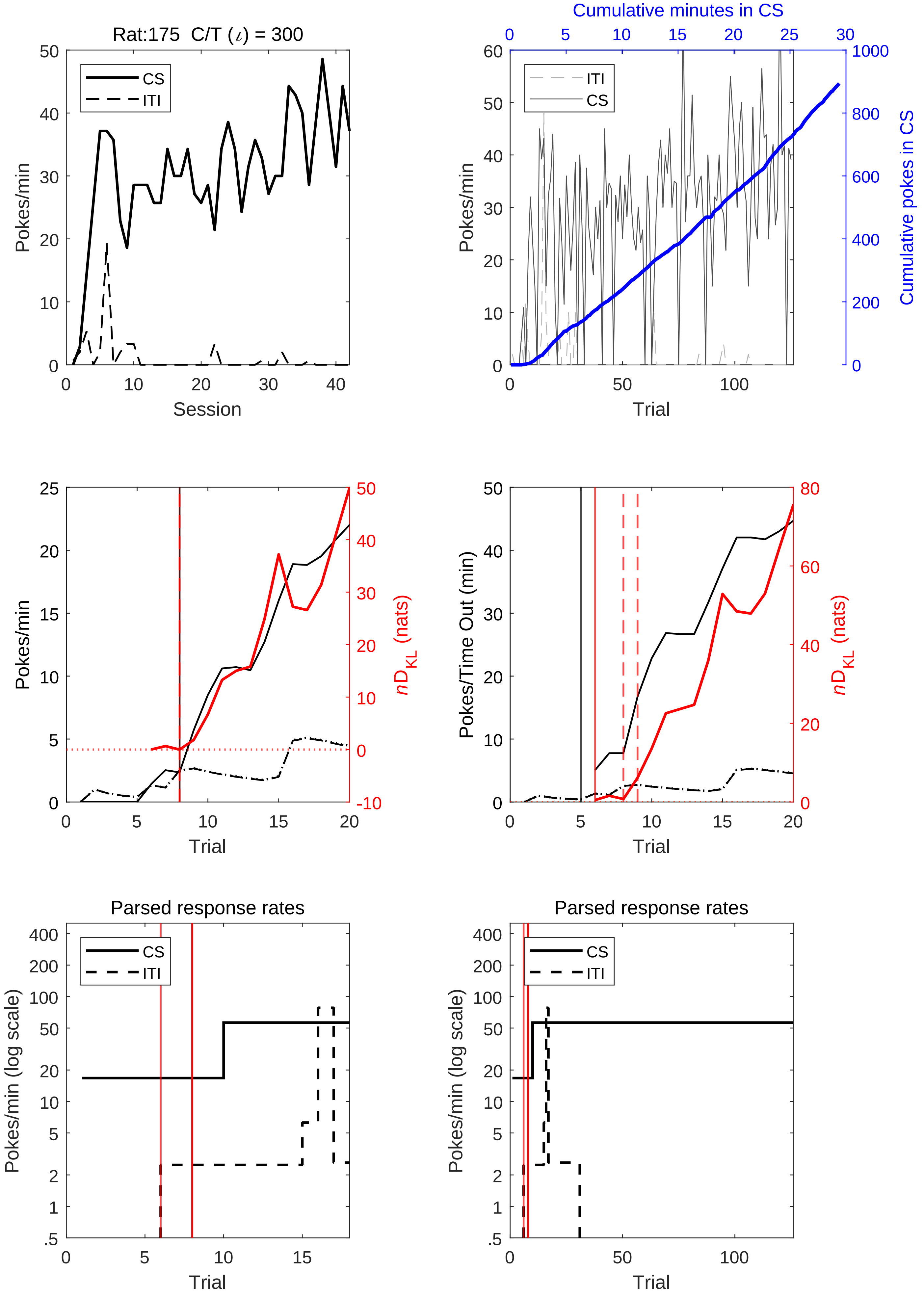
Rat:175 C/T ($\iota$) = 300
CS
ITI
Pokes/min
Session
Cumulative minutes in CS
Cumulative pokes in CS
Trial
$nD_{KL}$ (nats)
Pokes/Time Out (min)
Parsed response rates
Pokes/min (log scale)

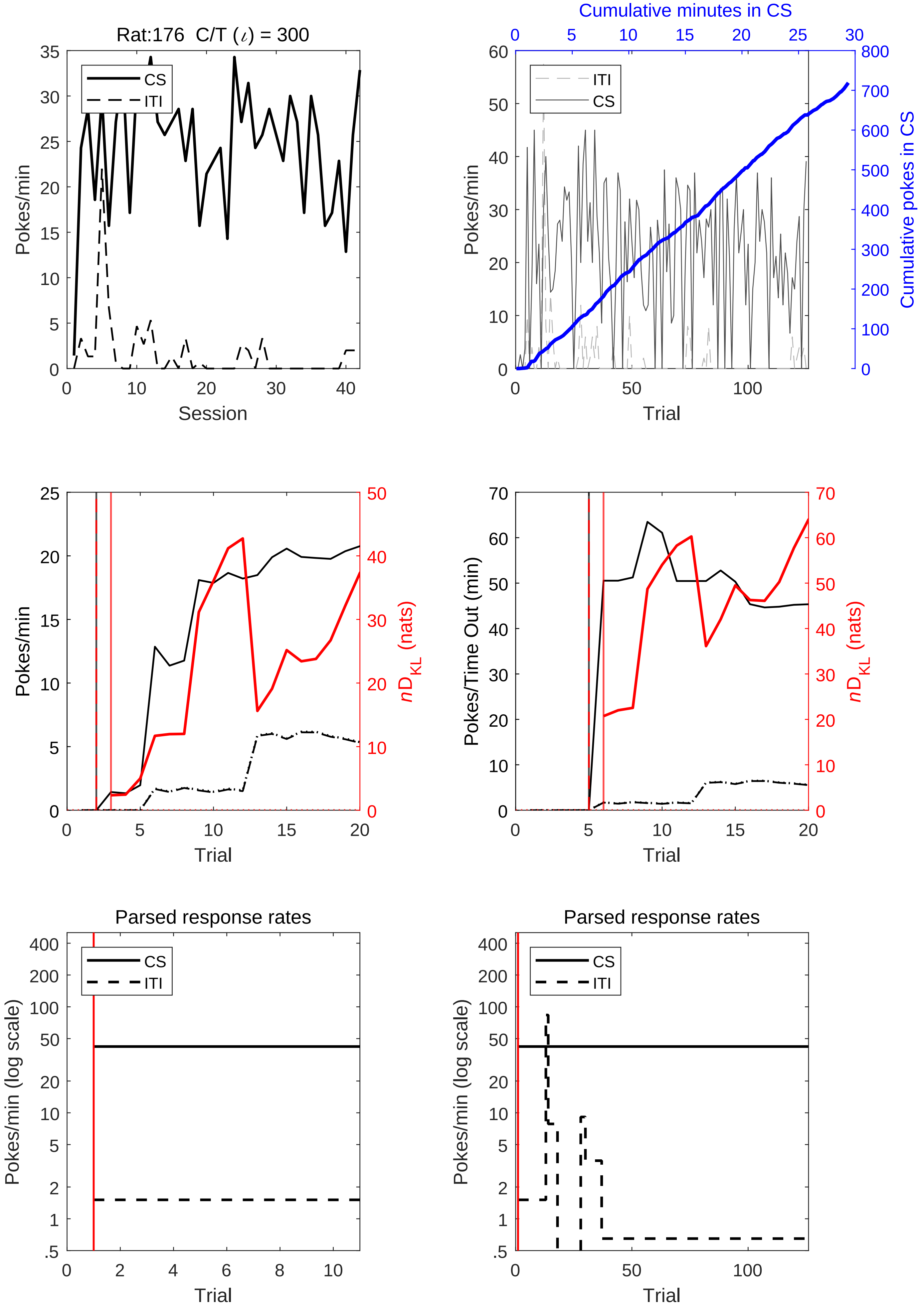
Rat:176 C/T (ι) = 300
CS
ITI
Pokes/min
Session
Cumulative minutes in CS
Cumulative pokes in CS
Trial
nD_KL (nats)
Pokes/Time Out (min)
Parsed response rates
Pokes/min (log scale)
